# Three Essential Ribonucleases—RNase Y, J1, and III—Control the Abundance of a Majority of *Bacillus subtilis* mRNAs

**DOI:** 10.1371/journal.pgen.1002520

**Published:** 2012-03-08

**Authors:** Sylvain Durand, Laetitia Gilet, Philippe Bessières, Pierre Nicolas, Ciarán Condon

**Affiliations:** 1 CNRS UPR 9073, University Paris Diderot, Sorbonne Paris Cité, Institut de Biologie Physico-Chimique, Paris, France; 2 INRA UR1077, Mathématique Informatique et Génome, Jouy en Josas, France; Hebrew University, Israel

## Abstract

*Bacillus subtilis* possesses three essential enzymes thought to be involved in mRNA decay to varying degrees, namely RNase Y, RNase J1, and RNase III. Using recently developed high-resolution tiling arrays, we examined the effect of depletion of each of these enzymes on RNA abundance over the whole genome. The data are consistent with a model in which the degradation of a significant number of transcripts is dependent on endonucleolytic cleavage by RNase Y, followed by degradation of the downstream fragment by the 5′–3′ exoribonuclease RNase J1. However, many full-size transcripts also accumulate under conditions of RNase J1 insufficiency, compatible with a model whereby RNase J1 degrades transcripts either directly from the 5′ end or very close to it. Although the abundance of a large number of transcripts was altered by depletion of RNase III, this appears to result primarily from indirect transcriptional effects. Lastly, RNase depletion led to the stabilization of many low-abundance potential regulatory RNAs, both in intergenic regions and in the antisense orientation to known transcripts.

## Author Summary

RNA turnover is an important way of controlling gene expression. While the characterization of the pathways and enzymes for RNA degradation are well-advanced in *Escherichia coli* and yeast, studies in Gram-positive bacteria have lagged behind. This tiling array study shows that two essential enzymes, the single-strand specific endonuclease RNase Y and the 5′–3′ exoribonuclease RNase J1, play central roles in the degradation of mRNAs in *Bacillus subtilis*. The double-strand specific enzyme RNase III plays a more minor role in RNA turnover, but has significant indirect effects on transcription. Depleting cells of these key enzymes led to the stabilization of many potentially regulatory RNAs, which were otherwise revealed only through testing a wide variety of experimental conditions. It is now possible to tell at a glance which of these three RNases is involved in the turnover of your favorite mRNA.

## Introduction

The amount of a particular mRNA in the cell is a function of the equilibrium between its synthesis and degradation. The pathways of RNA degradation are fairly well defined in the Gram-negative model bacterium *Escherichia coli* and in the eukaryotic paradigm *Saccharyomyces cerevisiae*. In *E. coli*, degradation of RNA is primarily dependent on the single-strand specific endonuclease RNase E, followed by degradation of the resulting fragments by 3′-5′ exoribonucleases (for recent review, see [1]). RNase E can either attack primary transcripts directly [2] or, in a more efficient reaction, following conversion of the 5′ triphosphate moiety to a monophosphate group by the RNA pyrophosphohydrolase RppH [3]. In yeast, mRNA is primarily degraded by exonucleases following removal of the methylguanosine ‘cap’ from the 5′ end (for recent review see [4]). The exoribonuclease Xrn1 operates in the 5′-3′ orientation, while the exosome complex degrades RNA from the 3′ end. Recent evidence has also indicated a role for endonucleolytic cleavages in the decay of some yeast mRNAs (for recent review see [5]).

Two pathways for RNA degradation have so far been characterized in the Gram-positive model organism *B. subtilis* (for recent review, see [6]). The first relies on cleavage of the mRNA by an endonuclease followed by degradation of the resulting fragments by exonucleases, similar to the *E. coli* model, but with different enzymes. The membrane-bound protein RNase Y has emerged as a major candidate for the endonucleolytic step [7]–[9], while the double-strand specific nuclease RNase III is a candidate for a minor role [10], [11]. Following endonucleolytic cleavage, the upstream fragment becomes a substrate of 3′-5′ exonucleases, principally PNPase [12], [13], while the downstream fragment is a target for the 5′-3′ exoribonuclease activity of RNase J1, as part of a complex with its non-essential and poorly active paralog RNase J2 [14], [15]. The RNase J1/J2 complex has been proposed to be part of an even larger assembly including RNase Y, PNPase and some glycolytic enzymes [7], but this has been the subject of some discussion [15]. In the second pathway, RNase J1/J2 attacks full-length primary transcripts once the 5′ triphosphate group has been converted to a 5′ monophosphate by the *B. subtilis* ortholog of RNA pyrophosphohydrolase, BsRppH, or a related enzyme [16]. In theory, an exonucleolytic degradation pathway directly from the 3′ end could also exist, as in yeast, but is not thought to be prevalent due to the presence of protective terminator stem loop structures at the 3′ end of most *B. subtilis* mRNAs. However, *B. subtilis* is known to have a polyadenylation activity [17] which, in *E. coli* and other organisms, helps destabilize stem-loop structures by providing on-ramps for 3′-5′ exonucleases. The identity of the *B. subtilis* polyadenylation enzyme remains elusive, however [18].

Recent experiments have suggested a role for the essential ribonucleases RNase J1 and RNase Y in global mRNA degradation in *B. subtilis* [8], [19]. Because of the nature of its substrate specificity, the double-stand specific enzyme RNase III was anticipated to have a relatively minor function in general mRNA turnover, but perhaps play a more important role in the degradation of antisense RNAs. We studied the extent of the roles of RNase J1, Y and III, by examining the relative abundance of individual RNAs isolated from cells depleted for each of these enzymes, using recently developed tiling arrays with 22 nucleotide resolution [20].

## Results

### Experimental strategy

There are two possible philosophies with regard to how to perform depletion experiments with essential enzymes, each of which has its merits and drawbacks. One strategy is to only partially deplete cells, to a point where the enzyme becomes limiting for growth. While this has the advantage that measurements are made under steady state conditions and growth rate effects are minimized, only the most sensitive (i.e. lowest affinity) RNase substrates are detected using this approach. To detect the maximum number of potential substrates, more severe depletion conditions are required, but this strategy has the obvious complication that cells are undergoing a dramatic slow-down in growth and potentially putting an appropriate stress response in place. To allow us to detect the maximum number of substrates, while at the same time controlling for growth slow-down caused by a severe insufficiency of an essential RNase, we decided to measure the effects of RNase J1, Y and III depletions under the same experimental conditions. We reasoned that general stress effects linked to impending growth arrest would be similar in all three depleted strains, allowing us to distinguish between general and specific effects. In this way, we could identify RNA species affected by depletion of individual RNases or a combination of two enzymes.

For these experiments, we used strains in which expression of the RNase encoding gene (*rnjA*, *rny* or *rnc*, encoding RNase J1, Y and III, respectively) was placed under control of an IPTG-inducible P*spac* promoter. The P*spac-rnjA* construct has been described previously [21] and is integrated at the native locus (strain CCB034). We first used a similar P*spac*-controlled construct for the *rny* gene (strain CCB012). However, in an initial tiling array experiment using this strain we noticed that, despite the presence of a potential transcription terminator downstream of *rny*, there was a significant polar effect on the transcription of the adjacent *ymdB* gene in the absence of IPTG (Table S1). YmdB has recently shown to be involved in biofilm formation [22]. The *rnc* gene is similarly part of an operon, with two downstream genes, *smc* and *ftsY*. To avoid complications due to polar effects on genes downstream of *rny* and *rnc*, we therefore made strains in which the P*spac-rny* (CCB294) and P*spac-rnc* (CCB288) constructs were integrated at the *amyE* locus and where the coding sequence (CDS) of the native gene was replaced by the CDS of the spectinomycin resistance gene (*spc*). No polar effects were observed in either of these two strains (Table S1). All depletion strains also contained pMAP65, providing extra copies of the LacI repressor to ensure tight regulation of the P*spac* promoter.

### Depletion conditions result in at least a 30-fold reduction in each enzyme

**Figure 1:**
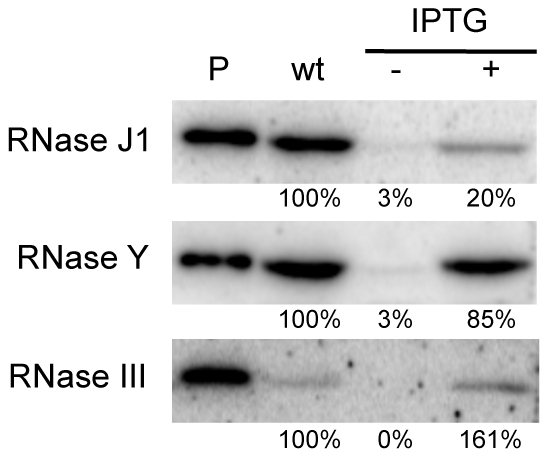
Western blot analysis of RNase depletion strains. Lane (P) shows signal from 50 ng purified RNase J1, RNase Y and RNase III proteins. Lanes labeled wt and −/+ IPTG show signals from 10 µg wild type and mutant cell extracts grown in the absence and presence of IPTG.

We first performed Western blots using specific antibodies to determine the relative levels of expression of each protein in wild-type and depleted CCB034, CCB294 and CCB288 strains. As observed previously, the fully induced (1 mM IPTG) P*spac-rnjA* construct produces about five-fold less RNase J1 than in wild-type cells (Figure 1). Under severe depletion conditions in the absence of IPTG, RNase J1 levels were decreased >30-fold reduced compared to wild-type cells. In the presence of IPTG, the P*spac-rny* construct produced very similar levels of RNase Y to wild-type cells, while the P*spac-rnc* construct slightly overproduced (1.6-fold) RNase III. In the absence of IPTG, the expression of both of these constructs was reduced by >30-fold compared to wild-type cells. Because the levels of expression of RNase J1 and RNase III in the presence of IPTG were different to those found in wild-type cells, we decided to compare wild-type expression levels of each RNA with those observed in the absence of IPTG. In this way, we consistently compare wild-type RNase levels with a >30-fold reduction in each enzyme.

### The abundance of *B. subtilis* mRNAs is primarily dependent on RNases Y and J1

**Figure 2:**
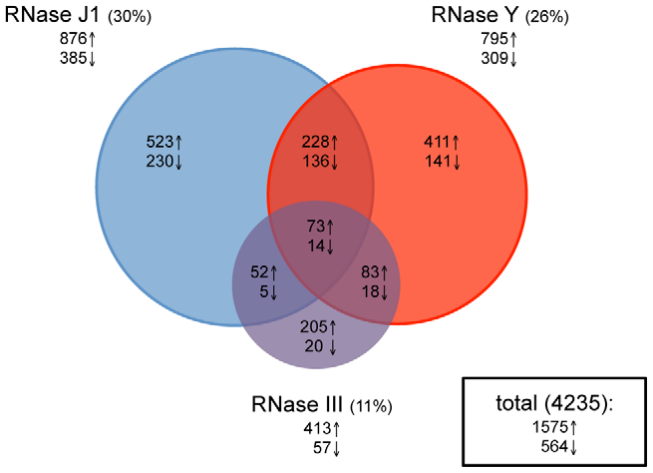
Effects of RNase J1, Y, and III depletion on abundance of *B. subtilis* mRNAs. The Venn diagram shows the number of mRNAs (open reading frames) altered in each of the three mutant strains CCB034 (RNase J1), CCB294 (RNase Y) and CCB288 (RNase III). Upward pointing arrows indicate the number of mRNAs showing increased abundance; downward pointing arrows indicate decreased abundance. The areas of the circles are proportional to the number of mRNAs showing altered abundance in each strain. The total number of mRNAs affected in the experiment is shown in the rectangle to the right of the Venn diagram.

Total RNA was isolated in duplicate from wild-type cells and depletion strains grown in the presence and absence of IPTG, Cy3-labelled by random priming and hybridized to Roche-Nimblegen tiling arrays as described previously [20]. The signal traces are shown in Figure S1 for the whole genome. Data were normalized using a least variable set of genes corresponding to about 10% of the genome and a statistical analysis was performed to establish lists of genes (with a False Discovery Rate (FDR)≤0.1) showing differential expression in the −IPTG condition compared to wild-type (see Materials and Methods). Depletion of RNase III led to a 2-fold increase in the abundance of 413 annotated transcripts and decreased levels of 57 RNA species (Figure 2 and Table S2). This accounts for about 11% of the *B. subtilis* genome and is remarkably similar to the effect of an RNase III deletion on gene expression levels in the *E. coli* genome where 12% of transcripts were affected [23]. In strains depleted for RNase J1, 876 transcripts showed a 2-fold increased abundance, while 385 mRNAs showed reduced levels, accounting for about 30% of the genome. This picture contrasts dramatically to that obtained previously under milder RNase J1 depletion conditions, where only 79 *B. subtilis* transcripts (<2%) were affected [19]. Depletion of RNase Y led to increased abundance of 795 transcripts and decreased expression of 309 mRNAs, accounting for about 26% of *B. subtilis* genes. Although this number of targets is comparable to that seen in a recent study under mild RNase Y depletion conditions [24], more than two-thirds of the predicted targets differ between the two studies (see Discussion). In all, the expression of over half (51%) of *B. subtilis* genes was affected by the depletion of one or other of these three RNases. Remarkably, only 87 transcripts (2%) were common to the depletion of all three RNases, suggesting that *B. subtilis* has not really evolved with a major strategy to deal with the type of stress caused by the loss of an essential RNase.

### Effects common to two RNases

Current models suggest combined enzyme activity plays an important role in the degradation of *B. subtilis* mRNAs. We were therefore very interested in determining which transcripts were common to the depletion of two enzymes, in particular those shared by an endo- and an exonuclease, e.g. RNase Y and J1 or RNase III and J1. About 60 transcripts were common to the RNase III and J1 depletion experiments (52 increased, 5 decreased abundance), while about a hundred mRNAs were shared between the RNase III and Y depletions (83 increased, 18 decreased abundance; Figure 2 and Table S2). A significantly greater number of mRNAs (228 increased, 136 decreased abundance) were common to the RNase J1 and Y depletion experiments. While this is consistent with a combined role for RNase Y endonucleolytic cleavage followed by RNase J1 5′-3′ exonucleolytic degradation for the turnover of about 5% of *B. subtilis* mRNA species, it was less than we anticipated based on current models on RNA turnover in *B. subtilis*. Rather, the increased levels of a relatively large number of mRNAs (12% for RNase J1, 10% for RNase Y) appeared to be dependent only on one or other of the two enzymes. It should be noted, however, that only endonucleolytic cleavages close to the 5′ end of transcripts followed by 5′-3′ degradation by RNase J1 would result in an accumulation of the signal averaged over the whole mRNA length in the RNase J1 mutant. RNAs cleaved closer to the 3′ end would not register as RNase J1-dependent, because only a small fraction of the total length of the mRNA is stabilised, resulting in a lower average response for the whole ORF. RNAs cleaved within the last 20–30 nts would not register either because the resulting fragments could only hybridize to 1 or two probes.

### RNase III depletion has surprisingly little effect on antisense RNAs

**Figure 3:**
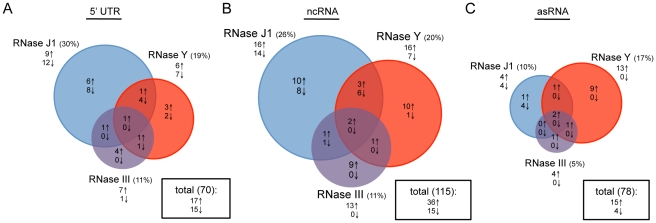
Effects of RNase J1, Y, and III depletion on abundance of *B. subtilis* regulatory RNAs. The Venn diagrams show the number of (A) 5′-UTRs, (B) potential ncRNAs and (C) potential asRNAs altered in each of the three mutant strains CCB034 (RNase J1), CCB294 (RNase Y) and CCB288 (RNase III). Upward pointing arrows indicate the number of RNAs showing increased abundance; downward pointing arrows indicate decreased abundance. The areas of the circles are proportional to the number of RNAs showing altered abundance in each strain. The total number of RNAs affected in the experiment is shown in the rectangle to the right of each Venn diagram.

We asked whether the relative importance of each enzyme was similar for regulatory RNAs, which are generally not translated. About 60 RNAs classified as ‘miscellaneous’ (BSU_misc_RNAs) have been annotated on the *B. subtilis* genome. Many are 5′ untranslated regions (5′-UTR), such as riboswitches or T-boxes, that modulate the expression of the downstream coding sequence through a transcription termination/antitermination mechanism. In addition, recent papers by Rasmussen *et al.* and Irnov *et al.* have identified a number of other candidate regulatory non-coding (nc) RNAs and antisense (as) RNAs [20], [25]. We examined a pool of 263 known or potential 5′ UTRs, ncRNAs and asRNAs for their RNase dependence. The impact of RNase III, J1 and Y depletion was generally fairly similar for 5′-UTRs and ncRNAs compared to translated RNAs (Figure 3 and Table S3). In contrast, for the asRNAs, where we expected to see a much greater role for RNase III due to the potential existence of extensive stretches of double-stranded RNA, RNase III-dependence was significantly reduced and the greatest impact was caused by the depletion of the single-strand specific nuclease RNase Y. Clearly, current models of asRNA turnover need to be re-evaluated (see Discussion).

### Support for the RNase Y + RNase J1–dependent degradation pathway

**Figure 4:**
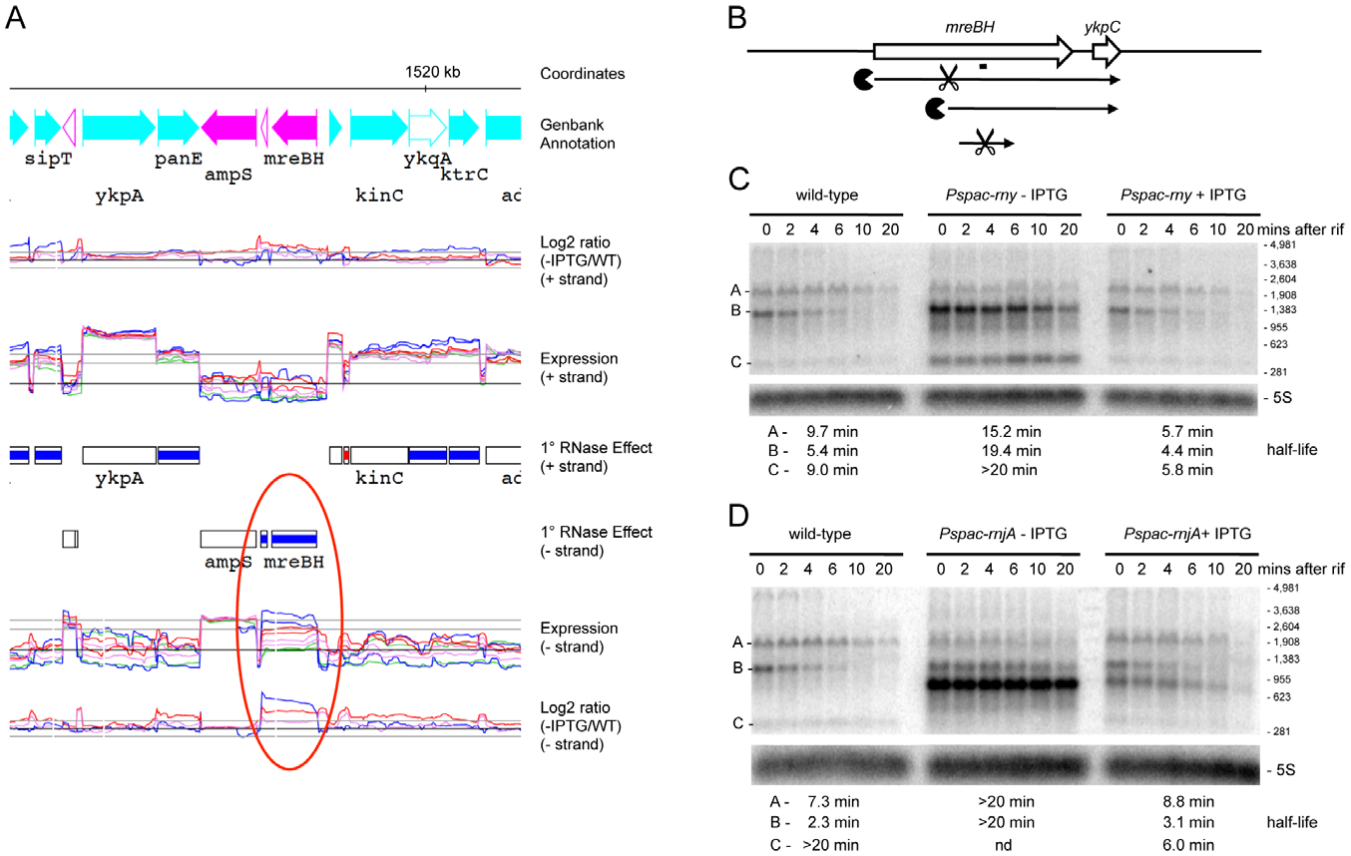
Degradation of the *mreBH ykpC* mRNA depends on both RNase Y and J1. (A) Example of trace of the expression data around the *mreBH ykpC* locus. Lanes are as follows (from top to bottom): (1) Genbank annotation, (2) effect of the depletion of each RNase (log2 ratio −IPTG to wt, calculated on normalized values) on the positive strand, (3) expression signal of wt and RNase depleted strains (normalized log2 values) on the positive strand, (4) summary of the gene-level statistical analysis showing primary (1°) RNase effect (5) expression signal on the negative strand, (6) effect of the depletion of each RNase on the negative strand. The color code for experiments is: wild-type, green; RNase III, violet; RNase J1, blue; RNase Y, red. In the plots of expression signal (lanes 3 and 5), the horizontal black line represents the global median over the whole chromosome and the two horizontal gray lines indicate 5× and 10× this value. In the plots of log2 ratios, the horizontal black line corresponds to base-line (no change) and two horizontal gray lines on either side indicate 2× up and 2× down changes. The summary of the gene-level statistical analysis shows which genes or expression segments were affected by the depletion of at least one of the three RNases: thick green line, gene showing decreased expression in at least one RNase depletion experiment; thick violet, blue or red line, gene showing increased expression in at least one RNase depletion experiment (in this case, the color indicates which depletion was observed to have the greatest effect); thick gray line, gene showing both increased and decreased expression depending on the RNase considered. Color codes for the Genbank annotation are as follows: cyan and magenta, annotated protein coding sequences on the positive and negative strands, respectively (solid symbol when function is known; hollow symbol when function is considered unknown in Genbank); red, ribosomal RNA; dark blue, tRNA; green, Misc_RNA. Traces were plotted using MuGen [43]. Only the signal from unique oligos are shown; gaps are due to non-unique genome sequences. Note: care should be taken when interpreting the log2 ratio signal on the non-coding strand because ratios of values close to background do not have a direct biological interpretation. In particular, these ratios are affected by artifacts such as those caused by reverse transcriptase copying of the cDNA strand, despite the presence of 40 µg/mL actinomycin D in this step. (B) Structure and predicted degradation pathway(s) of the *mreBH ykpC* transcript. ORFs are shown as large white arrows, transcripts as thin black arrows. Scissors indicate cleavage by RNase Y, ‘Pacman’ symbols represent 5′-3′ degradation by RNase J1. A short thick line indicates the position of the probe used. (C) Northern blot of total mRNA isolated at times after addition of rifampicin (rif) from wild-type and RNase Y depleted (*Pspac-rny*−IPTG) and RNase Y induced (*Pspac-rny*+IPTG) cells. The blot was probed with 5′-labeled oligo CCB832 (Table S6) and reprobed with oligo HP246 against 5S rRNA. The half-lives of the different RNA species (A, B, C) from the *mreBH ykpC* are given under the Northern blot. Migration positions of RNA markers are shown to the right of the blot. (D) Northern blot of total mRNA isolated at times after addition of rifampicin (rif) from wild-type and RNase J1 depleted (*Pspac-rnjA*−IPTG) and RNase J1 induced (*Pspac-rnjA*+IPTG) cells. Description as in panel (B); nd is not detected.

We performed Northern blots on a number of RNAs, to get an idea of the level of confidence in the tiling array data and whether the effects seen were at the transcriptional or post-transcriptional level. RNA half-lives were measured in time courses after blocking transcription initiation by addition of rifampicin. The *mreBH ykpC* operon showed a significantly increased abundance of mRNA levels in RNase J1 and RNase Y depleted cells in the tiling array experiment (Figure 4A) and hence we first chose to examine this operon. The *mreBH* gene encodes the actin-like protein involved in *B. subtilis* cell shape, while *ykpC* encodes a protein of unknown function. In wild-type cells, two transcripts were visible: a low abundance but relatively stable ∼1.9 kb transcript and a higher abundance, but unstable ∼1.3 kb transcript (Figure 4B–4D). The origin of the larger transcript is unclear, while the smaller mRNA corresponds to the size of the dicistronic transcript seen by tiling array. When RNase Y was depleted, the 1.3 kb transcript was significantly stabilized, in addition to a smaller degradation intermediate of about 300 nts (Figure 4C). The latter corresponds to a fragment from the middle of the transcript, judging from the position of the probe used. Intriguingly, under RNase J1 depletion conditions, the band pattern was different; a highly stable ∼900 nt degradation intermediate accumulated but the smaller 300 nt species did not (Figure 4D). This pattern is consistent with initial cleavage of the 1.3 kb *mreBH ykpC* by RNase Y about 400 nts from its 5′ end, followed by RNase J1-mediated degradation of the downstream cleavage product. Further cleavage by RNase Y is required for the degradation of the 300 nt fragment. Interestingly, the full-length 1.3 kb transcript was also stabilized in the absence of RNase J1. There is no obvious promoter sequence immediately upstream of the first oligo of the tiling array signal corresponding to this gene. It is possible that the 1.3 kb transcript is processed from the larger low-abundance species, which might account for its sensitivity to RNase J1.

**Figure 5:**
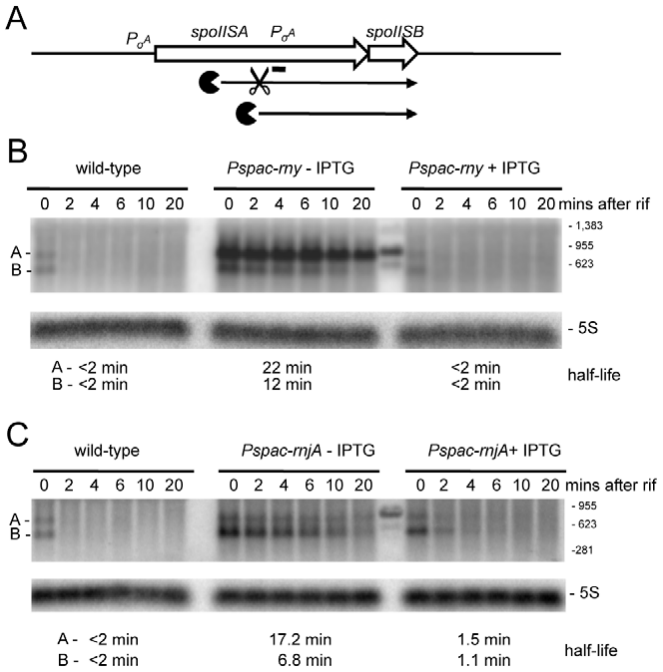
Degradation of the *spoIISAB* mRNA depends on both RNase Y and J1. (A) Structure and predicted degradation pathway(s) of the *spoIISAB* transcript. ORFs are shown as large white arrows, transcripts as thin black arrows. Scissors indicate cleavage by RNase Y, ‘Pacman’ symbols represent 5′-3′ degradation by RNase J1. A short thick line indicates the position of the probe used. Pσ^A^ indicates the approximate promoter position and relevant sigma factor mapped in [26]. (B) Northern blot of total mRNA isolated at times after addition of rifampicin (rif) from wild-type and RNase Y depleted (*Pspac-rny*−IPTG) and RNase Y induced (*Pspac-rny*+IPTG) cells. The blot was probed with 5′-labeled oligo CCB826 (Table S6) and reprobed with oligo HP246 against 5S rRNA. The half-lives of the different RNA species (A, B) from the *spoIISAB* are given under the Northern blot. Migration positions of RNA markers are shown to the right of the blot. Some cross-hybridization to the marker is visible in the lane between the (−) and (+) IPTG samples. (C) Northern blot of total mRNA isolated at times after addition of rifampicin (rif) from wild-type and RNase J1 depleted (*Pspac-rnjA*−IPTG) and RNase J1 induced (*Pspac-rnjA*+IPTG) cells. Description as in panel (B).

A similar pattern was seen in the turnover of a ∼950 nt transcript whose 5′ end maps within the first cistron of the *spoIISAB* operon (Figure S1; 1349 kb). This operon encodes the SpoIISA toxin that can cause lethal damage to the cell envelope during sporulation and its antitoxin SpoIISB [26]. This 5′ end lies well upstream of the predicted start site of the previously mapped *spoIISB* promoter, also within *spoIISA*, whose role is to ensure transcription of the antitoxin. As for *mreBH ykpC*, there is no obvious promoter sequence immediately upstream of the first strongly hybridizing oligo, to account for the expression of the ∼950 nt transcript, suggesting that it may be processed from the larger species that originates from the *spoIISA* promoter. The stability of the ∼950 nt transcript, encoding only the antitoxin, was increased dramatically in an RNase Y mutant, while that of a ∼750 nt degradation intermediate was increased under conditions of RNase J1 depletion (Figure 5). This pattern is also compatible with the model of initial endonucleolytic cleavage by RNase Y, about 200 nts from the 5′ end of this RNA, followed by 5′-3 exonuclease digestion of the downstream fragment by RNase J1. Here again, the full-length transcript was stabilized in the RNase J1 mutant, suggesting that some portion of the turnover of this mRNA proceeds from its 5′ end.

We also studied the turnover of a regulatory RNA, the 5′-UTR of the *proI* gene (Figure S1; 2473 kb), recently shown to be involved in T-box mediated regulation of *proI* gene expression [27]. The turnover pattern of this RNA is quite complex, with one major and one minor species visible in wild-type cells (Figure S2, species A and C). In RNase Y depleted cells, the full-length leader (A) is stabilized and two new species (B and D) accumulate. In RNase J1-depleted cells, three species are stabilized (A, C and E). Species B and D are slightly larger than C and E, respectively. This pattern is consistent with RNase Y cleavage of the *proI* leader close to the 5′ ends of species B and D, followed by degradation of the resulting C and E fragments by RNase J1.

### Transcripts affected by depletion of RNases III and J1

**Figure 6:**
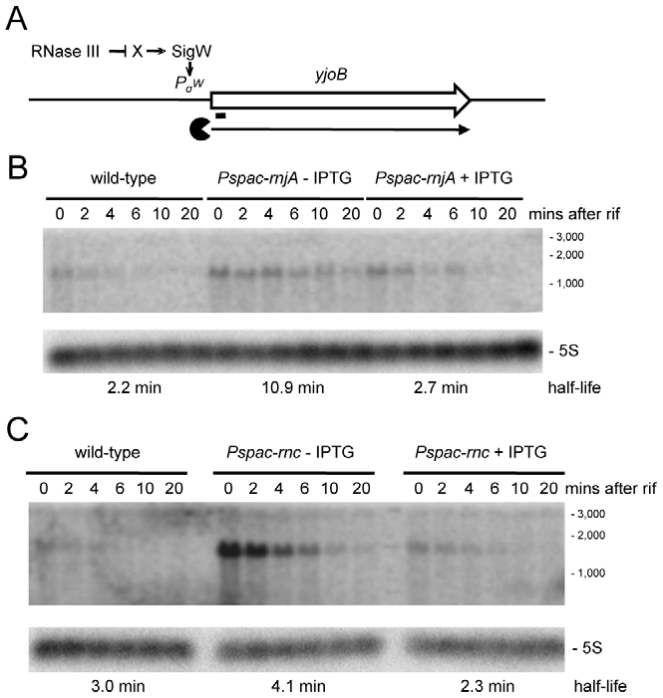
Degradation of the *yjoB* mRNA depends on RNase J1 while its transcription is dependent on RNase III. (A) Structure and predicted degradation pathway of the *yjoB* transcript. The ORF is shown as a large white arrow, transcript as a thin black arrow. The ‘Pacman’ symbol represent 5′-3′ degradation by RNase J1. A short thick line indicates the position of the probe used. Pσ^W^ indicates the approximate promoter position and relevant sigma factor mapped in [31], [32]. The schematic also depicts RNase III initiated degradation of a transcript encoding an unknown factor X early in the SigW cascade. (B) Northern blot of total mRNA isolated at times after addition of rifampicin (rif) from wild-type and RNase J1 depleted (*Pspac-rnjA*−IPTG) and RNase J1 induced (*Pspac-rnjA*+IPTG) cells. The blot was probed with 5′-labeled oligo CCB807 (Table S6) and reprobed with oligo HP246 against 5S rRNA. The half-life of the *yjoB* transcript is given under the Northern blot. Migration positions of RNA markers are shown to the right of the blot. (C) Northern blot of total mRNA isolated at times after addition of rifampicin (rif) from wild-type and RNase III depleted (*Pspac-rnc*−IPTG) and RNase III induced (*Pspac-rnc*+IPTG) cells. Description as in panel (B).

**Figure 7:**
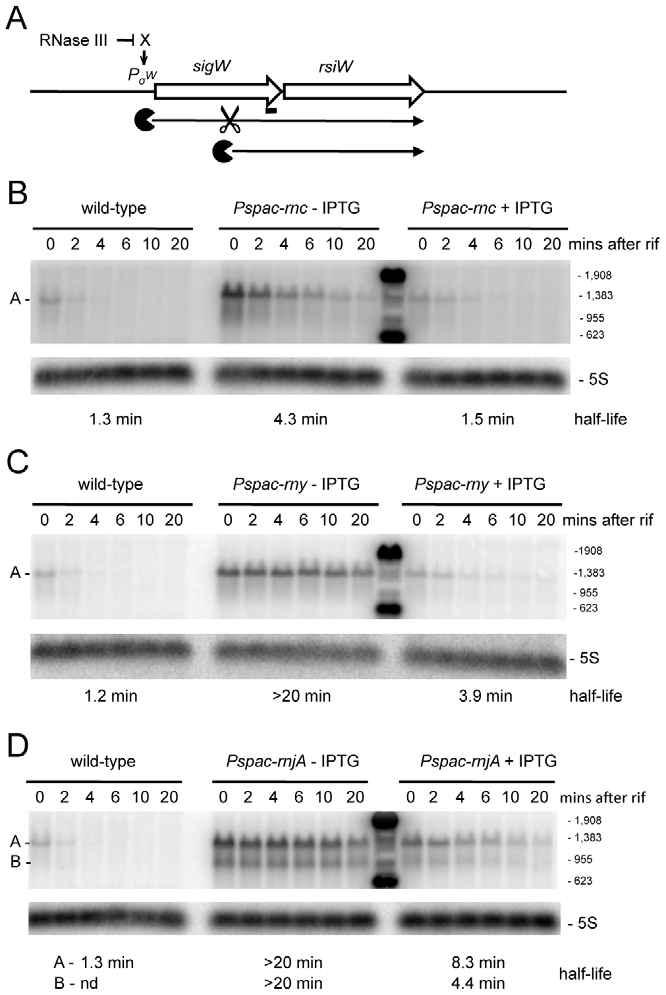
Degradation of the *sigW-rsiW* mRNA depends on RNase Y and RNase J1, while its transcription is dependent on RNase III. (A) Structure and predicted degradation pathway of the *sigW-rsiW* transcript. ORFs are shown as large white arrows, transcripts as thin black arrows. Scissors indicate cleavage by RNase Y, ‘Pacman’ symbols represent 5′-3′ degradation by RNase J1. A short thick line indicates the position of the probe used. Pσ^W^ indicates the approximate promoter position and relevant sigma factor mapped in [31], [32]. The schematic also depicts RNase III initiated degradation of a transcript encoding an unknown factor X early in the SigW cascade. (B) Northern blot of total mRNA isolated at times after addition of rifampicin (rif) from wild-type and RNase III depleted (*Pspac-rnc*−IPTG) and RNase III induced (*Pspac-rnc*+IPTG) cells. The blot was probed with 5′-labeled oligo CCB900 (Table S6) and reprobed with oligo HP246 against 5S rRNA. The half-life of the *sigW-rsiW* transcript is given under the Northern blot. Migration positions of RNA markers are shown to the right of the blot. Cross-hybridization to the marker is visible in the lane between the (−) and (+) IPTG samples. (C) Northern blot of total mRNA isolated at times after addition of rifampicin (rif) from wild-type and RNase Y depleted (*Pspac-rny*−IPTG) and RNase Y induced (*Pspac-rny*+IPTG) cells. Description as in panel (B). (D) Northern blot of total mRNA isolated at times after addition of rifampicin (rif) from wild-type and RNase J1 depleted (*Pspac-rnjA*−IPTG) and RNase J1 induced (*Pspac-rnjA*+IPTG) cells. Description as in panel (B).

We examined a number of transcripts that accumulated in strains depleted for RNase III and RNase J1 with the expectation that, in these cases, the initial endonucleolytic cleavage event would be catalyzed by RNase III. The *yjoB* gene (Figure S1; 1315 kb) encoding a member of the AAA family of putative molecular chaperones [28], the *yknWXYZ* operon (Figure S1; 1504 kb) encoding an ABC-type efflux pump [29] and the *fosB* gene (Figure S1; 1916 kb) encoding a fosfomycin resistance protein [30], were three such examples. While the effect of RNase J1 depletion on mRNA half-life was confirmed for each of these full-length RNAs (Figure 6 and Figures S3, S4), to our surprise, the effects of the RNase III-depletion were primarily due to transcriptional rather than post-transcriptional effects, i.e. the transcript accumulated, but its half-life was not sufficiently altered to explain the increase in abundance. A similar transcriptional effect of the RNase III-depletion was seen for the *yfhLM* operon (Figure S1; 930 kb), encoding the SdpC peptide immunity factor [29] and a predicted hydrolase, and for the *yrkA* transcript (Figure S1; 2720 kb), encoding a predicted membrane protein of unknown function (Figures S5, S6). Four of these five genes, initially chosen at random to study strong RNase III dependent effects, are members of the extracytoplasmic sigma factor SigW regulon, containing some 60–70 genes [31], [32]. We therefore studied the effect of RNase III-depletion on the *sigW*-*rsiW* transcript itself (Figure S1; 195 kb). While we measured an increase in *sigW*-*rsiW* half-life in the absence of RNase III compared to wild-type cells (1.3 vs. 4.3 minutes), it is clear that the greater effect was once again transcriptional (Figure 7). In fact, turnover of the *sigW*-*rsiW* mRNA is primarily dependent on the RNase Y/J1 pathway, while RNase III presumably affects the abundance of a yet unknown RNA upstream of SigW in the cascade. Thus, while RNase III has an impact on about 11% of the transcriptome, a significant subset of these changes in RNA levels are likely to be due to indirect transcriptional effects.

### Identification of new potential regulatory RNAs

**Figure 8:**
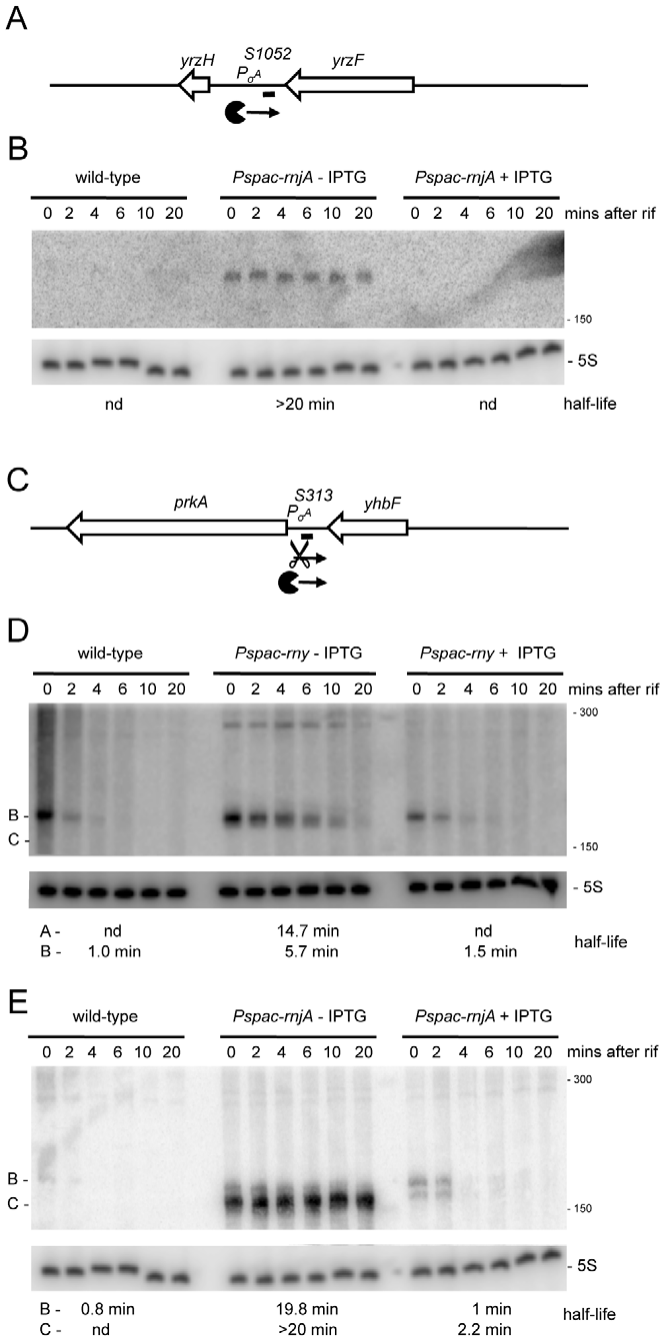
Detection of new potential regulatory RNAs in RNase Y and J1 mutants. (A) Structure and predicted degradation pathways of the S1052 RNA. ORFs are shown as large white arrows, transcripts as thin black arrows. ‘Pacman’ symbol represents 5′-3′ degradation by RNase J1. A short thick line indicates the position of the probe used. Pσ^A^ indicates the putative promoter and sigma factor for the regulatory RNA predicted by the DBTBS website (http://dbtbs.hgc.jp/) and in agreement with the size detected by Northern blot. (B) Northern blot of total mRNA isolated at times after addition of rifampicin (rif) from wild-type and RNase J1 depleted (*Pspac-rnjA*−IPTG) and RNase J1 induced (*Pspac-rnjA*+IPTG) cells. The blot was probed with 5′-labeled oligo CCB852 (Table S6) and reprobed with oligo HP246 against 5S rRNA. The half-life of the S1052 transcript is given under the Northern blot (nd is not detected). Migration positions of RNA markers are shown to the right of the blot. (C) Structure and predicted degradation pathway of the S313 RNA. Features as in panel (A); scissors indicate cleavage by RNase Y. (D) Northern blot of total mRNA isolated at times after addition of rifampicin (rif) from wild-type and RNase Y depleted (*Pspac-rny*−IPTG) and RNase Y induced (*Pspac-rny*+IPTG) cells. The half-lives of the different RNA species (A, B) containing S313 are given under the Northern blot (nd is not detected). Migration positions of RNA markers are shown to the right of the blot. (E) Northern blot of total mRNA isolated at times after addition of rifampicin (rif) from wild-type and RNase J1 depleted (*Pspac-rnjA*−IPTG) and RNase J1 induced (*Pspac-rnjA*+IPTG) cells. The blot was probed with 5′-labeled oligo CCB854 (Table S6) and reprobed with oligo HP246 against 5S rRNA. Description as in panel (D).

We anticipated that the tiling array analysis of RNase mutants would permit us to identify a number of new regulatory RNAs, both *trans*-acting small RNAs (sRNA) and *cis*-acting antisense RNAs (asRNA). Although mid-log phase in rich medium is not an optimal condition for the expression of most regulatory RNAs, we suspected that, in the absence of key RNases, many such RNAs would be sufficiently stabilized to permit their detection. The tiling array experiment indeed permitted the detection about 20 potential *trans*-acting regulatory RNAs (Table S4, “indep” and “indepMT”) not seen in the Rasmussen and Irnov studies [20], [25]. Most of these RNAs were also detected in a separate tiling array study of over a hundred different growth conditions and have been given a ‘segment’ number (S) according to their order of appearance on the *B. subtilis* genome (Nicolas *et al.*, unpublished data). The present study provides an independent validation of their existence and shows the power of using RNase mutants for their detection. Two examples are shown in Figure 8 and others are listed in Table S4. The negative strand of the intergenic region between *yrzF* and *yrzH* (Figure S1; 2841 kb) expresses a potential ∼190 nt sRNA called S1052 that is stabilized under conditions of RNase J1 depletion. Likewise, the negative strand of the *yhbF-prkA* intergenic region (Figure S1; 973 kb) expresses a potential ∼170 nt sRNA named S313 that is stabilized in an RNase Y mutant in the absence of IPTG. In the absence of RNase J1, a slightly shorter species (∼150 nt) is stabilized. This pattern is consistent with RNase Y cleavage about 20 nts from the 5′ end of S313 followed by RNase J1 digestion of the downstream fragment.

**Figure 9:**
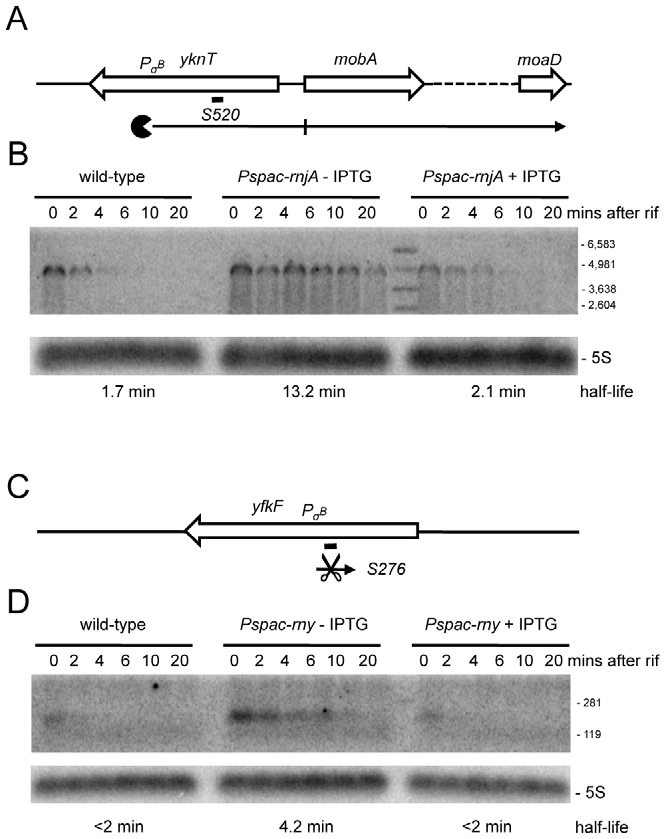
Detection of new potential asRNAs in RNase Y and J1 mutants. (A) Structure and predicted degradation pathways of the S520 RNA antisense to *yknT*. ORFs are shown as large white arrows, transcripts as thin black arrows. Scissors indicate cleavage by RNase Y, ‘Pacman’ symbols represent 5′-3′ degradation by RNase J1. A short thick line indicates the position of the probe used. Pσ^B^ indicates the putative promoter and sigma factor for the regulatory RNA predicted by the DBTBS website (http://dbtbs.hgc.jp/) and in agreement with the size detected by Northern blot. (B) Northern blot of total mRNA isolated at times after addition of rifampicin (rif) from wild-type and RNase J1 depleted (*Pspac-rnjA*−IPTG) and RNase J1 induced (*Pspac-rnjA*+IPTG) cells. The blot was probed with 5′-labeled oligo CCB857 (Table S6) and reprobed with oligo HP246 against 5S rRNA. The half-life of the S520 transcript is given under the Northern blot. Migration positions of RNA markers are shown to the right of the blot. (C) Structure and predicted degradation pathway of the S276 RNA antisense to *yfkF*. Features as in panel (A). (D) Northern blot of total mRNA isolated at times after addition of rifampicin (rif) from wild-type and RNase Y depleted (*Pspac-rny*−IPTG) and RNase Y induced (*Pspac-rny*+IPTG) cells. The blot was probed with 5′-labeled oligo CCB859 (Table S6) and reprobed with oligo HP246 against 5S rRNA. Description as in panel (B).

About 100 new antisense RNAs were also detected in the tiling array experiment of the RNase-depletion strains compared to the Rasmussen and Irnov studies [20], [25]. These asRNAs and their RNase-dependence are shown in Table S4 and Figure S7. All but two of these asRNA were also seen in the large-scale study of different growth conditions in *B. subtilis* (Nicolas *et al.*, unpublished data). A very interesting example is the 4.9 kb molybdopterin biosynthetic operon mRNA (*mobA* to *moaD*). This transcript has a very long 5′-UTR (∼800 nt) that is complementary to about two-thirds of the *yknT* mRNA (1495 kb) expressed from the opposite strand. The tiling array signal corresponding to the 5′-UTR (S520) is significantly increased in an RNase J1 mutant compared to wild-type cells and actually results from a stabilization of the full-length mRNA (Figure 9A, 9B). A second example is an asRNA called S276 that is complementary to an internal portion of the *yfkF* mRNA (Figure S1; 865 kb), encoding a predicted efflux transporter. A ∼180 nt asRNA is stabilized under conditions of RNase Y depletion (Figure 9C, 9D). It is somewhat smaller than that anticipated from the tiling array experiment (650 nts), suggesting that it may correspond to more than one species. Interestingly, both S520 and S276 have sequences matching a Sigma-B dependent promoter just upstream and may therefore be controlled by conditions of stress.

By far the most abundant class of new RNA species affected in the RNase-depletion strains were the 5′-UTRs (164 increased, 85 decreased abundance; shown in Table S4 and Figure S7). Most of these (60%) are likely to be too short (1–2 oligos) to have a regulatory function, but others are significantly longer and potentially control the expression of the downstream coding sequence. Curiously, the vast majority of the longer 5′-UTRs (≥300 nts) are also antisense RNAs, creating situations similar to that which we saw with S520 and *yknT*. We examined the turnover of S935, corresponding to the 5′-UTR of the *yqzDC* operon (Figure S1; 2577 kb), which encodes two proteins of unknown function. S935 overlaps the YqgL open reading frame (also of unknown function) on the opposite strand by about 30 nts. Although this 5-UTR showed increased abundance in all three depletion strains in the tiling array, a significantly greater accumulation was seen in the RNase Y mutant. Indeed, in Northern blots of cells depleted for RNase Y, a significant stabilization of S935 was seen as a 5′ extension of the *yqzDC* transcript, suggesting that cleavage by RNase Y removes the 5′-UTR (Figure S8).

## Discussion

The data described here provide evidence, at an unprecedented level of detail, that is compatible with both of the current models of RNA turnover in *B. subtilis*. Nonetheless, they also leave open the possibility that other models could be considered and/or that other enzymes may be involved. In the first model, RNAs are first subjected to endonucleolytic cleavage followed by exonucleolytic decay of the resulting fragments by exonucleases. This is similar to the *E. coli* model of mRNA degradation, but with different players. Our data are consistent with RNase Y playing the key endonucleolytic role, with the expression of over a quarter of the genome affected by RNase Y depletion. (It should be noted that only about half of *B. subtilis* genes are transcriptionally active in rich medium [20].) In this model, the downstream products of endonucleolytic cleavage are degraded by the 5′-3′ exoribonuclease activity of RNase J1, while the upstream cleavage products are degraded by 3′-5′ exonucleases. A similar number of transcripts were affected by the RNase J1 depletion, with about 30% overlap between the RNase J1 and RNase Y data sets. As pointed out earlier, this may be an underestimate of the number of substrates subjected to the combined action of RNase Y and J1, because only in those cases where RNase Y cleavage occurs near the 5′ end of the transcript would the average signal over the whole transcript be seen to increase in an RNase J1 mutant.

In the second model, RNase J1 attacks the 5′ end of primary transcripts following deprotection of the RNA by pyrophosphate removal [16]. This is reminiscent of the eukaryotic model of RNA decapping and degradation by the 5-3′ exoribonuclease Xrn1. There were 575 transcripts of increased abundance in the RNase J1 depletion strain that were not shared with RNase Y. We would anticipate that there should be substrates of the deprotection-dependent pathway of RNA turnover within this pool of mRNAs. Indeed, the previously characterized BsRppH-dependent *yhxA-glpP* transcript [16] is found within this group of RNAs. We also considered the possibility that some of the RNase J1-dependent effects might be indirect, if RNase J1-depletion were to lead to a decrease in RNase Y expression, for example. However, RNase Y transcript levels remain unchanged in RNase J1 depleted strains and *vice versa* (Table S2). Indeed, there seems to be little evidence for cross-regulation between any of the three RNases at the transcript level. This observation does not exclude, however, the possibility of effects at the level of translation or enzyme activity, through protein-protein interactions, for example. Although it has been suggested that RNase Y interacts with RNase J1 in a degradosome-like complex in *B. subtilis* [7], which could also explain the accumulation of full-length transcripts in RNase J1 mutant, we were unable to detect this interaction in independent experiments [15].

Although the abundance of 25–30% of *B. subtilis* transcripts was altered in each of the strains depleted for RNase J1 and Y, this number is likely to be an underestimate of the full involvement of these two enzymes in mRNA turnover in this organism for a number of reasons. While we have achieved a >30-fold depletion of each enzyme, it is clear that we have not attained a level of depletion equivalent to a gene knock-out. Very high affinity substrates of these enzymes, i.e. those for which only a very low concentration of RNase is sufficient for binding and activity, have therefore probably escaped detection. Secondly, about 100 transcripts are already close to saturation levels in wild-type cells; further stabilization of these mRNAs in RNase-depletion strains would not give an increased signal. Lastly, it is also possible that, because of the nature of the severe depletion experiment, some stabilization effects are obscured by decreased levels of transcription due to the slow down in growth rate. Such transcripts would escape detection in our analysis. This is almost certainly also the case for the *rpsO* mRNA, for example, which encodes ribosomal protein S20 and is known to be stabilized under RNase Y depletion conditions [9]. Components of the translational apparatus are typically down-regulated at decreased growth rate, likely explaining why *rpsO* levels were not increased in the tiling array analysis of cells severely depleted for RNase Y. A similar phenomenon may explain the lack of accumulation of the *yitJ* leader region (BSU_MiscRNA_12; Table S3) and *trp* leader regions (S857; Table S4), two other RNAs previously shown to accumulate under mild RNase Y depletion conditions [8], [33].

Decreased abundance of a particular transcript under RNase depletion conditions is likely explained through indirect effects, stabilization of an RNA encoding a negative regulator, for example. An example of this type of phenomenon was seen with the *ybeC* transcript (Figure S1; 232 kb), encoding a potential amino acid-proton symporter, whose transcription levels were significantly decreased in an RNase J1 mutant (Figure S9). Somewhat unexpectedly, the *ybeC* mRNA was also significantly stabilized under RNase J1 depletion conditions, although not enough to compensate for the decreased transcription of the gene. Thus, it is possible to find direct RNase targets even among transcripts showing decreased abundance.

All candidate RNAs showing increased abundance in RNase Y and J1 depleted strains by tiling array that we tested showed increased half-lives when analyzed by Northern blot. On the other hand, RNAs showing increased abundance in the RNase III mutant showed primarily increased transcriptional levels by Northern analysis. This suggests that the number of RNAs subjected to direct endonuclease activity by RNase III is significantly less than the 470 transcripts whose abundance was altered in the RNase III mutant. Four out of five RNAs examined, including the *sigW-rsiW* RNA itself, belonged to the *sigW* regulon that responds to membrane stress, suggesting that RNase III affects the stability of an RNA encoding an effector upstream of SigW in the cascade. The transcripts of two known effectors of *sigW* expression, AbrB and Spo0A, are not significantly affected by the RNase III depletion by tiling array (Table S2). The target of RNase III in this pathway therefore remains to be identified.

We were surprised that RNase III did not have a greater effect on potential antisense RNAs as we expected that extended duplexes of sense and antisense RNAs would be ideal substrates for RNase III. Rather, the greatest proportion (17%) of antisense RNAs was affected by depletion of the single-strand specific nuclease RNase Y (Figure 3C). In the regulation of plasmid R1 replication, the productive complex between the antisense RNA *copA* and its target *copT* is not an extended duplex, but rather a four-way helical junction [34]. A similar conformation was seen in the duplex between the antisense RNA *inc* and its target *repZ* of plasmid Col1b-P9 [35]. The results of the tiling array experiment would suggest that complexes analogous to *copA/copT* or *inc/repZ*, in which some single-stranded regions exist that are recognized by RNase Y, may be more the norm than extended duplexes between sense/antisense hybrids in *B. subtilis*.

A recent study also provided evidence in support of a role for RNase Y in the turnover of about 20% of *B. subtilis* mRNAs [24]. In a transcriptome analysis performed under conditions of mild RNase Y depletion in a polar strain, the abundance of about 900 transcripts (550 up; 350 down) was deemed to be altered using the arbitrary cut-off value of ≥1.5-fold. Only 263 candidate genes (219 increased and 44 decreased abundance) were in common with the RNase Y-depletion study presented here (Table S5). By decreasing our cut-off value to ≥1.5-fold to match that of the Lehnik-Habrink study, only 75 more candidates (63 up, 12 down) were added to the shared pool (Table S5). The mild RNase Y depletion coupled with a cut-off value that may be close to background probably accounts for the relatively small overlap in specific targets between the two studies, despite their similar overall numbers of candidate targets. In addition to the greater RNase depletion levels and detection of higher affinity substrates, the high resolution tiling array analysis described here has the further advantage of revealing the behavior of both characterized and previously unknown potential regulatory RNAs (5′UTRs, ncRNAs and asRNAs) in response to RNase insufficiency.

We performed a statistical analysis of the functional categories (metabolism, regulation etc.) of the different mRNAs affected in the three RNase mutants (Table S7). About 110 functional categories and sub-categories were examined in total. A number of categories were over- and under-represented among RNAs showing decreased abundance in the tiling arrays, but it is not clear that these are direct effects and therefore what their significance might be. Eleven functional sub-categories were over-represented and four under-represented among RNAs showing increased stability and/or abundance. A summary of the significant effects of the three RNases on is shown in Table 1. The large number of genes from the SigW regulon, whose expression was increased under conditions of RNase III-depletion (see above), accounts in part for the over-representation of the ‘cell envelope stress proteins’ sub-category. Interestingly, this sub-category was also over-represented in the RNase Y mutant. Indeed, RNase Y seems to have an important general role in the synthesis of components of the cell envelope and cell-wall, which is intriguing given its sub-cellular localisation in the membrane. Another intriguing role for RNase Y is in the expression of genes from the prophage PBSX; practically the whole of PBSX (92% of genes) shows increased mRNA abundance under RNase Y-depletion conditions. The significance of the effects of the different RNases on these functional categories of genes remains to be determined.

**Table 1:** Functional categories of mRNAs stabilised in RNase III, J1, and Y mutants.

Functional Category	Number genes	RNase III % up	RNase J1 % up	RNase Y % up
Cellular processes * cell envelope and cell division * cell wall synthesis	74			51.4
Cellular processes * homeostasis * metal ion homeostasis (K, Na, Ca, Mg)	26	42.3		
Cellular processes * transporters * transporters/other	195		33.3	
Groups of genes * essential genes * NA	258	2.7		
Groups of genes * membrane proteins * NA	1073		28.5	
Information processing * protein synthesis, modification and degradation * translation	177			6.8
Lifestyles * coping with stress * biosynthesis of antibacterial compounds	52			0.0
Lifestyles * coping with stress * cell envelope stress proteins (controlled by SigM, W, X, Y)	129	28.7		41.9
Lifestyles * coping with stress * coping with hyper-osmotic stress	22	50.0		
Lifestyles * coping with stress * resistance against toxins/antibiotics	54		50.0	
Lifestyles * exponential and early post-exponential lifestyles * biofilm formation	45		46.7	
Metabolism * additional metabolic pathways * biosynthesis of cell wall components	56			55.4
Metabolism * amino acid/nitrogen metabolism * biosynthesis/acquisition of amino acids	145	26.2		
Metabolism * amino acid/nitrogen metabolism * use of nitrogen sources other than amino acids	43	39.5		
Prophages and mobile genetic elements * prophages * PBSX prophage	38			92.1
Prophages and mobile genetic elements * prophages * SP-beta prophage	186		6.5	6.5
Reference values for whole genome	4234	9.8	20.7	18.8

Different levels of functional categories are separated by asterisks.

This study has given us an exceptionally detailed view of RNA turnover in *B. subtilis* and revealed many new RNAs, previously difficult to detect because of their low expression levels. Using the trace shown in Figure S1, it is now possible to zoom in on any particular gene of interest and, not only determine which of the three essential RNases, RNase J1, RNase Y or RNase III, impact its abundance, but also the 5′ and 3′ extremities of the relevant species to within a few nucleotides.

## Materials and Methods

### Construction of bacterial strains

The *B. subtilis* strains used in this study were derivatives of W168.

Strain CCB034 has been described previously [21].

Strain CCB012 was constructed by transforming strain YMDAp (a kind gift from N. Ogasawara) with pMAP65. The description of its construction can be found at http://bacillus.genome.ad.jp/.

Strain CCB294 was constructed as follows: First, the *Pspac-ymdA* construct was amplified from CCB012 using oligos CC802 and CC803 (Table S6) and digested with EcoRI and SphI. Then, an SphI/BamHI fragment containing the *lacI* gene was purified from plasmid pDG148 [36]. These two fragments were ligated together with integration vector pDG1662 [37] digested with EcoRI and BamHI to create plasmid pDG1662-*Pspac-ymdA*-*lacI*, which was then integrated at the *amyE* locus of *B. subtilis* W168 to create strain CCB292. The native *rny* coding sequence was replaced in CCB292 with that of a spectinomycin resistance gene as follows: Three PCR fragments, containing the *spc* coding sequence (oligos CC798/801) and sequences immediately upstream (oligos CC768/799) and downstream (oligos CC800/770) of the *rny* coding sequence were assembled by overlapping PCR and then re-amplified by nested PCR using oligos CC774/775. The resulting PCR fragment was used to transform CCB292 in the presence of IPTG to create strain CCB293. Inactivation of the native *rny* gene was confirmed by PCR using oligos CC770/797. This strain was transformed with pMAP65 to obtain strain CCB294.

Strain CCB288 was constructed by transferring the *amyE::Pspac-rnc* and *acp::spc::smc* (the native *rnc* CDS replaced by the *spc* CDS) constructs from BG324 [38] to *B. subtilis* W168 in two steps, followed by transformation with pMAP65.

### Tiling array analysis

Overnight cultures of depletion strains grown in the presence of 1 mM IPTG were washed twice in 2xYT medium and inoculated in fresh medium at an OD_600_ of 0.0025–0.05, with or without IPTG. Cultures lacking IPTG typically plateau at OD_600_ of around 0.6 under these conditions. 20 mL of OD_600_∼0.6 cultures were centrifuged and RNA was prepared by the glass beads method [39], with an additional RQ DNase (Promega) step (0.01 units/µL, 37°C for 30 mins) after the second phenol extraction. RNA concentrations were measured and sent to Roche/Nimblegen for labeling and analysis on second-generation (T2) tiling arrays according to the BaSysBio protocol described in Rasmussen *et al.* [20].

### Data analysis

Transcriptional profiles along the chromosome were estimated for each of the hybridizations using a model of signal shift and drift that accounts for differential probe affinity ([40]; Figure S1). Aggregated gene expression values (log2-scale) were then calculated as the median of the estimated probe-level values, using probes lying entirely within the coding sequence and with a unique match on the genome sequence. The large number of transcripts affected in addition to the strong imbalance between up- and down- effects of RNase depletion precluded the use of the most common normalization methods (such as quantile-normalization) to reduce technical variations between experiments. In keeping with the reasoning of [41], normalization was therefore performed by selecting a sub-set of annotated genes that showed low expression variance across the four main conditions of interest (the wild-type and the three RNase depleted strains). To avoid bias in the expression values of this gene set, we first grouped the genes into 10 equal expression intervals according to their average values and then selected the 10% least-variant genes within each group. We used the data for this least-variant set to fit the non-linear transformation (using the ‘loess’ function provided in R (http://www.R-project.org) with span parameter set to 0.5) that then served for the normalization of the whole gene set for each of the hybridizations. To identify differentially expressed genes, we considered a single linear model for each gene, where expression in condition i and replicate j writes x_ij_ = wt+ΔRNase_i_+ε_ij_, with ΔRNase_i_ modeling the effect of the depletion of each particular RNase with respect to the wild-type and ε_ij_ accounting for the experimental variance. Then, we tested whether the term ΔRNase_i_ was non-null (using the ‘lm’ function in R) and we transformed the p-values associated with these tests into q-values that served to set the False Discovery Rate (FDR) [42] when establishing lists of differentially expressed genes.

We included the following expressed regions in the analysis in addition to annotated genes: (i) transcribed segments (labeled “S” in Table S4), mapped in a thorough study of the wild-type strain in over 100 different growth conditions (Nicolas *et al.*, unpublished data), which reached an expression threshold 5× above the chromosome median (considered as background) in at least one hybridization of this study before normalization (ii) new segments (labeled “T” in Table S4), where expression reached 10× above background and for which aggregated expression values suggested differential expression between our four main experiments (ANOVA F-test with a p-value cut-off set to 0.05).

### Western blot analysis

Ten micrograms of sonicated RNase-depleted cell extracts (OD_600_∼0.6) were run on 10% SDS-PAGE gels with 50 ng of purified RNase J1, III or Y as controls. Gels were transferred to hybond C membrane and incubated overnight with a 1∶10000 dilution of antibodies against RNase J1 and III or 1∶1000 dilution for antibodies against RNase Y. Western blot were revealed by incubating with protein A-I^125^ (1∶1000 dilution) and quantified by PhosphorImager.

### Northern blot analysis

RNAs were isolated from OD_600_∼0.6 cultures containing or lacking 1 mM IPTG at different times after addition of rifampicin (150 µg/mL). To stop cell growth and rapidly chill cells, 20 mL culture was pipetted into 10 mL of 10 mM azide frozen in a slanted position. The mixture was then vortexed until azide melted and centrifuged immediately. RNAs were isolated as above. Typically 5 µg RNA was run on 1% agarose or 5% acrylamide gels and transferred to hybond-N membranes (GE-Healthcare). Hybridization was performed using 5′-labeled oligonucleotides using Ultra-Hyb (Ambion) or Roti-Hybri-Quick (Carl Roth) hybridization buffer at 42°C for a minimum of 4 hours. Membranes were washed twice in 2× SSC 0.1% SDS (once rapidly at room temperature (RT) and once for 10 min at 42°C) and then 5 times for 2 mins in 0.2× SSC 0.1% SDS at RT. Oligonucleotides used are shown in Table S6.

## Supporting Information

Figure S1 Trace of the expression data along the whole genome. Lanes are as follows (from top to bottom): (1) Genbank annotation, (2) effect of the depletion of each RNase (log2 ratio −IPTG to wt, calculated on normalized values) on the positive strand, (3) expression signal of wt and RNase depeleted strains (normalized log2 values) on the positive strand, (4) summary of the gene-level statistical analysis, (5) expression signal on the negative strand, (6) effect of the depletion of each RNase on the negative strand. The color code for experiments is: wild-type, green; RNase III, violet; RNase J1, blue; RNase Y, red. In the plots of expression signal (lanes 3 and 5), the horizontal black line represents the global median over the whole chromosome and the two horizontal gray lines indicate 5× and 10× this value. In the plots of log2 ratios, the horizontal black line corresponds to base-line (no change) and two horizontal gray lines on either side indicate 2× up and 2× down changes. The summary of the gene-level statistical analysis shows which genes or expression segments were affected by the depletion of at least one of the three RNases: thick green line, gene showing decreased expression in at least one RNase depletion experiment; thick violet, blue or red line, gene showing increased expression in at least one RNase depletion experiment (in this case, the color indicates which depletion was observed to have the greatest effect); thick gray line, gene showing both increased and decreased expression depending on the RNase considered. Color codes for the Genbank annotation are as follows: cyan and magenta, annotated protein coding sequences on the positive and negative strands, respectively (solid symbol when function is known; hollow symbol when function is considered unknown in Genbank); red, ribosomal RNA; dark blue, tRNA; green, Misc_RNA. Traces were plotted using MuGen [43]. Only the signal from unique oligos are shown; gaps are due to non-unique genome sequences. Note: care should be taken when interpreting the log2 ratio signal on the non-coding strand because ratios of values close to background do not have a direct biological interpretation. In particular, these ratios are affected by artifacts such as those caused by reverse transcriptase copying of the cDNA strand, despite the presence of 40 µg/mL actinomycin D in this step.
(PDF)

Figure S2 Degradation of the *proI* leader depends on RNases Y and J1. (A) Structure and predicted degradation pathway of the *proI* leader. ORFs are shown as large white arrows, transcript as a thin black arrow. Scissors indicate cleavage by RNase Y; ‘Pacman’ symbols represent 5′-3′ degradation by RNase J1. A short thick line indicates the position of the probe used. Pσ^A^ indicates the approximate promoter position and relevant sigma factor mapped in [27]. (B) Northern blot of total mRNA isolated at times after addition of rifampicin (rif) from wild-type and RNase Y depleted (*Pspac-rny*−IPTG) and RNase Y induced (*Pspac-rny*+IPTG) cells. The blot was probed with 5′-labeled oligo CCB853 (Table S7) and reprobed with oligo HP246 against 5S rRNA. The half-lives of the different *proI* leader species (A, B... etc) are given under the Northern blot. Migration positions of RNA markers are shown to the right of the blot. (C) Northern blot of total mRNA isolated at times after addition of rifampicin (rif) from wild-type and RNase J1 depleted (*Pspac-rnjA*−IPTG) and RNase J1 induced (*Pspac-rnjA*+IPTG) cells. Description as in panel (B).
(TIF)

Figure S3 Degradation of the *fosB* mRNA depends on RNase J1 while its transcription is dependent on RNase III. (A) Structure and predicted degradation pathway of the *fosB* transcript. ORFs are shown as large white arrows, transcript as a thin black arrow. The ‘Pacman’ symbol represents 5′-3′ degradation by RNase J1. A short thick line indicates the position of the probe used. Pσ^W^ indicates the approximate promoter position and relevant sigma factor mapped in [31]. The schematic also depicts RNase III initiated degradation of a transcript encoding an unknown factor X early in the SigW cascade. (B) Northern blot of total mRNA isolated at times after addition of rifampicin (rif) from wild-type and RNase J1 depleted (*Pspac-rnjA*−IPTG) and RNase J1 induced (*Pspac-rnjA*+IPTG) cells. The blot was probed with 5′-labeled oligo CCB813 (Table S7) and reprobed with oligo HP246 against 5S rRNA. The half-life of the *fosB* transcript is given under the Northern blot. Migration positions of RNA markers are shown to the right of the blot. (C) Northern blot of total mRNA isolated at times after addition of rifampicin (rif) from wild-type and RNase III depleted (*Pspac-rnc*−IPTG) and RNase III induced (*Pspac-rnc*+IPTG) cells. Description as in panel (B).
(TIF)

Figure S4 Degradation of the *yknWXYZ* mRNA depends on RNase J1 while its transcription is dependent on RNase III. (A) Structure and predicted degradation pathway of the *yknWXYZ* transcript. ORFs are shown as large white arrows, transcript as a thin black arrow. The ‘Pacman’ symbol represents 5′-3′ degradation by RNase J1. A short thick line indicates the position of the probe used. Pσ^W^ indicates the approximate promoter position and relevant sigma factor mapped in [31]. The schematic also depicts RNase III initiated degradation of a transcript encoding an unknown factor X early in the SigW cascade. (B) Northern blot of total mRNA isolated at times after addition of rifampicin (rif) from wild-type and RNase J1 depleted (*Pspac-rnjA*−IPTG) and RNase J1 induced (*Pspac-rnjA*+IPTG) cells. The blot was probed with 5′-labeled oligo CCB814 (Table S7) and reprobed with oligo HP246 against 5S rRNA. The half-life of the *yknWXYZ* transcript is given under the Northern blot. Migration positions of RNA markers are shown to the right of the blot. (C) Northern blot of total mRNA isolated at times after addition of rifampicin (rif) from wild-type and RNase III depleted (*Pspac-rnc*−IPTG) and RNase III induced (*Pspac-rnc*+IPTG) cells. Description as in panel (B).
(TIF)

Figure S5 Transcription of the *yfhLM* mRNA is stimulated in an RNase III mutant. (A) Structure and predicted degradation pathway of the *yfhLM* transcript. ORFs are shown as large white arrows, transcript as a thin black arrow. A short thick line indicates the position of the probe used. Pσ^W^ indicates the putative promoter and sigma factor for the transcript predicted by the DBTBS website (http://dbtbs.hgc.jp/) and in agreement with the size detected by Northern blot. The schematic also depicts RNase III initiated degradation of a transcript encoding an unknown factor X early in the SigW cascade. (B) Northern blot of total mRNA isolated at times after addition of rifampicin (rif) from wild-type and RNase III depleted (*Pspac-rnc*−IPTG) and RNase III induced (*Pspac-rnc*+IPTG) cells. The blot was probed with 5′-labeled oligo CCB809 (Table S7) and reprobed with oligo HP246 against 5S rRNA. The half-life of the *yfhLM* transcript is given under the Northern blot. Migration positions of RNA markers are shown to the right of the blot.
(TIF)

Figure S6 Transcription of the *yrkA* mRNA is stimulated in an RNase III mutant. (A) Structure and predicted degradation pathway of the *yrkA* transcript. ORFs are shown as large white arrows, transcript as a thin black arrow. A short thick line indicates the position of the probe used. Pσ^A^ indicates the putative promoter and sigma factor for the transcript predicted by the DBTBS website (http://dbtbs.hgc.jp/) and in agreement with the size detected by Northern blot. The schematic also depicts RNase III initiated degradation of a transcript encoding an unknown factor X that activates yrkA transcription. (B) Northern blot of total mRNA isolated at times after addition of rifampicin (rif) from wild-type and RNase III depleted (*Pspac-rnc*−IPTG) and RNase III induced (*Pspac-rnc*+IPTG) cells. The blot was probed with 5′-labeled oligo CCB811 (Table S7) and reprobed with oligo HP246 against 5S rRNA. The half-lives of the *yrkA* transcripts (A, B) are given under the Northern blot. Migration positions of RNA markers are shown to the right of the blot.
(TIF)

Figure S7 Effects of RNase J1, Y and III depletion on abundance of new *B. subtilis* segments. The Venn diagrams show the number of (A) 5′-UTRs, (B) potential sRNAs and (C) asRNAs altered in each of the three mutant strains CCB034 (RNase J1), CCB294 (RNase Y) and CCB288 (RNase III). Upward pointing arrows indicate the number of RNAs identified by Nicolas *et al.* (unpublished data) showing increased abundance; downward pointing arrows indicate decreased abundance. The areas of the circles are proportional to the number of RNAs showing altered abundance in each strain. The total number of RNAs affected in the experiment is shown in the rectangle to the right of each Venn diagram.
(TIF)

Figure S8 Both transcription and degradation of the *ybeC* transcript are dependent on RNase J1. (A) Structure and predicted degradation pathway of *ybeC*. ORFs are shown as large white arrows, transcript as a thin black arrow. The ‘Pacman’ symbol represents 5′-3′ degradation by RNase J1. A short thick line indicates the position of the probe used. Pσ^A^ indicates the putative promoter and sigma factor for the transcript predicted by the DBTBS website (http://dbtbs.hgc.jp/) and in agreement with the size detected by Northern blot. (B) Northern blot of total mRNA isolated at times after addition of rifampicin (rif) from wild-type and RNase J1 depleted (*Pspac-rnjA*−IPTG) and RNase Y induced (*Pspac-rnjA*+IPTG) cells. The blot was probed with 5′-labeled oligo CCB825 (Table S7) and reprobed with oligo HP246 against 5S rRNA. The half-life of the transcript is given under the Northern blot. Migration positions of RNA markers are shown to the right of the blot.
(TIF)

Figure S9 Degradation of the 5′-UTR (S935) of the *yqzDC* operon mRNA is dependent on RNase Y. (A) Structure and predicted degradation pathway of S935. ORFs are shown as large white arrows, transcript as a thin black arrow. Scissors indicate cleavage by RNase Y; ‘Pacman’ symbols represent 5′-3′ degradation by RNase J1. A short thick line indicates the position of the probe used. Pσ^B^ indicates the putative promoter and sigma factor for the transcript predicted by the DBTBS website (http://dbtbs.hgc.jp/) and in agreement with the size detected by Northern blot. (B) Northern blot of total mRNA isolated at times after addition of rifampicin (rif) from wild-type and RNase Y depleted (*Pspac-rny*−IPTG) and RNase Y induced (*Pspac-rny*+IPTG) cells. The blot was probed with 5′-labeled oligo CCB821 (Table S7) and reprobed with oligo HP246 against 5S rRNA. The half-life of the transcript is given under the Northern blot. Migration positions of RNA markers are shown to the right of the blot.
(TIF)

Table S1 Expression levels of *rnc*, *rnjA*, *rny* and downstream genes compared to wild-type.
(DOCX)

Table S2 Fold changes in annotated genes. Column labeled ‘WT’ is the average log2 value measured in the wild-type strain. Columns labeled ‘ΔRNase III’, ΔRNase J1’ and ‘ΔRNase Y’ show the average log2 difference compared to the wild-type for each RNase depleted strain. Columns labeled ‘Qval_RNase III’, ‘Qval_RNase J1’ and ‘Qval_RNase Y’ show calculated q-values for each gene associated with the testing of a non-null difference between each RNase depleted strain and the wild-type. Columns labeled ‘Pval_RNase III’, ‘Pval_RNase J1’, and ‘Pval_RNase Y’ show the raw p-values used for the calculation of the q-values. Columns labeled ‘Profile III/J1/Y’ (2) or (1.5) indicates increased (U), decreased (D) or unchanged (−) expression levels (Q-value≤0.1) that are 2-fold or 1.5-fold greater than WT, respectively. Data sheets labeled “All 3 RNases”, “RNase III & J1”, “RNase Y” etc. show candidate genes common to different RNase depletion strains with a 2-fold increased or decreased abundance (q-value≤0.1) compared to WT (in absolute values).
(XLSX)

Table S3 Fold changes in published potential regulatory RNAs. Column labeled ‘WT’ is the average log2 value measured in the wild-type strain. Columns labeled ‘ΔRNase III’, ΔRNase J1’ and ‘ΔRNase Y’ show the average log2 difference compared to the wild-type for each RNase depleted strain Columns labeled ‘Qval_RNase III’, ‘Qval_RNase J1’ and ‘Qval_RNase Y’ show calculated q-values for each gene associated with the testing of a non-null difference between each RNase depleted strain and the wild-type. Columns labeled ‘Pval_RNase III’, ‘Pval_RNase J1’, and ‘Pval_RNase Y’ show the raw p-values used for the calculation of the q-values. Columns labeled ‘Profile III/J1/Y’ (2) or (1.5) indicates increased (U), decreased (D) or unchanged (−) expression levels (Q-value≤0.1) that are 2-fold or 1.5-fold greater than WT, respectively. Data sheets labeled “All 3 RNases”, “RNase III & J1”, “RNase Y” etc. show candidate genes common to different RNase depletion strains with a 2-fold increased or decreased abundance (q-value≤0.1) compared to WT (in absolute values).
(XLSX)

Table S4 New RNA segments stabilized in RNase mutants. Column labeled ‘WT’ is the average log2 value measured in the wild-type strain. Columns labeled ‘ΔRNase III’, ΔRNase J1’ and ‘ΔRNase Y’ show the average log2 difference compared to the wild-type for each RNase depleted strain. Columns labeled ‘Qval_RNase III’, ‘Qval_RNase J1’ and ‘Qval_RNase Y’ show calculated q-values for each gene associated with the testing of a non-null difference between each RNase depleted strain and the wild-type. Columns labeled ‘Pval_RNase III’, ‘Pval_RNase J1’, and ‘Pval_RNase Y’ show the raw p-values used for the calculation of the q-values. Columns labeled ‘Profile III/J1/Y’ (2) or (1.5) indicates increased (U), decreased (D) or unchanged (−) expression levels (Q-value≤0.1) that are 2-fold or 1.5-fold greater than WT, respectively. Data sheets labeled “All 3 RNases”, “RNase III & J1”, “RNase Y” etc. show candidate genes common to different RNase depletion strains with a 2-fold increased or decreased abundance (q-value≤0.1) compared to WT (in absolute values). Segments labeled “S” were also found in the study by Nicolas *et al.* (unpublished data); segments unique to this study are labeled “T”. Column labeled ‘Nicolas Class’ refers to the classification of each segment in the study by Nicolas *et al.* (unpublished data), defined as follows: 3′: 3′ untranslated region (UTR) ending with a signal downshift. 3′MT: 3′ transcribed segment lacking a site for transcription termination (no abrupt signal downshift) and exhibiting a 3′ extended mRNA with a slow decrease in the signal. 3′PT: 3′ transcribed segment resulting from a partial (incomplete) termination of transcription. 5′: 5′ UTR. Indep: independent segment transcribed from its own promoter and ending with a downshift. IndepMT: independent segments transcribed from their own promoter that do not end with a downshift but exhibit a 3′ extended mRNA with a slow decrease in the signal intensity. Inter: a transcribed segment between two genes with distinct promoters. Intra: transcribed segment lying between two genes under the control of the same promoter.
(XLSX)

Table S5 Comparison with RNase Y substrates in Lehnik-Habrink study. Data sheets list candidate genes 2-fold and 1.5-fold greater than WT (“2× up” and “1.5× up”, respectively) and 2-fold and 1.5-fold decreased compared to WT (“2×-down” and 1.5× down”, respectively) in present study also found in Lehnik-Habrink study [24].
(XLSX)

Table S6 Oligonucleotides used in this study. Hybridizing sequences in first PCR cycle are in upper case; non-hybridizing sequences are lower case.
(DOCX)

Table S7 Functional categories of genes showing altered expression in RNase mutants. Functional categories are from Subtiwiki v20110601, level 3 (http://subtiwiki.uni-goettingen.de/wiki/index.php/Categories). The percentage of genes in each functional category showing significantly increased abundance (%Up), not significantly changed (%-), or significantly decreased abundance (%Down) is shown for each RNase. The p-value of the Fisher exact test for rejecting the null hypothesis of a distribution similar to that of the whole gene set is provided for each enzyme. Significant p-values (0.05 level after Bonferroni correction for multiple testing) compared to the expected distribution for the null hypothesis are highlighted in the columns labeled ‘summary’. The “+Up” and “−Up” codes refer to over- and under-representation of up-regulated genes, while “+Down” and “−Down”, refer to over- and under-representation of down-regulated genes, respectively. Over-represented categories are highlighted in green; under-represented categories in orange; reference values for the whole geneome in yellow.
(XLSX)
