# Supplementary material for: Three Essential Ribonucleases—RNase Y, J1, and III—Control the Abundance of a Majority of Bacillus subtilis mRNAs
Source: PLoS Genet. 2012 Mar 8;8(3):e1002520. doi: 10.1371/journal.pgen.1002520 (PMC3297567; doi:10.1371/journal.pgen.1002520)
Supplement: Figure S1 — Trace of the expression data along the whole genome. Lanes are as follows (from top to bottom): (1) Genbank annotation, (2) effect of the depletion of each RNase (log2 ratio −IPTG to wt, calculated on normalized values) on the positive strand, (3) expression signal of wt and RNase depeleted strains (normalized log2 values) on the positive strand, (4) summary of the gene-level statistical analysis, (5) expression signal on the negative strand, (6) effect of the depletion of each RNase on the negative strand. The color code for experiments is: wild-type, green; RNase III, violet; RNase J1, blue; RNase Y, red. In the plots of expression signal (lanes 3 and 5), the horizontal black line represents the global median over the whole chromosome and the two horizontal gray lines indicate 5× and 10× this value. In the plots of log2 ratios, the horizontal black line corresponds to base-line (no change) and two horizontal gray lines on either side indicate 2× up and 2× down changes. The summary of the gene-level statistical analysis shows which genes or expression segments were affected by the depletion of at least one of the three RNases: thick green line, gene showing decreased expression in at least one RNase depletion experiment; thick violet, blue or red line, gene showing increased expression in at least one RNase depletion experiment (in this case, the color indicates which depletion was observed to have the greatest effect); thick gray line, gene showing both increased and decreased expression depending on the RNase considered. Color codes for the Genbank annotation are as follows: cyan and magenta, annotated protein coding sequences on the positive and negative strands, respectively (solid symbol when function is known; hollow symbol when function is considered unknown in Genbank); red, ribosomal RNA; dark blue, tRNA; green, Misc_RNA. Traces were plotted using MuGen [43]. Only the signal from unique oligos are shown; gaps are due to non-unique genome sequences. Note: c [file pgen.1002520.s001.pdf]

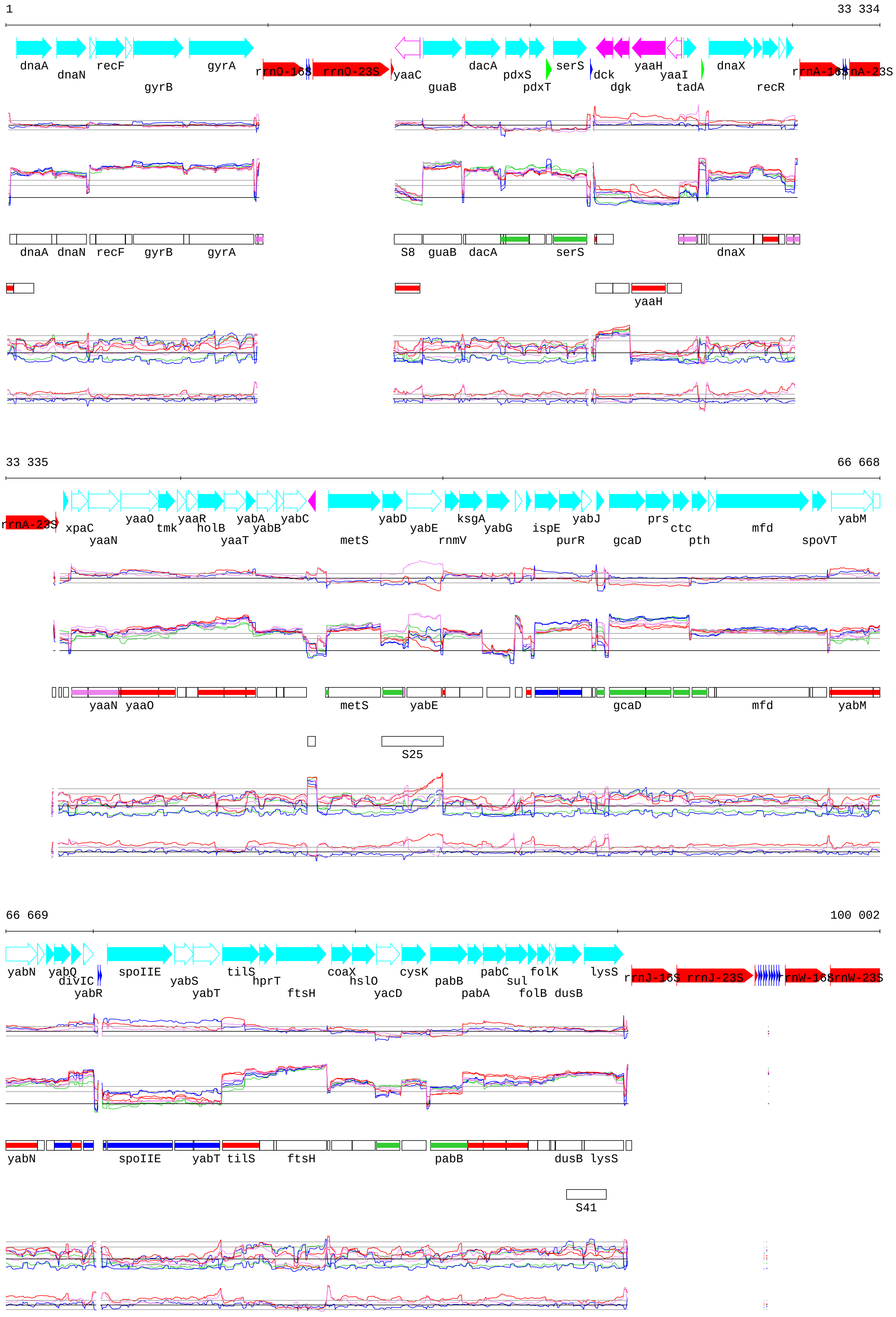

1
33 334
dnaA dnaN recF gyrB gyrA rrnO-16S rrnO-23S yaaC guaB dacA pdxS pdxT serS dck dgk yaaH yaaI tadA dnaX recR rrnA-16S rrnA-23S
dnaA dnaN recF gyrB gyrA S8 guaB dacA serS dnaX
yaaH
33 335
66 668
rrnA-23S xpaC yaaN yaaO tmk yaaR holB yaaT yabA yabB yabC metS yabD yabE ksgA rnmV yabG ispE purR yabJ gcaD prs ctc pth mfd spoVT yabM
yaaN yaaO metS yabE gcaD mfd yabM
S25
66 669
100 002
yabN yabQ divIC yabR spoIIE yabS yabT tilS hprT ftsH coaX hslO yacD cysK pabB pabC pabA sul folK folB dusB lysS rrnJ-16S rrnJ-23S rrnW-16S rrnW-23S
yabN spoIIE yabT tilS ftsH pabB dusB lysS
S41

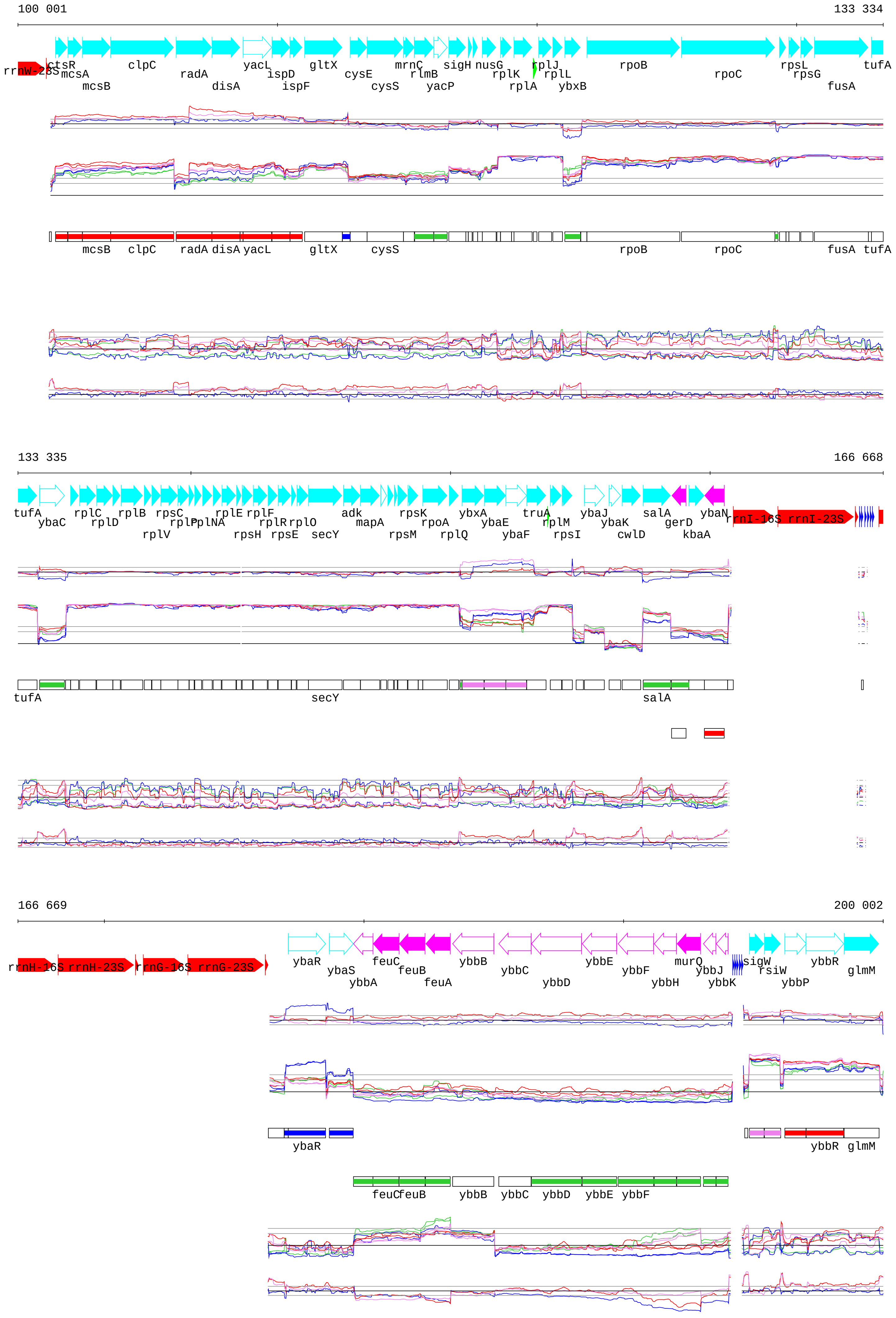

100 001
133 334
rrnW-23S
ctsR
mcsA
mcsB
clpC
radA
disA
yacL
ispD
ispF
gltX
cysE
cysS
mrnC
rlmB
yacP
sigH
nusG
rplK
rplA
rplJ
rplL
ybxB
rpoB
rpoC
rpsL
rpsG
fusA
tufA
mcsB clpC radA disA yacL gltX cysS rpoB rpoC fusA tufA
133 335
166 668
tufA
ybaC
rplC
rplD
rplB
rplV
rpsC
rplP
rplNA
rplE
rplF
rpsH
rplR
rplO
rpsE
secY
adk
mapA
rpsK
rpsM
rpoA
rplQ
ybxA
ybaE
ybaF
truA
rplM
rpsI
ybaJ
ybaK
cwlD
salA
gerD
kbaA
ybaN
rrnI-16S
rrnI-23S
tufA secY salA
166 669
200 002
rrnH-16S
rrnH-23S
rrnG-16S
rrnG-23S
ybaR
ybaS
ybbA
feuC
feuB
feuA
ybbB
ybbC
ybbD
ybbE
ybbF
ybbH
murQ
ybbJ
ybbK
sigW
rsiW
ybbP
ybbR
glmM
ybaR
ybbR glmM
feuC feuB ybbB ybbC ybbD ybbE ybbF

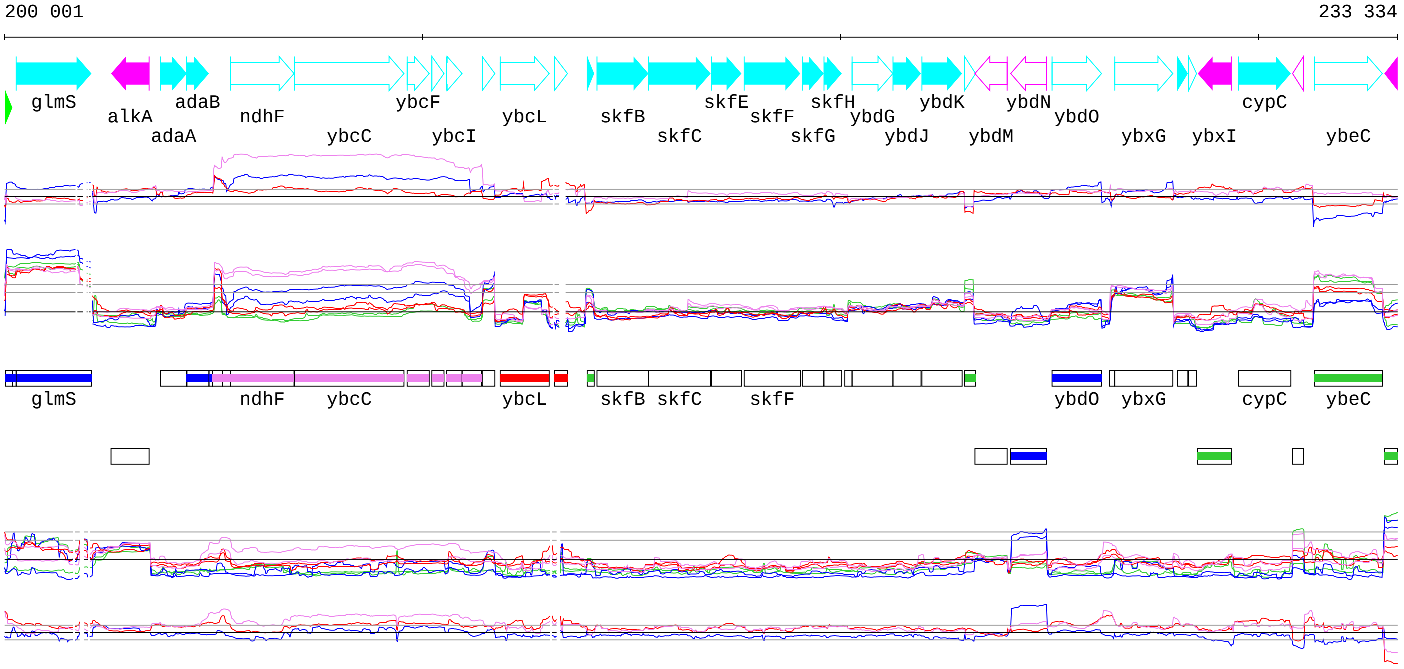
200 001
233 334
glmS alkA adaB adaA ndhF ybcC ybcF ybcI ybcL skfB skfC skfE skfF skfG skfH ybdG ybdJ ybdK ybdM ybdN ybdO ybxG ybxI cypC ybeC
glmS ndhF ybcC ybcL skfB skfC skfF ybdO ybxG cypC ybeC

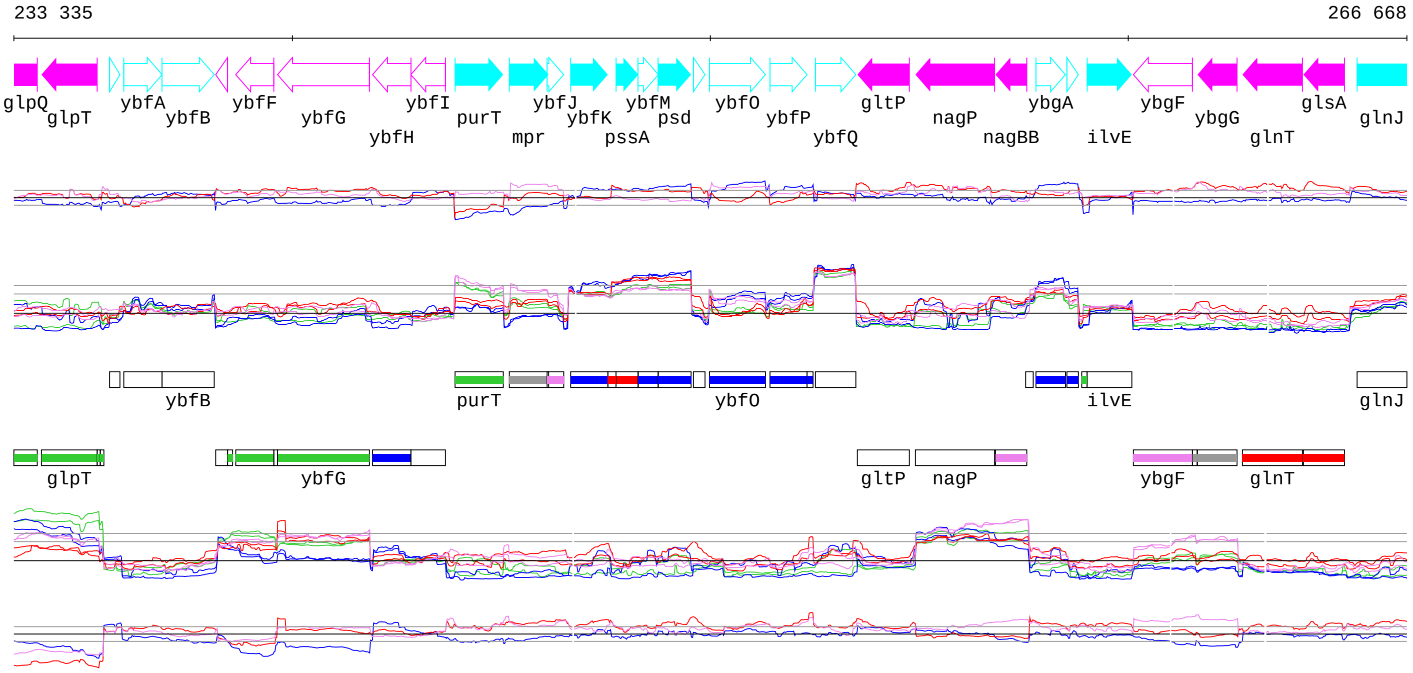
233 335
266 668
glpQ glpT ybfA ybfB ybfF ybfG ybfH ybfI purT mpr ybfJ ybfK pssA ybfM psd ybfO ybfP ybfQ gltP nagP nagBB ybgA ilvE ybgF ybgG glnT glsA glnJ
ybfB purT ybfO ilvE glnJ
glpT ybfG gltP nagP ybgF glnT

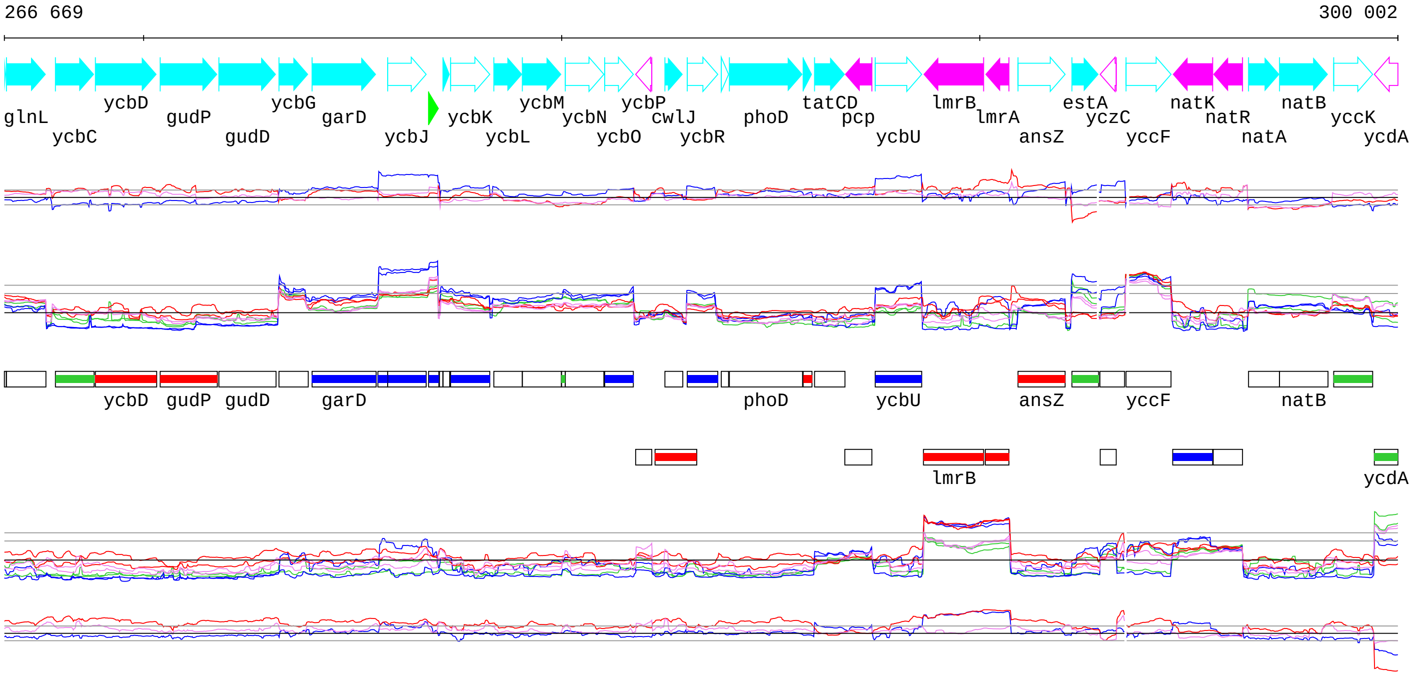
266 669
300 002
glnL ycbC ycbD gudP gudD ycbG garD ycbJ ycbK ycbL ycbM ycbN ycbO ycbP cwlJ ycbR phoD tatCD pcp ycbU lmrB lmrA ansZ estA yczC yccF natK natR natA natB yccK ycdA
ycbD gudP gudD garD phoD ycbU ansZ yccF natB
lmrB ycdA

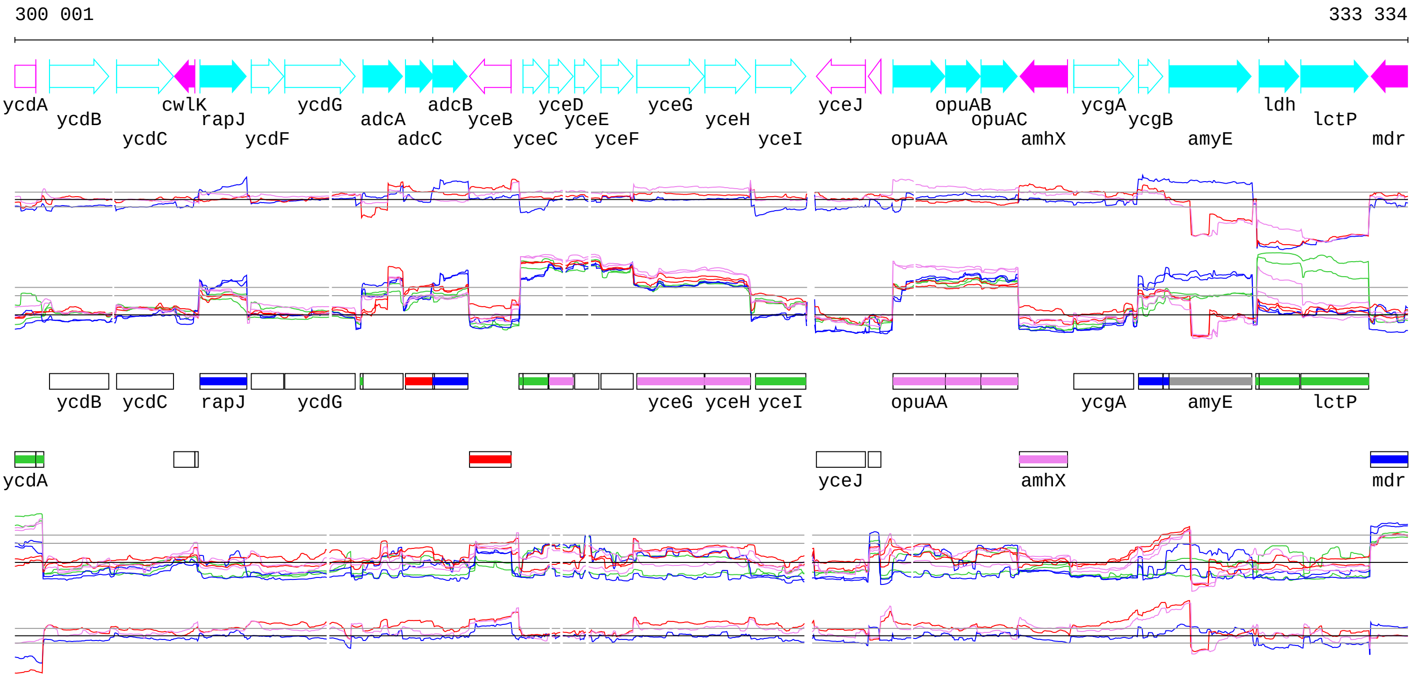
300 001
333 334
ycdA ycdB ycdC cwlK rapJ ycdF ycdG adcA adcC adcB yceB yceC yceD yceE yceF yceG yceH yceI yceJ opuAA opuAB opuAC amhX ycgA ycgB amyE ldh lctP mdr
ycdB ycdC rapJ ycdG yceG yceH yceI opuAA ycgA amyE lctP
ycdA yceJ amhX mdr

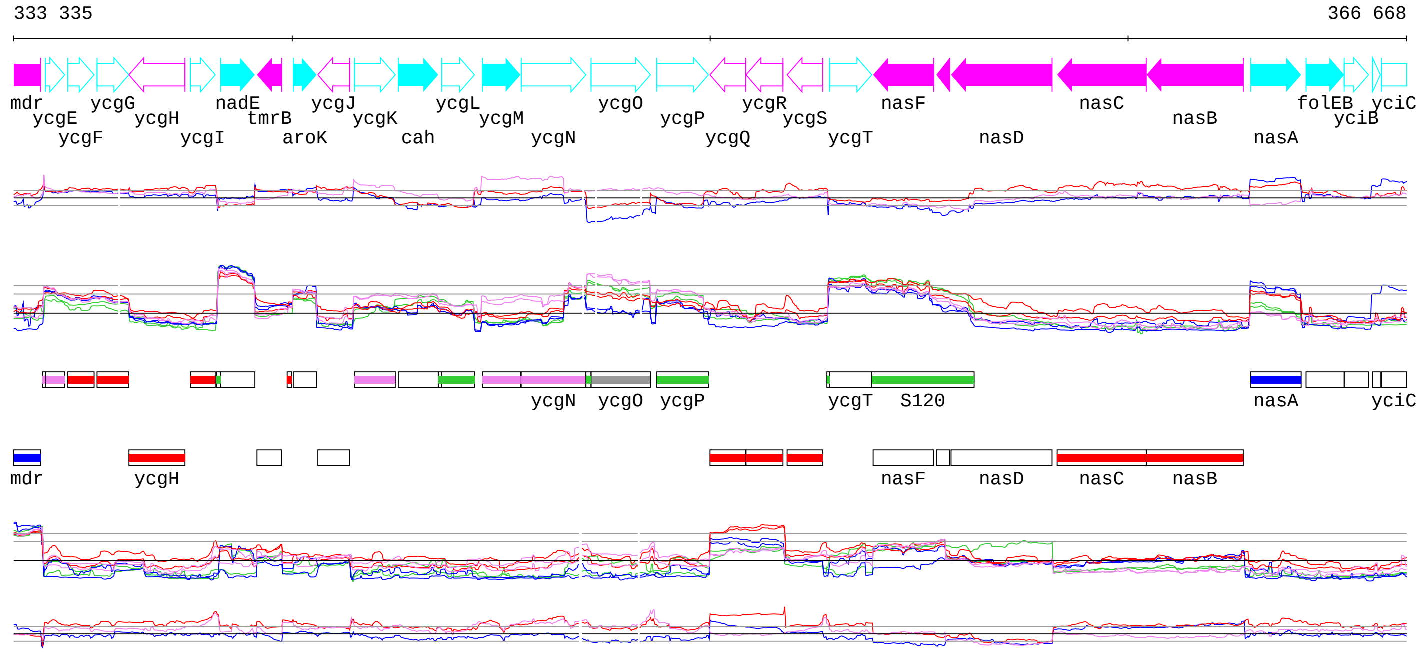
333 335
366 668
mdr ycgE ycgF ycgG ycgH ycgI nadE tmrB aroK ycgJ ycgK cah ycgL ycgM ycgN ycgO ycgP ycgQ ycgR ycgS ycgT nasF nasD nasC nasB nasA folEB yciB yciC
ycgN ycgO ycgP ycgT S120 nasA yciC
mdr ycgH nasF nasD nasC nasB

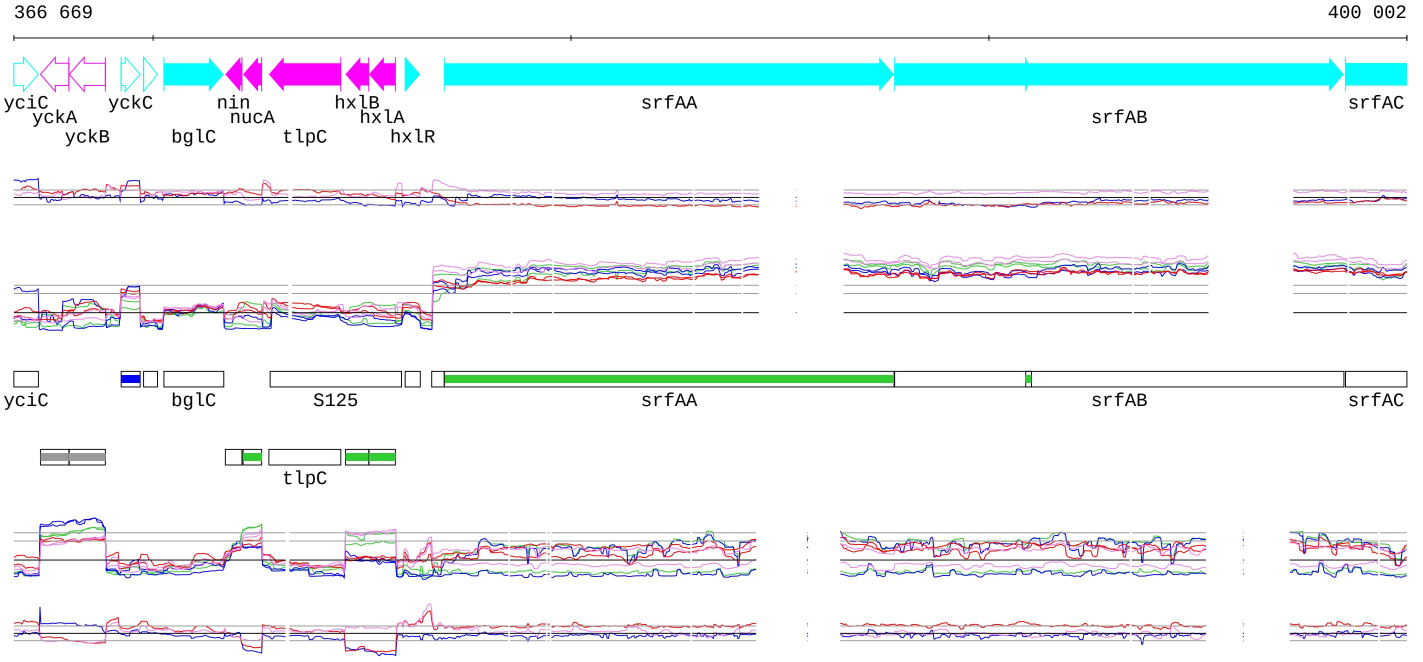
366 669
400 002
yciC yckA yckB yckC bglC nin nucA tlpC hxlB hxlA hxlR srfAA srfAB srfAC
yciC bglC S125 srfAA srfAB srfAC
tlpC

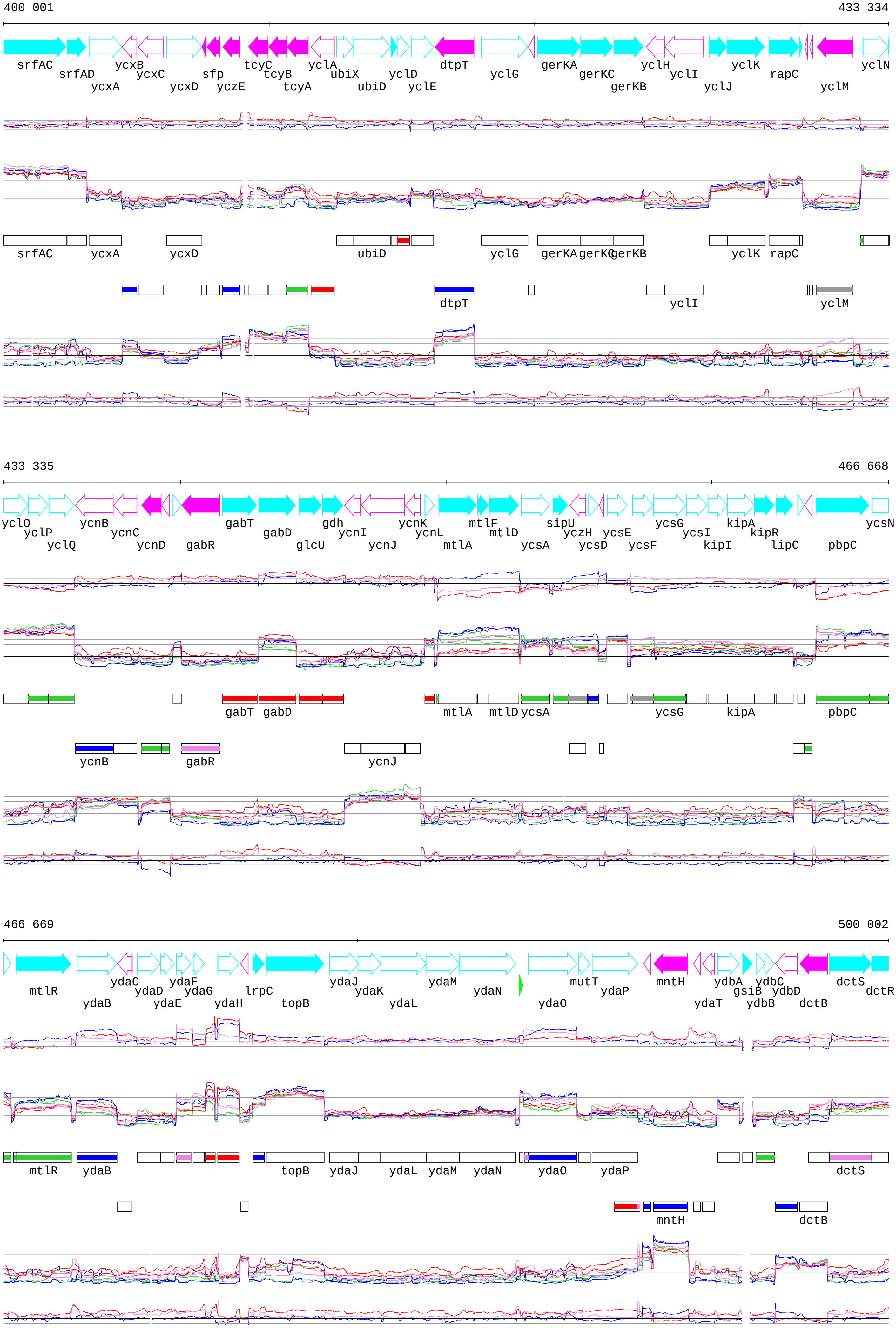
400 001
433 334
srfAC
srfAD
ycxA
ycxB
ycxC
ycxD
sfp
yczE
tcyC
tcyB
tcyA
yclA
ubiX
ubiD
yclD
yclE
dtpT
yclG
gerKA
gerKC
gerKB
yclH
yclI
yclJ
yclK
rapC
yclM
yclN
433 335
466 668
yclO
yclP
yclQ
ycnB
ycnC
ycnD
gabR
gabT
gabD
glcU
gdh
ycnI
ycnJ
ycnK
ycnL
mtlA
mtlF
mtlD
ycsA
sipU
yczH
ycsD
ycsE
ycsF
ycsG
ycsI
kipI
kipA
kipR
lipC
pbpC
ycsN
466 669
500 002
mtlR
ydaB
ydaC
ydaD
ydaE
ydaF
ydaG
ydaH
lrpC
topB
ydaJ
ydaK
ydaL
ydaM
ydaN
ydaO
mutT
ydaP
mntH
ydaT
ydbA
gsiB
ydbB
ydbC
ydbD
dctB
dctS
dctR

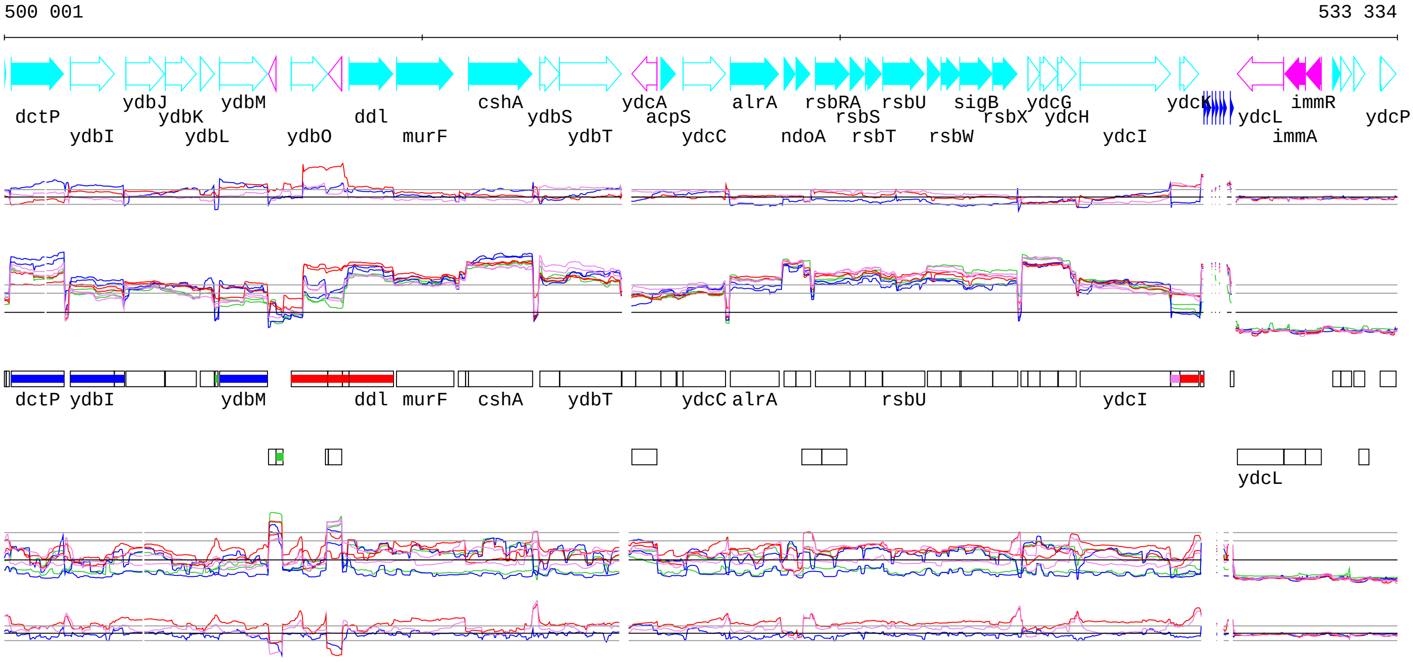
500 001
533 334
dctP ydbI ydbJ ydbK ydbL ydbM ydbO ddl murF cshA ydbS ydbT ydcA acpS ydcC alrA ndoA rsbRA rsbS rsbT rsbU rsbW sigB rsbX ydcG ydcH ydcI ydcK ydcL immR immA ydcP
dctP ydbI ydbM ddl murF cshA ydbT ydcC alrA rsbU ydcI
ydcL

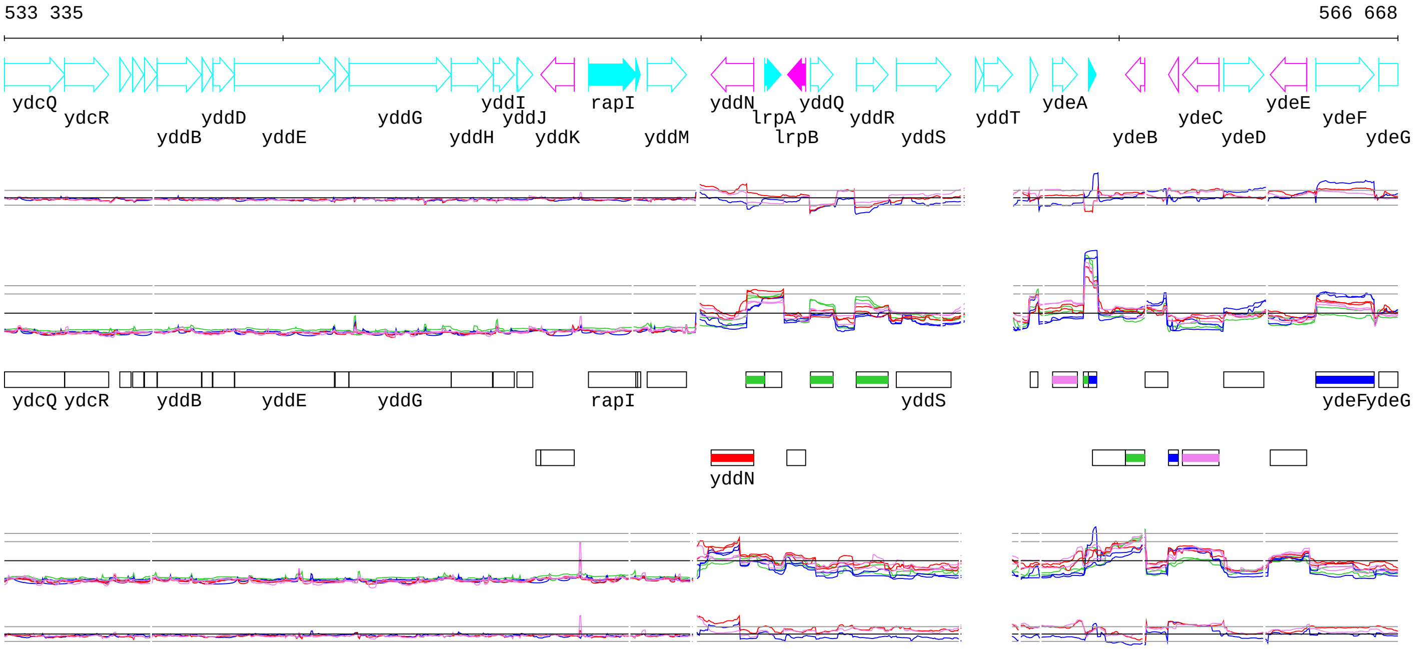
533 335
566 668
ydcQ ydcR yddB yddD yddE yddG yddH yddI yddJ yddK rapI yddM yddN lrpA lrpB yddQ yddR yddS yddT ydeA ydeB ydeC ydeD ydeE ydeF ydeG
ydcQ ydcR yddB yddE yddG rapI yddS ydeF ydeG
yddN

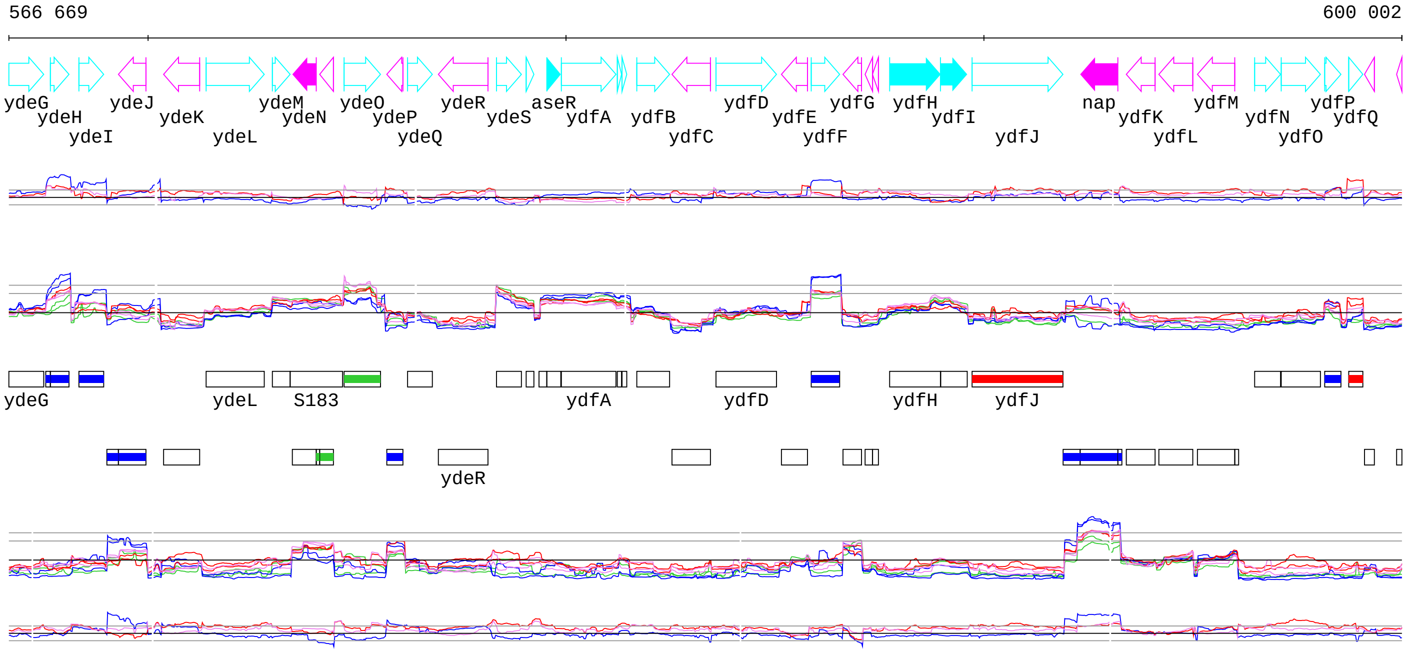
566 669
600 002
ydeG ydeH ydeI ydeJ ydeK ydeL ydeM ydeN ydeO ydeP ydeQ ydeR ydeS aseR ydfA ydfB ydfC ydfD ydfE ydfF ydfG ydfH ydfI ydfJ nap ydfK ydfL ydfM ydfN ydfO ydfP ydfQ
ydeG ydeL S183 ydfA ydfD ydfH ydfJ
ydeR

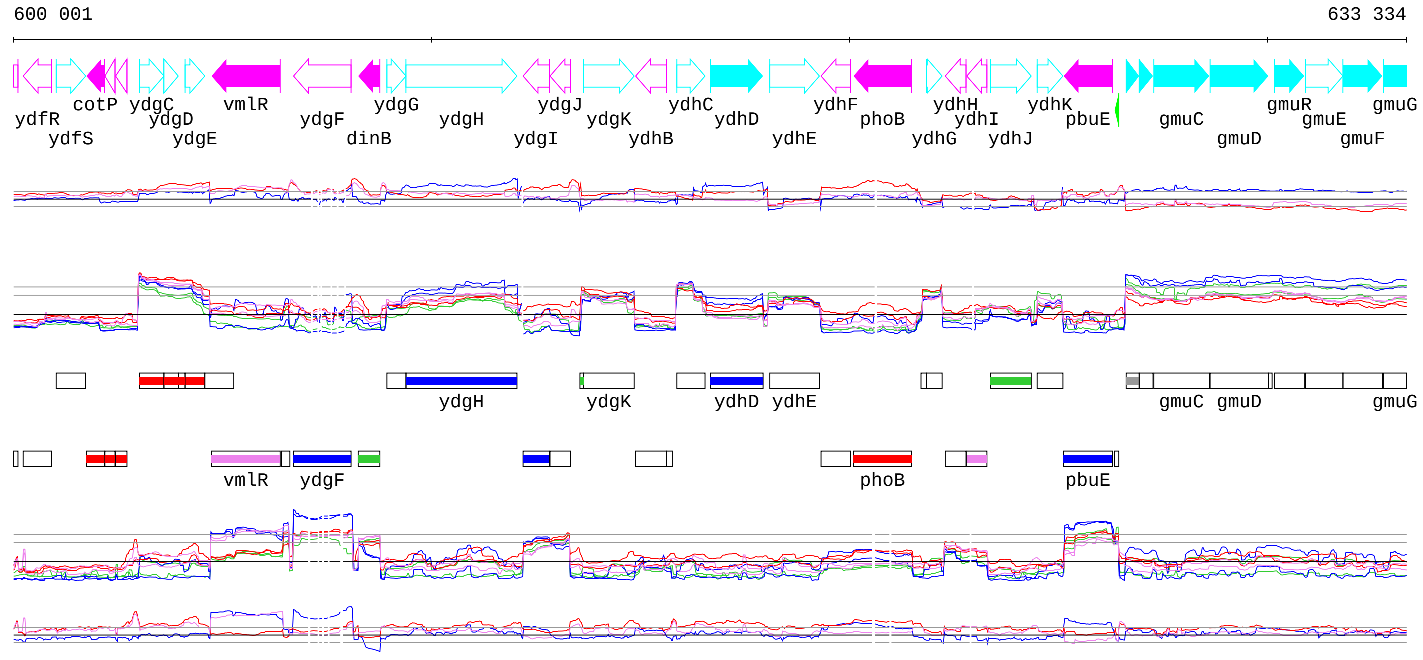
600 001
633 334
ydfR cotP ydgC ydgD vmlR ydgF ydgG dinB ydgH ydgJ ydgI ydgK ydhC ydhB ydhD ydhF ydhE phoB ydhH ydhI ydhG ydhK ydhJ pbuE gmuC gmuR gmuD gmuE gmuG gmuF
ydfS ydgE
ydgH ydgK ydhD ydhE gmuC gmuD gmuG
vmlR ydgF phoB pbuE

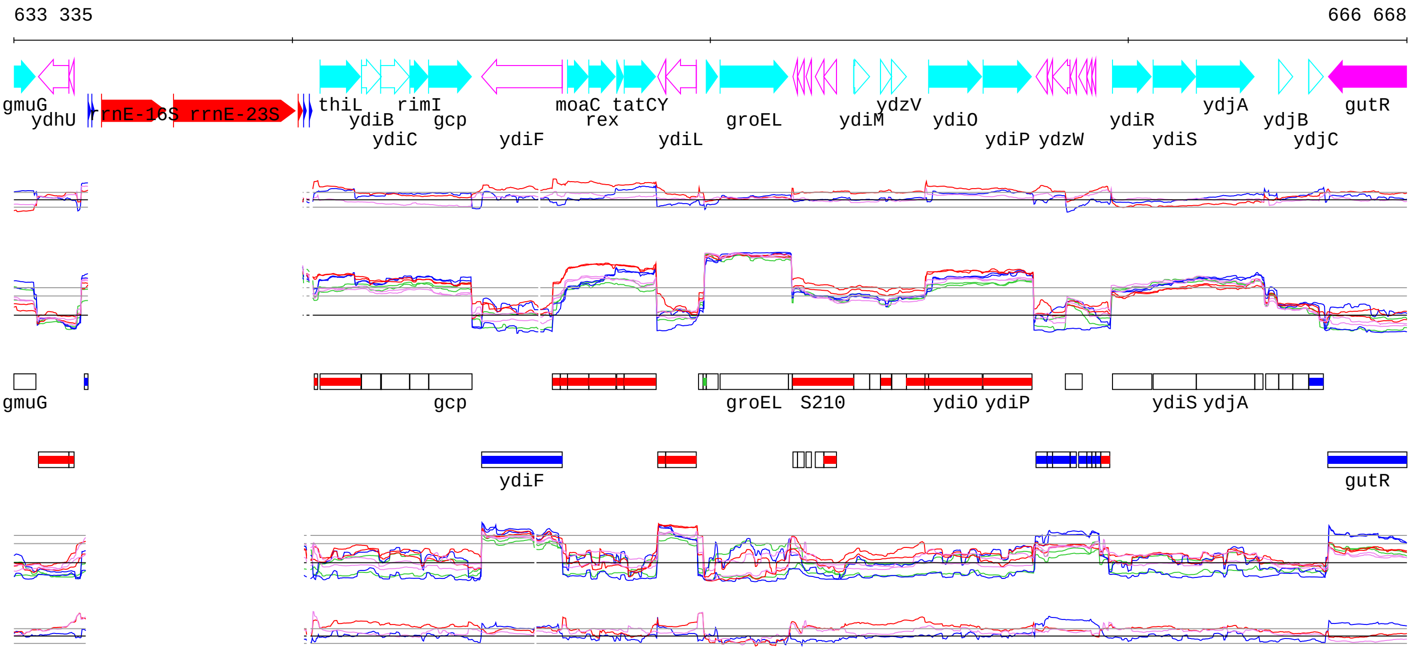
633 335
666 668
gmuG ydhU rrnE-16S rrnE-23S thiL ydiB rimI gcp ydiC ydiF moaC tatCY rex ydiL groEL ydiM ydzV ydiO ydiP ydzW ydiR ydiS ydjA ydjB ydjC gutR
gmuG gcp groEL S210 ydiO ydiP ydiS ydjA
ydiF gutR

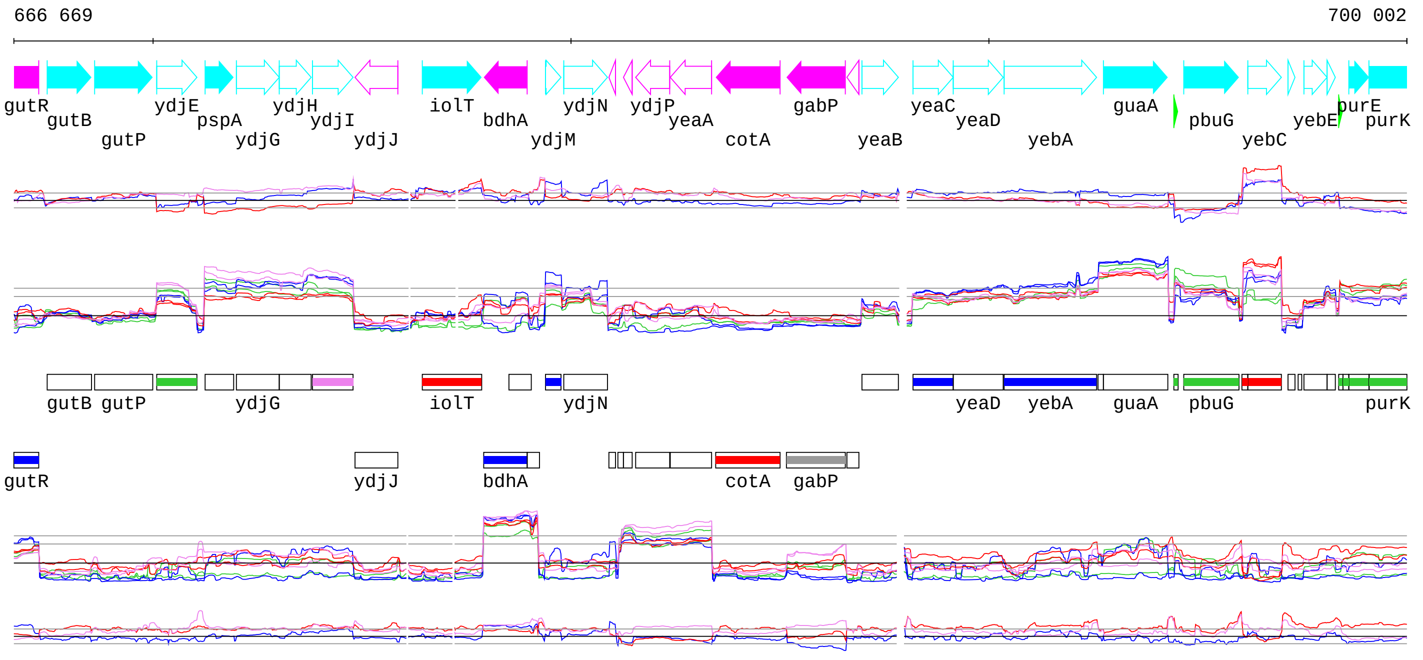
666 669
700 002
gutR gutB gutP ydjE pspA ydjG ydjH ydjI ydjJ iolT bdhA ydjN ydjM ydjP yeaA cotA gabP yeaB yeaC yeaD yebA guaA pbuG yebC yebE purE purK
gutB gutP ydjG iolT ydjN yeaD yebA guaA pbuG purK
gutR ydjJ bdhA cotA gabP

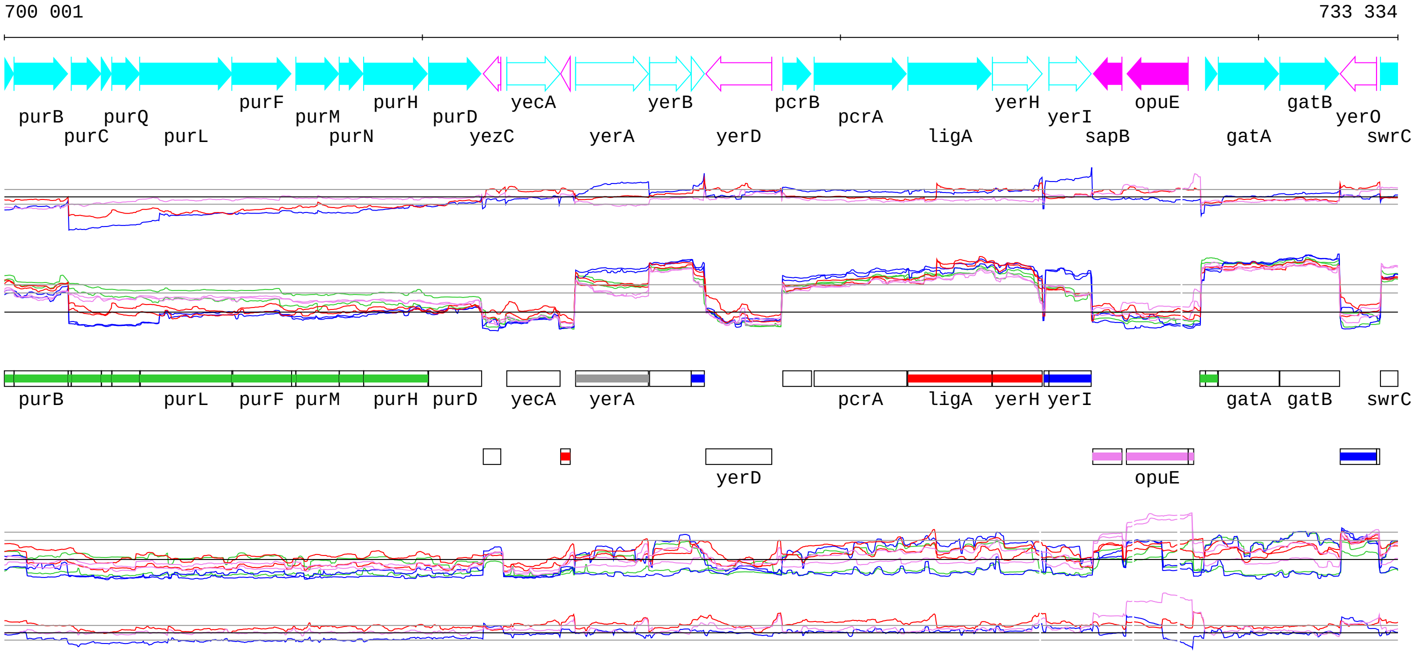
700 001
733 334
purB purQ purF purM purH purD yecA yerB pcrB pcrA yerH yerI opuE gatB yerO
purC purL purN yezC yerA yerD ligA sapB gatA swrC
purB purL purF purM purH purD yecA yerA pcrA ligA yerH yerI gatA gatB swrC
yerD opuE

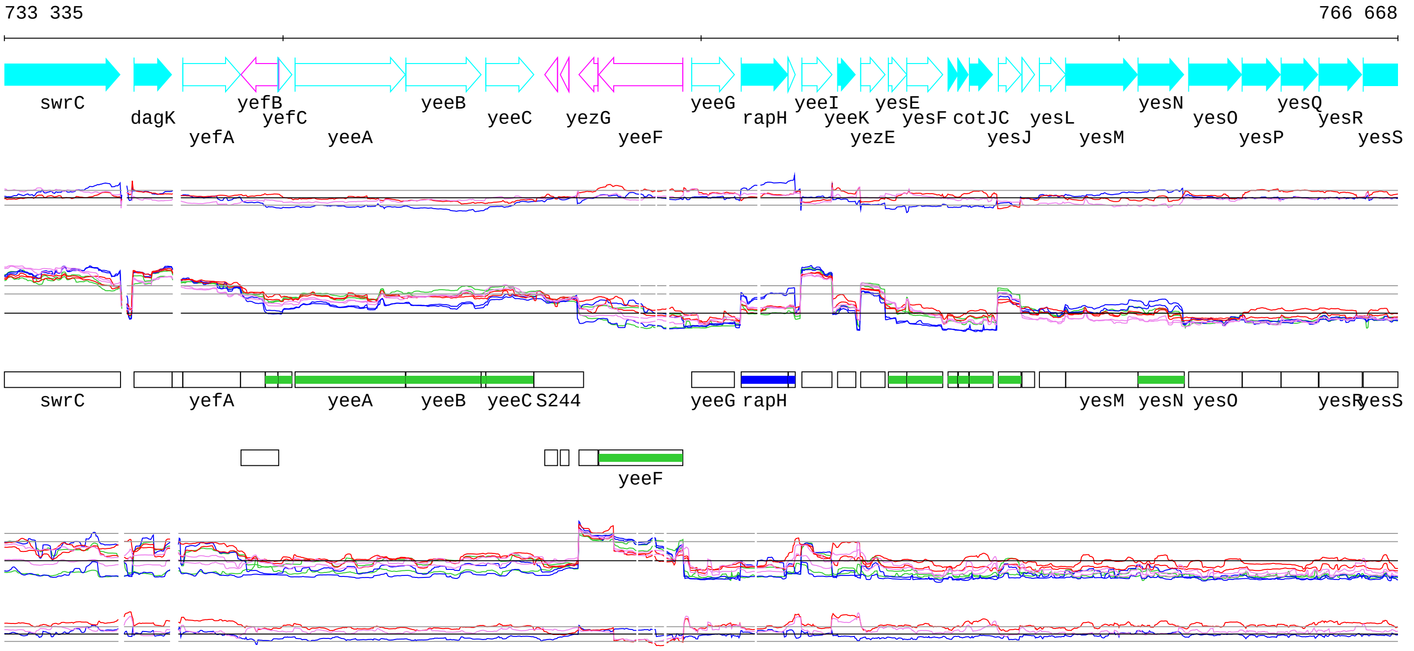
733 335
766 668
swrC dagK yefB yefC yefA yeeA yeeB yeeC yezG yeeF yeeG rapH yeeI yeeK yesE yezE yesF cotJC yesL yesJ yesM yesN yesO yesQ yesP yesR yesS
swrC yefA yeeA yeeB yeeC S244 yeeG rapH yesM yesN yesO yesR yesS
yeeF

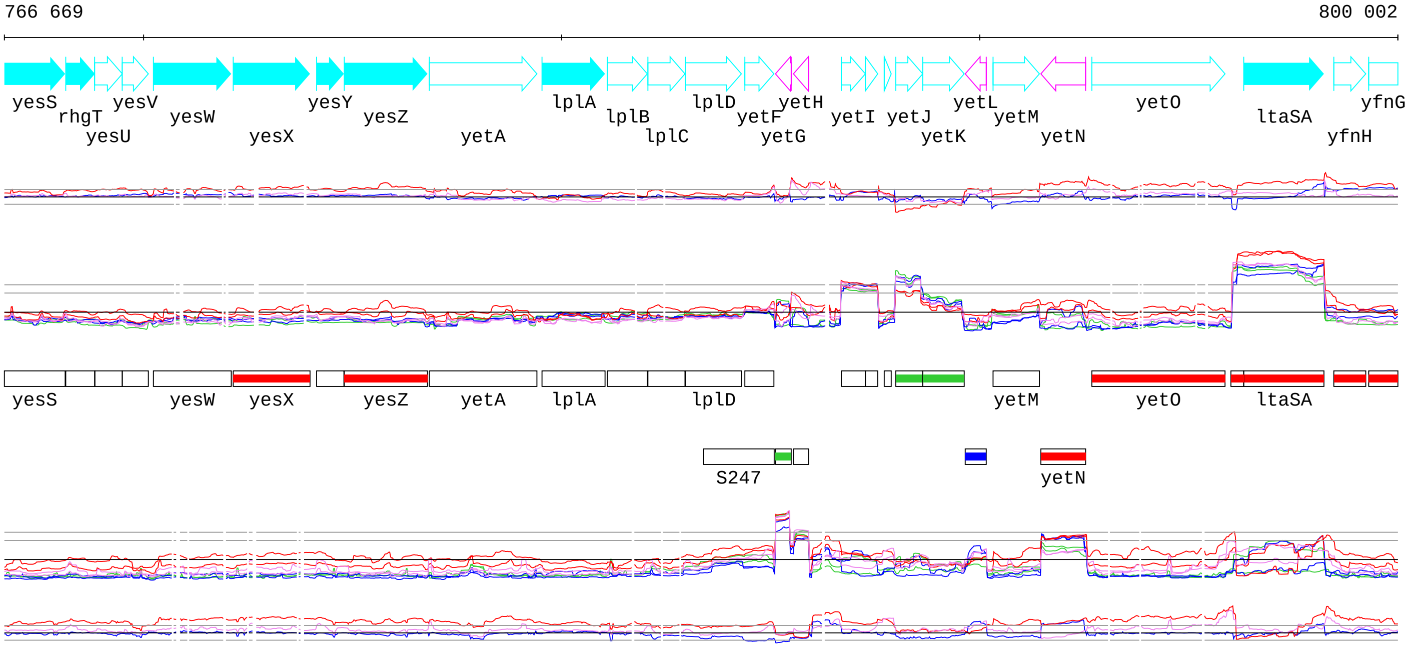
766 669
800 002
yesS rhgT yesV yesU yesW yesY yesX yesZ yetA lplA lplB lplC lplD yetF yetG yetH yetI yetJ yetK yetL yetM yetN yetO ltaSA yfnG yfnH
yesS yesW yesX yesZ yetA lplA lplD yetM yetO ltaSA
S247 yetN

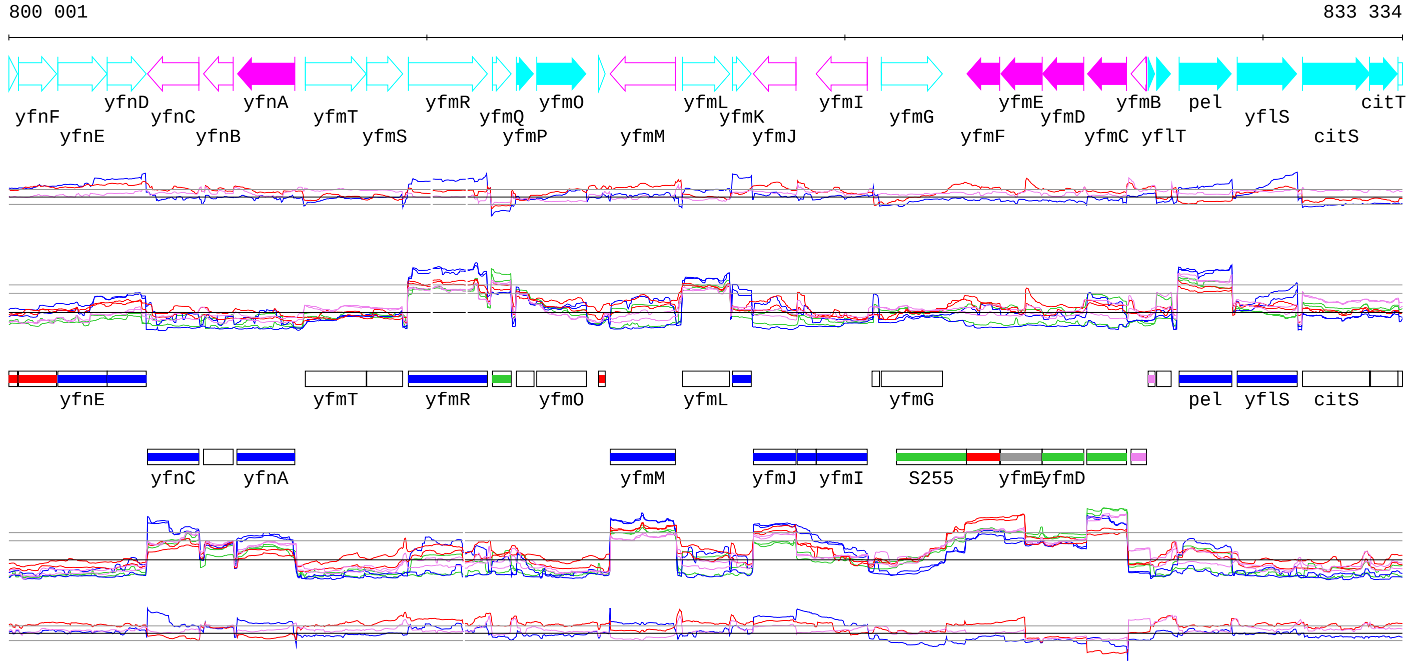
800 001
833 334
yfnF yfnE yfnD yfnC yfnB yfnA yfmT yfmS yfmR yfmQ yfmP yfmO yfmM yfmL yfmK yfmJ yfmI yfmG yfmF yfmE yfmD yfmC yfmB yflT pel yflS citS citT
yfnE yfmT yfmR yfmO yfmL yfmG pel yflS citS
yfnC yfnA yfmM yfmJ yfmI S255 yfmEyfmD

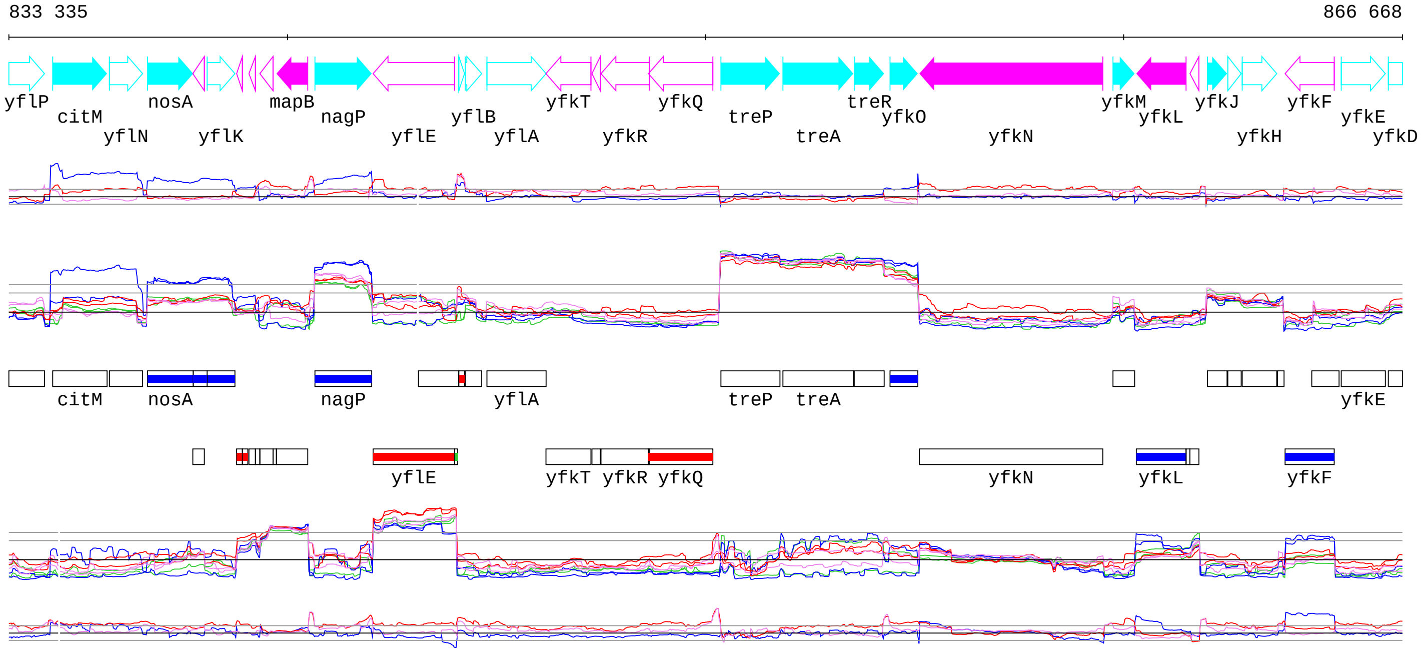
833 335
866 668
yflP citM yflN nosA yflK mapB nagP yflE yflB yflA yfkT yfkR yfkQ treP treA treR yfkO yfkN yfkM yfkL yfkJ yfkH yfkF yfkE yfkD
citM nosA nagP yflA treP treA yfkE
yflE yfkT yfkR yfkQ yfkN yfkL yfkF

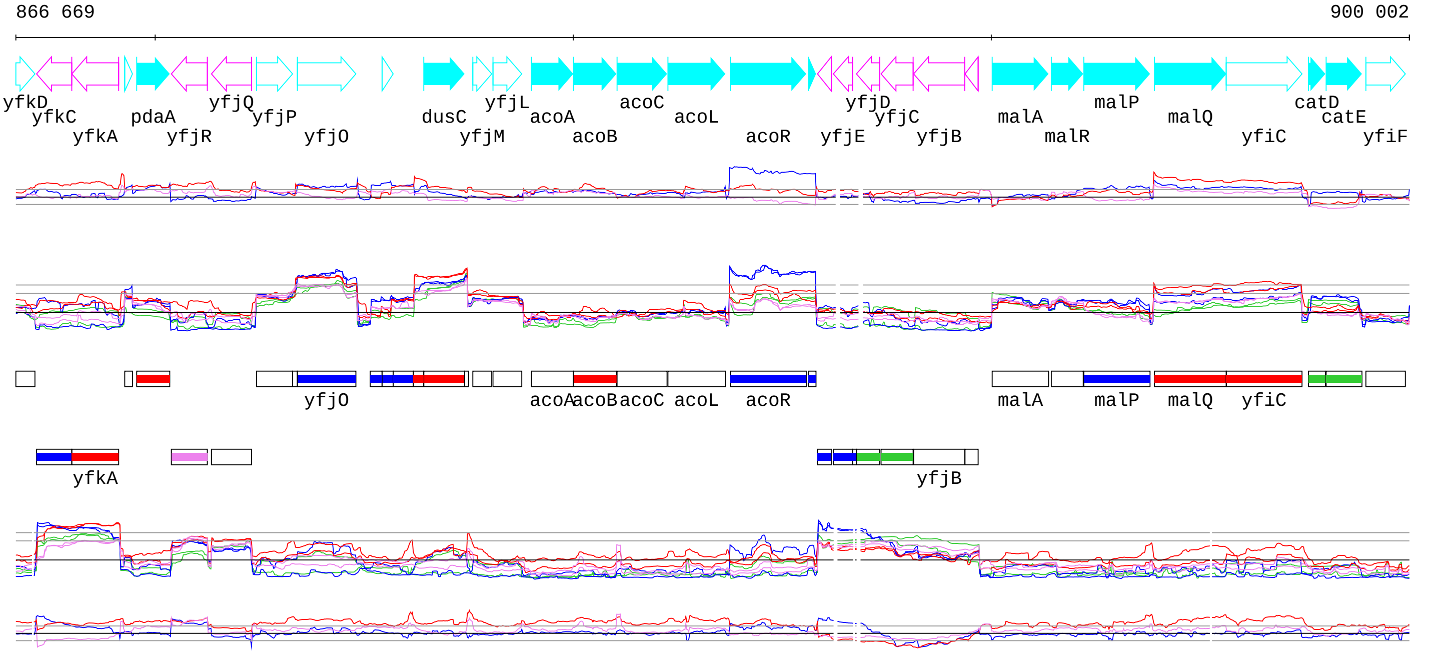
866 669
900 002
yfkD yfkC yfkA pdaA yfjR yfjQ yfjP yfjO dusC yfjM yfjL acoA acoB acoC acoL acoR yfjE yfjD yfjC yfjB malA malR malP malQ yfiC catD catE yfiF
yfjO acoAacoBacoC acoL acoR malA malP malQ yfiC
yfkA yfjB

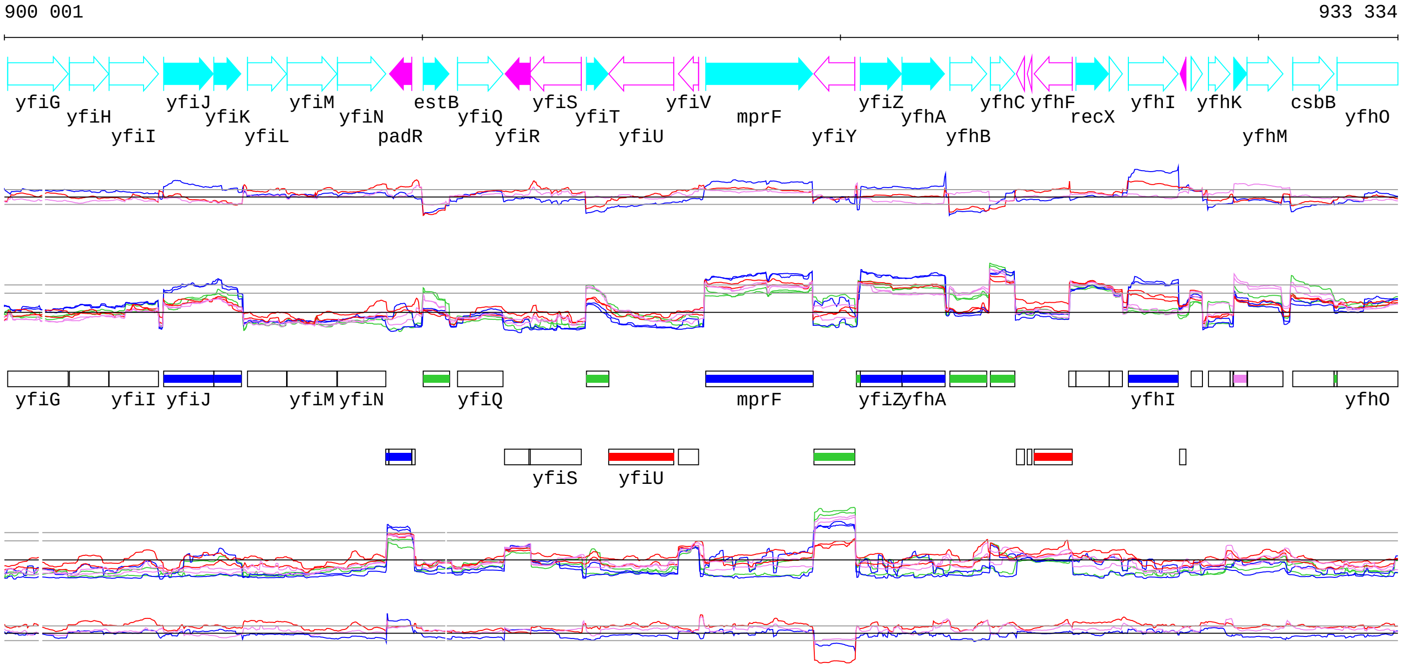
900 001
933 334
yfiG yfiJ yfiM estB yfiS yfiV yfiZ yfhC yfhF yfhI yfhK csbB
yfiH yfiK yfiN yfiQ yfiT mprF yfhA recX yfhO
yfiI yfiL padR yfiR yfiU yfiY yfhB yfhM
yfiG yfiI yfiJ yfiM yfiN yfiQ mprF yfiZyfhA yfhI yfhO
yfiS yfiU

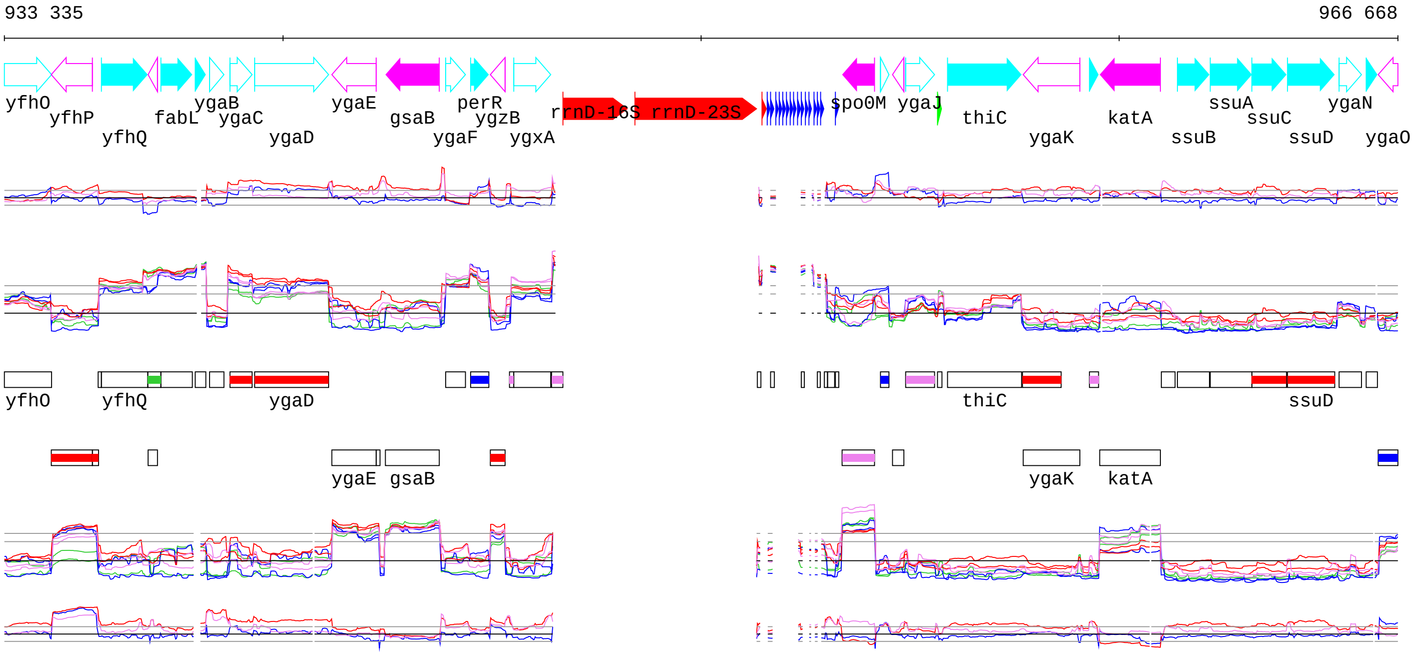
933 335
966 668
yfhO ygaB ygaE perR rrnD-16S rrnD-23S spoOM ygaJ ssuA ygaN
yfhP fabL ygaC gsaB ygzB thiC katA ssuC
yfhQ ygaD ygaF ygxA ygaK ssuB ssuD ygaO
yfhO yfhQ ygaD thiC ssuD
ygaE gsaB ygaK katA

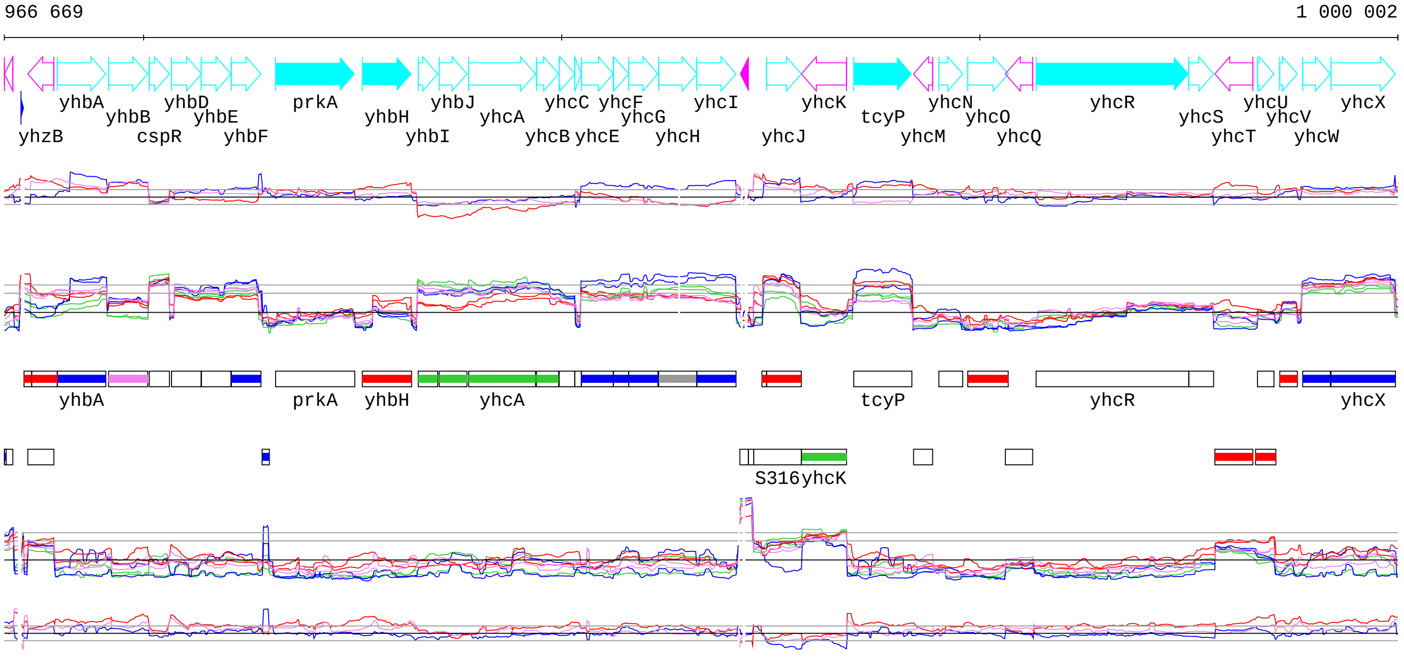
966 669
1 000 002
yhbA yhbD prkA yhbJ yhcC yhcF yhcI yhcK yhcN yhcR yhcU yhcX
yhbB yhbE yhbH yhcA yhcG tcyP yhcO yhcS yhcV
yhzB cspR yhbF yhbI yhcB yhcE yhcH yhcJ yhcM yhcQ yhcT yhcW
yhbA prkA yhbH yhcA tcyP yhcR yhcX
S316yhcK

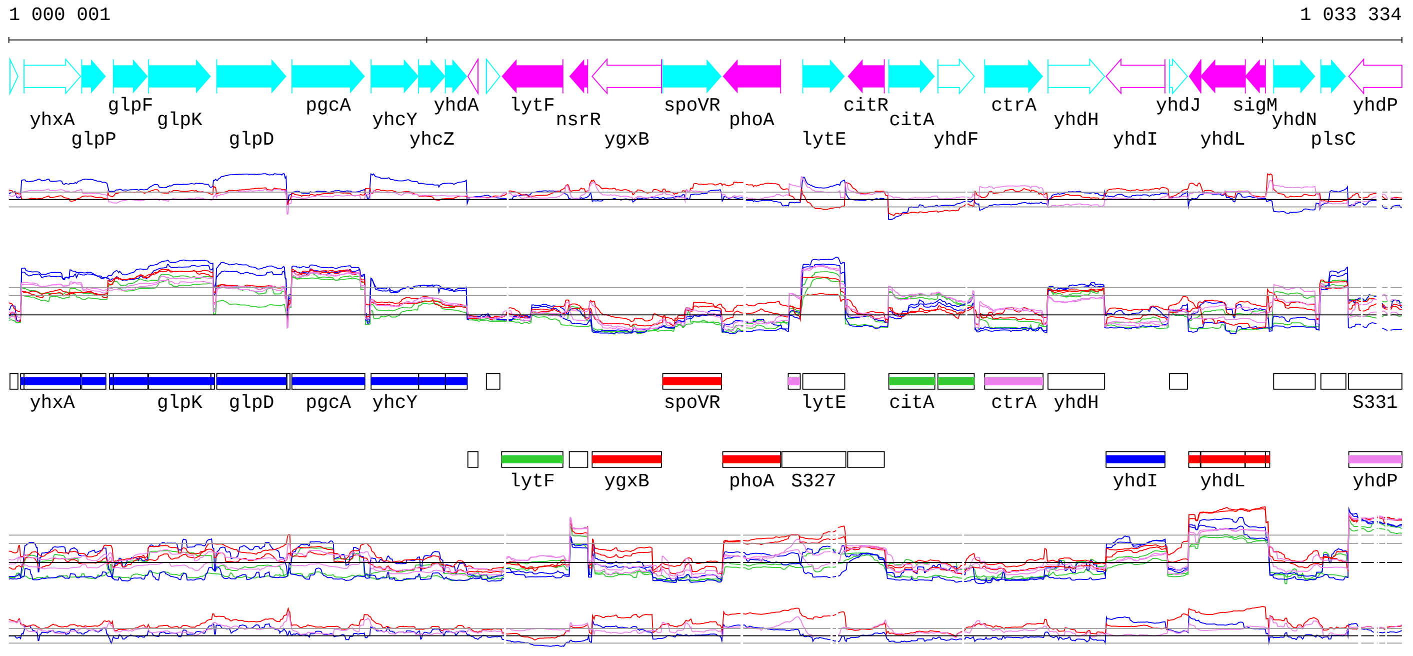
1 000 001
1 033 334
yhxA glpF glpK pgcA yhcY yhdA lytF spoVR phoA citR ctrA yhdJ sigM yhdP
glpP glpD yhcZ nsrR ygxB lytE citA yhdF yhdH yhdI yhdL yhdN plsC
yhxA glpK glpD pgcA yhcY spoVR lytE citA ctrA yhdH S331
lytF ygxB phoA S327 yhdI yhdL yhdP

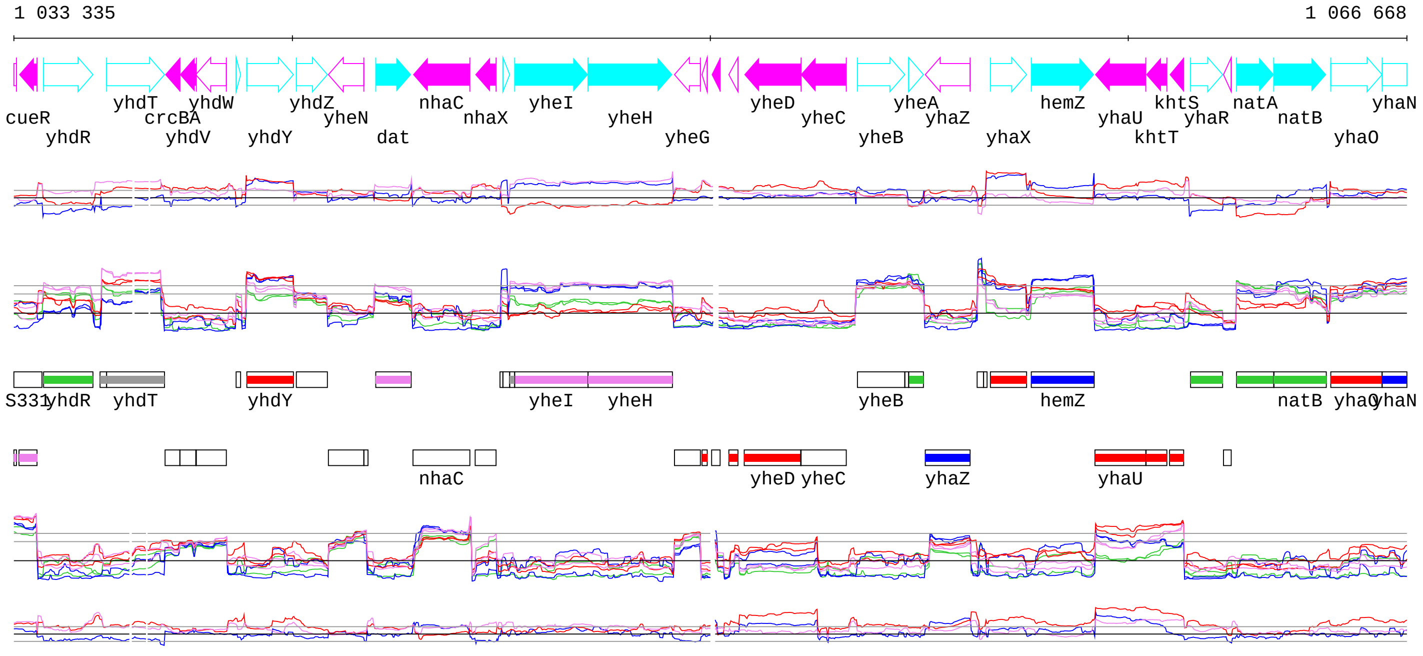
1 033 335
1 066 668
cueR yhdT crcBA yhdW yhdZ yheN nhaC nhaX yheI yheH yheD yheC yheA yhaZ hemZ yhaU khtS yhaR natA natB yhaN
yhdR yhdV yhdY dat yheG yheB yhaX khtT yhaO
S331 yhdR yhdT yhdY yheI yheH yheB hemZ natB yhaO yhaN
nhaC yheD yheC yhaZ yhaU

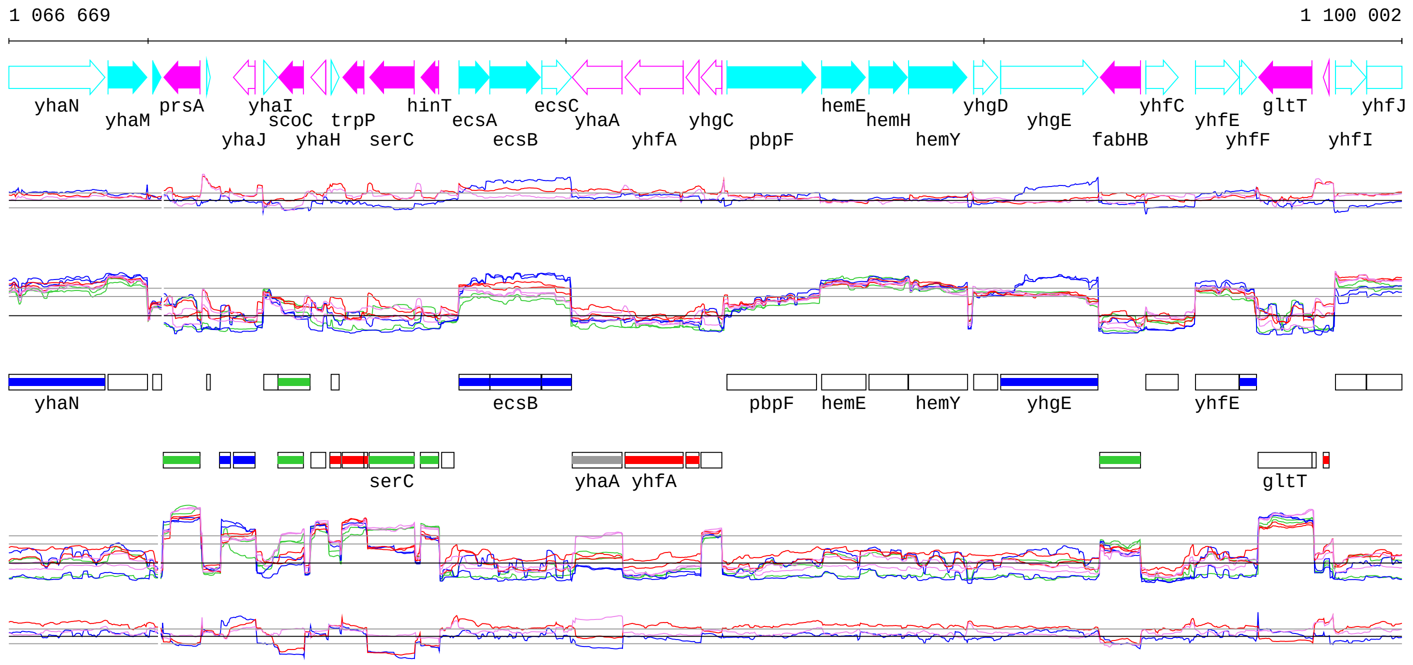
1 066 669
1 100 002
yhaN yhaM prsA yhaI scoC trpP hinT ecsA ecsC yhaA yhgC hemE hemH yhgD yhgE yhfC yhfE gltT yhfJ
yhaJ yhaH serC ecsB yhfA pbpF hemY fabHB yhfF yhfI
yhaN ecsB pbpF hemE hemY yhgE yhfE
serC yhaA yhfA gltT

1 100 001 — 1 133 334

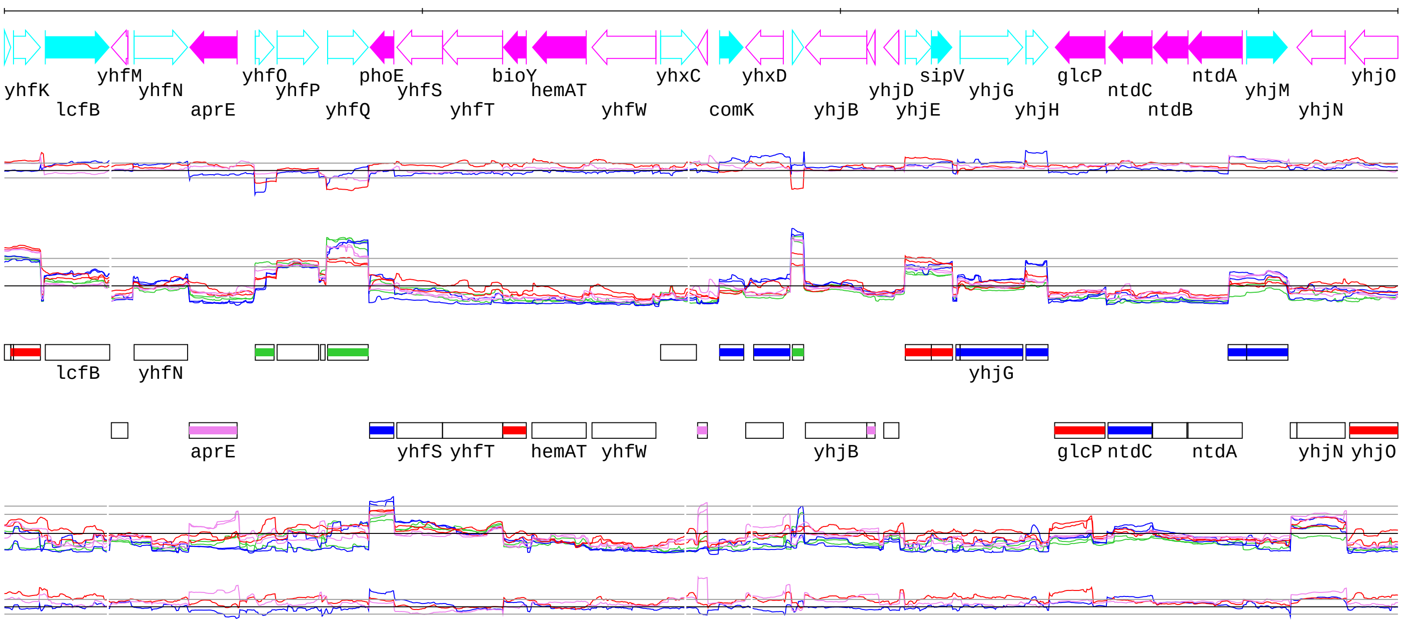

1 133 335 — 1 166 668

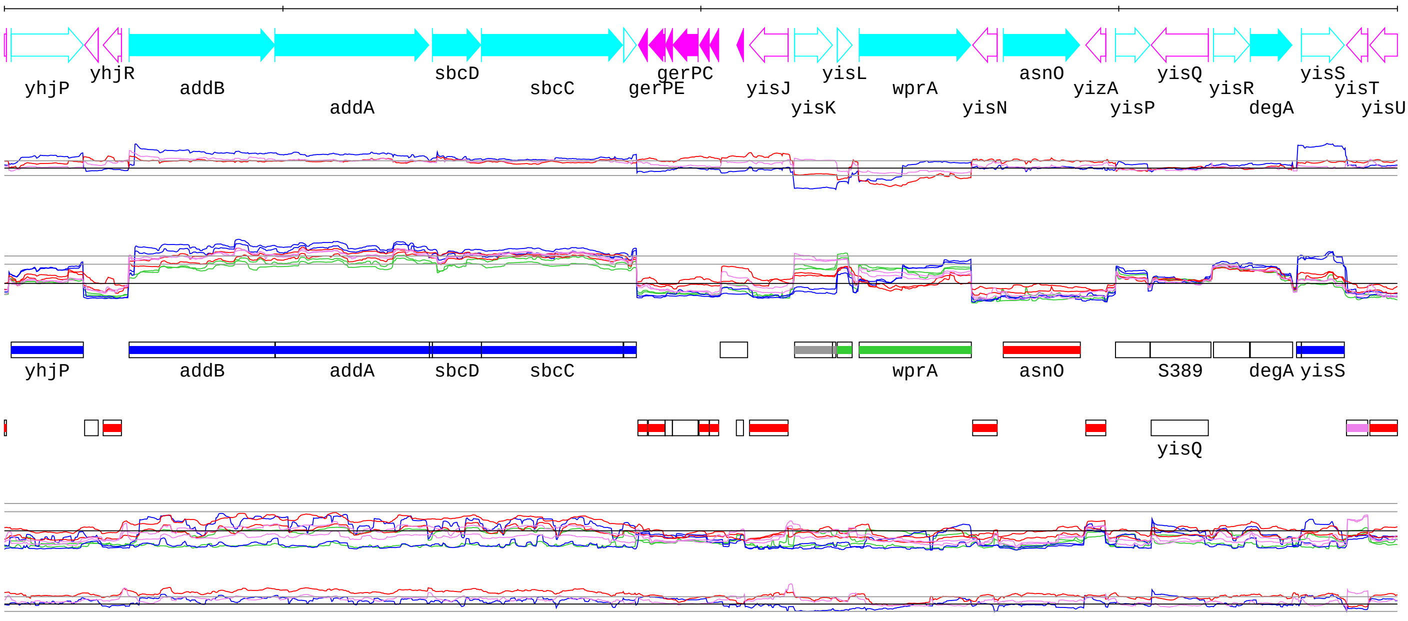

1 166 669 — 1 200 002

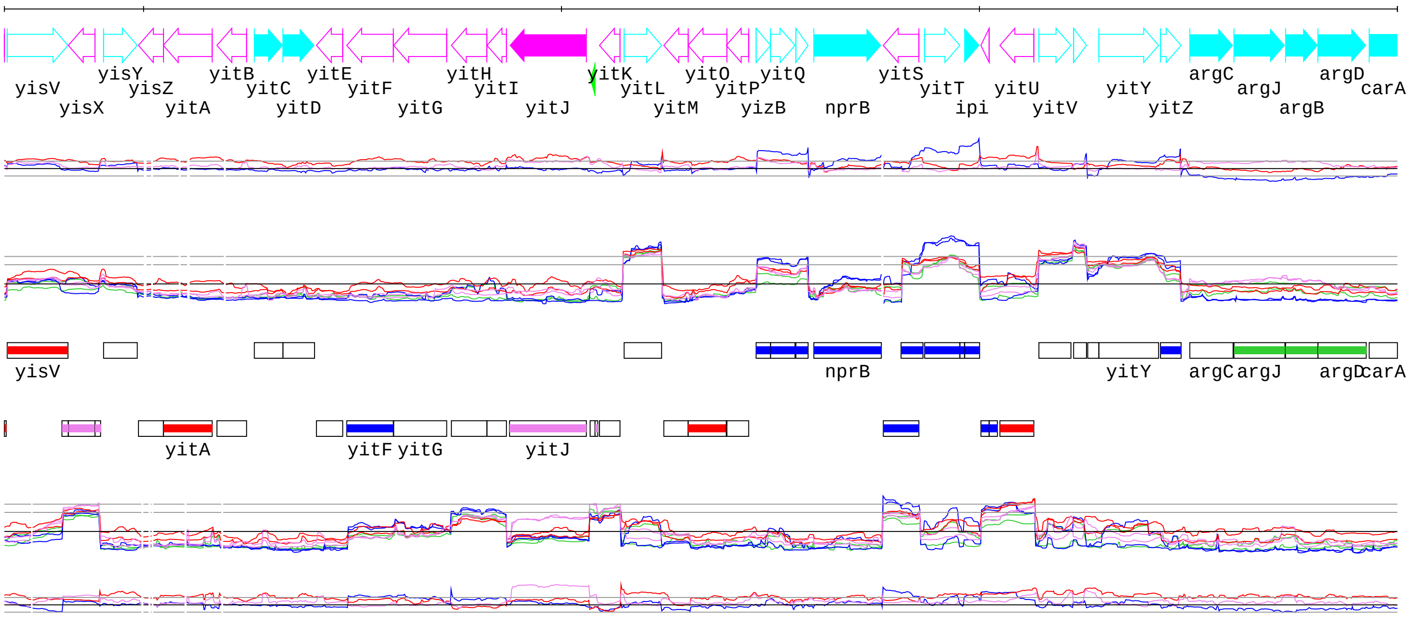

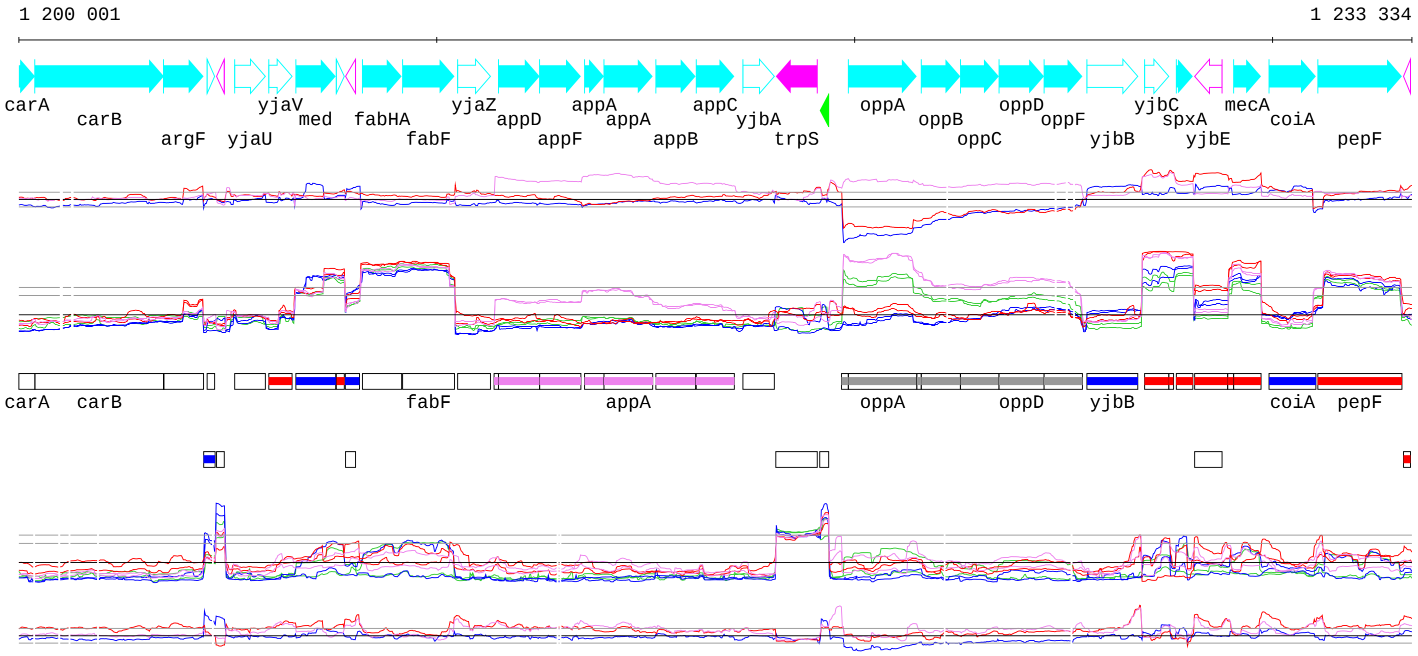
1 200 001
1 233 334
carA carB argF yjaU yjaV med fabHA fabF yjaZ appD appF appA appA appB appC yjbA trpS oppA oppB oppC oppD oppF yjbB yjbC spxA yjbE mecA coiA pepF
carA carB fabF appA oppA oppD yjbB coiA pepF

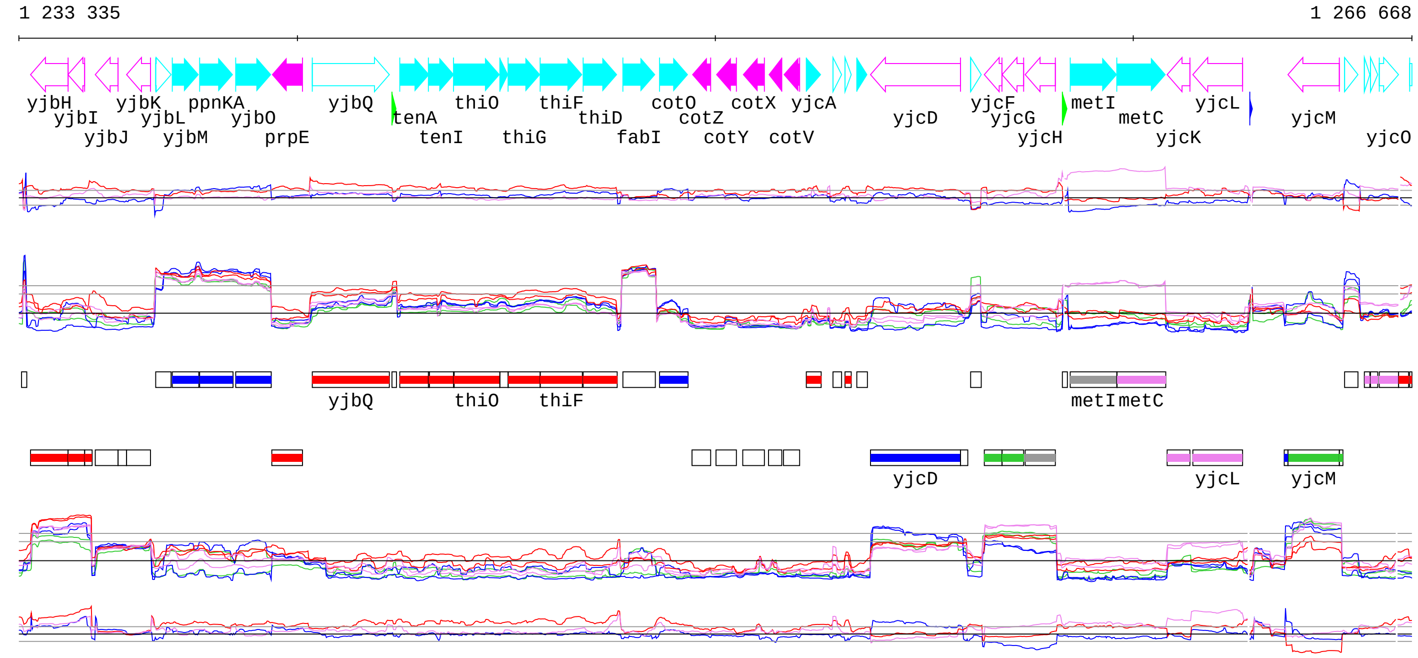
1 233 335
1 266 668
yjbH yjbI yjbJ yjbK yjbL yjbM ppnKA yjbO prpE yjbQ tenA tenI thiO thiG thiF thiD fabI cotO cotZ cotY cotX cotV yjcA yjcD yjcF yjcG yjcH metI metC yjcK yjcL yjcM yjcO
yjbQ thiO thiF metI metC
yjcD yjcL yjcM

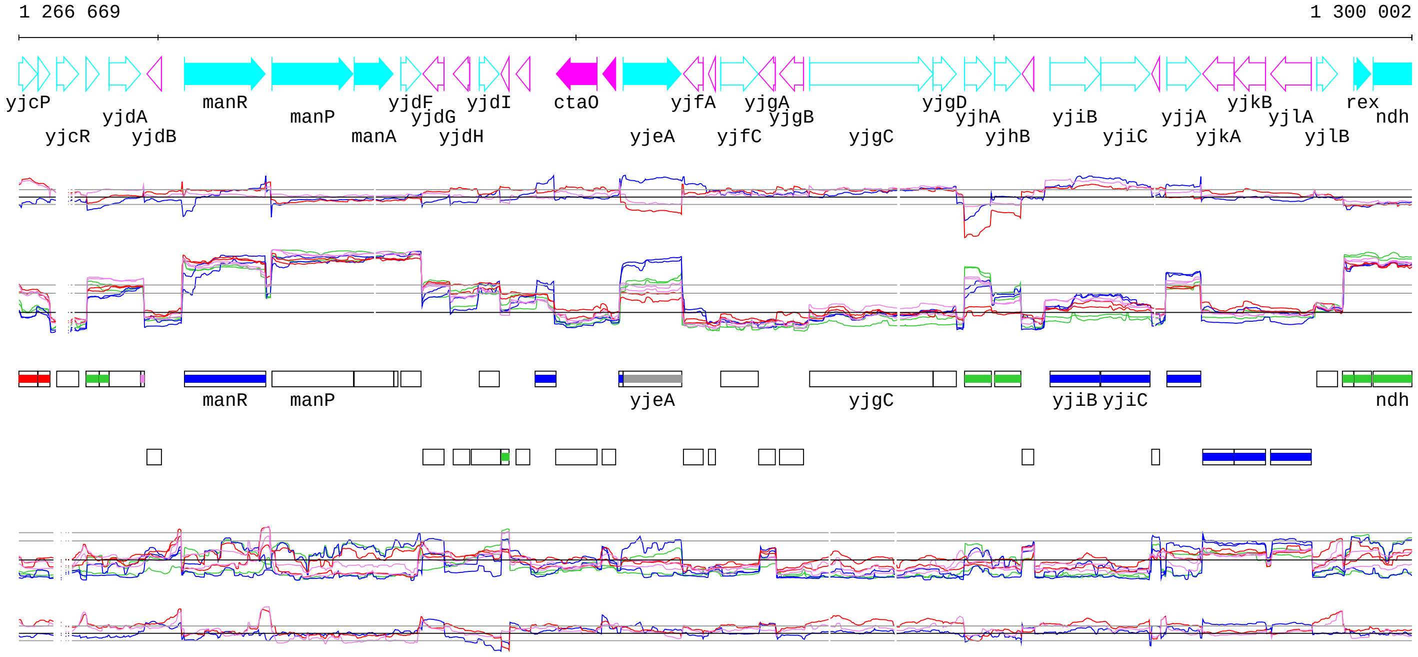
1 266 669
1 300 002
yjcP yjcR yjdA yjdB manR manP manA yjdF yjdG yjdH yjdI ctaO yjeA yjfA yjfC yjgA yjgB yjgC yjgD yjhA yjhB yjiB yjiC yjjA yjkA yjkB yjlA yjlB rex ndh
manR manP yjeA yjgC yjiB yjiC ndh

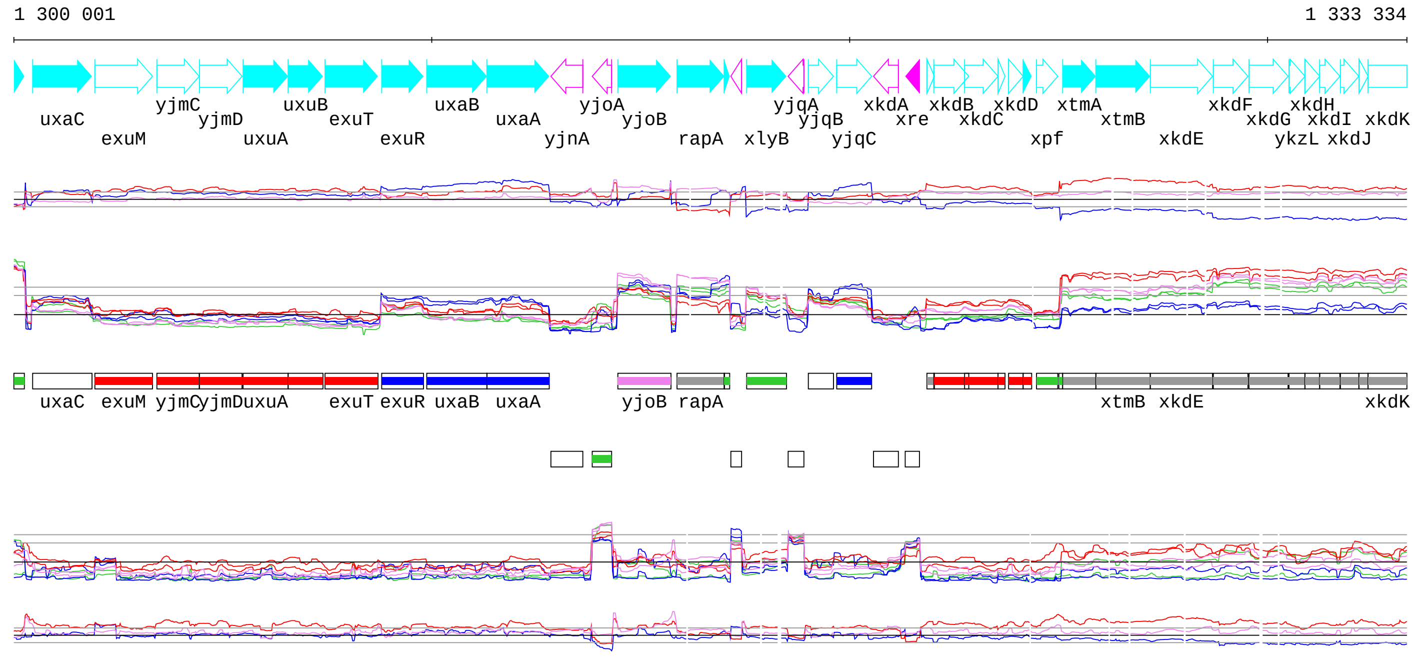
1 300 001
1 333 334
uxaC exuM yjmC yjmD uxuB uxuA exuT exuR uxaB uxaA yjoA yjnA yjoB rapA xlyB yjqA yjqB yjqC xkdA xre xkdB xkdC xkdD xtmA xpf xtmB xkdE xkdF xkdG xkdH xkdI xkdK ykzL xkdJ
uxaC exuM yjmCyjmDuxuA exuT exuR uxaB uxaA yjoB rapA xtmB xkdE xkdK

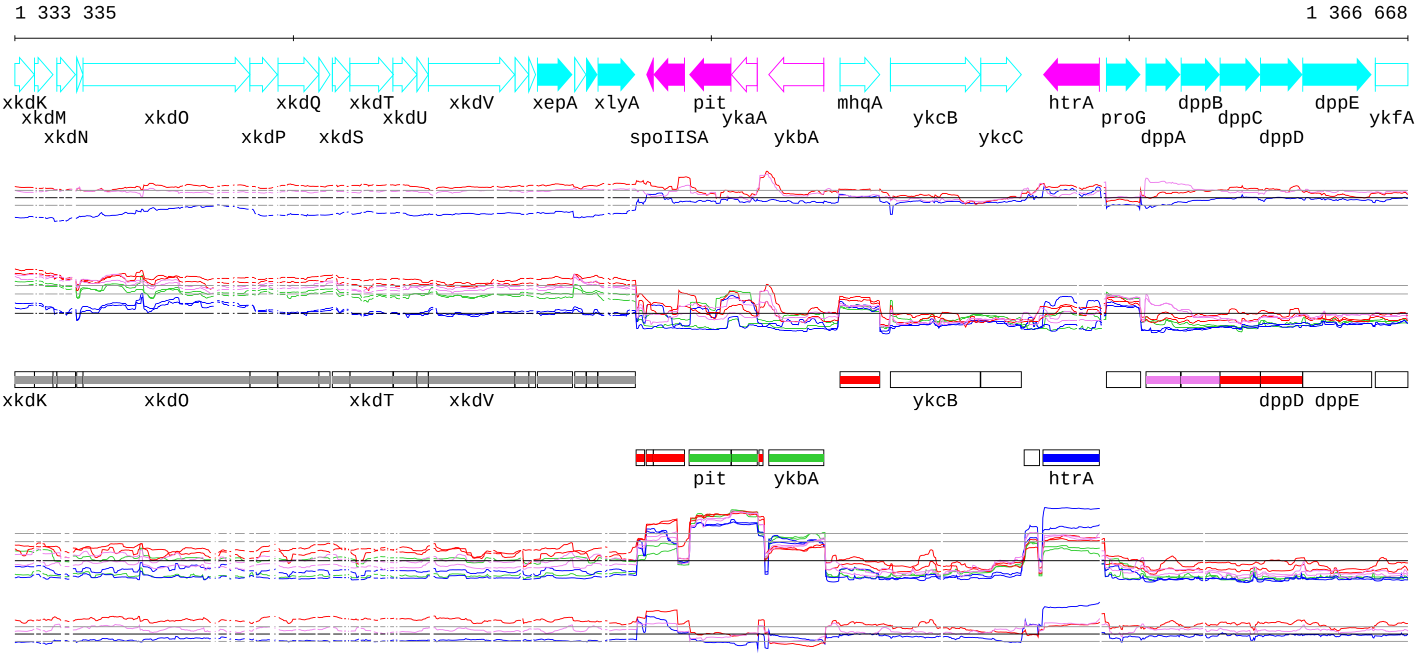
1 333 335
1 366 668
xkdK xkdM xkdN xkdO xkdP xkdQ xkdS xkdT xkdU xkdV xepA xlyA spoIISA pit ykaA ykbA mhqA ykcB ykcC htrA proG dppA dppB dppC dppD dppE ykfA
xkdK xkdO xkdT xkdV ykcB dppD dppE
pit ykbA htrA

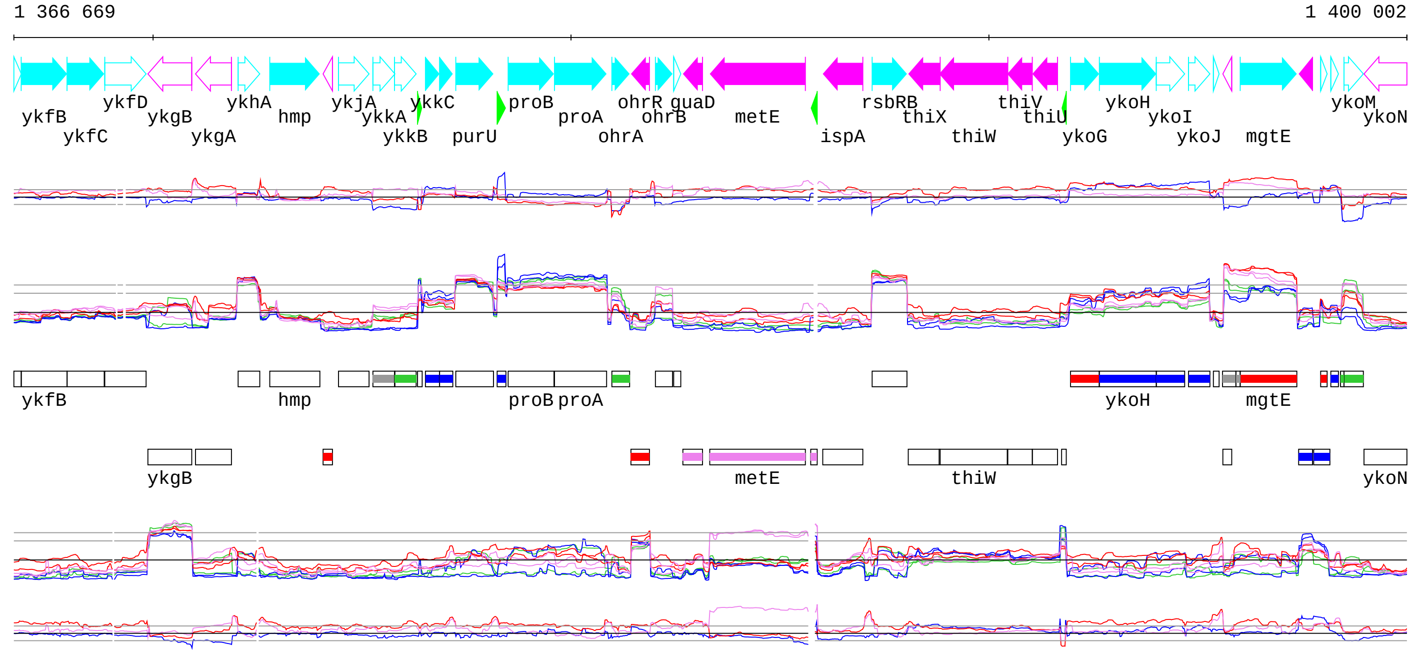
1 366 669
1 400 002
ykfB ykfC ykfD ykgB ykgA ykhA hmp ykjA ykkA ykkB ykkC purU proB proA ohrR ohrA ohrB guaD metE ispA rsbRB thiX thiW thiV thiU ykoG ykoH ykoI ykoJ mgtE ykoM ykoN
ykfB hmp proB proA ykoH mgtE
ykgB metE thiW ykoN

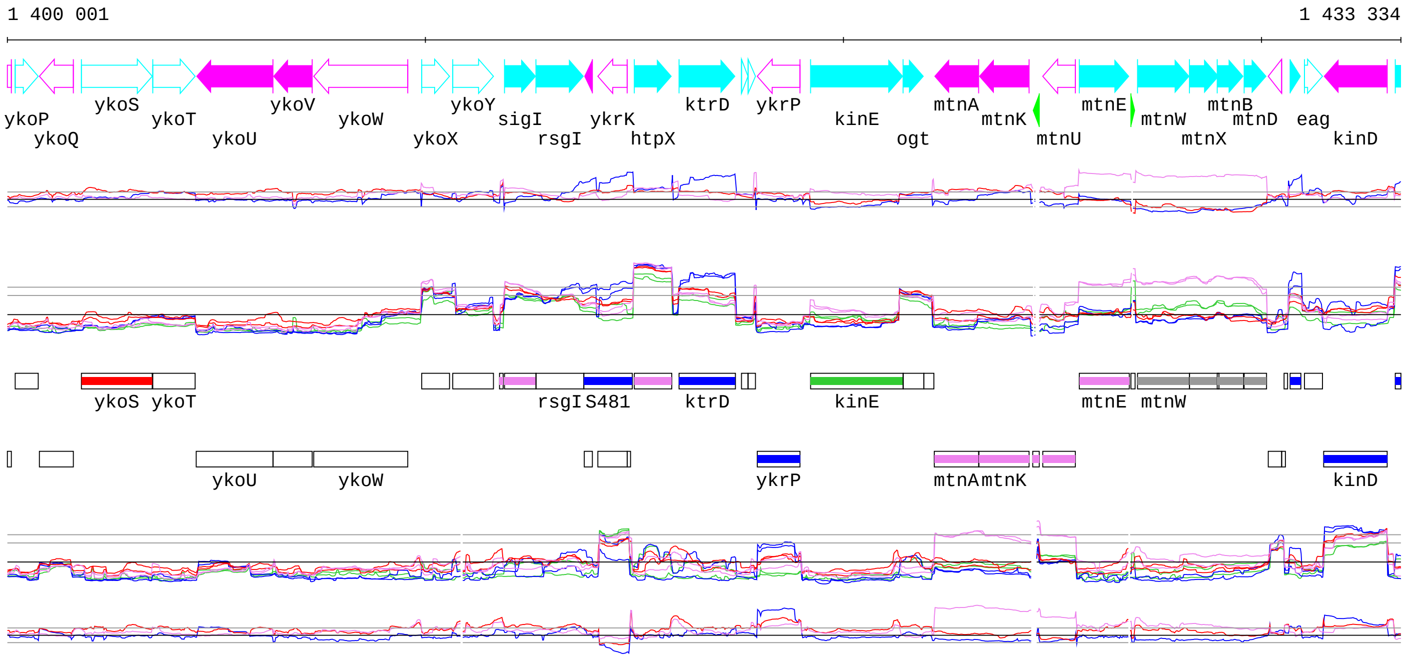
1 400 001
1 433 334
ykoP ykoS ykoT ykoV ykoW ykoY sigI ykrK ktrD ykrP kinE mtnA mtnK mtnE mtnW mtnB mtnD eag
ykoQ ykoU ykoX rsgI htpX ogt mtnU mtnX kinD
ykoS ykoT rsgI S481 ktrD kinE mtnE mtnW
ykoU ykoW ykrP mtnA mtnK kinD

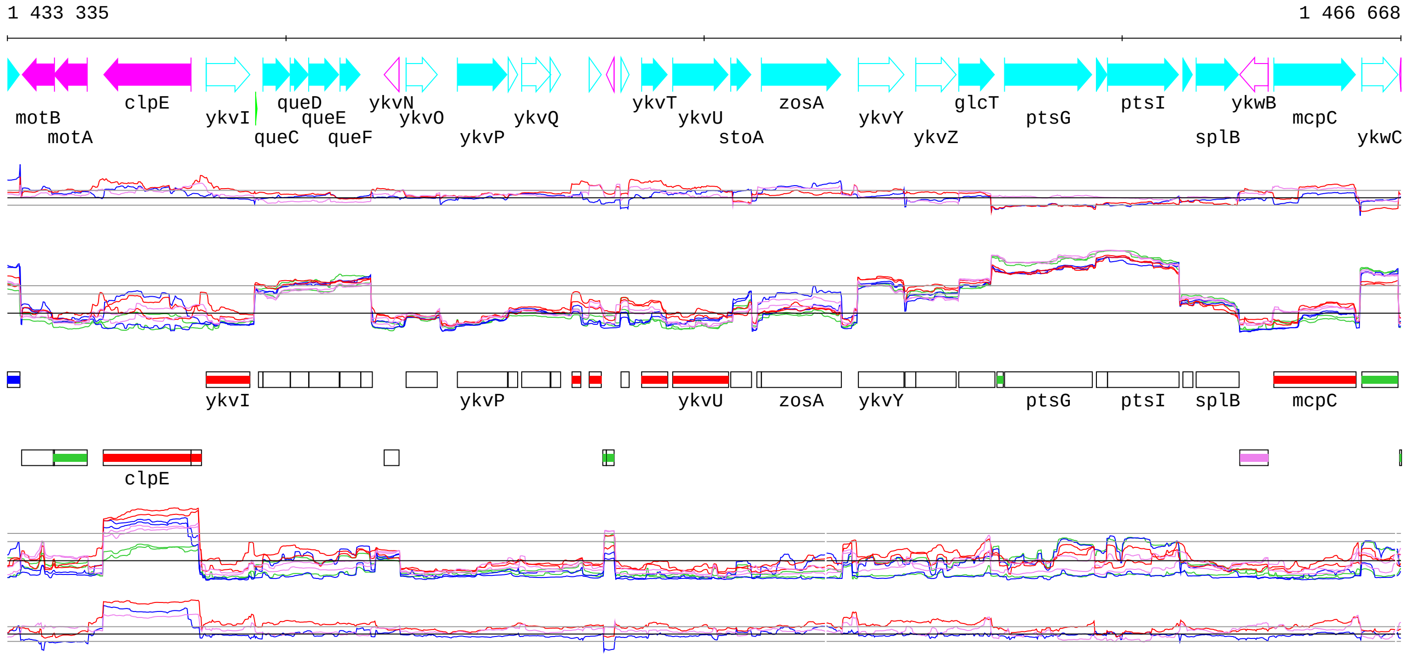
1 433 335
1 466 668
motB clpE ykvI queD queE ykvN ykvO ykvQ ykvT ykvU zosA ykvY glcT ptsG ptsI ykwB mcpC
motA queC queF ykvP stoA ykvZ splB ykwC
ykvI ykvP ykvU zosA ykvY ptsG ptsI splB mcpC
clpE

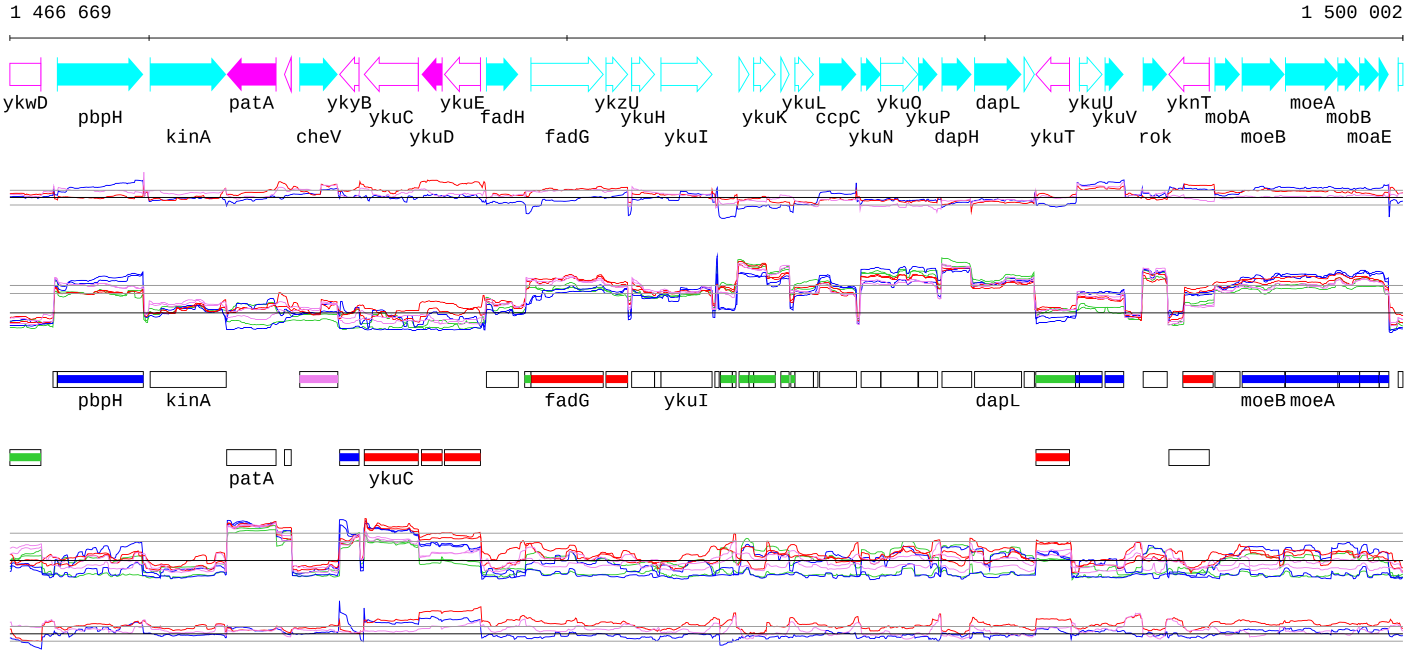
1 466 669
1 500 002
ykwD pbpH patA ykyB ykuC ykuE fadH ykzU ykuH ykuL ykuK ccpC ykuO ykuP dapL ykuU ykuV yknT mobA moeA mobB
kinA cheV ykuD fadG ykuI ykuN dapH ykuT rok moeB moaE
pbpH kinA fadG ykuI dapL moeB moeA
patA ykuC

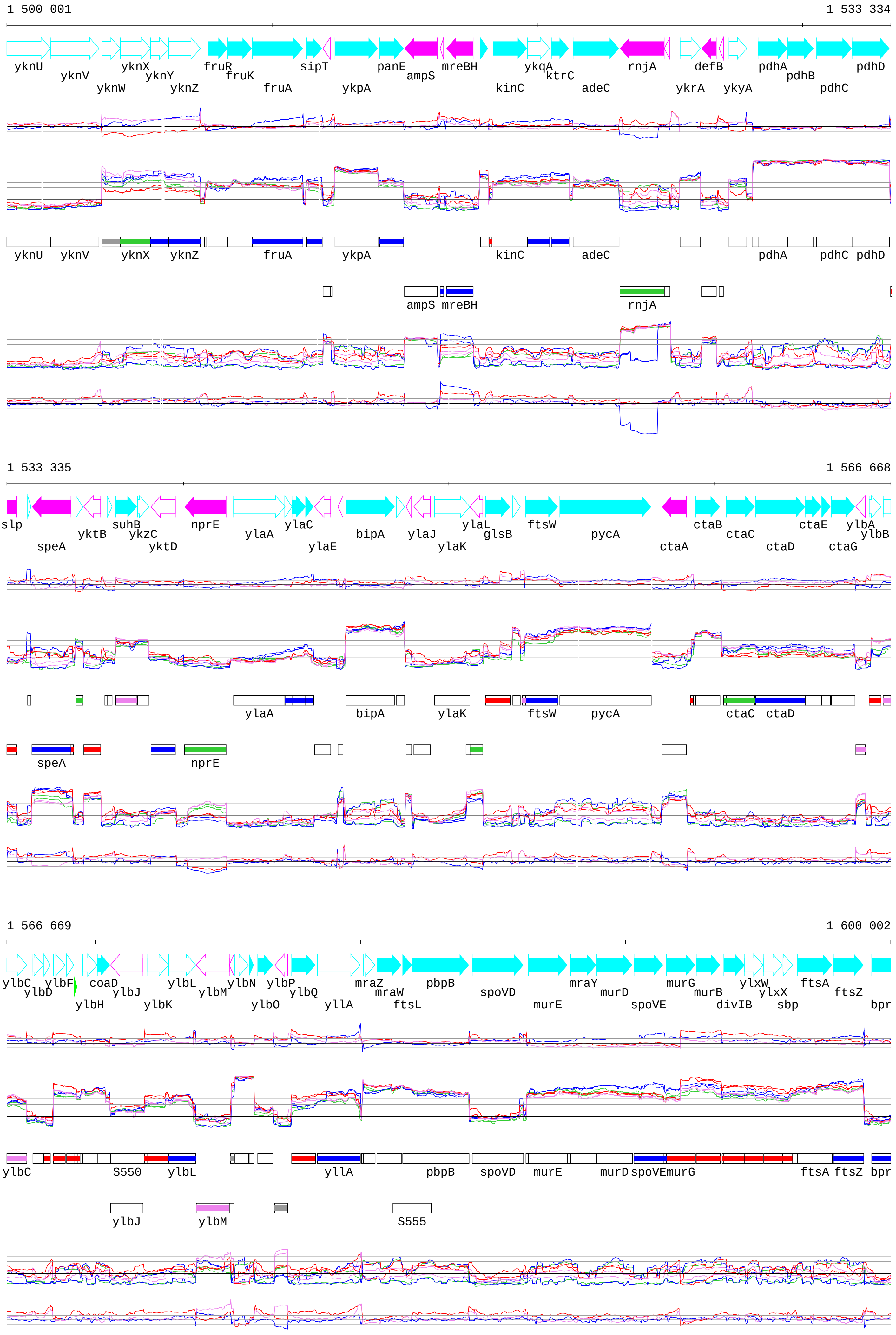
1 500 001
1 533 334
yknU yknV yknW yknX yknY yknZ fruR fruK fruA sipT ykpA panE ampS mreBH kinC ykqA ktrC adeC rnjA ykrA defB ykyA pdhA pdhB pdhC pdhD
yknU yknV yknX yknZ fruA ykpA kinC adeC pdhA pdhC pdhD
ampS mreBH rnjA
1 533 335
1 566 668
slp speA yktB suhB ykzC yktD nprE ylaA ylaC ylaE bipA ylaJ ylaK ylaL glsB ftsW pycA ctaA ctaB ctaC ctaD ctaE ctaG ylbA ylbB
ylaA bipA ylaK ftsW pycA ctaC ctaD
speA nprE
1 566 669
1 600 002
ylbC ylbD ylbF ylbH coaD ylbJ ylbK ylbL ylbM ylbN ylbO ylbP ylbQ yllA mraZ mraW ftsL pbpB spoVD murE mraY murD spoVE murG murB divIB ylxW ylxX sbp ftsA ftsZ bpr
ylbC S550 ylbL yllA pbpB spoVD murE murD spoVE murG ftsA ftsZ bpr
ylbJ ylbM S555

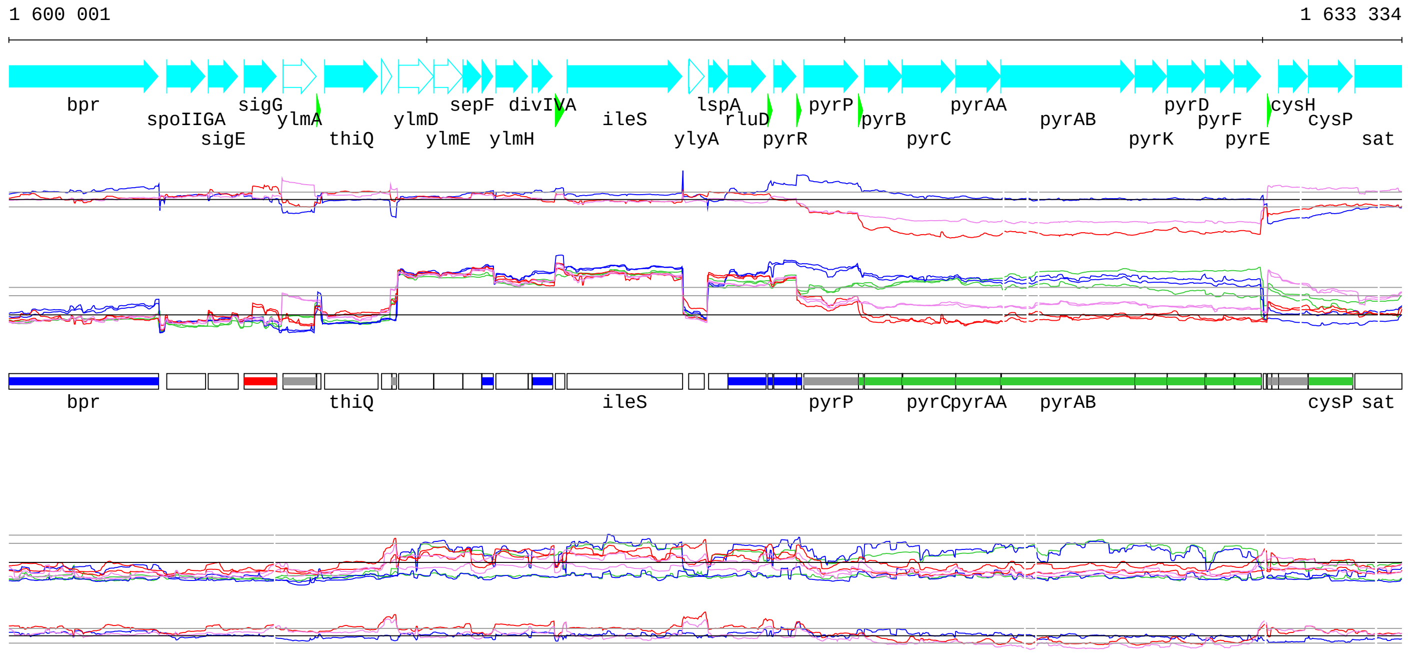
1 600 001
1 633 334
bpr
sigG
spoIIGA
ylmA
sigE
thiQ
ylmD
ylmE
sepF
ylmH
divIVA
ileS
lspA
ylyA
rluD
pyrR
pyrP
pyrB
pyrC
pyrAA
pyrAB
pyrD
pyrK
pyrF
pyrE
cysH
cysP
sat
bpr
thiQ
ileS
pyrP
pyrCpyrAA
pyrAB
cysP sat

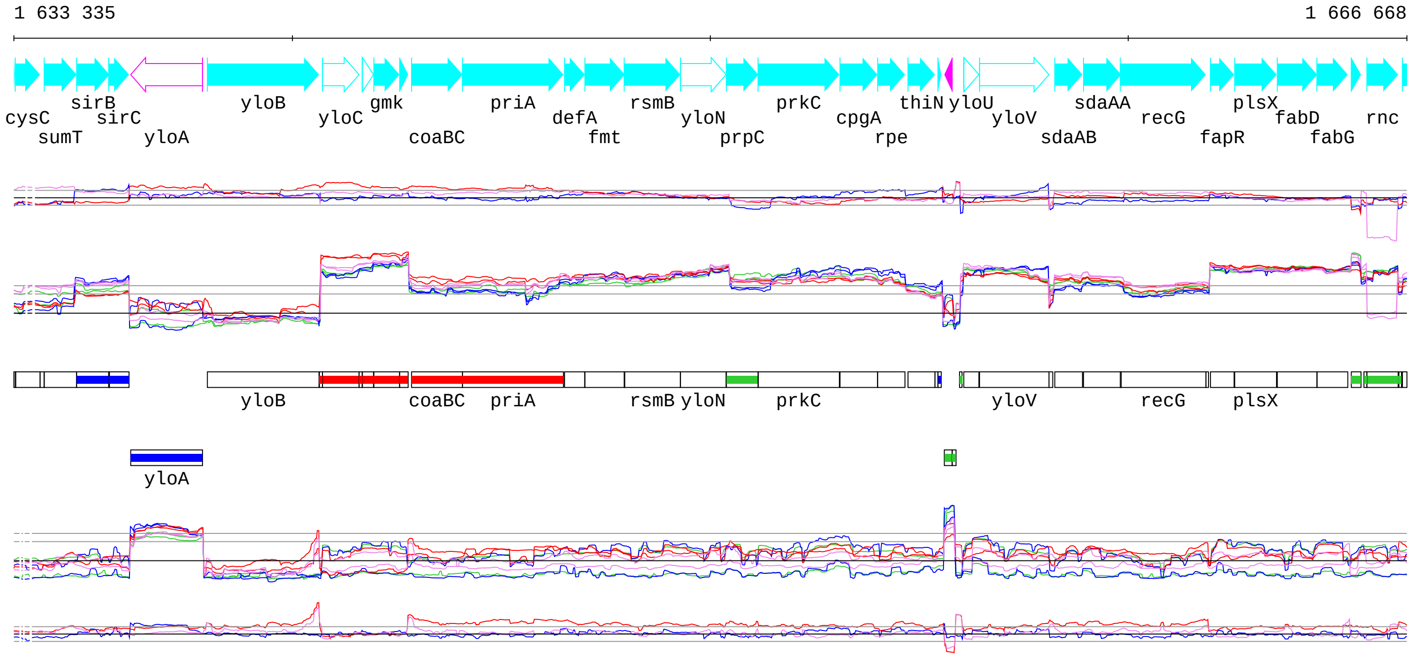
1 633 335
1 666 668
cysC
sirB
sumT
sirC
yloA
yloB
yloC
gmk
coaBC
priA
defA
fmt
rsmB
ylоN
prpC
prkC
cpgA
rpe
thiN
yloU
ylоV
sdaAA
sdaAB
recG
fapR
plsX
fabD
fabG
rnc
yloB
coaBC
priA
rsmB ylоN
prkC
ylоV
recG
plsX
yloA

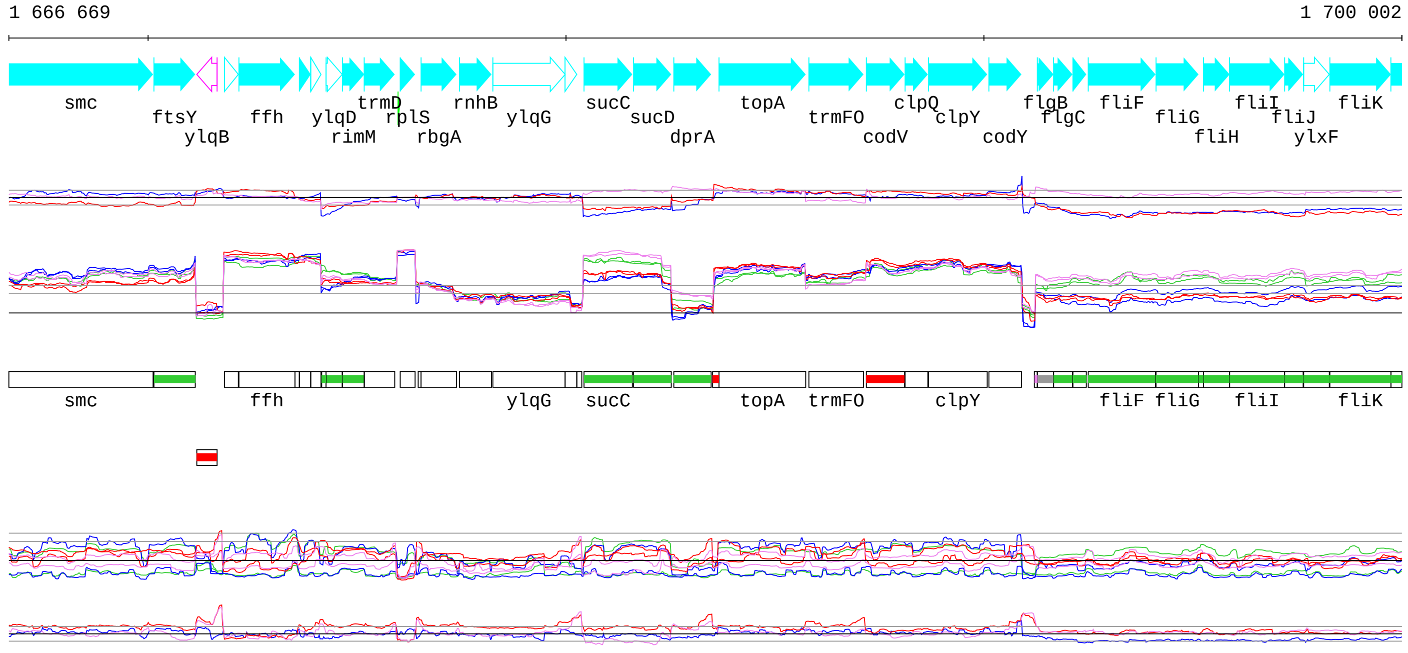
1 666 669
1 700 002
smc
ftsY
ylqB
ffh
ylqD
trmD
rimM
rplS
rnhB
rbgA
ylqG
sucC
sucD
dprA
topA
trmFO
codV
clpQ
clpY
codY
flgB
flgC
fliF
fliG
fliH
fliI
fliJ
ylxF
fliK
smc
ffh
ylqG
sucC
topA
trmFO
clpY
fliF fliG
fliI
fliK

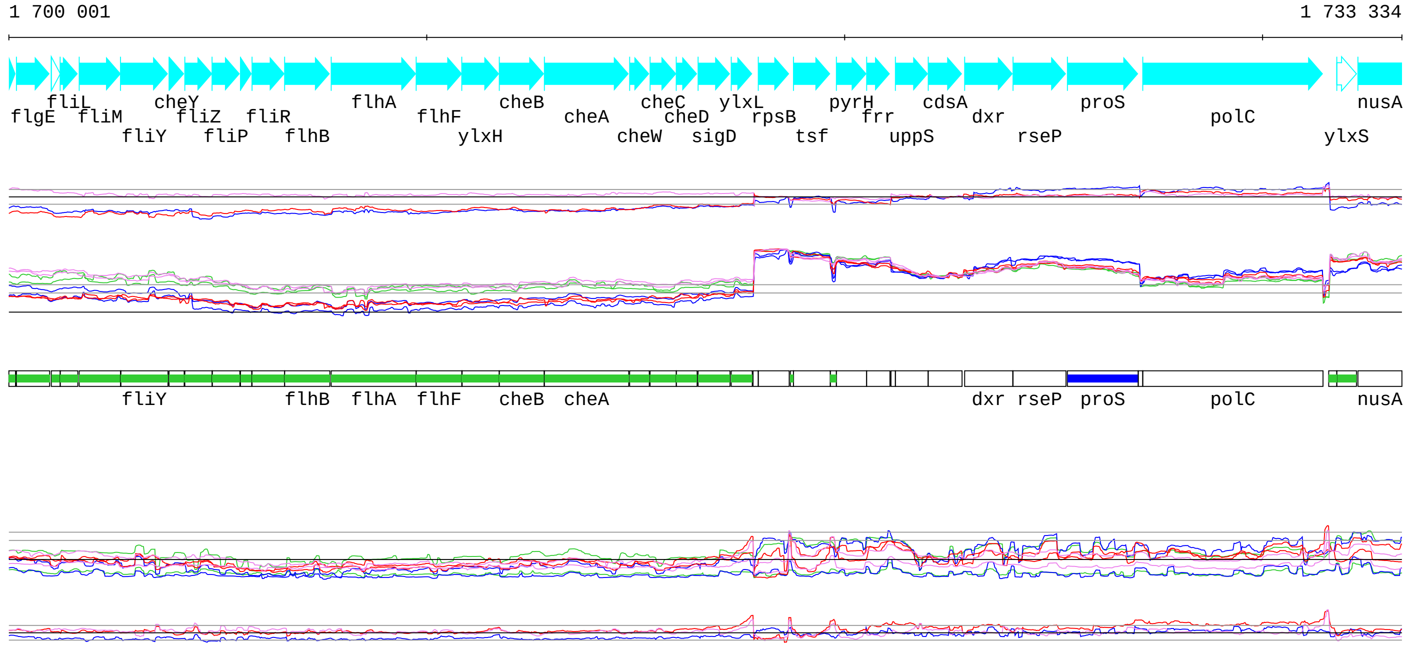
1 700 001
1 733 334
fliL flgE fliM cheY fliZ fliR fliY fliP flhB flhA flhF ylxH cheB cheA cheC cheD cheW sigD ylxL rpsB tsf pyrH frr uppS cdsA dxr rseP proS polC nusA ylxS
fliY flhB flhA flhF cheB cheA dxr rseP proS polC nusA

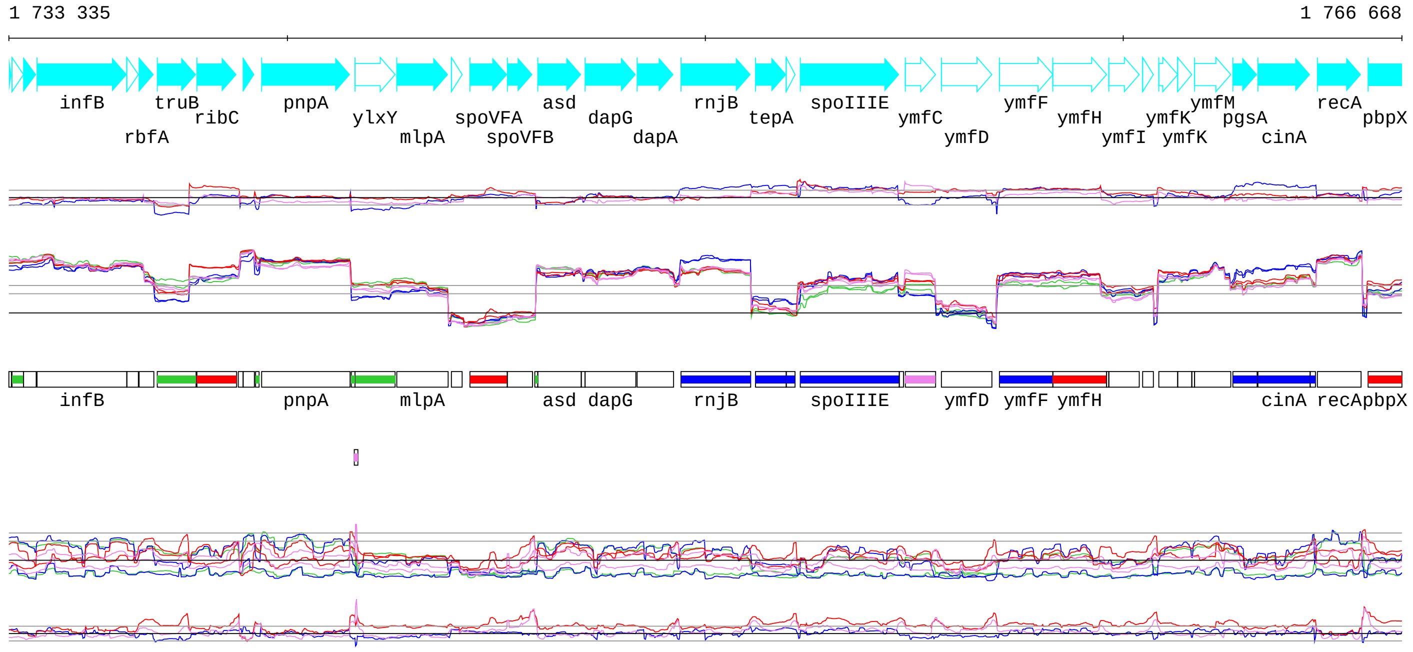
1 733 335
1 766 668
infB truB ribC rbfA pnpA ylxY mlpA spoVFA spoVFB asd dapG dapA rnjB tepA spoIIIE ymfC ymfD ymfF ymfH ymfI ymfK ymfM pgsA cinA recA pbpX
infB pnpA mlpA asd dapG rnjB spoIIIE ymfD ymfF ymfH cinA recApbpX

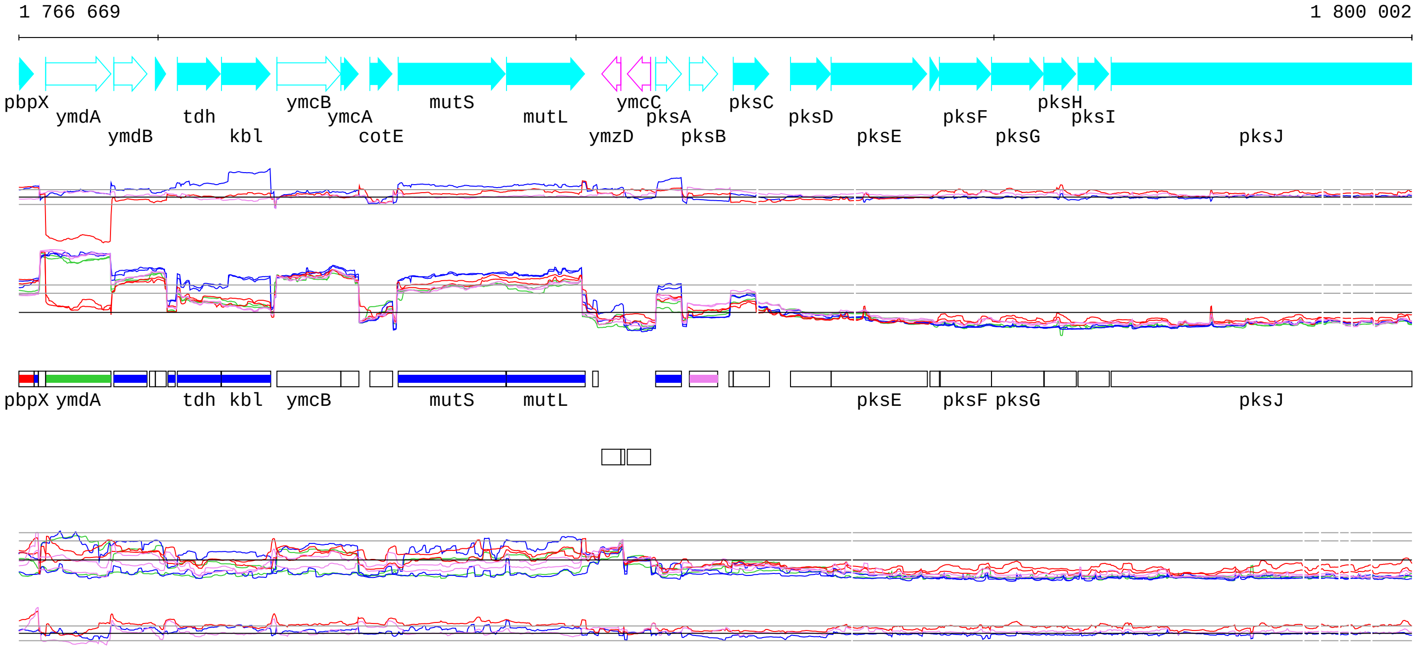
1 766 669
1 800 002
pbpX ymdA ymdB tdh kbl ymcB ymcA cotE mutS mutL ymcC ymzD pksA pksB pksC pksD pksE pksF pksG pksH pksI pksJ
pbpX ymdA tdh kbl ymcB mutS mutL pksE pksF pksG pksJ

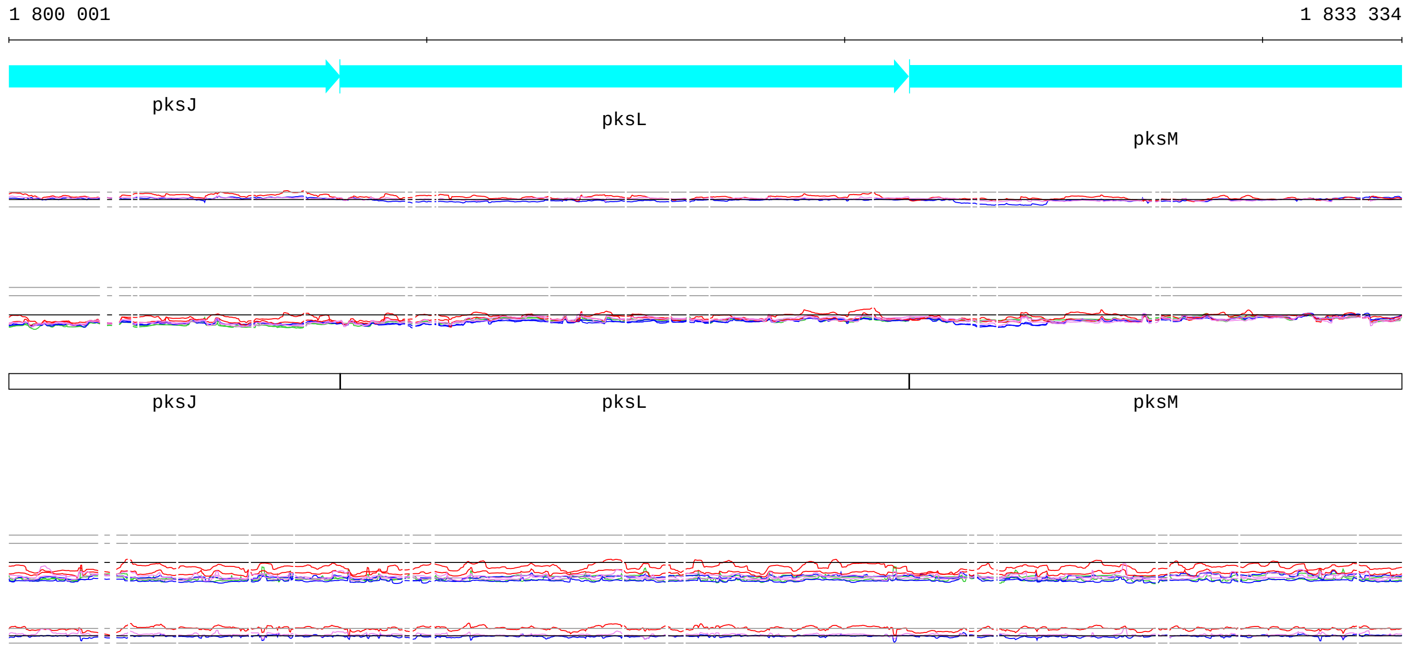
1 800 001
1 833 334
pksJ
pksL
pksM
pksJ
pksL
pksM

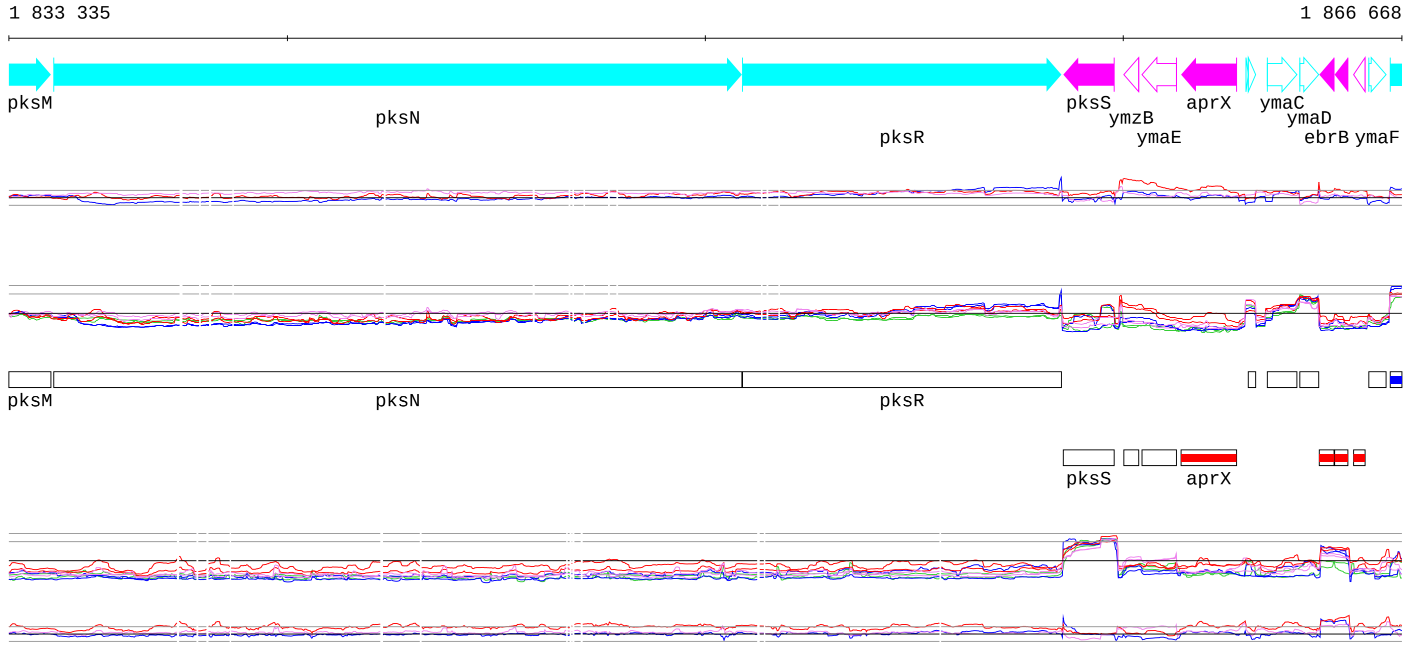
1 833 335
1 866 668
pksM
pksN
pksR
pksS
ymzB
ymaE
aprX
ymaC
ymaD
ebrB
ymaF
pksM
pksN
pksR
pksS
aprX

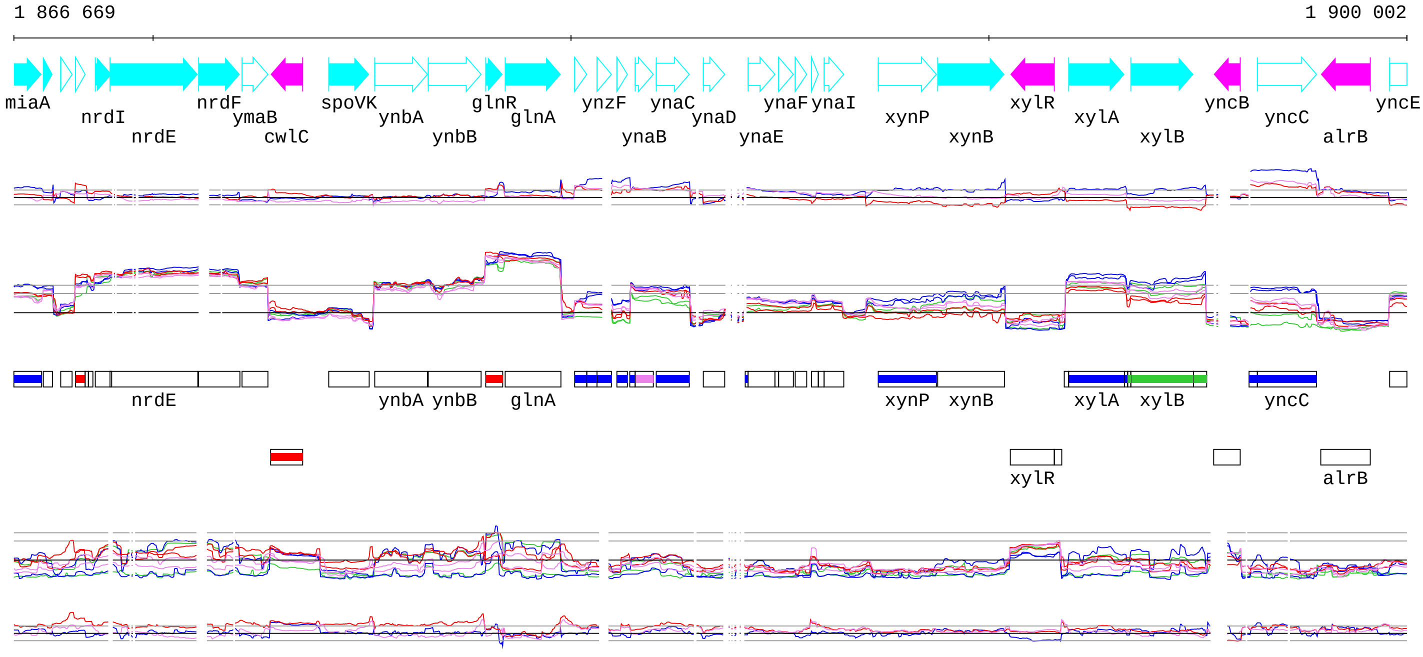
1 866 669
1 900 002
miaA
nrdI
nrdE
nrdF
ymaB
cwlC
spoVK
ynbA
ynbB
glnR
glnA
ynzF
ynaB
ynaC
ynaD
ynaE
ynaF
ynaI
xynP
xynB
xylR
xylA
xylB
yncB
yncC
alrB
yncE
nrdE
ynbA
ynbB
glnA
xynP
xynB
xylA
xylB
yncC
xylR
alrB

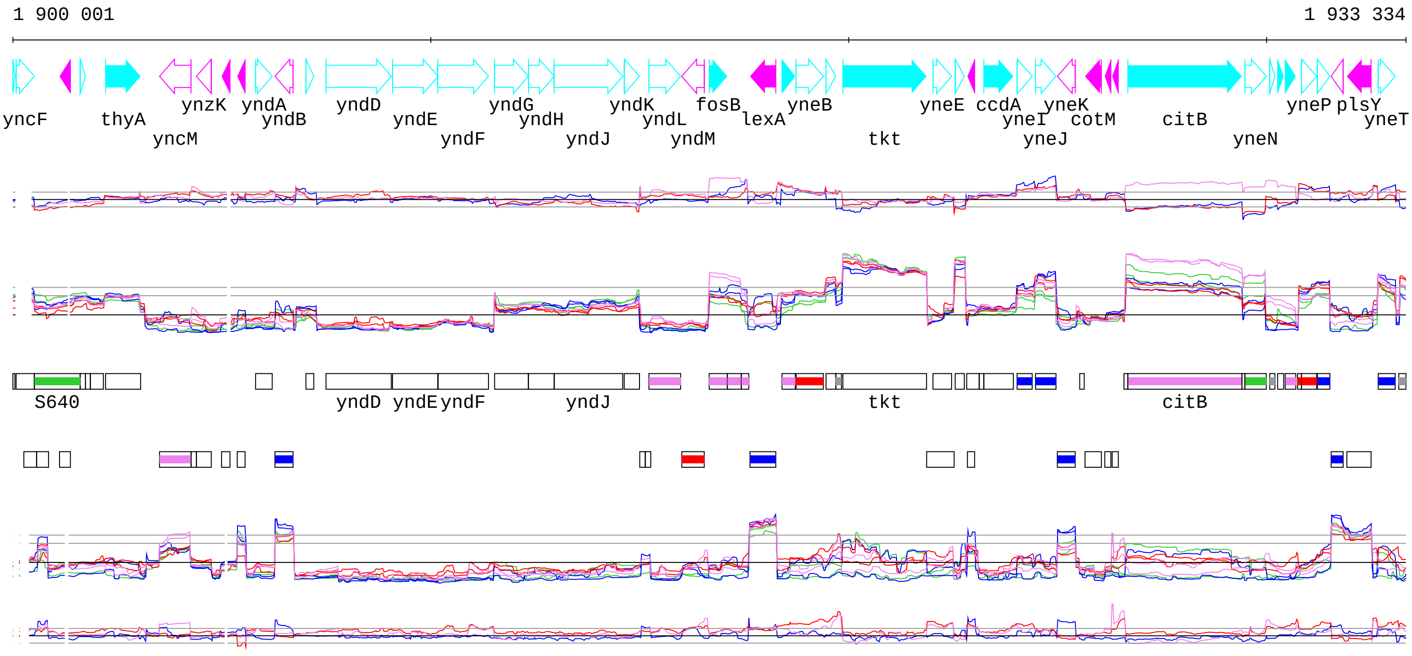
1 900 001
1 933 334
yncF thyA ynzK yndA yndB yncM yndD yndE yndF yndG yndH yndJ yndK yndL yndM fosB lexA yneB tkt yneE ccdA yneI yneJ yneK cotM citB yneN yneP plsY yneT
S640 yndD yndE yndF yndJ tkt citB

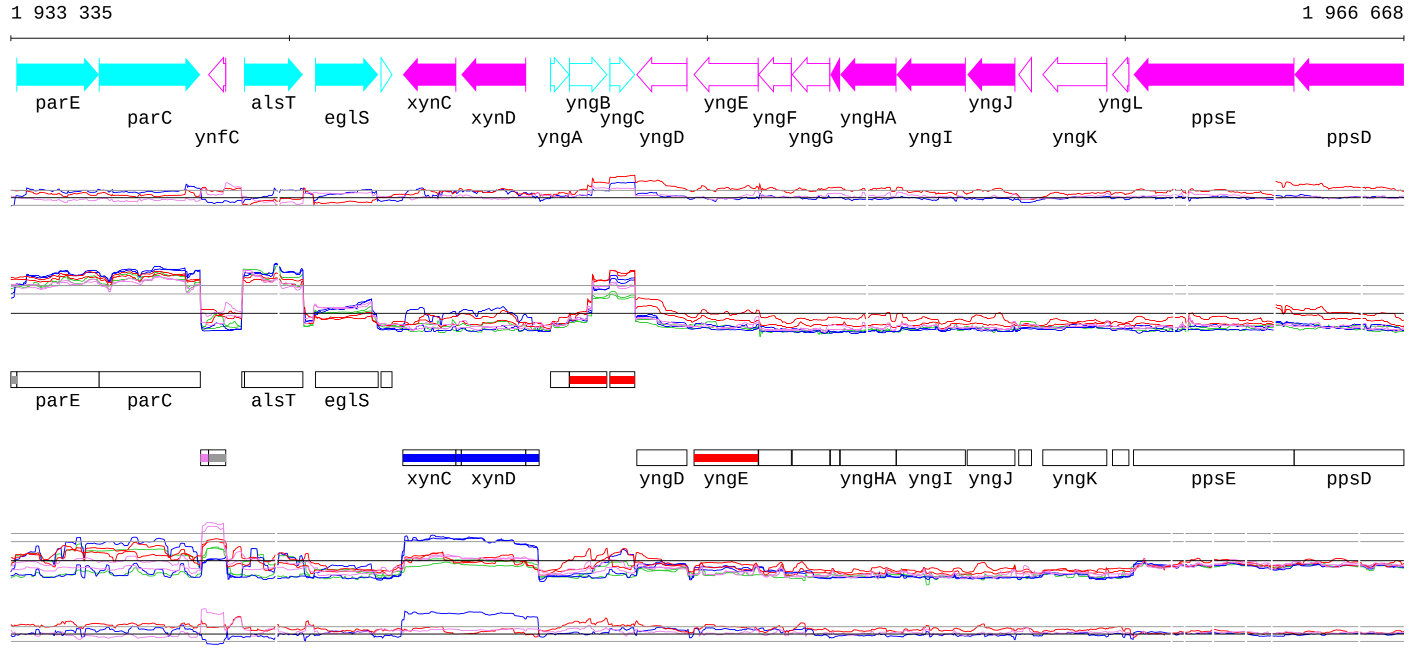
1 933 335
1 966 668
parE parC ynfC alsT eglS xynC xynD yngA yngB yngC yngD yngE yngF yngG yngHA yngI yngJ yngK yngL ppsE ppsD
parE parC alsT eglS
xynC xynD yngD yngE yngHA yngI yngJ yngK ppsE ppsD

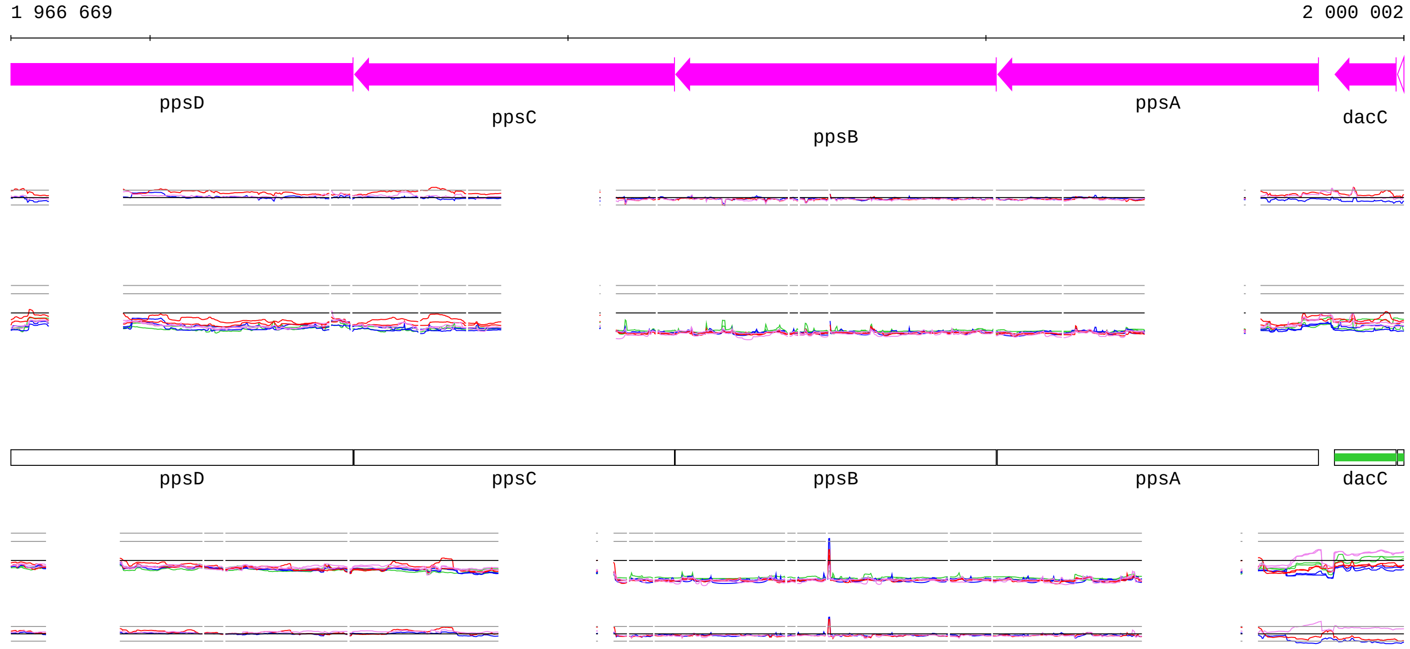
1 966 669
2 000 002
ppsD ppsC ppsB ppsA dacC
ppsD ppsC ppsB ppsA dacC

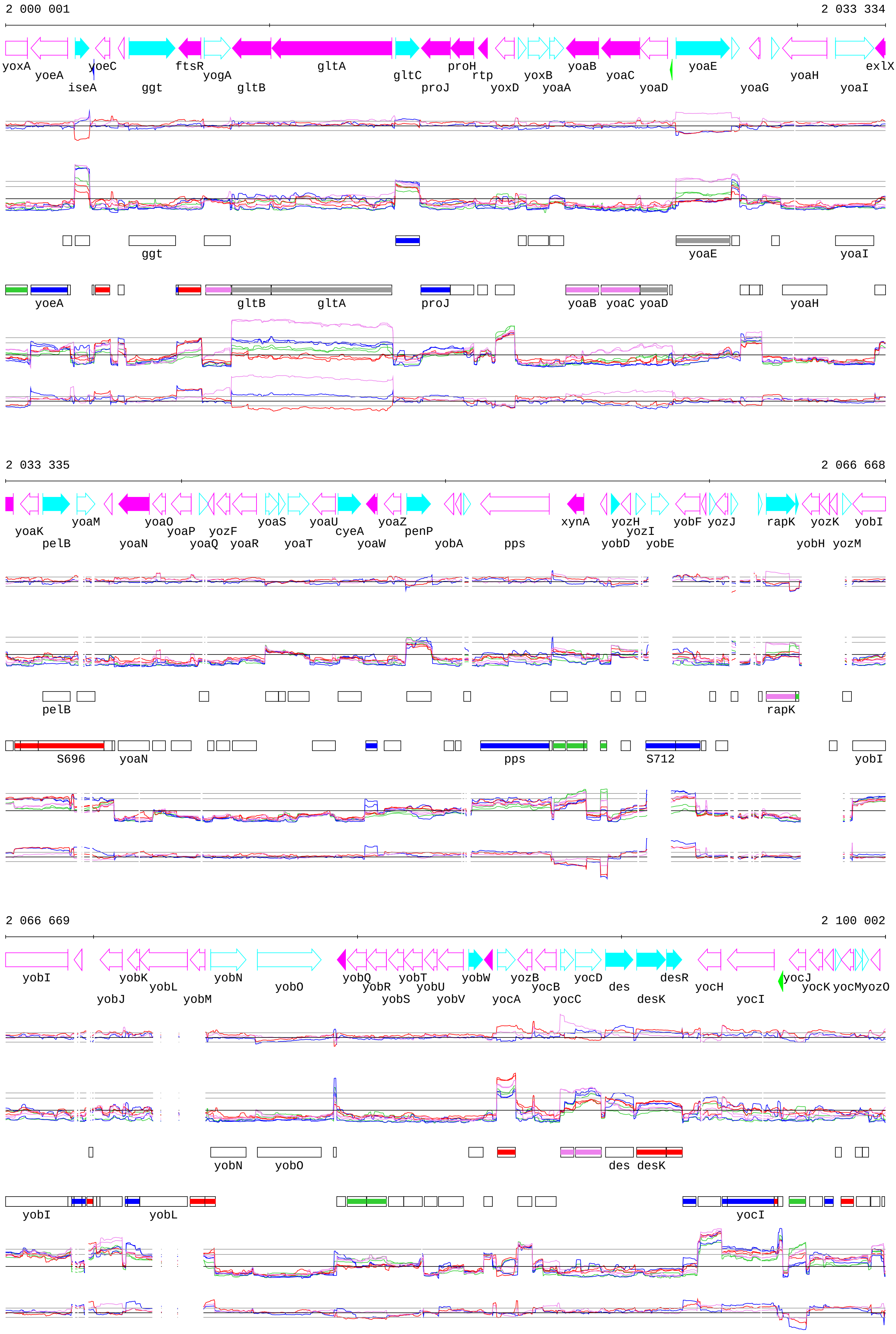

2 000 001
2 033 334
yoxA yoeA yoeC iseA ggt ftsR yogA gltB gltA gltC proJ proH rtp yoxD yoxB yoaA yoaB yoaC yoaD yoaE yoaG yoaH exlX yoaI
ggt
yoaE
yoaI
yoeA
gltB gltA
proJ
yoaB yoaC yoaD
yoaH
2 033 335
2 066 668
yoaK pelB yoaM yoaN yoaO yoaP yoaQ yozF yoaR yoaS yoaT yoaU cyeA yoaW yoaZ penP yobA pps xynA yozH yobD yozI yobE yobF yozJ rapK yobH yozK yozM yobI
pelB
rapK
S696 yoaN
pps
S712
yobI
2 066 669
2 100 002
yobI yobJ yobK yobL yobM yobN yobO yobQ yobR yobS yobT yobU yobV yobW yocA yozB yocB yocC yocD des desK desR yocH yocI yocJ yocK yocM yozO
yobN yobO
des desK
yobI
yobL
yocI

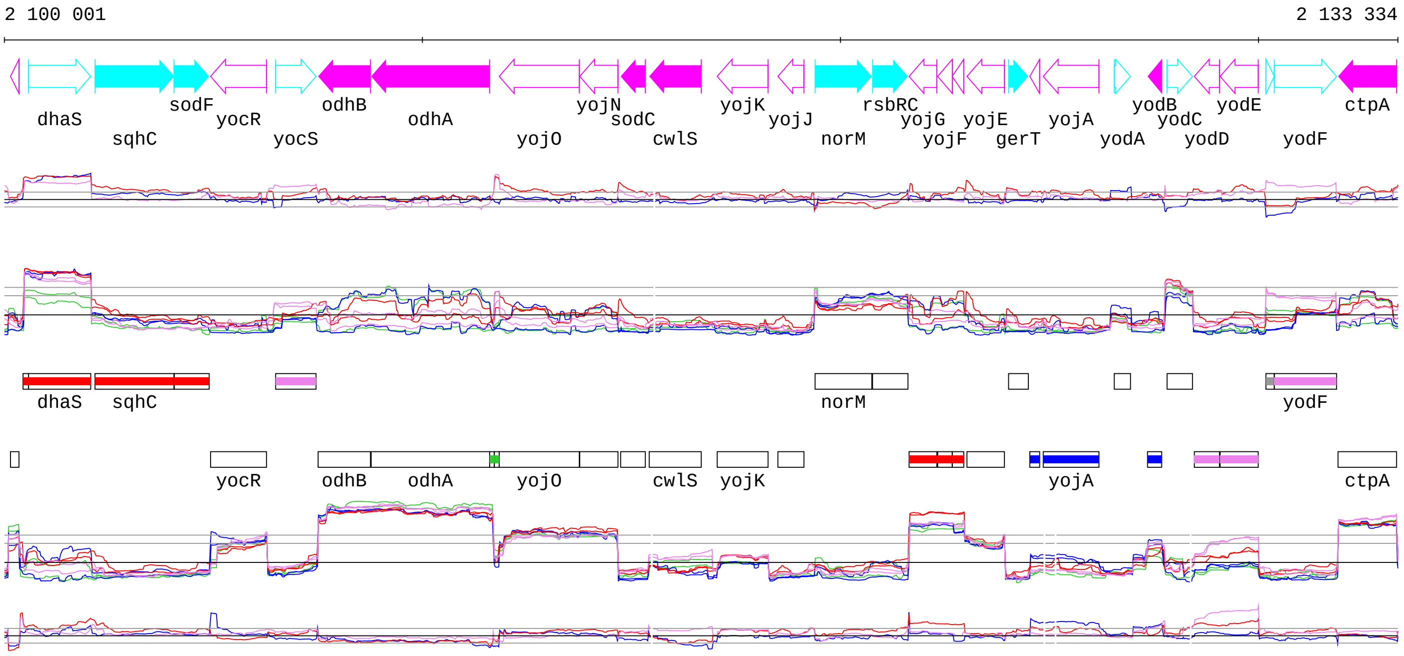
2 100 001
2 133 334
dhaS sqhC sodF yocR yocS odhB odhA yojN yojO sodC cwlS yojK yojJ norM rsbRC yojG yojF yojE gerT yojA yodA yodB yodC yodE yodD yodF ctpA
dhaS sqhC
norM yodF
yocR odhB odhA yojO cwlS yojK yojA ctpA

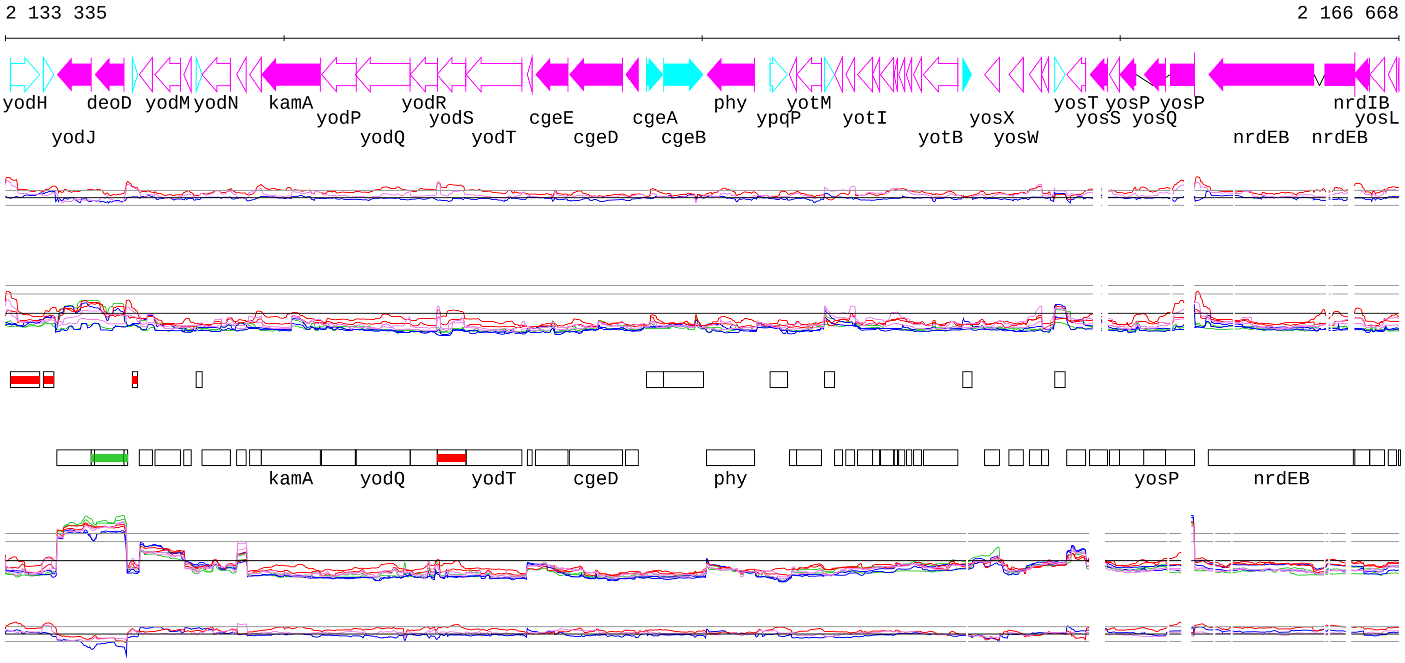
2 133 335
2 166 668
yodH yodJ deoD yodM yodN kamA yodP yodQ yodR yodS yodT cgeE cgeD cgeA cgeB phy ypqP yotM yotI yotB yosX yosW yosT yosS yosP yosQ yosP nrdEB nrdIB nrdEB yosL
kamA yodQ yodT cgeD phy yosP nrdEB

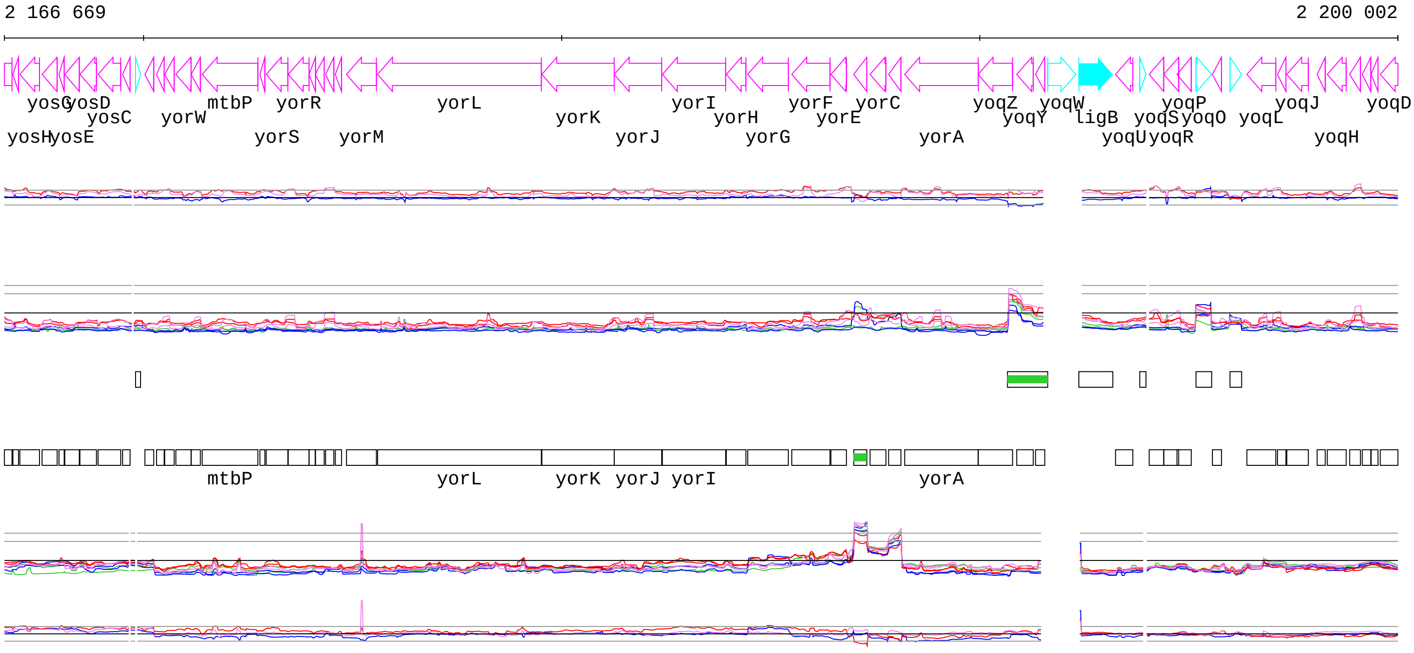
2 166 669
2 200 002
yosG yosD yosH yosC yosE yorW mtbP yorR yorS yorM yorL yorK yorJ yorI yorH yorG yorF yorE yorC yorA yoqZ yoqY yoqW ligB yoqU yoqS yoqR yoqP yoqO yoqL yoqJ yoqH yoqD
mtbP yorL yorK yorJ yorI yorA

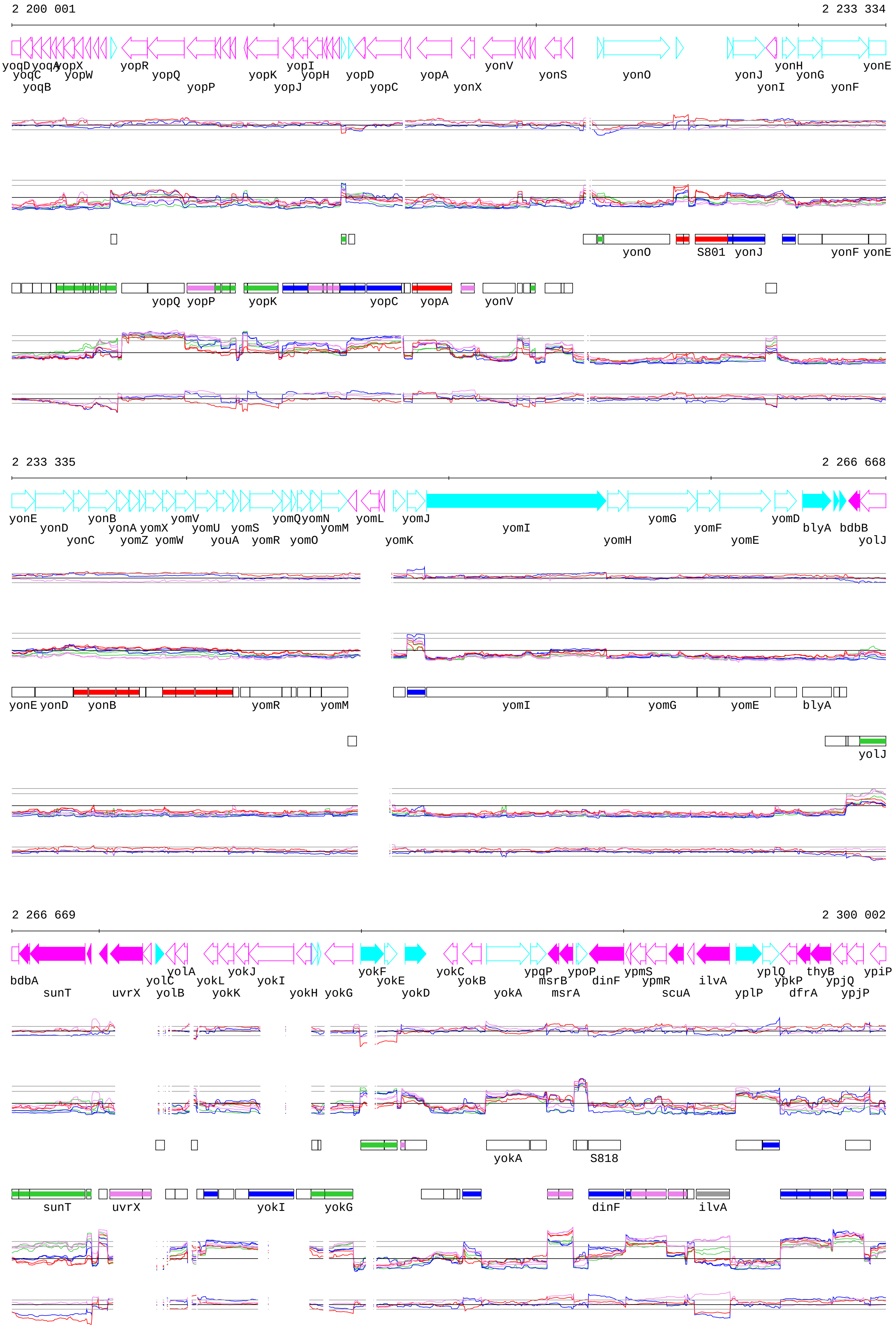
2 200 001
2 233 334
yoqD yoqA yopX
yoqC yopW
yoqB
yopR
yopQ
yopP
yopK
yopI
yopH
yopJ
yopD
yopC
yopA
yonX
yonV
yonS
yonO
yonJ
yonI
yonH
yonG
yonF
yonE
yonO
S801 yonJ
yonF yonE
yopQ yopP
yopK
yopC
yopA
yonV
2 233 335
2 266 668
yonE
yonD
yonC
yonB
yonA yomX
yomZ yomW
yomV
yomU
youA
yomS
yomR
yomQ yomN
yomO
yomM
yomL
yomK
yomJ
yomI
yomH
yomG
yomF
yomE
yomD
blyA
bdbB
yolJ
yonE yonD
yonB
yomR
yomM
yomI
yomG
yomE
blyA
yolJ
2 266 669
2 300 002
bdbA
sunT
uvrX
yolC
yolA
yolB
yokL
yokK
yokJ
yokI
yokH yokG
yokF
yokE
yokD
yokC
yokB
yokA
ypqP
msrB
msrA
ypoP
dinF
ypmS
ypmR
scuA
ilvA
yplQ
yplP
ypkP
thyB
dfrA
ypjQ
ypjP
ypiP
yokA
S818
sunT
uvrX
yokI
yokG
dinF
ilvA

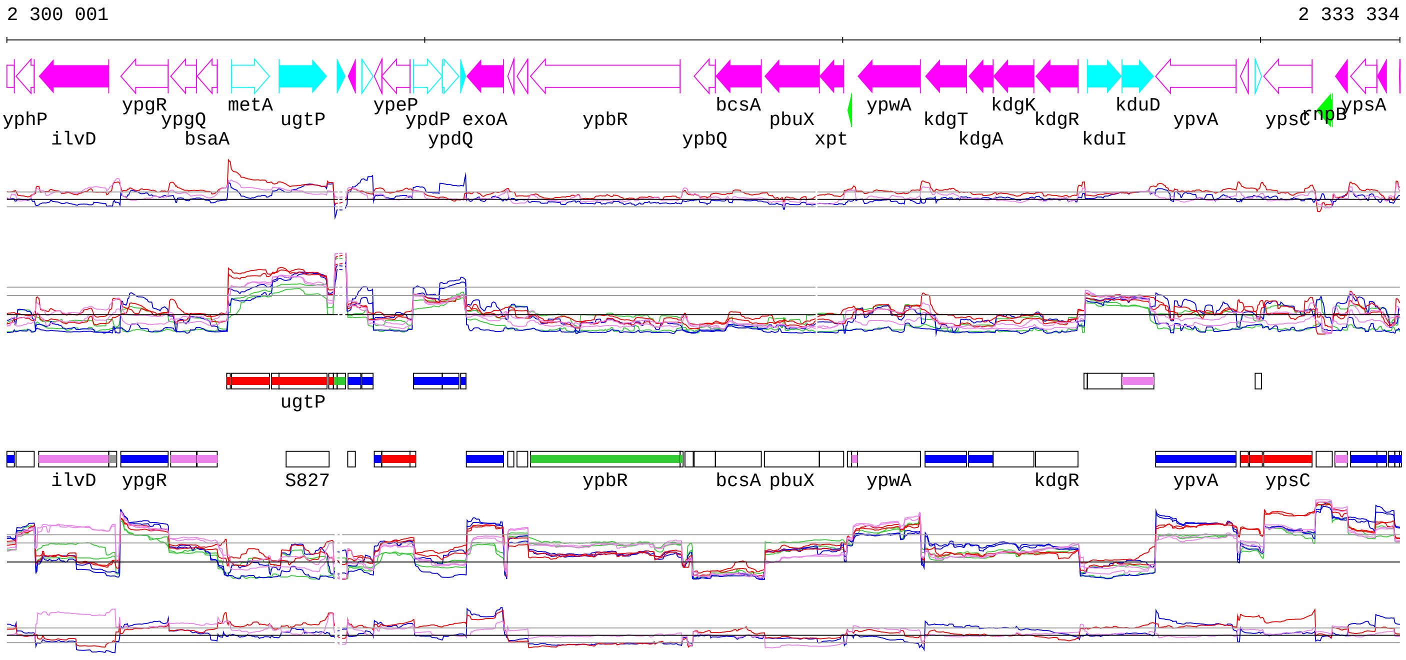
2 300 001
2 333 334
yphP ilvD ypgR ypgQ bsaA metA ugtP ypeP ypdP ypdQ exoA ypbR ypbQ bcsA pbuX xpt ypwA kdgT kdgK kdgA kdgR kduD kduI ypvA ypsC rnpB ypsA
ugtP
ilvD ypgR S827 ypbR bcsA pbuX ypwA kdgR ypvA ypsC

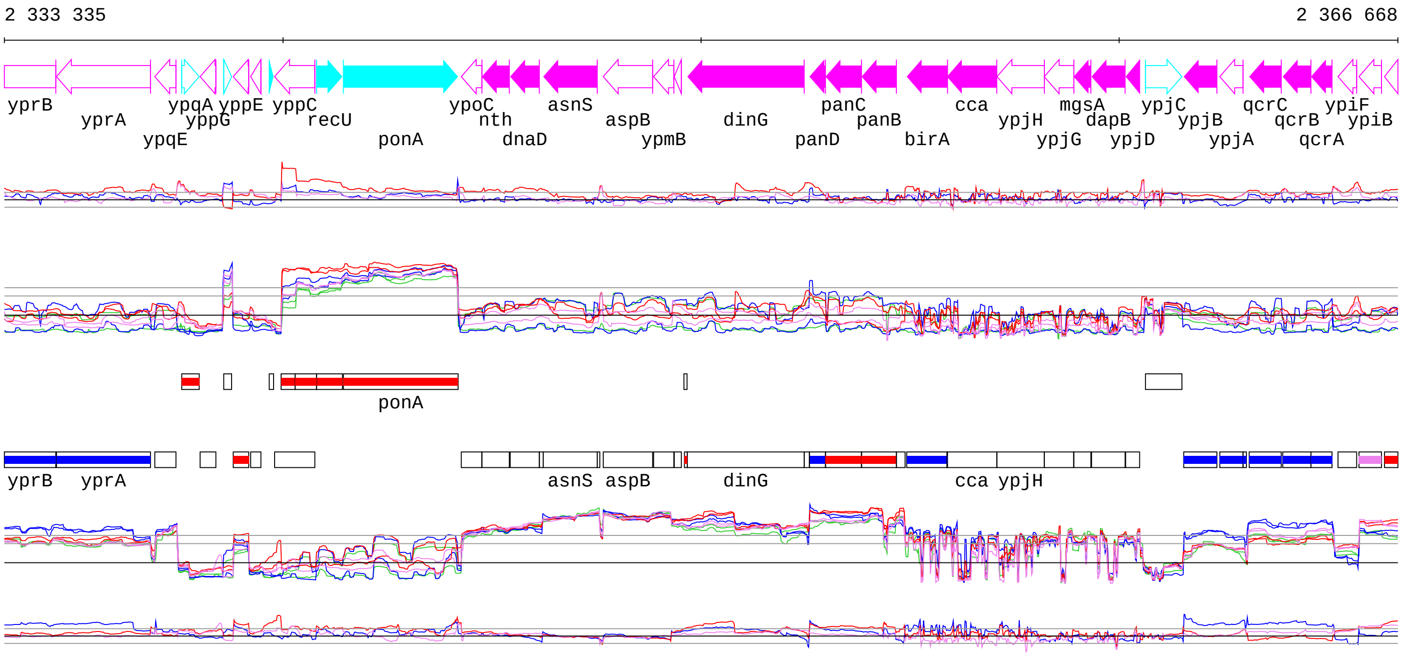
2 333 335
2 366 668
yprB yprA ypqE ypqA yppG yppE yppC recU ponA ypoC nth dnaD asnS aspB ypmB dinG panD panC panB birA cca ypjH ypjG mgsA dapB ypjD ypjC ypjB ypjA qcrC qcrB qcrA ypiF ypiB
ponA
yprB yprA asnS aspB dinG cca ypjH

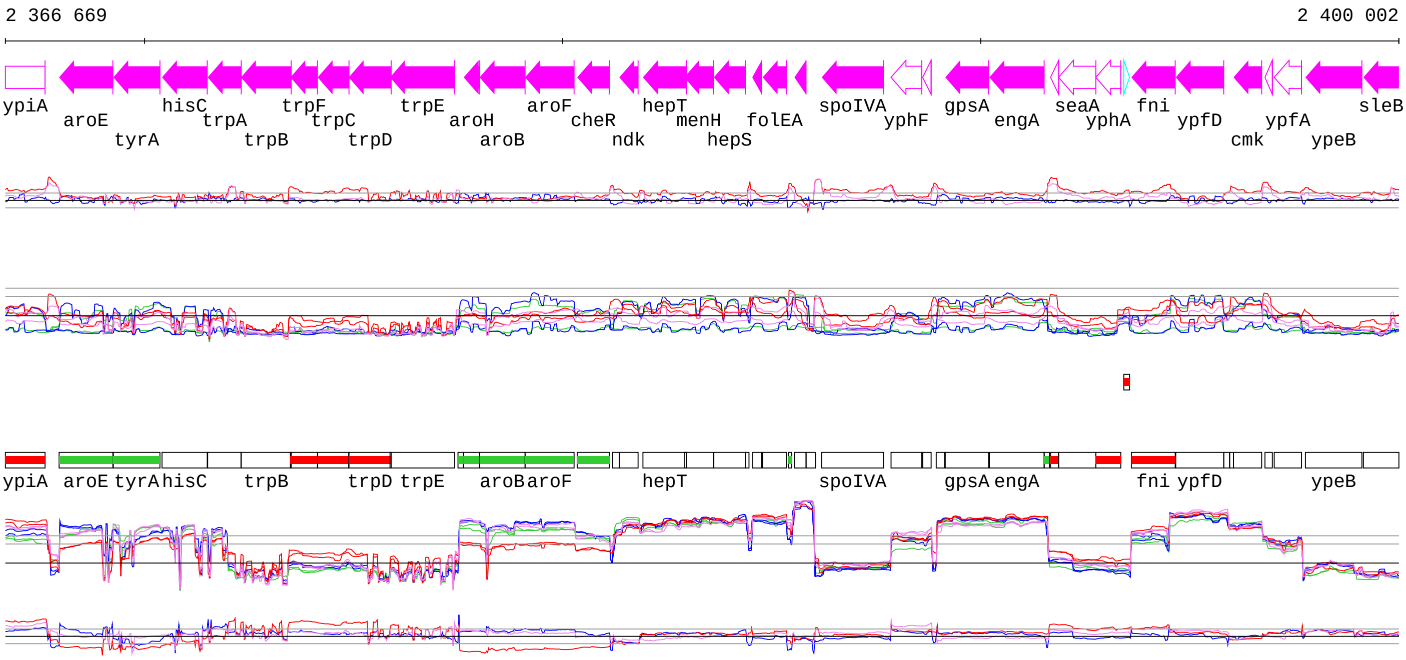
2 366 669
2 400 002
ypiA aroE tyrA hisC trpA trpB trpF trpC trpD trpE aroH aroB aroF cheR ndk hepT menH hepS folEA spoIVA yphF gpsA engA seaA yphA fni ypfD cmk ypfA ypeB sleB
ypiA aroE tyrA hisC trpB trpD trpE aroB aroF hepT spoIVA gpsA engA fni ypfD ypeB

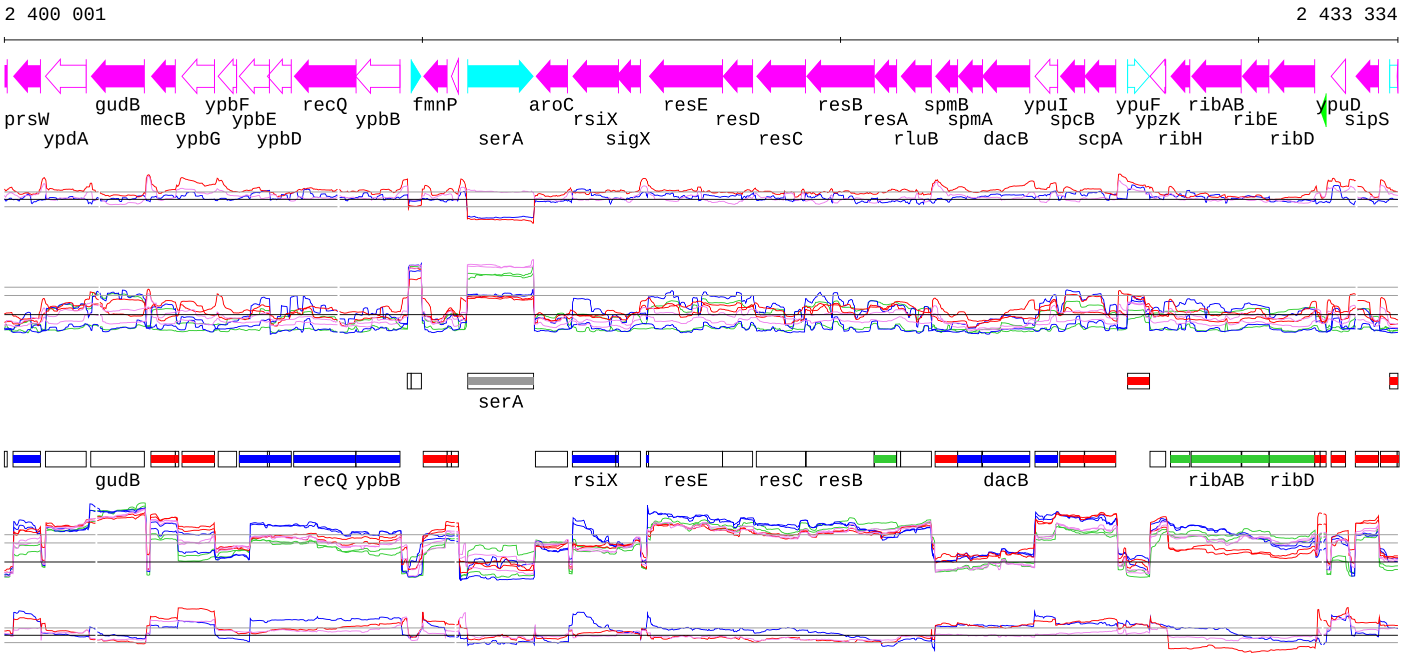
2 400 001
2 433 334
prsW gudB mecB ypbF ypbE recQ ypbB fmnP aroC rsiX resE resD resB resA spmB spmA ypuI spcB ypuF ypzK ribAB ribE ypuD sipS
ypdA ypbG ypbD serA sigX resC rluB dacB scpA ribH ribD
serA
gudB recQ ypbB rsiX resE resC resB dacB ribAB ribD

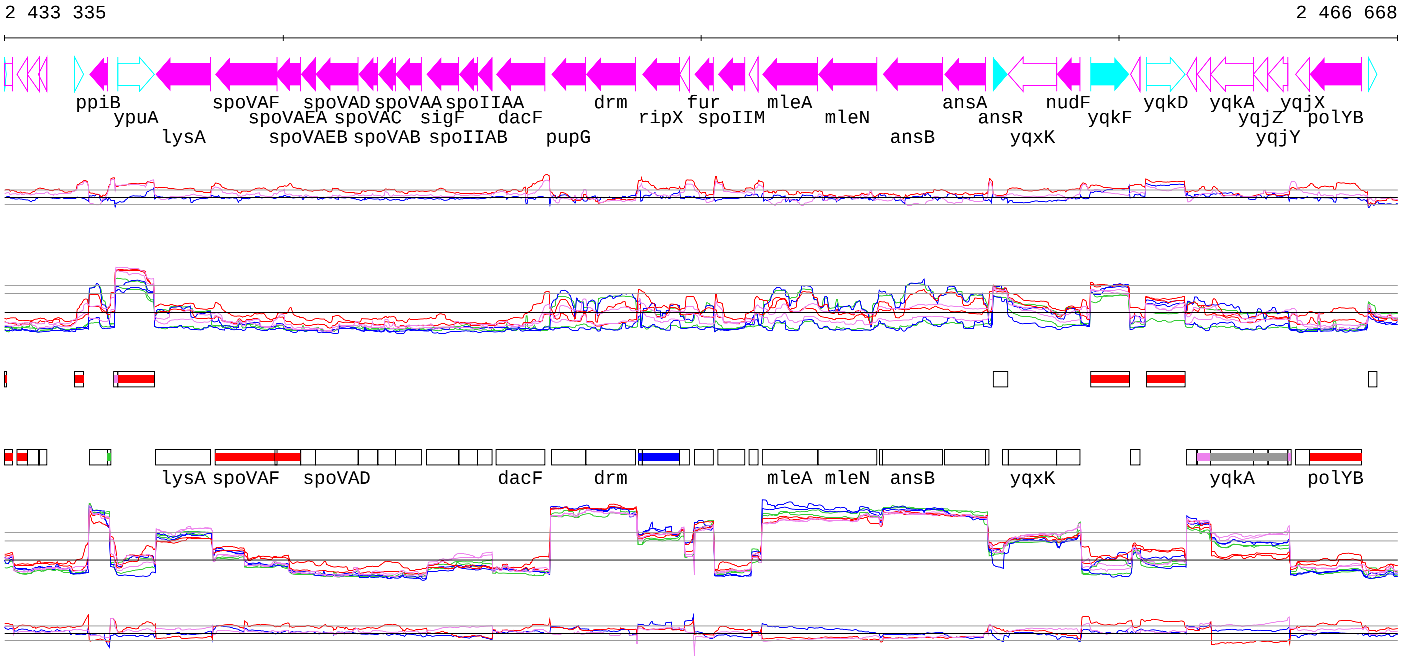
2 433 335
2 466 668
ppiB ypuA spoVAF spoVAEA spoVAD spoVAC spoVAA sigF spoIIAA dacF drm ripX fur spoIIM mleA mleN ansA ansR nudF yqkF yqkD yqkA yqjZ yqjX polYB
lysA spoVAEB spoVAB spoIIAB pupG ansB yqxK yqjY
lysA spoVAF spoVAD dacF drm mleA mleN ansB yqxK yqkA polYB

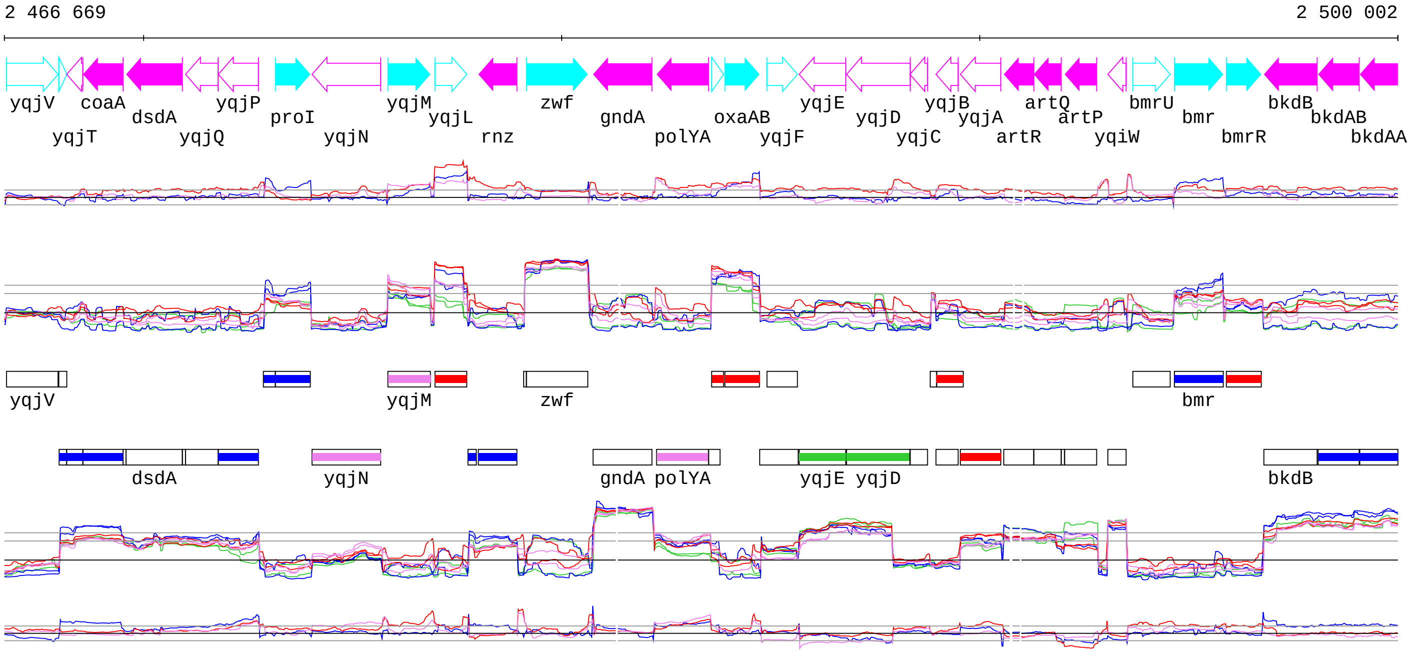
2 466 669
2 500 002
yqjV coaA dsdA yqjP proI yqjM yqjL zwf gndA oxaAB yqjE yqjD yqjB yqjA artQ artP bmrU bmr bkdB bkdAB
yqjT yqjQ yqjN rnz polYA yqjF yqjC artR yqiW bmrR bkdAA
yqjV yqjM zwf bmr
dsdA yqjN gndA polYA yqjE yqjD bkdB

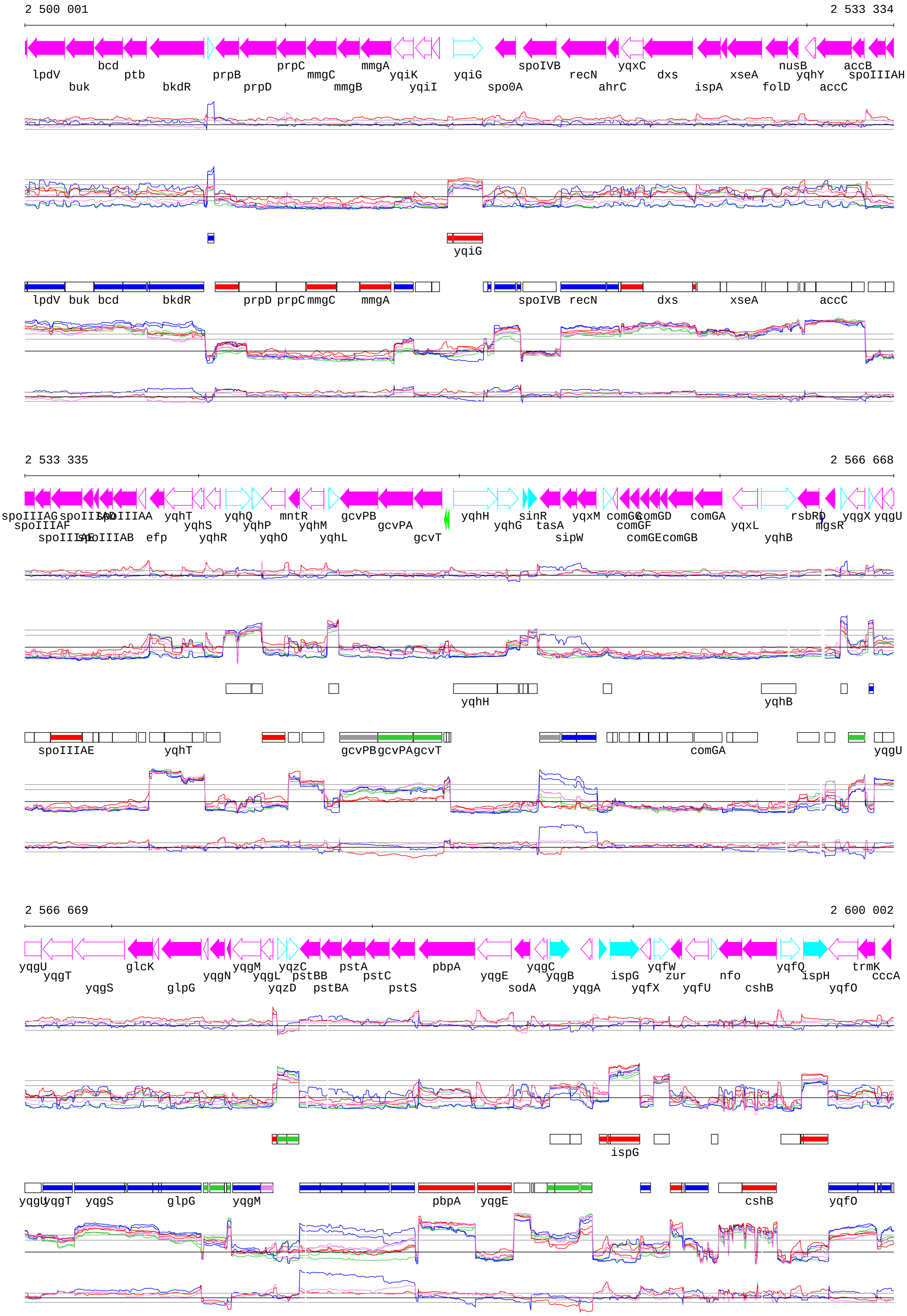

2 500 001
2 533 334
lpdV buk bcd ptb bkdR prpB prpD prpC mmgC mmgB mmgA yqiK yqiI yqiG spo0A spoIVB recN ahrC yqxC dxs ispA xseA nusB folD yqhY accB accC spoIIIAH
yqiG
lpdV buk bcd bkdR prpD prpC mmgC mmgA spoIVB recN dxs xseA accC
2 533 335
2 566 668
spoIIIAG spoIIIAF spoIIIAE spoIIIAD spoIIIAB spoIIIAA efp yqhT yqhS yqhR yqhQ yqhP yqhO mntR yqhM yqhL gcvPB gcvPA gcvT yqhH yqhG sinR tasA sipW yqxM comGG comGF comGE comGD comGB comGA yqxL yqhB rsbRD mgsR yqgX yqgU
yqhH
yqhB
spoIIIAE yqhT gcvPB gcvPA gcvT comGA yqgU
2 566 669
2 600 002
yqgU yqgT yqgS glcK glpG yqgN yqgM yqgL yqzC yqzD pstBB pstBA pstA pstC pstS pbpA yqgE sodA yqgC yqgB yqgA ispG yqfX yqfW zur yqfU nfo cshB yqfQ ispH yqfO trmK cccA
ispG
yqgU yqgT yqgS glpG yqgM pbpA yqgE cshB yqfO

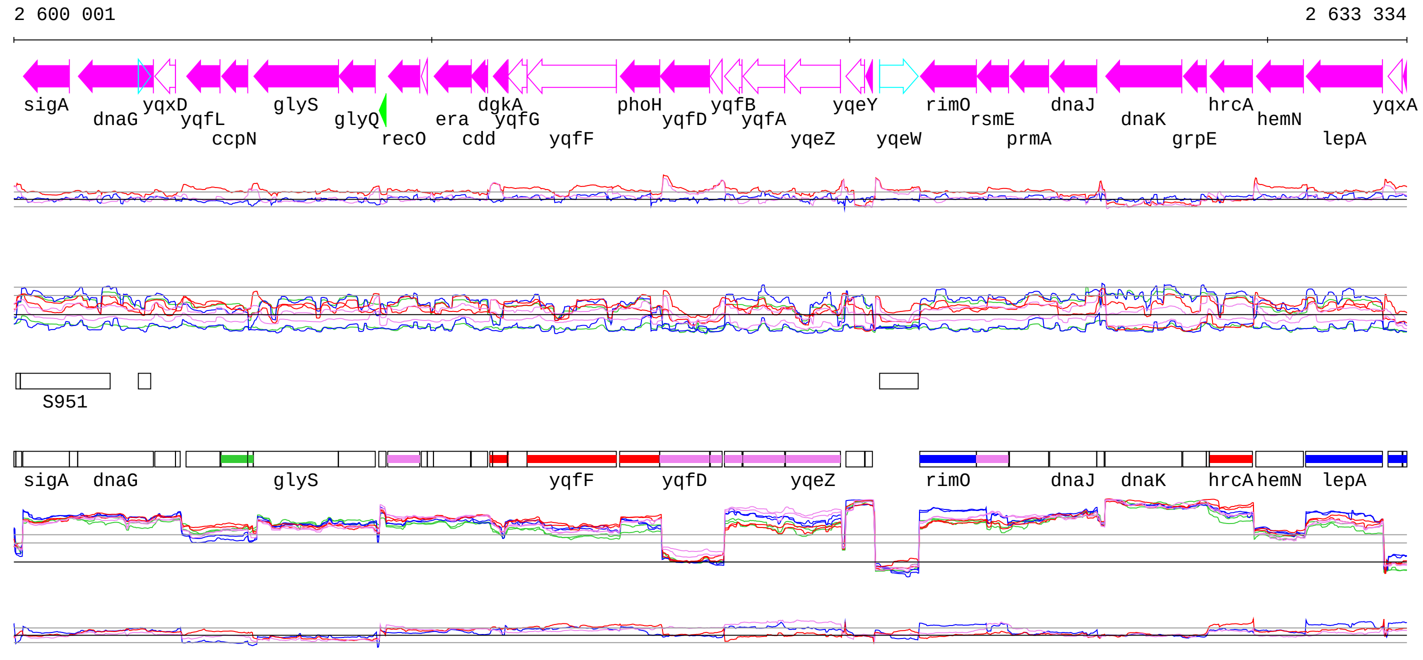
2 600 001
2 633 334
sigA dnaG yqxD yqfL ccpN glyS glyQ recO era dgkA cdd yqfG yqfF phoH yqfD yqfB yqfA yqeZ yqeY yqeW rimO rsmE prmA dnaJ dnaK grpE hrcA hemN lepA yqxA
S951
sigA dnaG glyS yqfF yqfD yqeZ rimO dnaJ dnaK hrcA hemN lepA

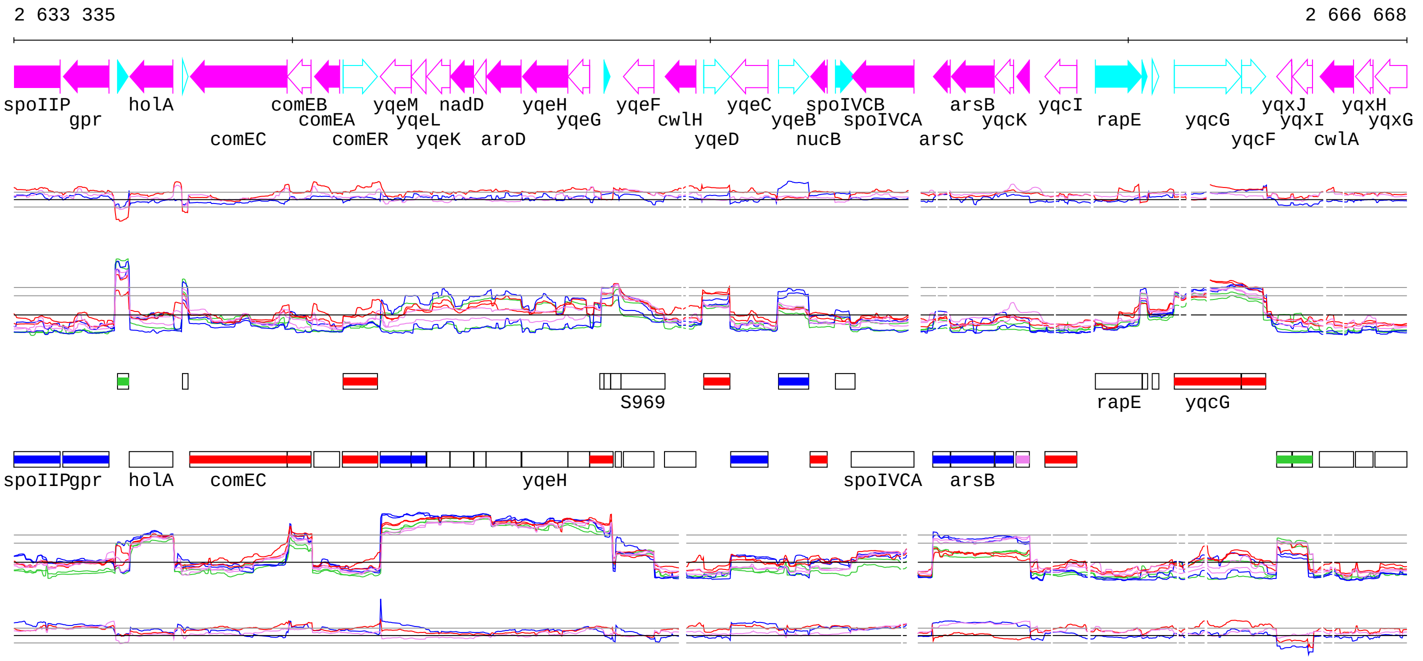
2 633 335
2 666 668
spoIIP gpr holA comEC comEB comEA comER yqeM yqeL yqeK nadD aroD yqeH yqeG yqeF cwlH yqeD yqeC yqeB nucB spoIVCB spoIVCA arsB arsC yqcK yqcI rapE yqcG yqxJ yqcF yqxI cwlA yqxH yqxG
S969 rapE yqcG
spoIIPgpr holA comEC yqeH spoIVCA arsB

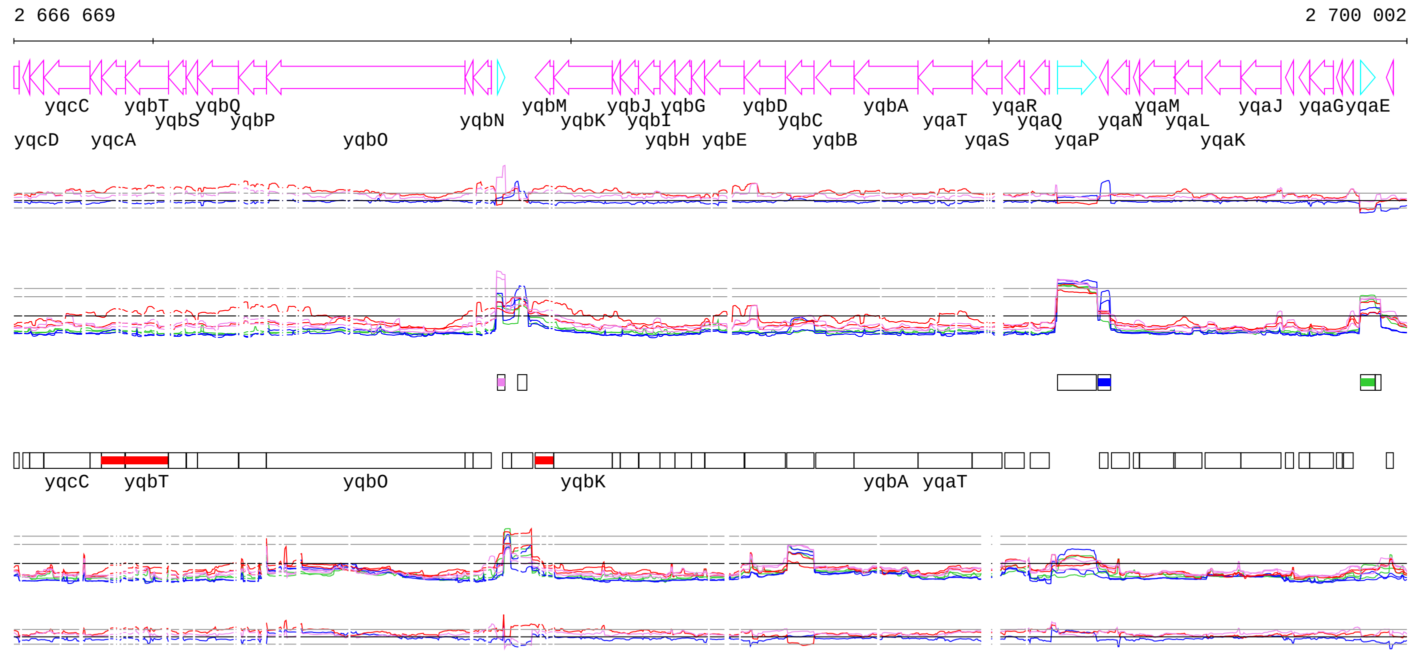
2 666 669
2 700 002
yqcD yqcC yqcA yqbT yqbS yqbQ yqbP yqbO yqbN yqbM yqbK yqbJ yqbI yqbH yqbG yqbE yqbD yqbC yqbB yqbA yqaT yqaS yqaR yqaQ yqaP yqaN yqaM yqaL yqaK yqaJ yqaG yqaE
yqcC yqbT yqbO yqbK yqbA yqaT

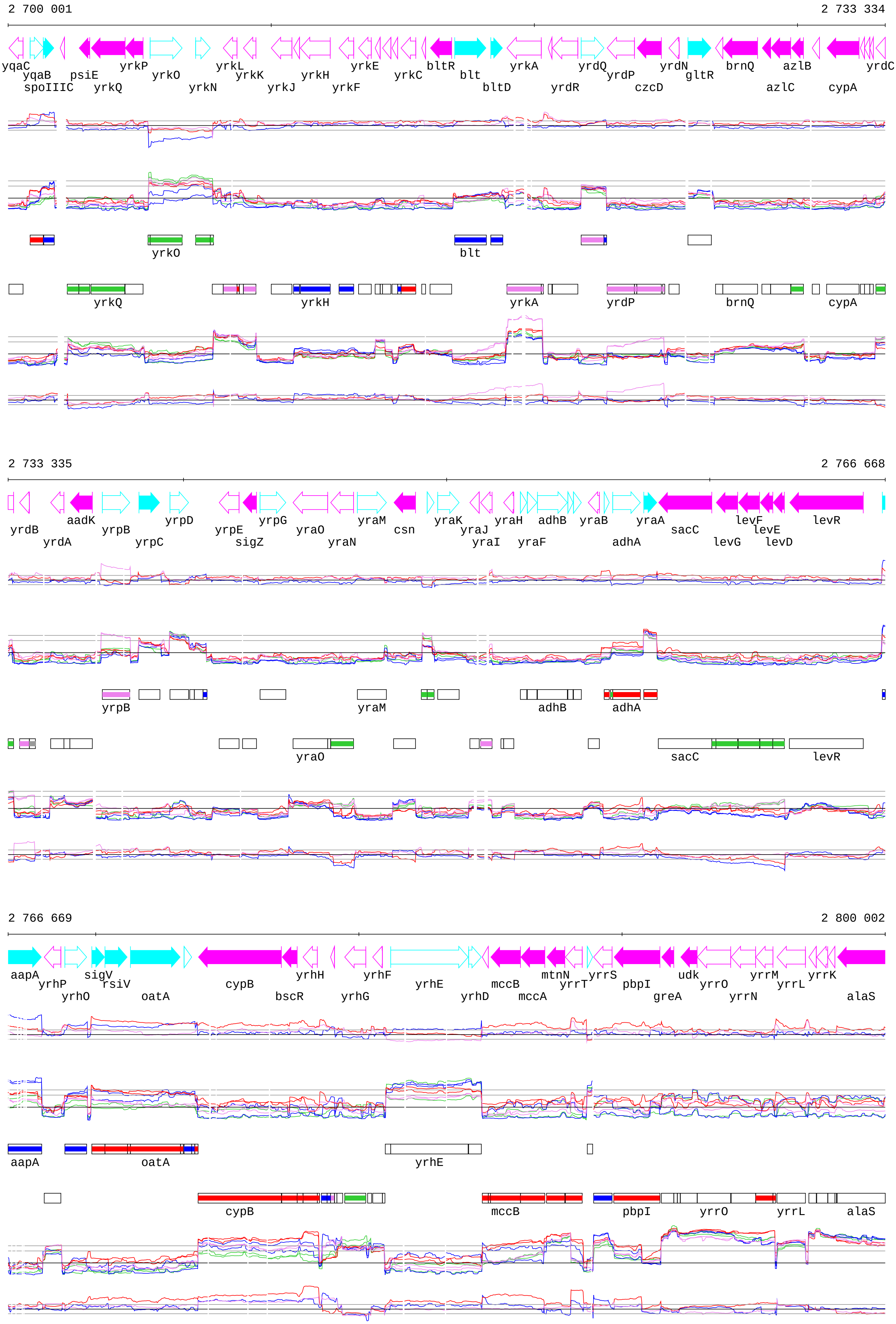

2 700 001
2 733 334
yqaC yrkP yrkL yrkE bltR yrkA yrdQ yrdN brnQ azlB yrdC
yqaB psiE yrkO yrkK yrkH yrkC blt yrdP gltR
spoIIIC yrkQ yrkN yrkJ yrkF bltD yrdR czcD azlC cypA
yrkO
blt
yrkQ
yrkH
yrkA
yrdP
brnQ
cypA
2 733 335
2 766 668
aadK yrpD yrpG yraM yraK yraH adhB yraB yraA levF levR
yrdB yrpB yrpE yraO csn yraJ sacC levE
yrdA yrpC sigZ yraN yraI yraF adhA levG levD
yrpB
yraM
adhB
adhA
yraO
sacC
levR
2 766 669
2 800 002
aapA sigV yrhH yrhF mtnN yrrS udk yrrM yrrK
yrhP rsiV cypB yrhE mccB yrrT pbpI yrrO yrrL
yrhO oatA bscR yrhG yrhD mccA greA yrrN alaS
aapA
oatA
yrhE
cypB
mccB
pbpI
yrrO
yrrL
alaS

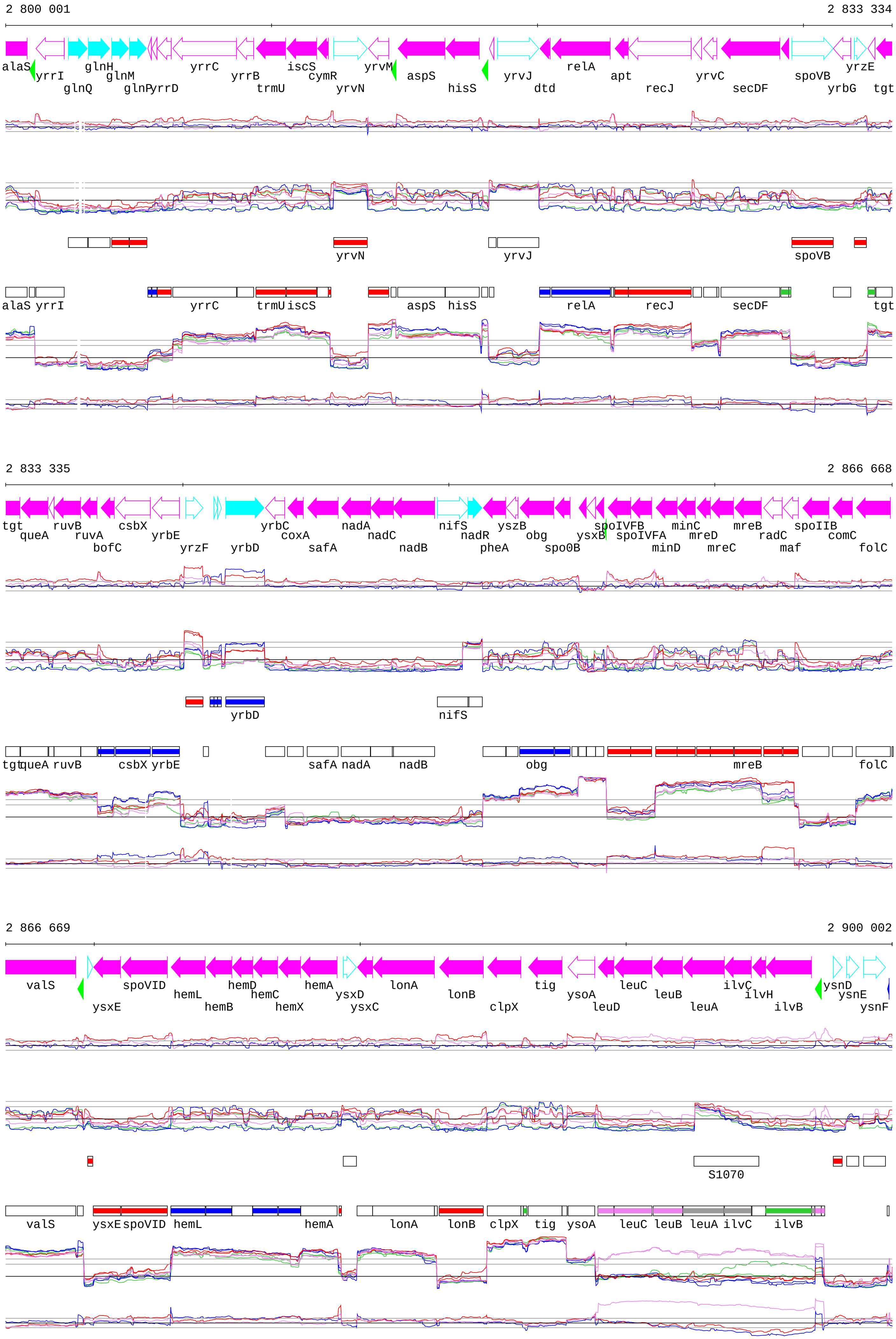
2 800 001
2 833 334
alaS yrrI glnQ glnH glnM glnP yrrD yrrC yrrB trmU iscS cymR yrvN yrvM aspS hisS yrvJ dtd relA apt recJ yrvC secDF spoVB yrbG yrzE tgt
yrvN yrvJ spoVB
alaS yrrI yrrC trmU iscS aspS hisS relA recJ secDF tgt
2 833 335
2 866 668
tgt queA ruvB ruvA bofC csbX yrbE yrzF yrbD yrbC coxA safA nadA nadC nadB nifS nadR pheA yszB obg spo0B ysxB spoIVFB spoIVFA minC minD mreD mreC mreB radC maf spoIIB comC folC
yrbD nifS
tgt queA ruvB csbX yrbE safA nadA nadB obg mreB folC
2 866 669
2 900 002
valS ysxE spoVID hemL hemB hemD hemC hemX hemA ysxD ysxC lonA lonB clpX tig ysoA leuD leuC leuB leuA ilvC ilvH ilvB ysnD ysnE ysnF
S1070
valS ysxE spoVID hemL hemA lonA lonB clpX tig ysoA leuC leuB leuA ilvC ilvB

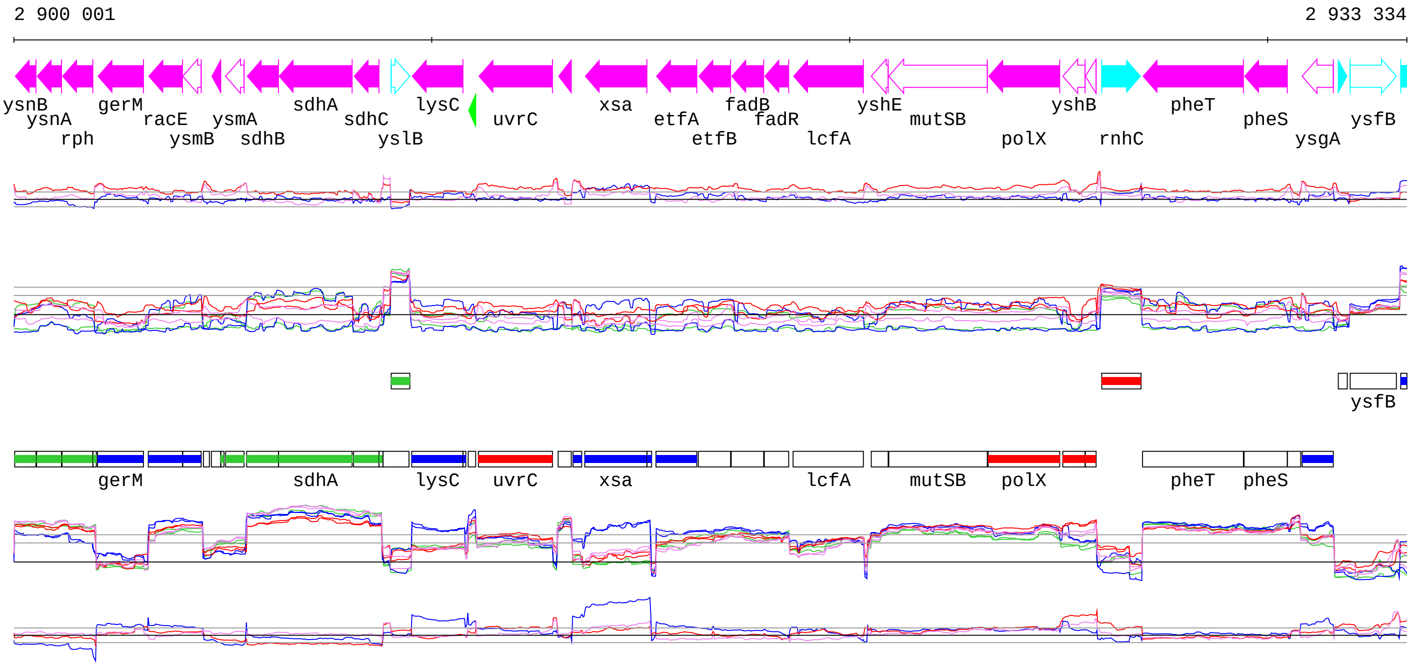
2 900 001
2 933 334
ysnB ysnA rph germM racE ysmB ysmA sdhB sdhA sdhC yslB lysC uvrC xsa etfA etfB fadB fadR lcfA yshE mutSB polX yshB rnhC pheT pheS ysgA ysfB
ysfB
germM sdhA lysC uvrC xsa lcfA mutSB polX pheT pheS

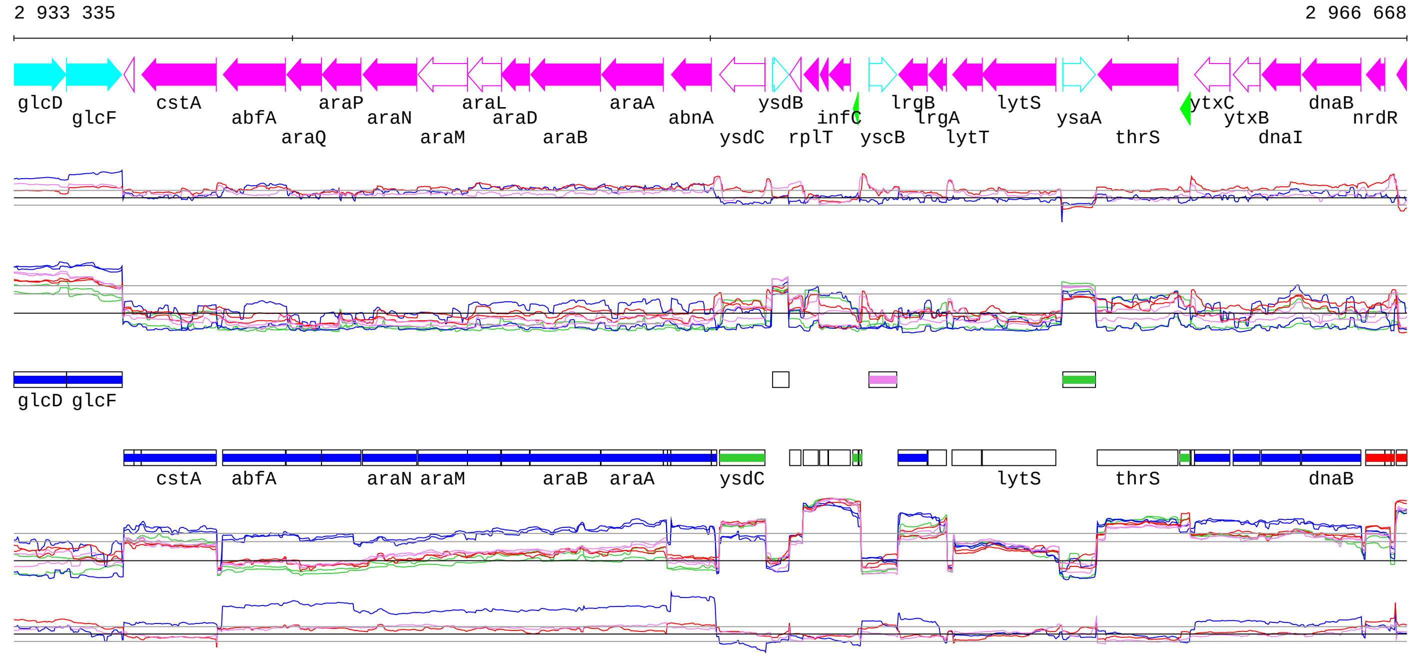
2 933 335
2 966 668
glcD glcF cstA abfA araQ araP araN araM araL araD araB araA abnA ysdC ysdB rplT infC yscB lrgB lrgA lytT lytS ysaA thrS ytxC ytxB dnaI dnaB nrdR
glcD glcF
cstA abfA araN araM araB araA ysdC lytS thrS dnaB

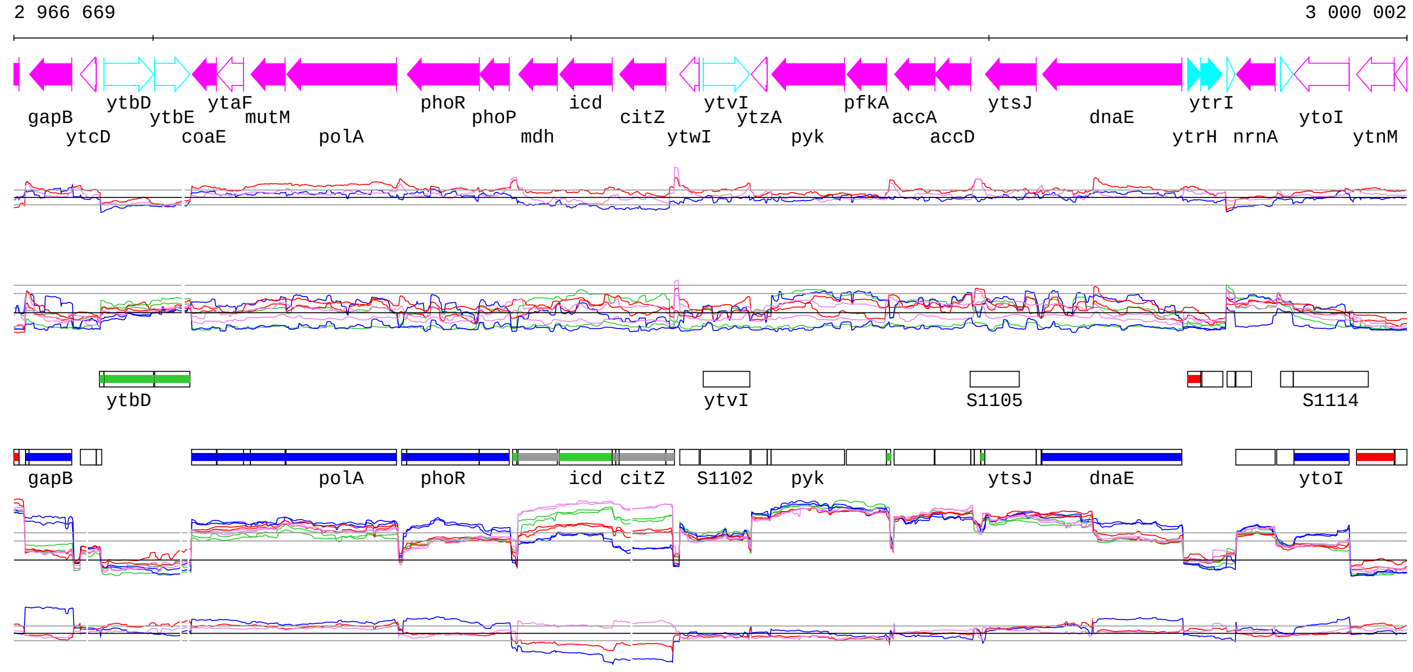
2 966 669
3 000 002
gapB ytcD ytbD ytbE coaE ytaF mutM polA phoR phoP mdh icd citZ ytwI ytvI ytzA pyk pfkA accA accD ytsJ dnaE ytrI ytrH nrnA ytoI ytnM
ytbD ytvI S1105 S1114
gapB polA phoR icd citZ S1102 pyk ytsJ dnaE ytoI

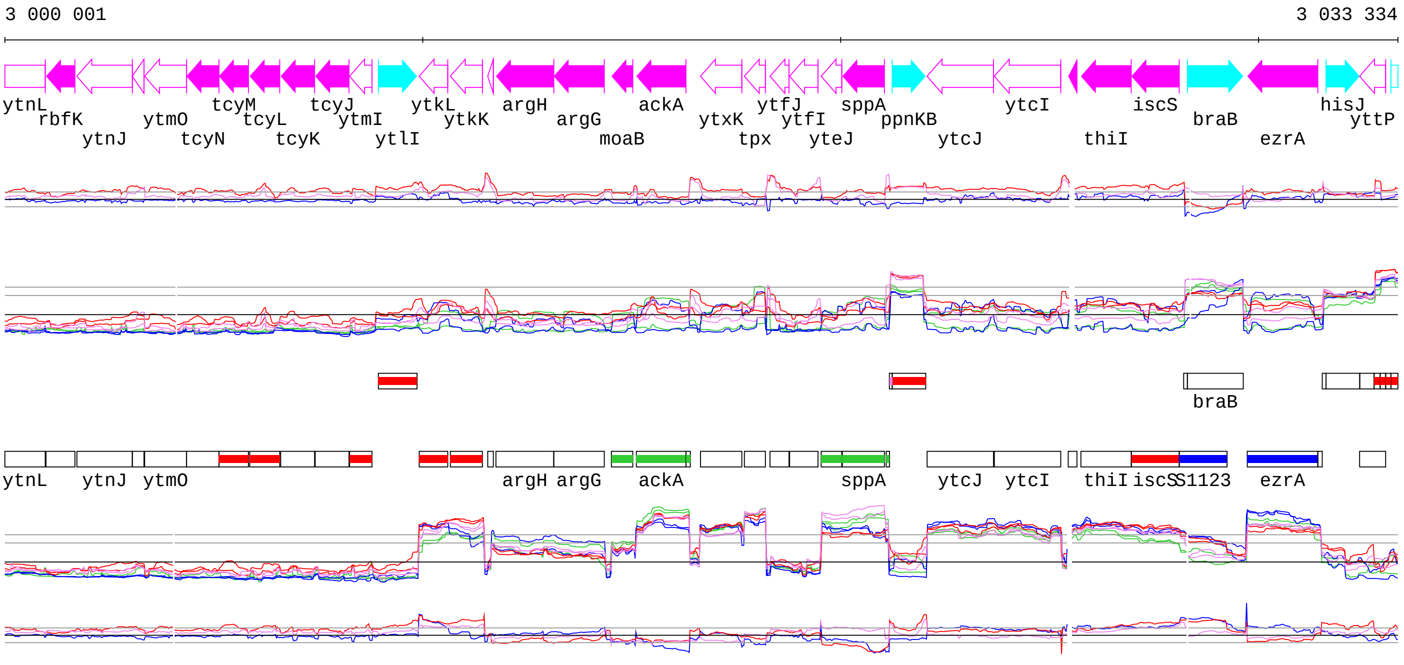
3 000 001
3 033 334
ytnL rbfK ytnJ ytmO tcyM tcyL tcyN tcyJ ytmI tcyK ytlI ytkL ytkK argH argG moaB ackA ytxK tpx ytfJ ytfI yteJ sppA ppnKB ytcJ ytcI thiI iscS braB ezrA hisJ yttP
braB
ytnL ytnJ ytmO argH argG ackA sppA ytcJ ytcI thiI iscS S1123 ezrA

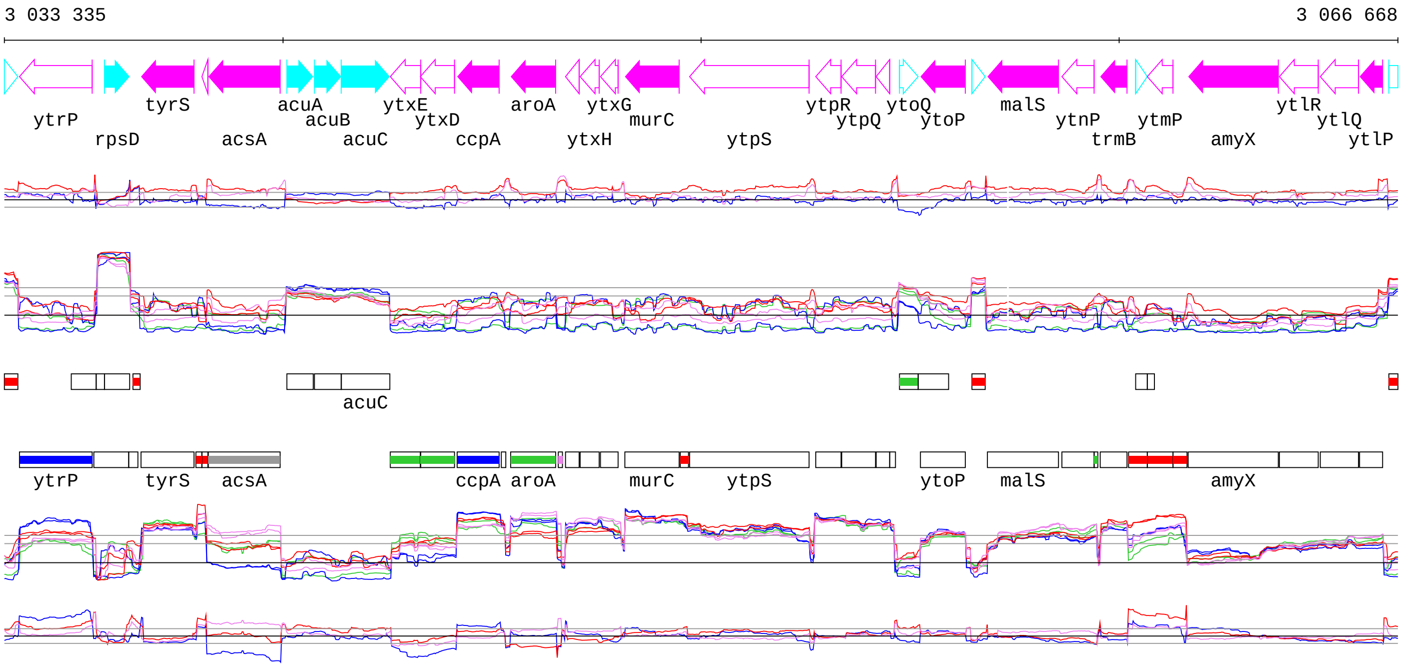
3 033 335
3 066 668
ytrP rpsD tyrS acsA acuA acuB acuC ytxE ytxD ccpA aroA ytxH ytxG murC ytpS ytpR ytpQ ytoQ ytoP malS ytnP trmB ytmP amyX ytlR ytlQ ytlP
acuC
ytrP tyrS acsA ccpA aroA murC ytpS ytoP malS amyX

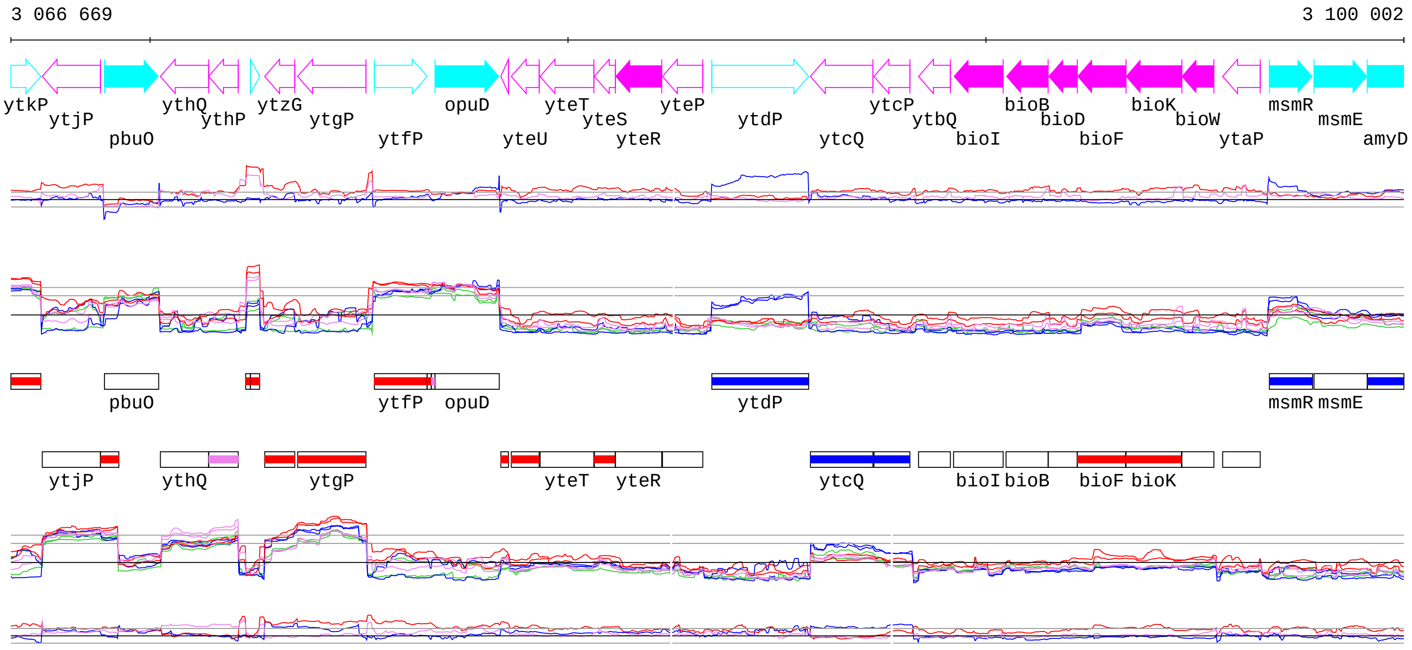
3 066 669
3 100 002
ytkP ytjP pbuO ythQ ythP ytzG ytgP ytfP opuD yteU yteT yteS yteR yteP ytdP ytcQ ytcP ytbQ bioI bioB bioD bioF bioK bioW ytaP msmR msmE amyD
pbuO ytfP opuD ytdP msmR msmE
ytjP ythQ ytgP yteT yteR ytcQ bioI bioB bioF bioK

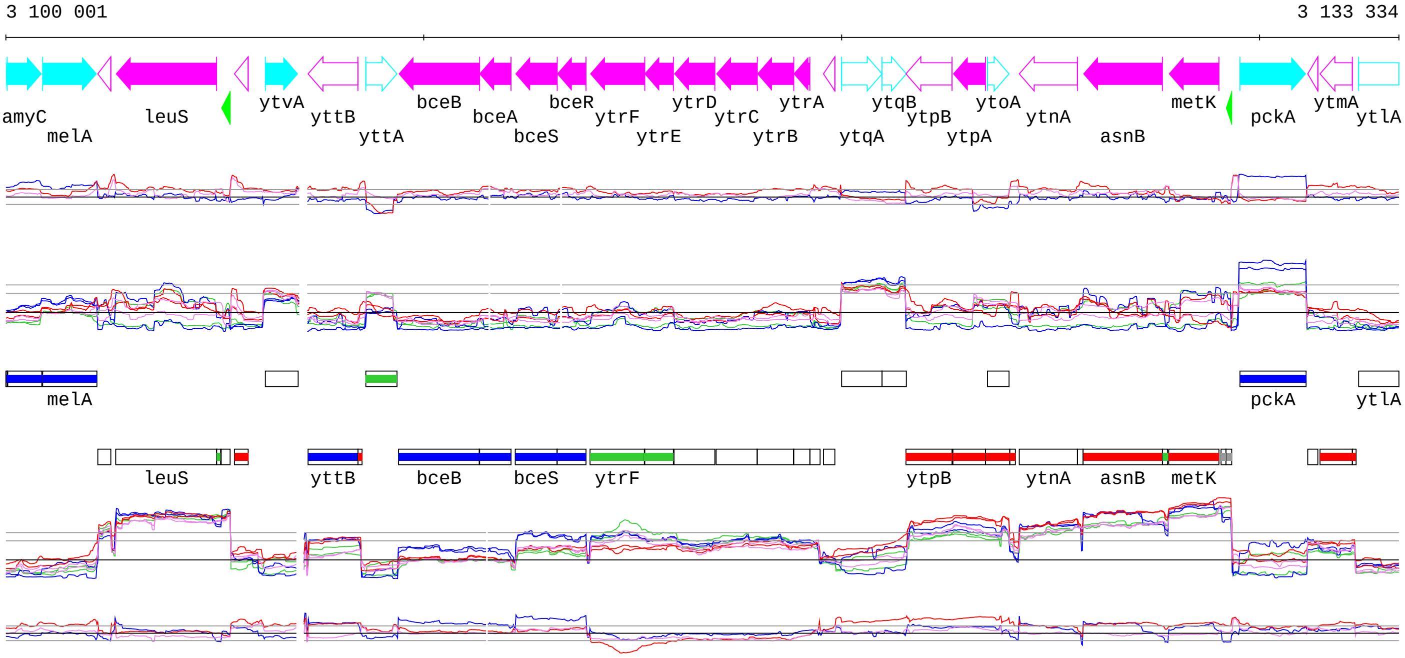

3 100 001
3 133 334
amyC melA leuS ytvA yttB yttA bceB bceA bceS bceR ytrF ytrE ytrD ytrC ytrB ytrA ytqA ytqB ytpB ytpA ytoA ytnA asnB metK pckA ytmA ytlA
melA
pckA
ytlA
leuS
yttB
bceB
bceS
ytrF
ytpB
ytnA
asnB
metK

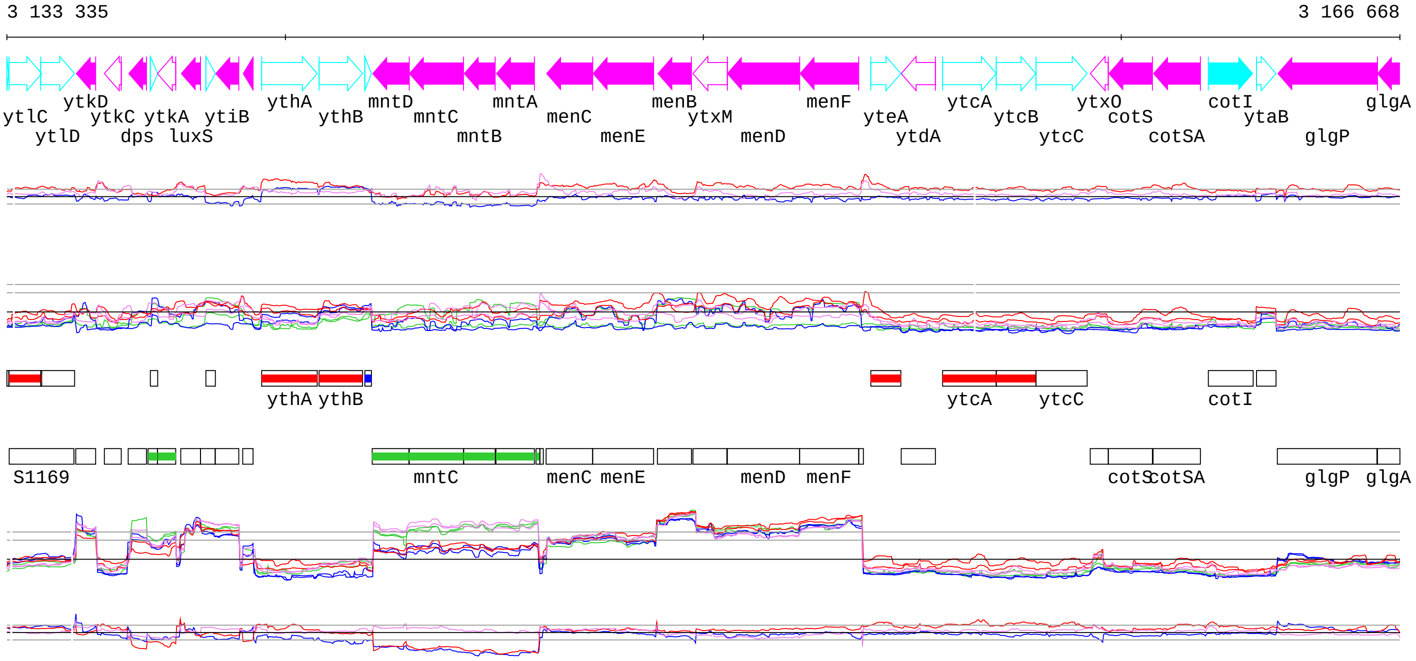

3 133 335
3 166 668
ytlC ytlD ytkD ytkC dps ytkA luxS ytiB ythA ythB mntD mntC mntB mntA menC menE menB ytxM menD menF yteA ytdA ytcA ytcB ytcC ytxO cotS cotSA cotI ytaB glgP glgA
ythA ythB
ytcA ytcC
cotI
S1169
mntC
menC menE
menD menF
cotS cotSA
glgP glgA

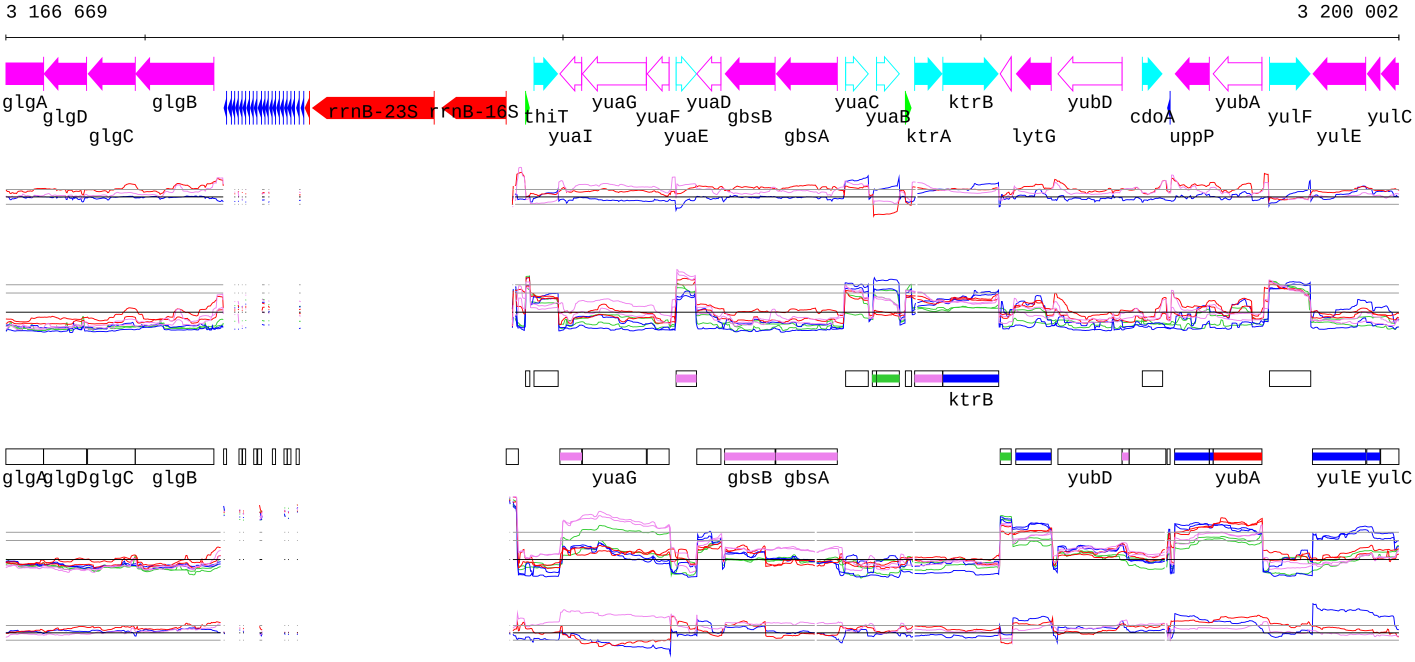

3 166 669
3 200 002
glgA glgD glgC glgB rrnB-23S rrnB-16S thiT yuaI yuaG yuaF yuaE yuaD gbsB gbsA yuaC yuaB ktrA ktrB lytG yubD cdoA uppP yubA yulF yulE yulC
ktrB
glgA glgD glgC glgB
yuaG
gbsB gbsA
yubD
yubA
yulE yulC

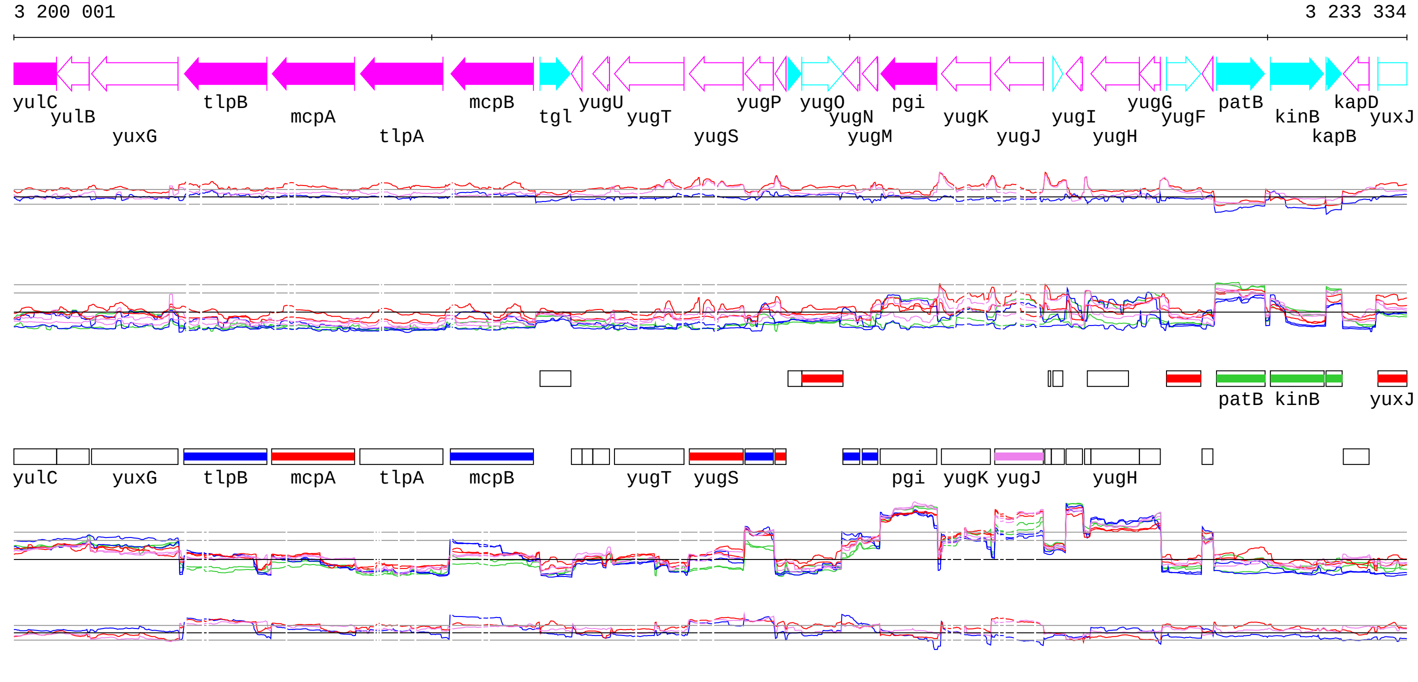
3 200 001
3 233 334
yulC yulB yuxG tlpB mcpA tlpA mcpB tgl yugU yugT yugS yugP yugO yugN yugM pgi yugK yugJ yugI yugH yugG yugF patB kinB kapB kapD yuxJ
patB kinB yuxJ
yulC yuxG tlpB mcpA tlpA mcpB yugT yugS pgi yugK yugJ yugH

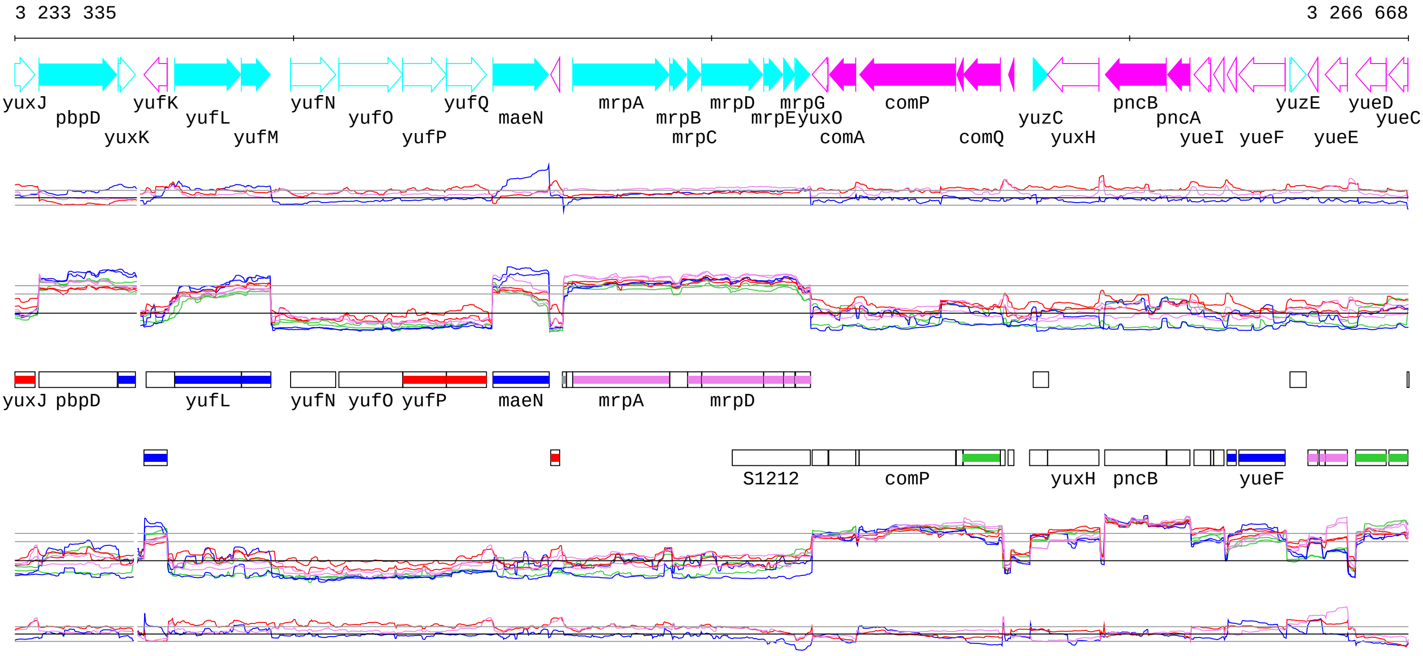
3 233 335
3 266 668
yuxJ pbpD yuxK yufK yufL yufM yufN yufO yufP yufQ maeN mrpA mrpB mrpC mrpD mrpG mrpE yuxO comA comP comQ yuzC yuxH pncB pncA yueI yuzE yueF yueD yueE yueC
yuxJ pbpD yufL yufN yufO yufP maeN mrpA mrpD
S1212 comP yuxH pncB yueF

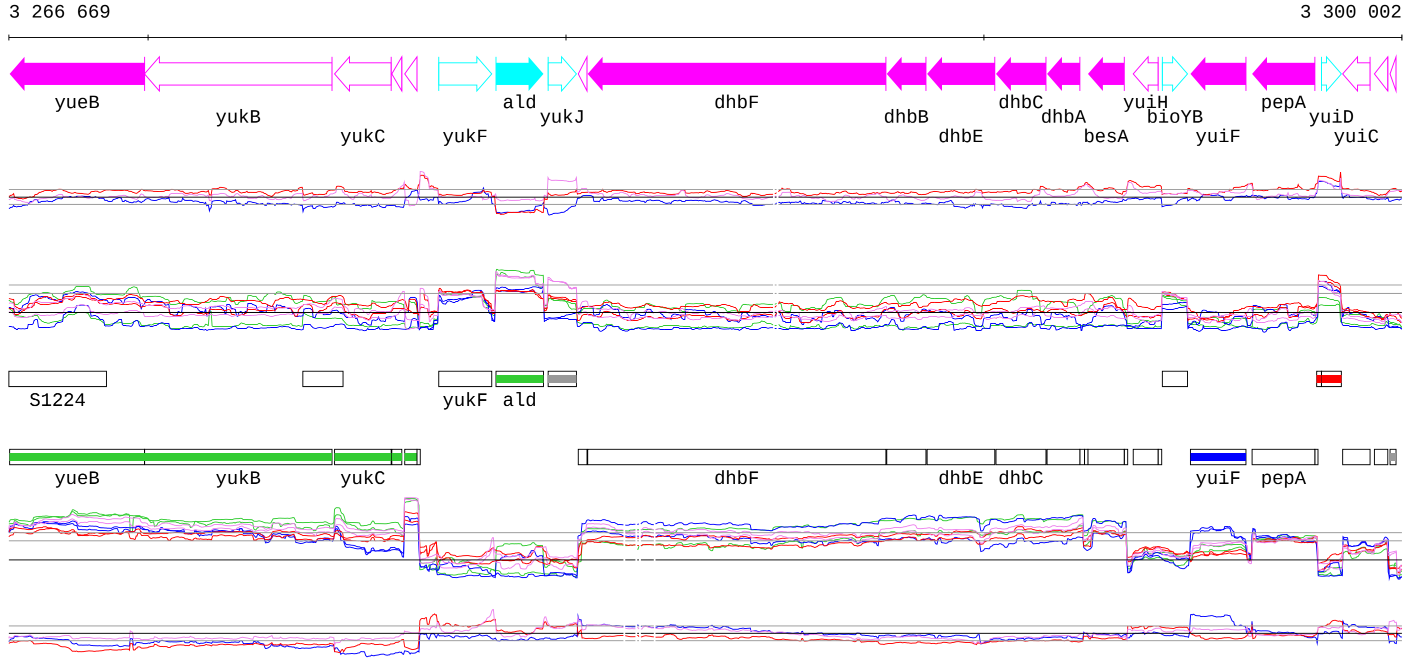
3 266 669
3 300 002
yueB yukB yukC yukF ald yukJ dhbF dhbB dhbE dhbC dhbA besA yuiH bioYB yuiF pepA yuiD yuiC
S1224 yukF ald
yueB yukB yukC dhbF dhbE dhbC yuiF pepA

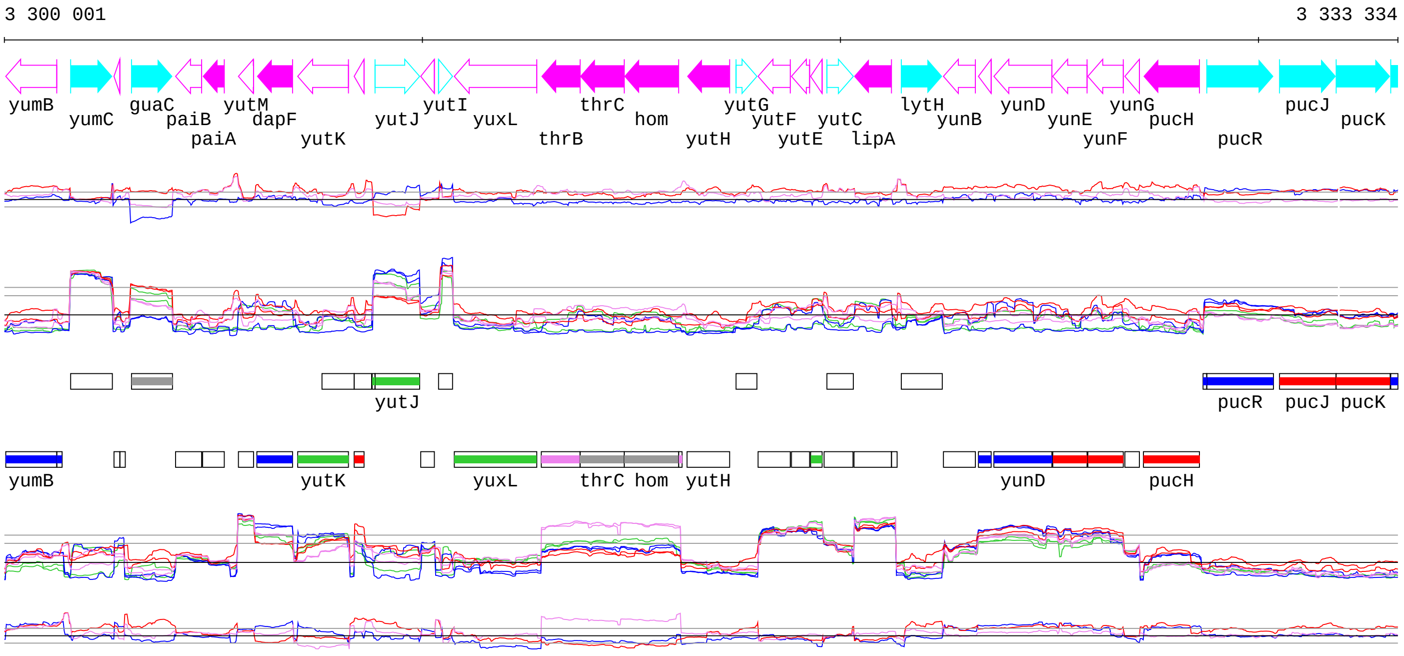
3 300 001
3 333 334
yumB yumC guaC paiB paiA yutM dapF yutK yutJ yutI yuxL thrC thrB hom yutH yutG yutF yutE yutC lipA lytH yunB yunD yunE yunF yunG pucH pucR pucJ pucK
yutJ pucR pucJ pucK
yumB yutK yuxL thrC hom yutH yunD pucH

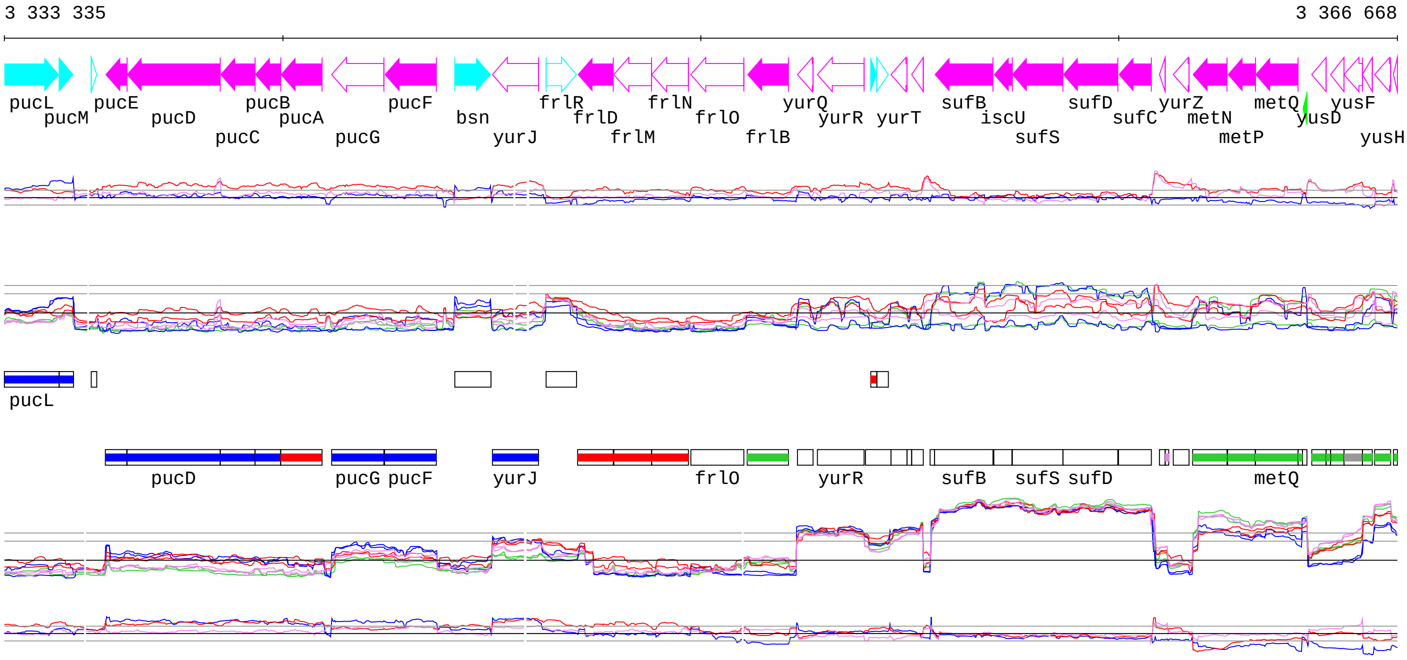
3 333 335
3 366 668
pucL pucM pucE pucD pucC pucB pucA pucG pucF bsn yurJ frlR frlD frlM frlN frlO frlB yurQ yurR yurT sufB iscU sufS sufD sufC yurZ metN metP metQ yusD yusF yusH
pucL
pucD pucG pucF yurJ frlO yurR sufB sufS sufD metQ

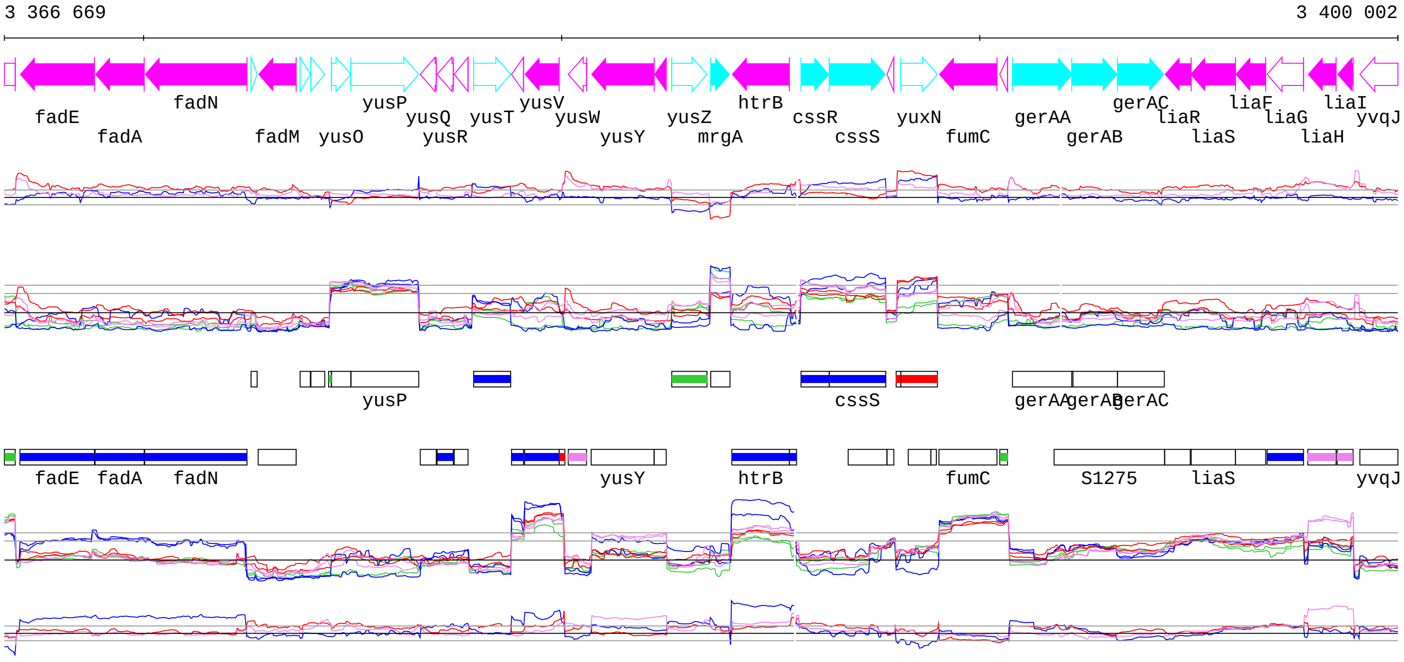
3 366 669
3 400 002
fadE fadA fadN fadM yusO yusP yusQ yusR yusT yusV yusW yusY yusZ mrgA htrB cssR cssS yuxN fumC gerAA gerAB gerAC liaR liaS liaF liaG liaH liaI yvqJ
yusP cssS gerAA gerAB gerAC
fadE fadA fadN yusY htrB fumC S1275 liaS yvqJ

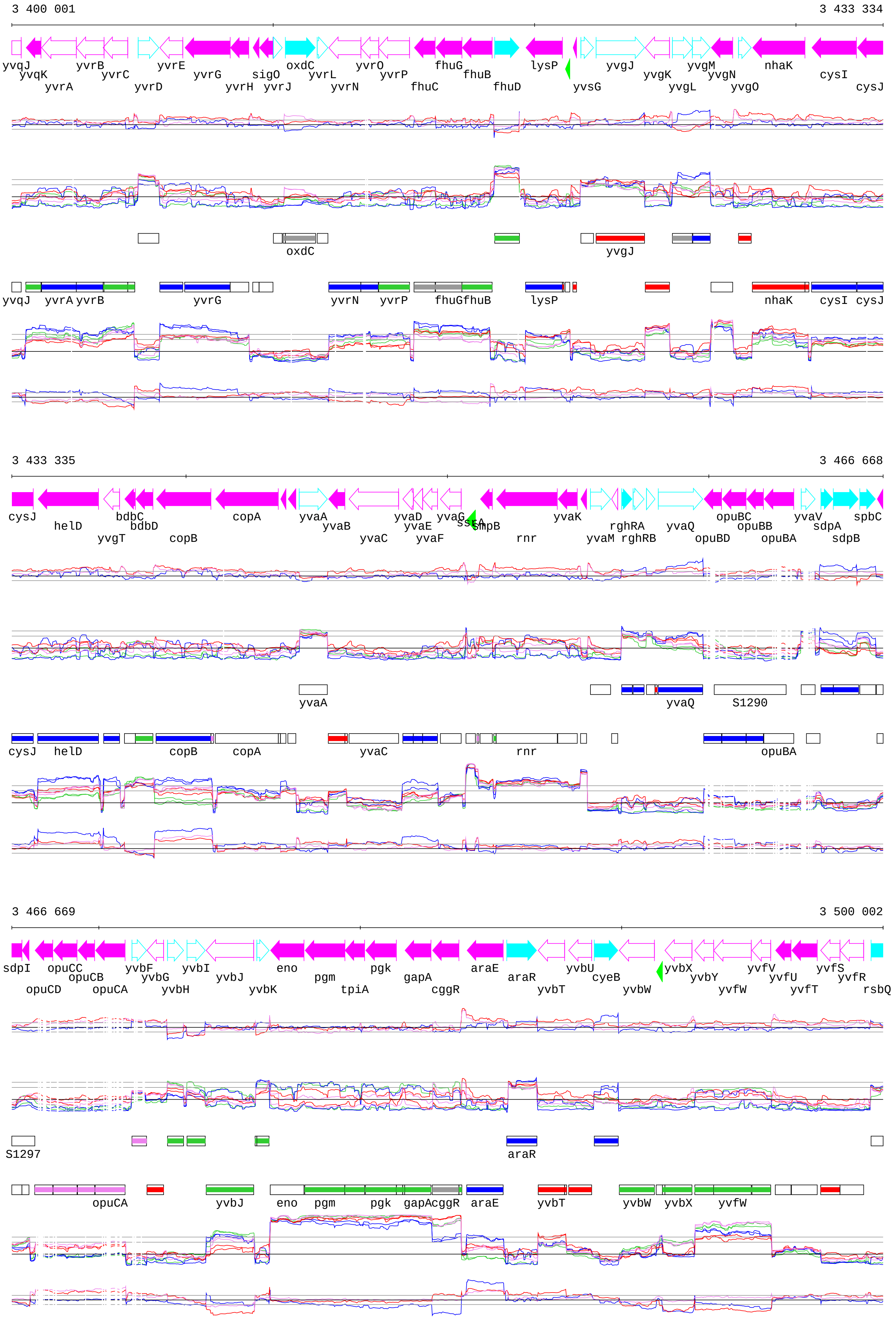
3 400 001
3 433 334
yvqJ yvqK yvrA yvrB yvrC yvrD yvrE yvrG yvrH sigO yvrJ oxdC yvrL yvrN yvrO yvrP fhuC fhuG fhuB fhuD lysP yvsG yvgJ yvgK yvgL yvgM yvgN yvgO nhaK cysI cysJ
oxdC
yvgJ
yvqJ yvrA yvrB yvrG yvrN yvrP fhuGfhuB lysP nhaK cysI cysJ
3 433 335
3 466 668
cysJ helD yvgT bdbC bdbD copB copA yvaA yvaB yvaC yvaD yvaE yvaF yvaG smpB rnr yvaK yvaM rghRA rghRB yvaQ opuBD opuBC opuBB opuBA yvaV sdpA sdpB spbC
yvaA yvaQ S1290
cysJ helD copB copA yvaC rnr opuBA
3 466 669
3 500 002
sdpI opuCD opuCC opuCB opuCA yvbF yvbG yvbH yvbI yvbJ yvbK eno pgm tpiA pgk gapA cggR araE araR yvbT yvbU cyeB yvbW yvbX yvbY yvfW yvfV yvfU yvfT yvfS yvfR rsbQ
S1297 araR
opuCA yvbJ eno pgm pgk gapAcggR araE yvbT yvbW yvbX yvfW

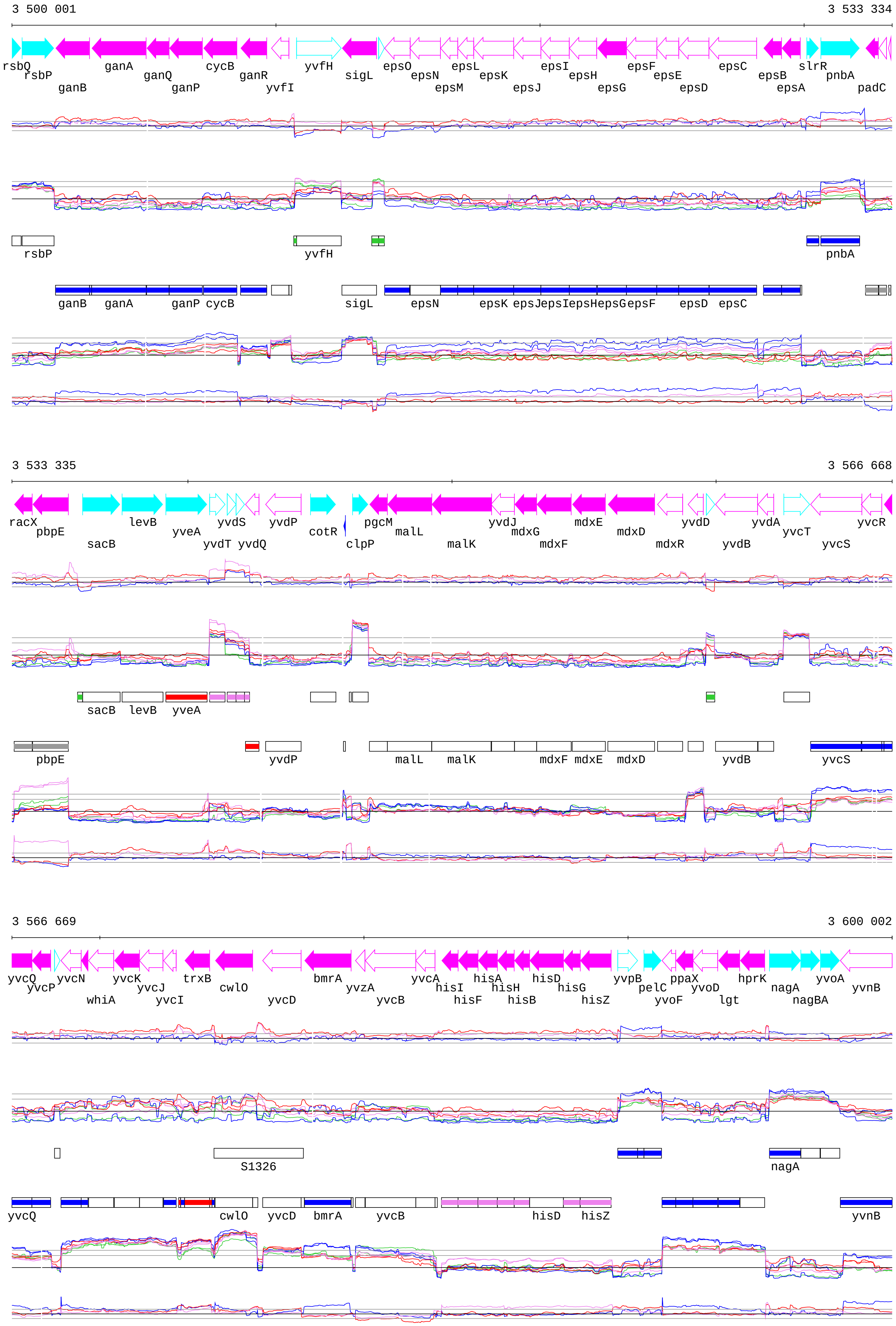

3 500 001
3 533 334
rsbQ rsbP ganB ganA ganQ ganP cycB ganR yvfI yvfH sigL epsO epsN epsM epsL epsK epsJ epsI epsH epsG epsF epsE epsD epsC epsB epsA slrR pnbA padC
rsbP
yvfH
pnbA
ganB ganA ganP cycB
sigL epsN epsK epsJepsIepsHepsGepsF epsD epsC
3 533 335
3 566 668
racX pbpE sacB levB yveA yvdS yvdT yvdQ yvdP cotR clpP pgcM malL malK yvdJ mdxG mdxF mdxE mdxD mdxR yvdD yvdB yvdA yvcT yvcS yvcR
sacB levB yveA
pbpE yvdP malL malK mdxF mdxE mdxD yvdB yvcS
3 566 669
3 600 002
yvcQ yvcP yvcN whiA yvcK yvcJ yvcI trxB cwlO yvcD bmrA yvzA yvcB yvcA hisI hisF hisA hisH hisB hisD hisG hisZ yvpB pelC yvoF ppaX yvoD lgt hprK nagA nagBA yvoA yvnB
S1326
nagA
yvcQ cwlO yvcD bmrA yvcB hisD hisZ yvnB

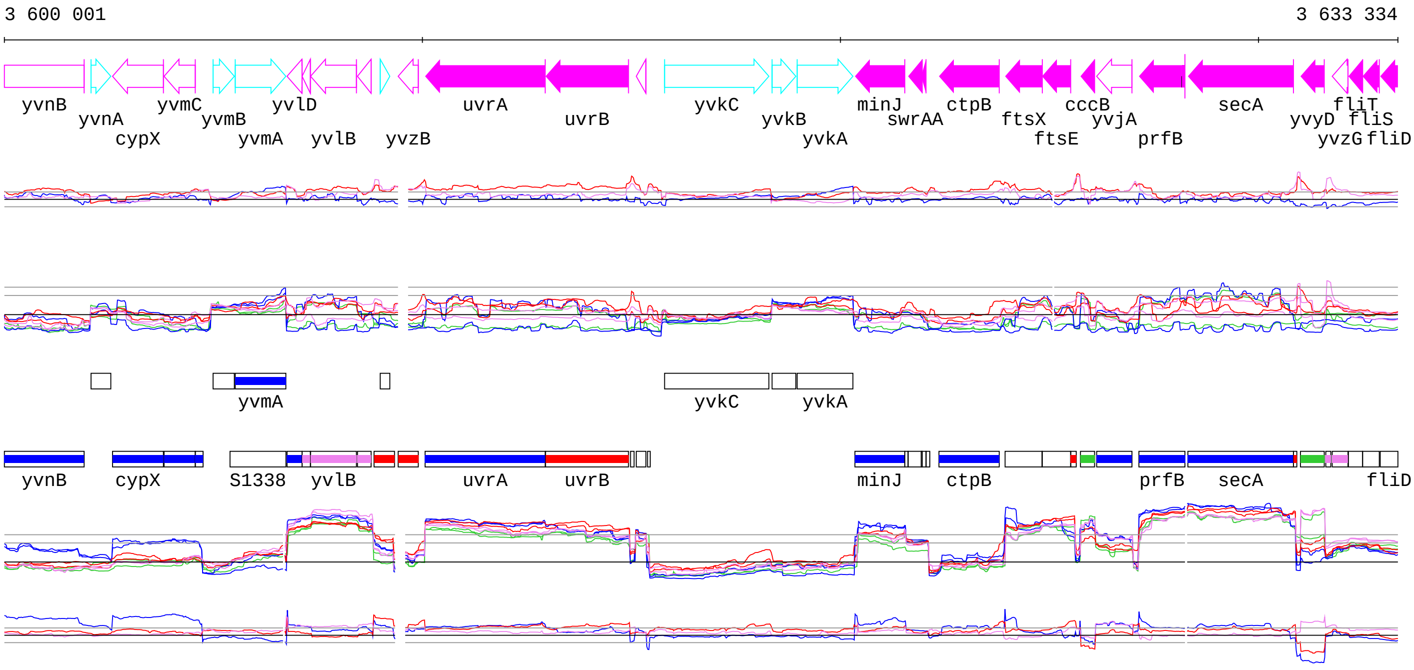

3 600 001
3 633 334
yvnB yvnA cypX yvmC yvmB yvmA yvlD yvlB yvzB uvrA uvrB yvkC yvkB yvkA minJ swrAA ctpB ftsX ftsE cccB yvjA prfB secA yvyD fliT yvzG fliS fliD
yvmA
yvkC yvkA
yvnB cypX S1338 yvlB uvrA uvrB minJ ctpB prfB secA fliD

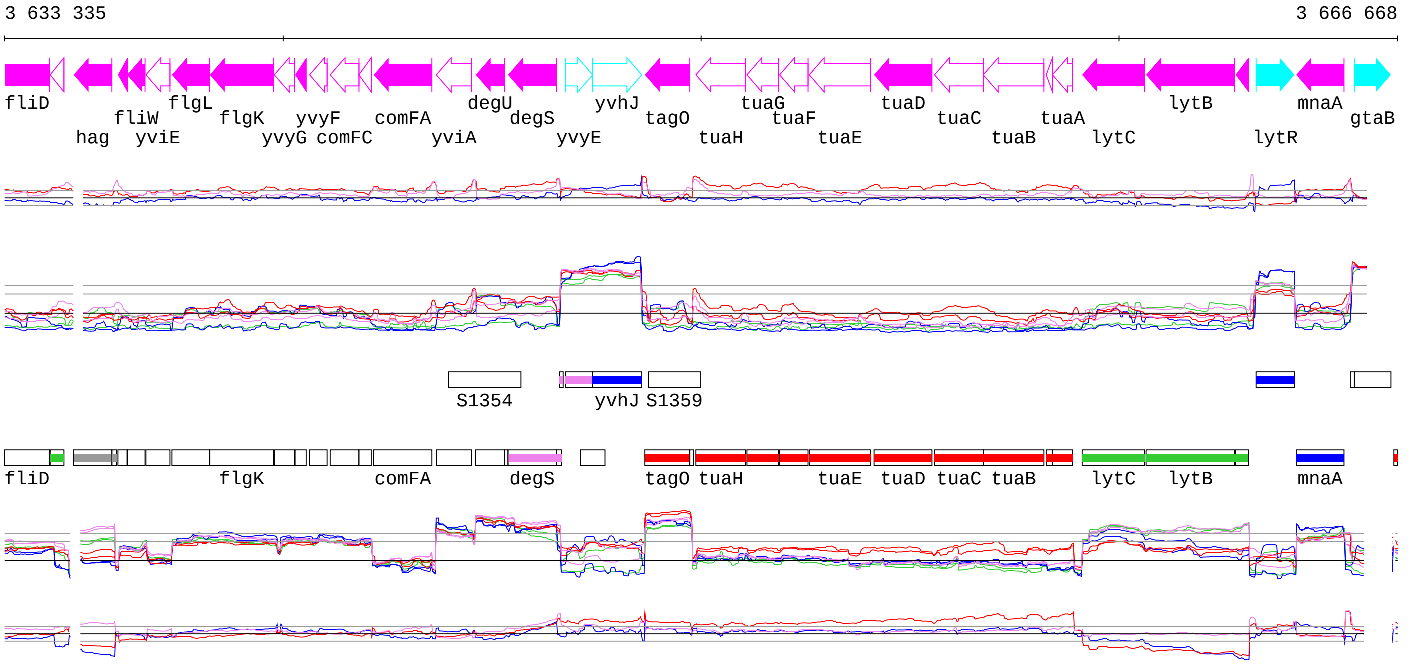

3 633 335
3 666 668
fliD hag fliW yviE flgL flgK yvyG yvyF comFC comFA yviA degU degS yvyE yvhJ tagO tuaH tuaG tuaF tuaE tuaD tuaC tuaB tuaA lytC lytB lytR mnaA gtaB
S1354 yvhJ S1359
fliD flgK comFA degS tagO tuaH tuaE tuaD tuaC tuaB lytC lytB mnaA

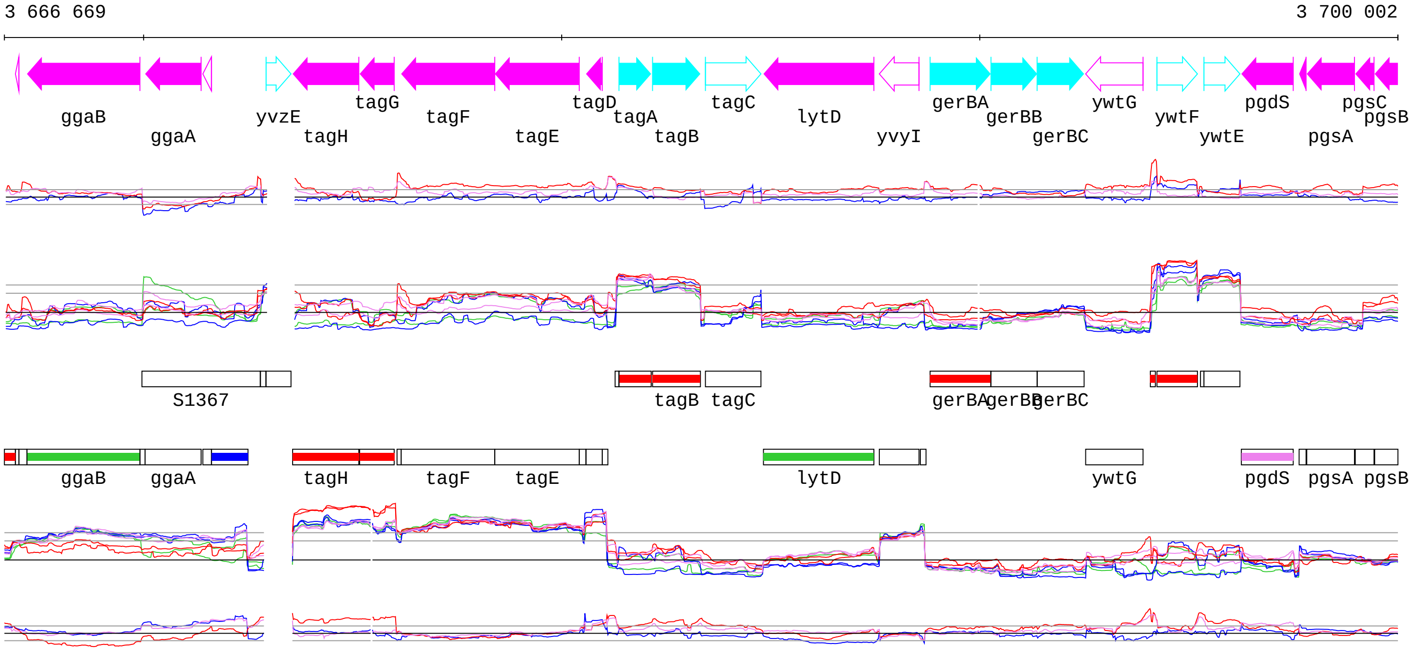

3 666 669
3 700 002
ggaB ggaA yvzE tagH tagG tagF tagE tagD tagA tagB tagC lytD yvyI gerBA gerBB gerBC ywtG ywtF ywtE pgdS pgsA pgsC pgsB
S1367
tagB tagC
gerBA gerBB gerBC
ggaB ggaA tagH tagF tagE lytD ywtG pgdS pgsA pgsB

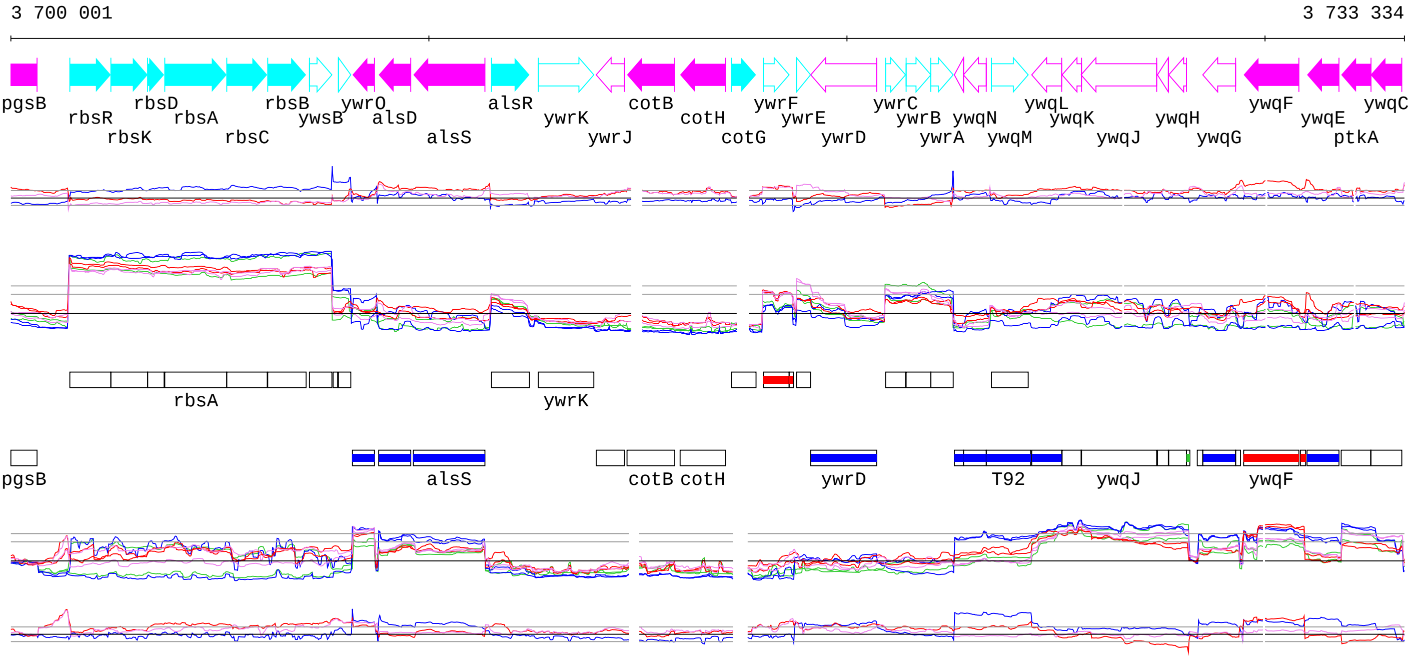
3 700 001
3 733 334
pgsB rbsR rbsK rbsD rbsA rbsC rbsB ywsB ywrO alsD alsS alsR ywrK ywrJ cotB cotH cotG ywrF ywrE ywrD ywrC ywrB ywrA ywqN ywqM ywqL ywqK ywqJ ywqH ywqG ywqF ywqE ptkA ywqC
rbsA ywrK
pgsB alsS cotB cotH ywrD T92 ywqJ ywqF

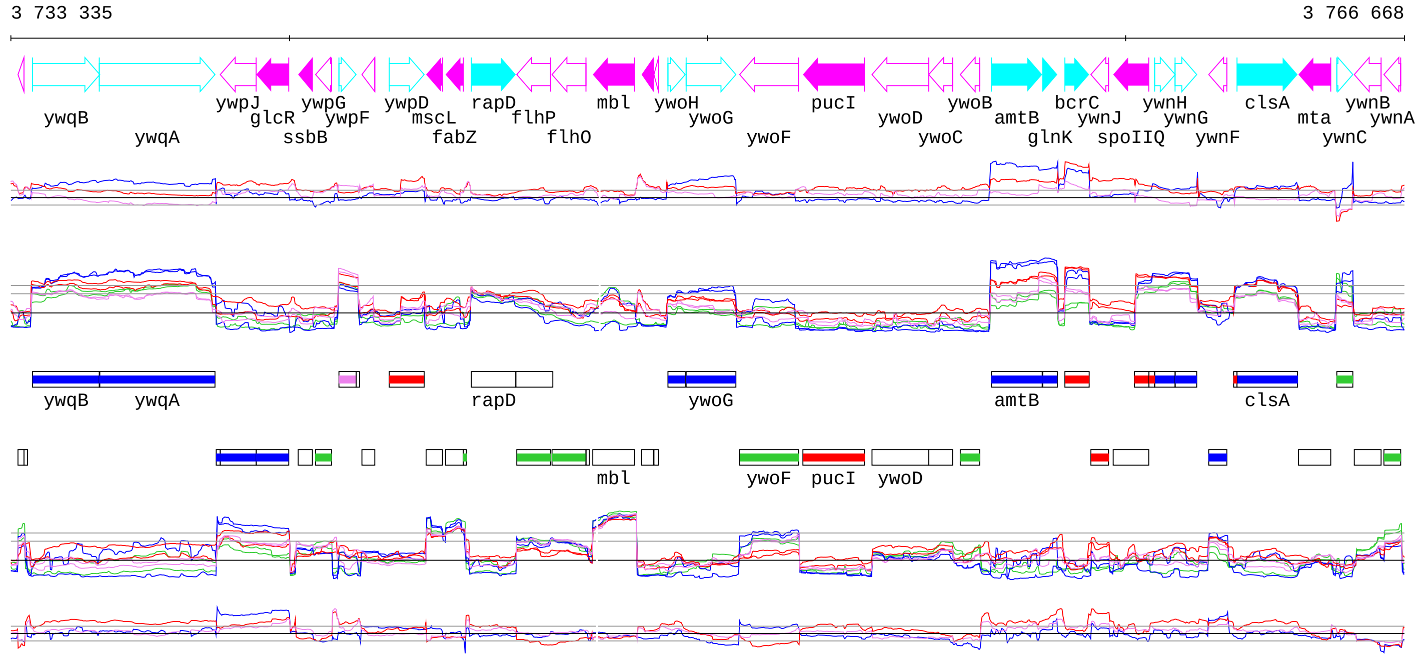
3 733 335
3 766 668
ywqB ywqA ywpJ glcR ssbB ywpG ywpF ywpD mscL fabZ rapD flhP flhO mbl ywoH ywoG ywoF pucI ywoD ywoC ywoB amtB glnK bcrC ywnJ spoIIQ ywnH ywnG ywnF clsA mta ywnC ywnB ywnA
ywqB ywqA rapD ywoG amtB clsA
mbl ywoF pucI ywoD

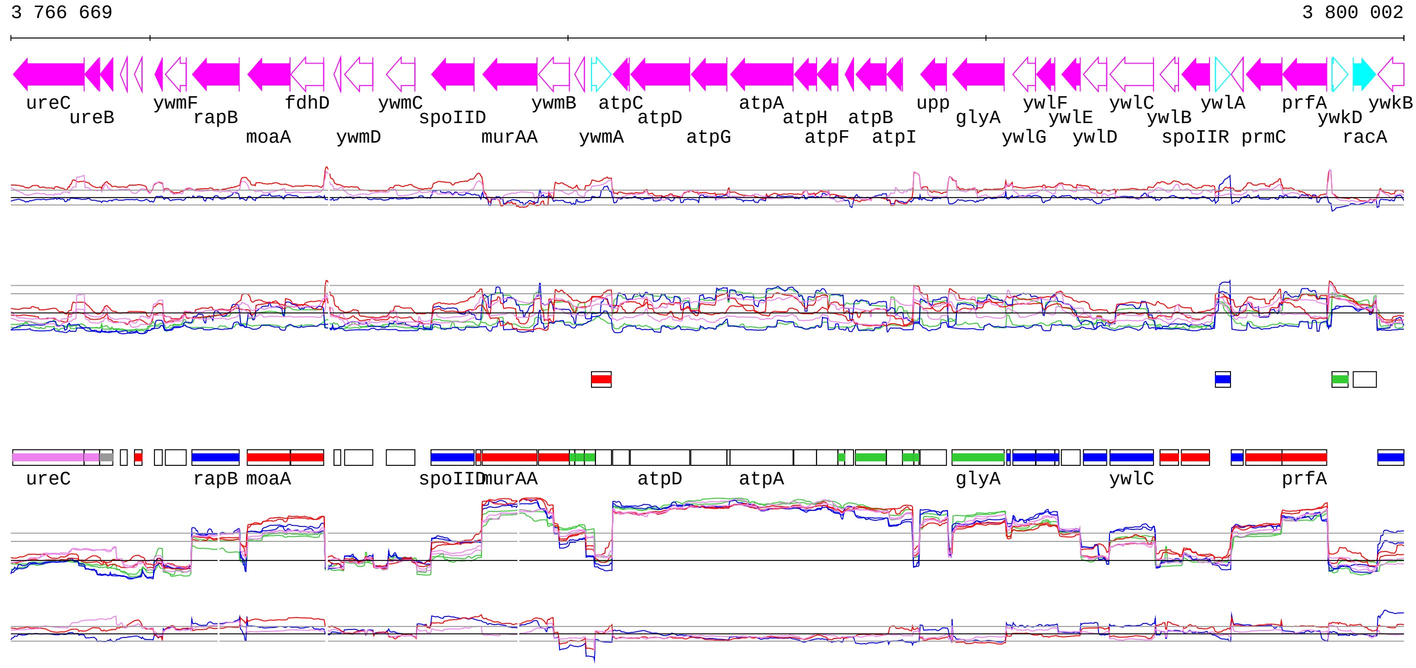
3 766 669
3 800 002
ureC ureB ywmF rapB moaA fdhD ywmD ywmC spoIID murAA ywmB ywmA atpC atpD atpG atpA atpH atpF atpB atpI upp glyA ywlG ywlF ywlE ywlD ywlC ywlB spoIIR ywlA prmC prfA ywkD racA ywkB
ureC rapB moaA spoIIDmurAA atpD atpA glyA ywlC prfA

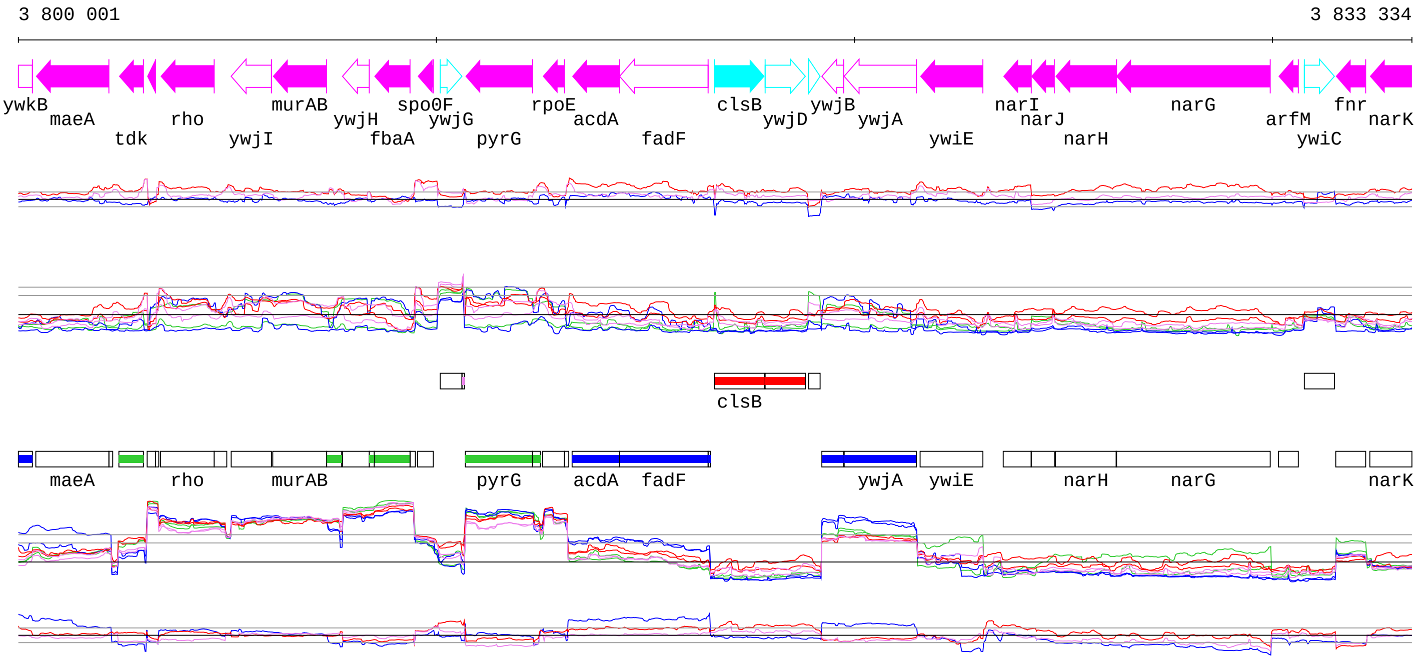
3 800 001
3 833 334
ywkB maeA tdk rho ywjI murAB ywjH fbaA spo0F ywjG pyrG rpoE acdA fadF clsB ywjD ywjB ywjA ywiE narI narJ narH narG arfM ywiC fnr narK
clsB
maeA rho murAB pyrG acdA fadF ywjA ywiE narH narG narK

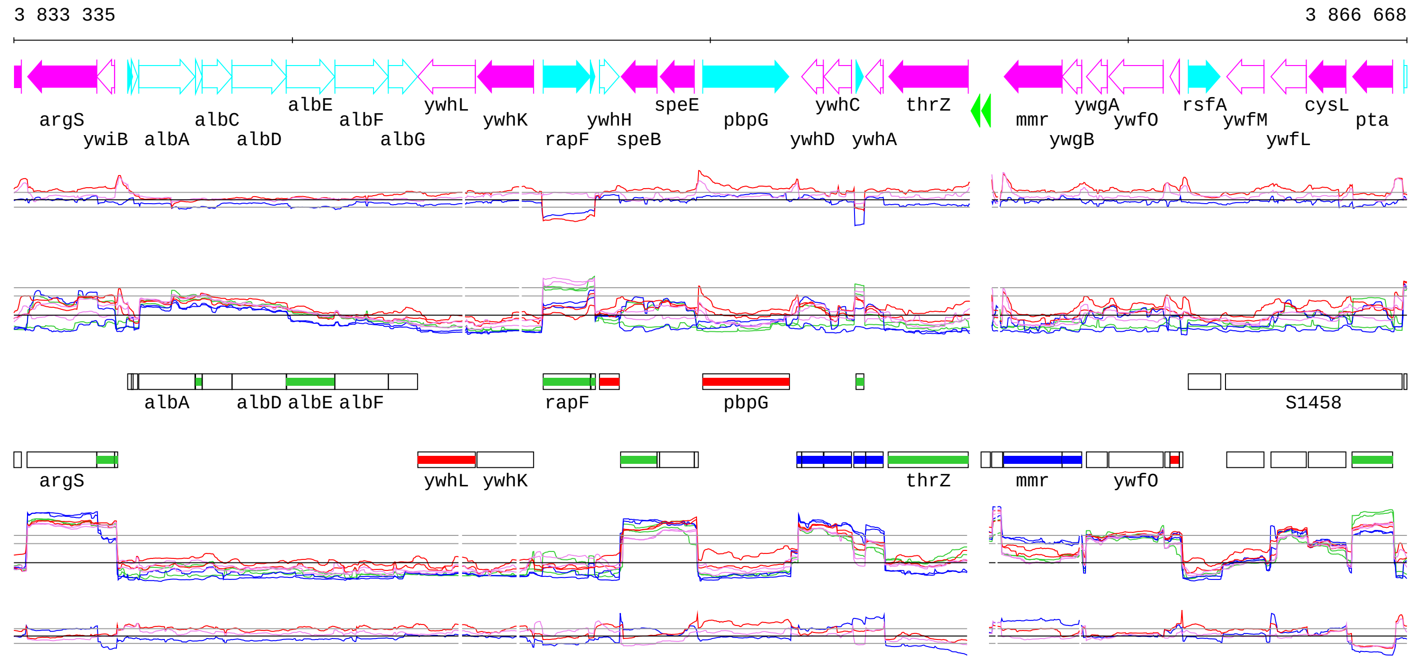
3 833 335
3 866 668
argS ywiB albA albC albD albE albF albG ywhL ywhK ywhH rapF speE speB pbpG ywhD ywhC ywhA thrZ mmr ywgB ywgA ywfO rsfA ywfM ywfL cysL pta
albA albD albE albF rapF pbpG S1458
argS ywhL ywhK thrZ mmr ywfO

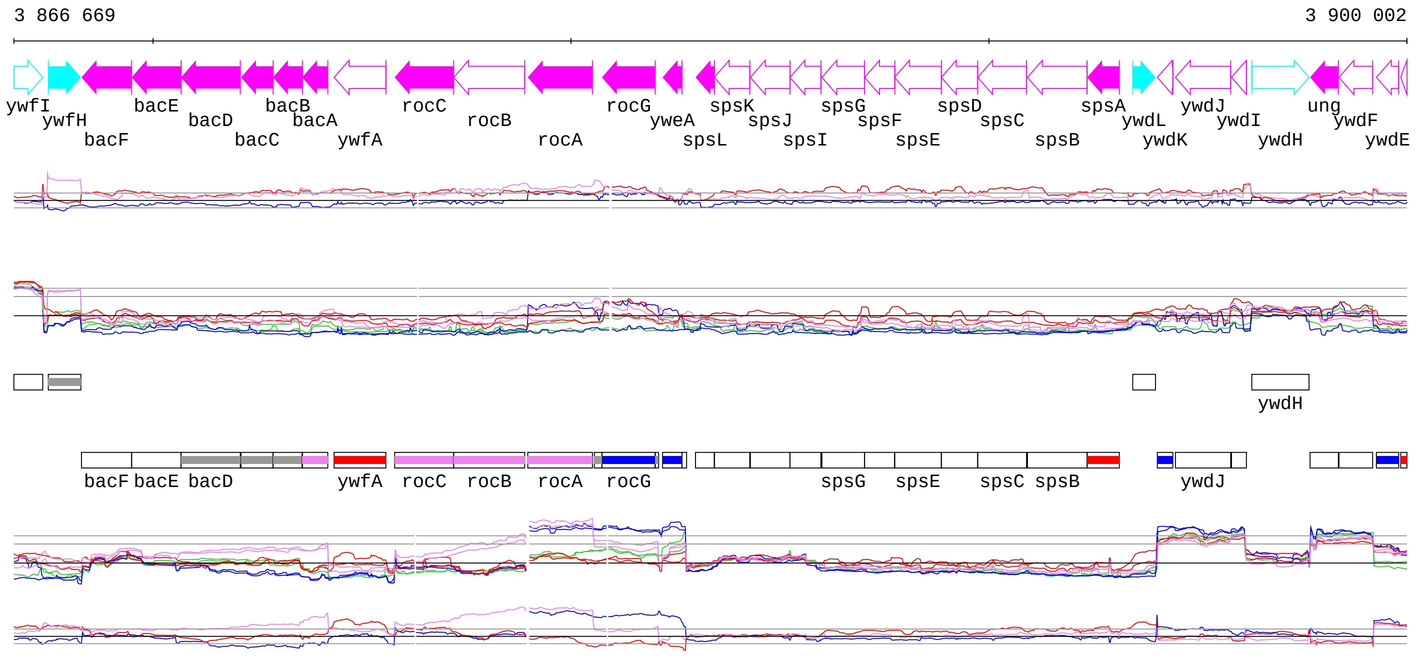
3 866 669
3 900 002
ywfI ywfH bacF bacE bacD bacC bacB bacA ywfA rocC rocB rocA rocG yweA spsK spsL spsJ spsI spsG spsF spsE spsD spsC spsB spsA ywdL ywdK ywdJ ywdI ywdH ung ywdF ywdE
ywdH
bacF bacE bacD ywfA rocC rocB rocA rocG spsG spsE spsC spsB ywdJ

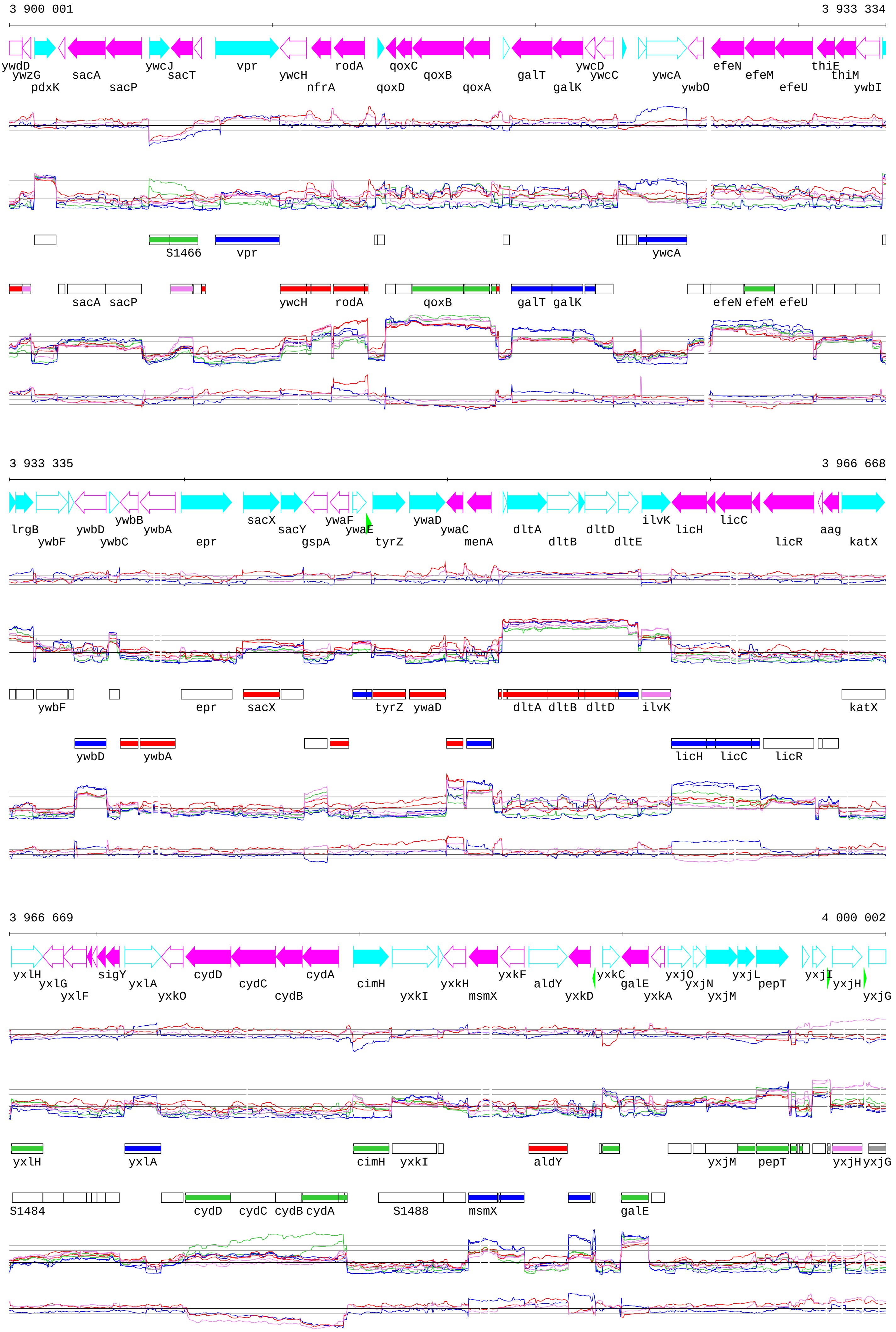
3 900 001
3 933 334
ywdD ywzG pdxK sacA sacP ywcJ sacT vpr ywcH nfrA rodA qoxD qoxC qoxB qoxA galT galK ywcD ywcC ywcA ywbO efeN efeM efeU thiE thiM ywbI
S1466 vpr ywcA
sacA sacP ywcH rodA qoxB galT galK efeN efeM efeU
3 933 335
3 966 668
lrgB ywbF ywbD ywbC ywbB ywbA epr sacX sacY gspA ywaF ywaE tyrZ ywaD ywaC menA dltA dltB dltD dltE ilvK licH licC licR aag katX
ywbF epr sacX tyrZ ywaD dltA dltB dltD ilvK katX
ywbD ywbA licH licC licR
3 966 669
4 000 002
yxlH yxlG yxlF sigY yxlA yxkO cydD cydC cydB cydA cimH yxkI yxkH msmX yxkF aldY yxkD yxkC galE yxkA yxjO yxjN yxjM yxjL pepT yxjI yxjH yxjG
yxlH yxlA cimH yxkI aldY yxjM pepT yxjH yxjG
S1484 cydD cydC cydB cydA S1488 msmX galE

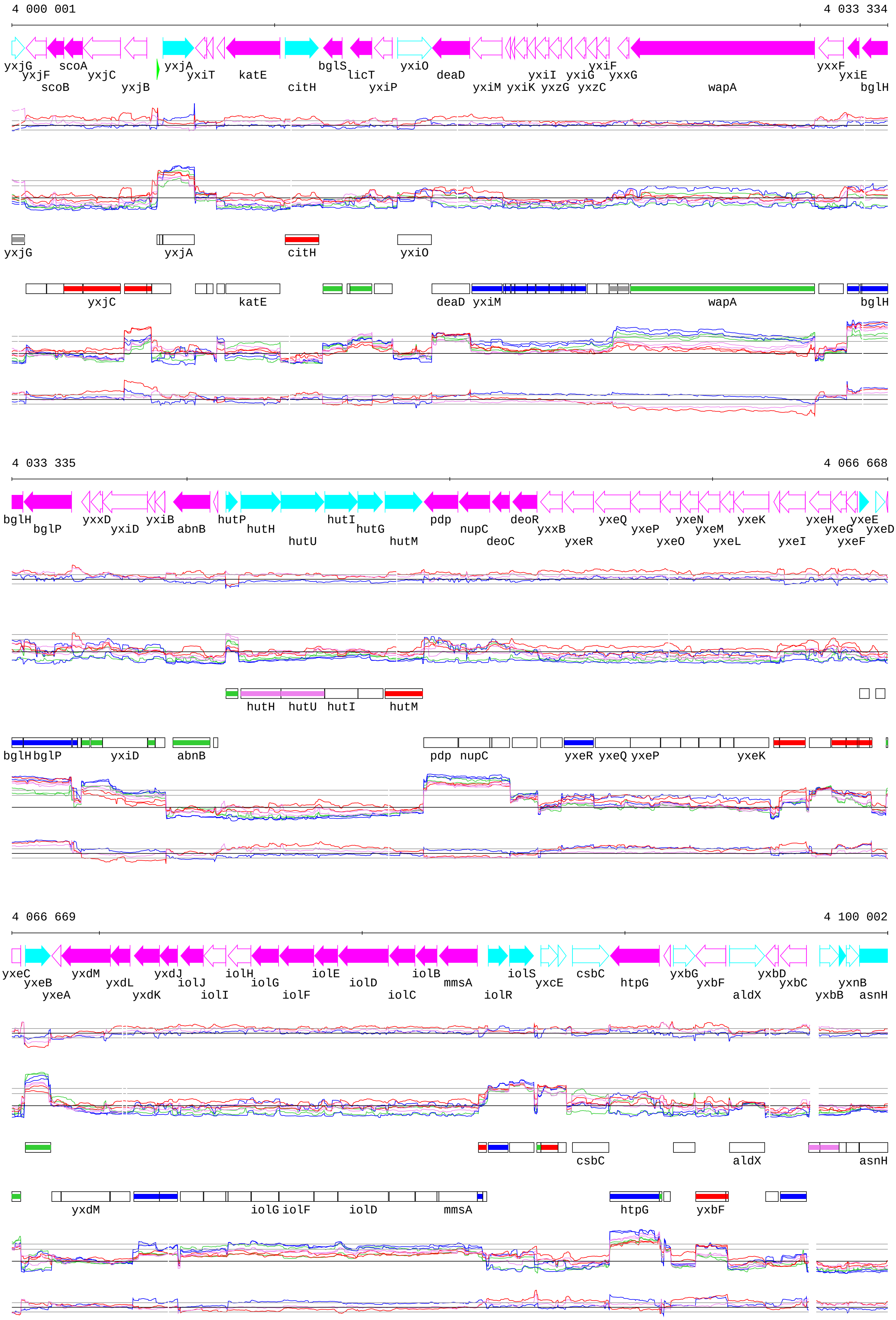

4 000 001
4 033 334
yxjG yxjF scoA scoB yxjC yxjB yxjA yxiT katE citH bglS licT yxiP yxiO deaD yxiM yxiK yxiI yxzG yxiG yxzC yxiF yxxG wapA yxxF yxiE bglH
yxjG yxjA citH yxiO
yxjC katE deaD yxiM wapA bglH
4 033 335
4 066 668
bglH bglP yxxD yxiD yxiB abnB hutP hutH hutU hutI hutG hutM pdp nupC deoC deoR yxxB yxeR yxeQ yxeP yxeO yxeN yxeM yxeL yxeK yxeI yxeH yxeG yxeF yxeE yxeD
hutH hutU hutI hutM
bglH bglP yxiD abnB pdp nupC yxeR yxeQ yxeP yxeK
4 066 669
4 100 002
yxeC yxeB yxeA yxdM yxdL yxdK yxdJ iolJ iolI iolH iolG iolF iolE iolD iolC iolB mmsA iolR iolS yxcE csbC htpG yxbG yxbF aldX yxbD yxbC yxnB yxbB asnH
csbC aldX asnH
yxdM iolG iolF iolD mmsA htpG yxbF

4 100 001 — 4 133 334

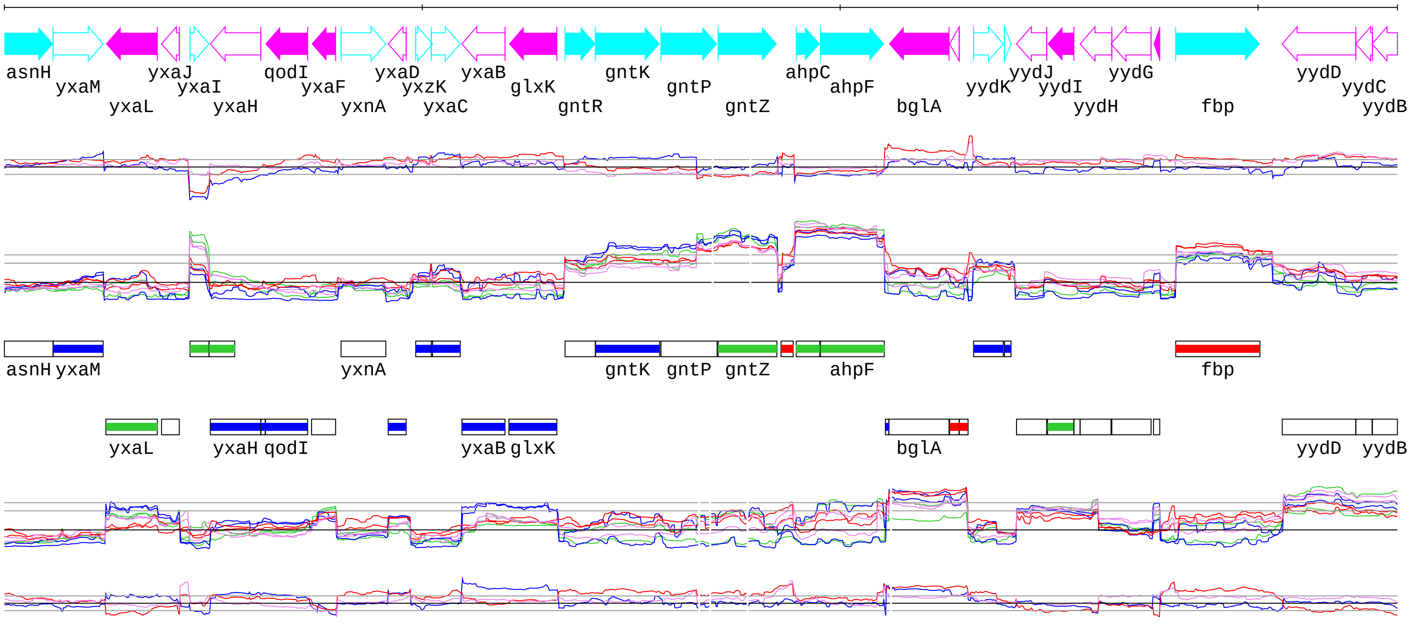

4 133 335 — 4 166 668

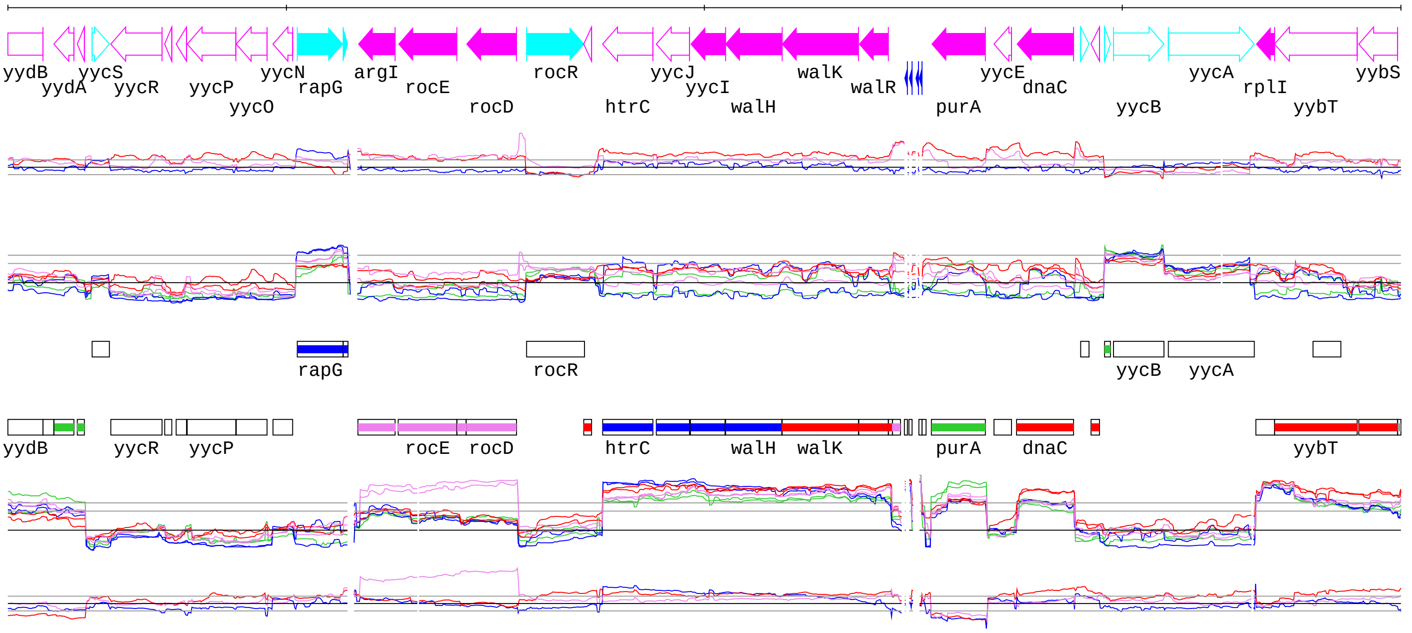

4 166 669 — 4 200 002

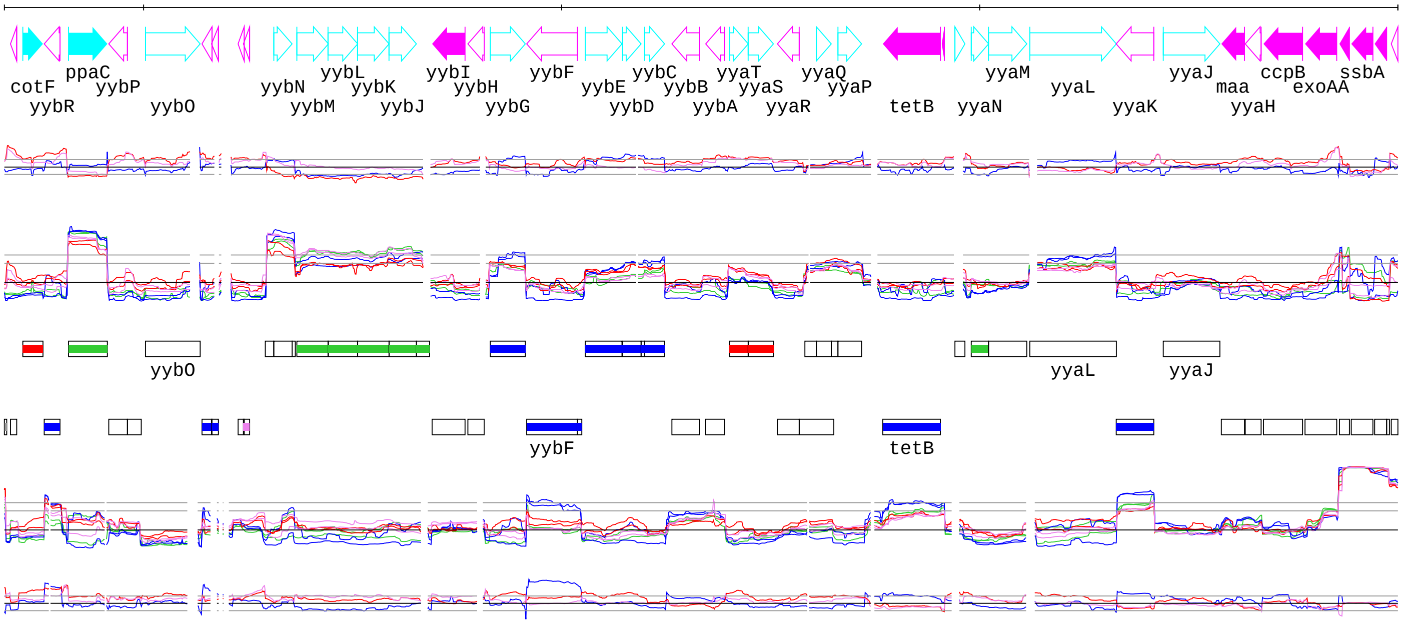

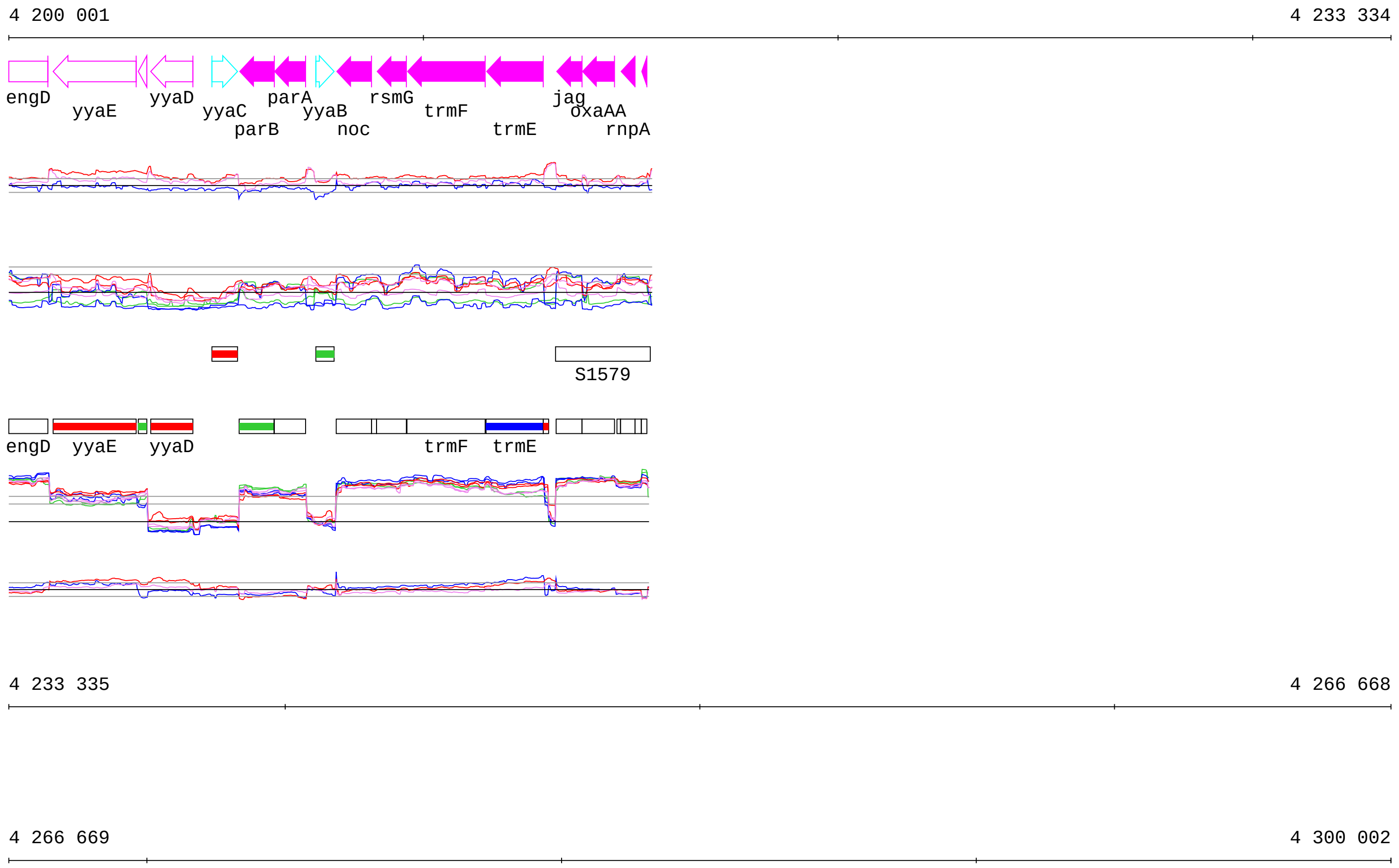
4 200 001
4 233 334
engD
yyaE
yyaD
yyaC
parB
parA
yyaB
noc
rsmG
trmF
trmE
jag
oxaAA
rnpA
S1579
engD
yyaE
yyaD
trmF
trmE
4 233 335
4 266 668
4 266 669
4 300 002
