# Supplementary figures and images for: Three Essential Ribonucleases—RNase Y, J1, and III—Control the Abundance of a Majority of Bacillus subtilis mRNAs
Source: PLoS Genet. 2012 Mar 8;8(3):e1002520. doi: 10.1371/journal.pgen.1002520 (PMC3297567; doi:10.1371/journal.pgen.1002520)

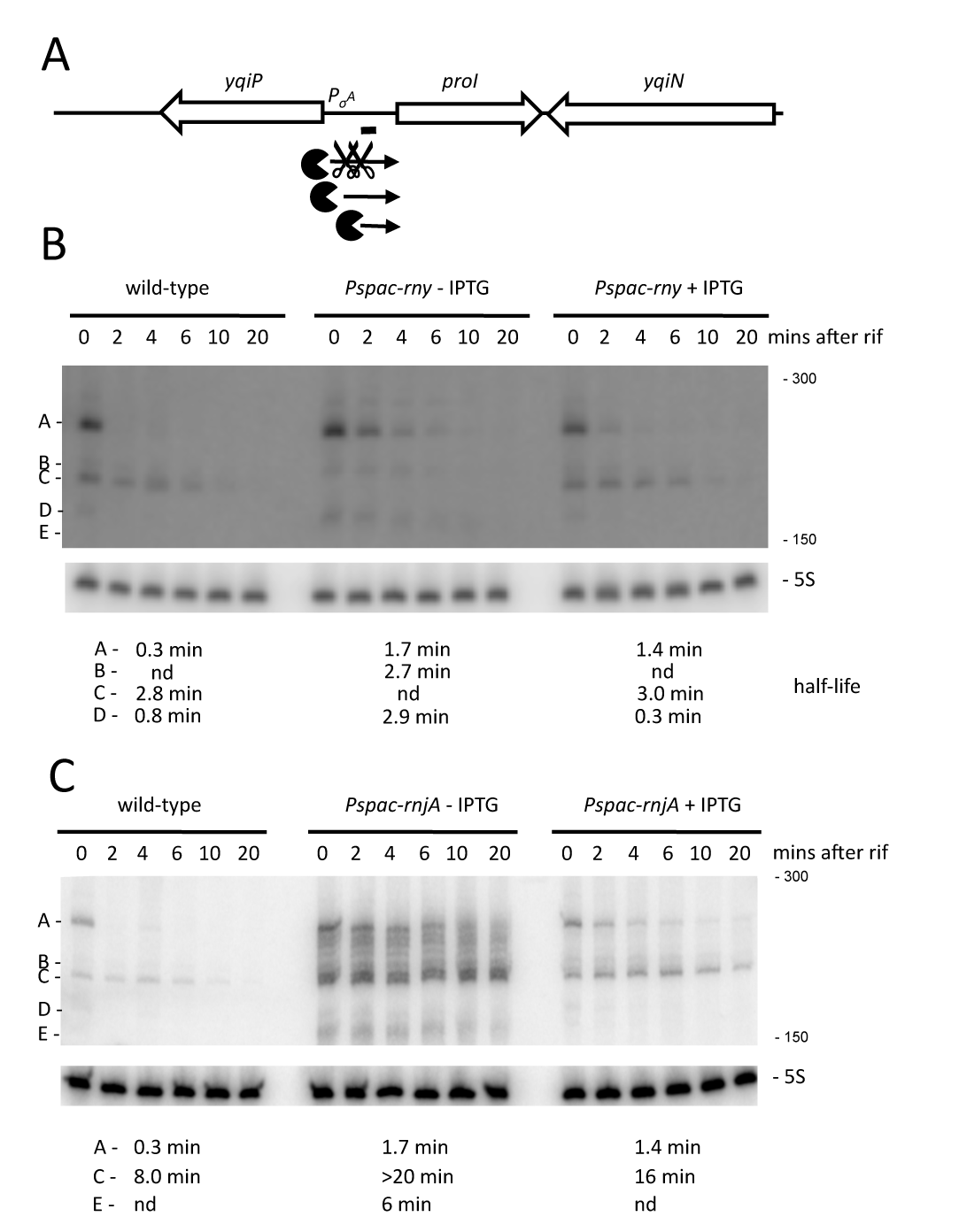

Supplement: Figure S2 — Degradation of the proI leader depends on RNases Y and J1. (A) Structure and predicted degradation pathway of the proI leader. ORFs are shown as large white arrows, transcript as a thin black arrow. Scissors indicate cleavage by RNase Y; ‘Pacman’ symbols represent 5′-3′ degradation by RNase J1. A short thick line indicates the position of the probe used. PσA indicates the approximate promoter position and relevant sigma factor mapped in [27]. (B) Northern blot of total mRNA isolated at times after addition of rifampicin (rif) from wild-type and RNase Y depleted (Pspac-rny−IPTG) and RNase Y induced (Pspac-rny+IPTG) cells. The blot was probed with 5′-labeled oligo CCB853 (Table S7) and reprobed with oligo HP246 against 5S rRNA. The half-lives of the different proI leader species (A, B... etc) are given under the Northern blot. Migration positions of RNA markers are shown to the right of the blot. (C) Northern blot of total mRNA isolated at times after addition of rifampicin (rif) from wild-type and RNase J1 depleted (Pspac-rnjA−IPTG) and RNase J1 induced (Pspac-rnjA+IPTG) cells. Description as in panel (B). (TIF) [file pgen.1002520.s002.tif]

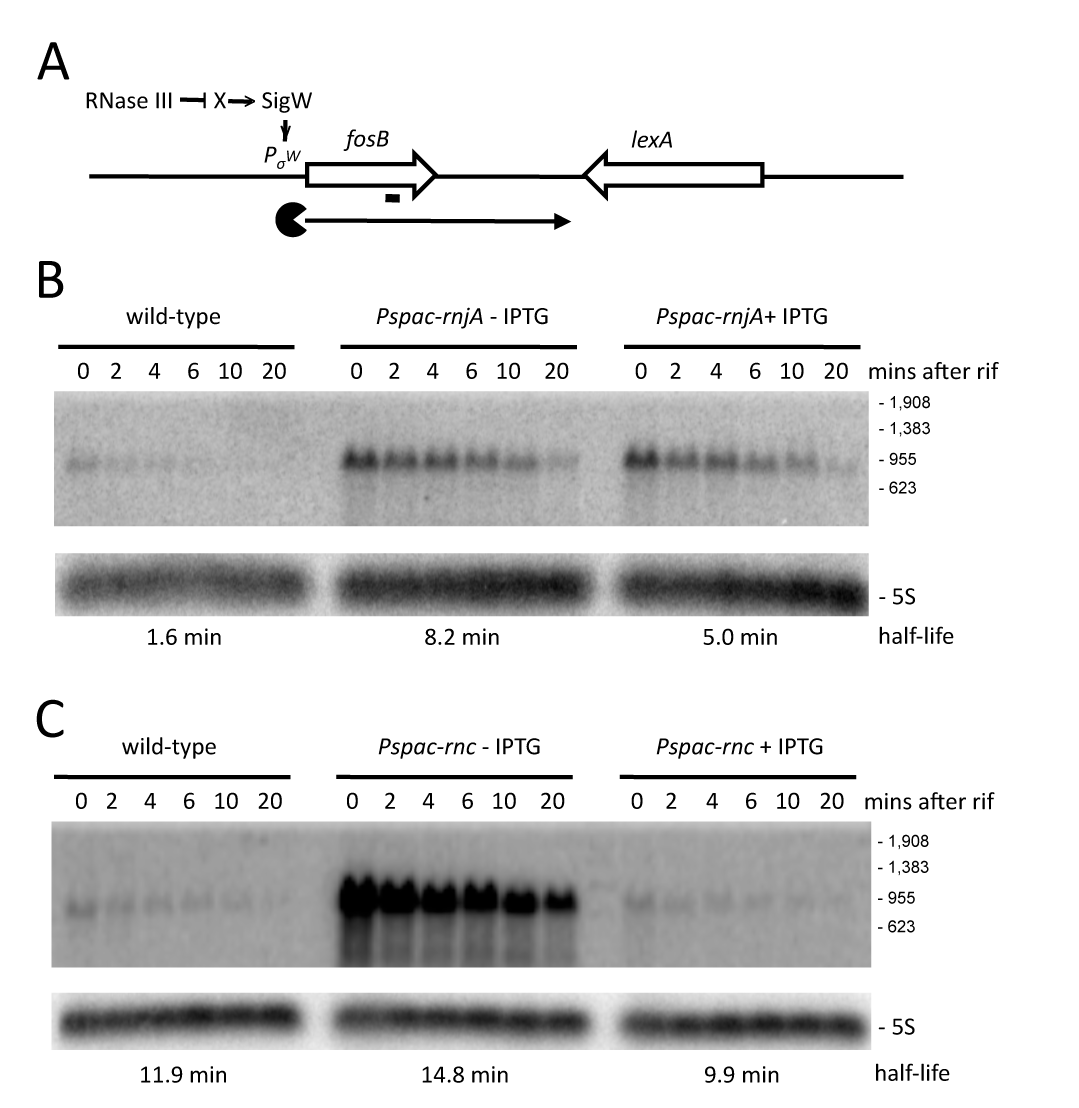

Supplement: Figure S3 — Degradation of the fosB mRNA depends on RNase J1 while its transcription is dependent on RNase III. (A) Structure and predicted degradation pathway of the fosB transcript. ORFs are shown as large white arrows, transcript as a thin black arrow. The ‘Pacman’ symbol represents 5′-3′ degradation by RNase J1. A short thick line indicates the position of the probe used. PσW indicates the approximate promoter position and relevant sigma factor mapped in [31]. The schematic also depicts RNase III initiated degradation of a transcript encoding an unknown factor X early in the SigW cascade. (B) Northern blot of total mRNA isolated at times after addition of rifampicin (rif) from wild-type and RNase J1 depleted (Pspac-rnjA−IPTG) and RNase J1 induced (Pspac-rnjA+IPTG) cells. The blot was probed with 5′-labeled oligo CCB813 (Table S7) and reprobed with oligo HP246 against 5S rRNA. The half-life of the fosB transcript is given under the Northern blot. Migration positions of RNA markers are shown to the right of the blot. (C) Northern blot of total mRNA isolated at times after addition of rifampicin (rif) from wild-type and RNase III depleted (Pspac-rnc−IPTG) and RNase III induced (Pspac-rnc+IPTG) cells. Description as in panel (B). (TIF) [file pgen.1002520.s003.tif]

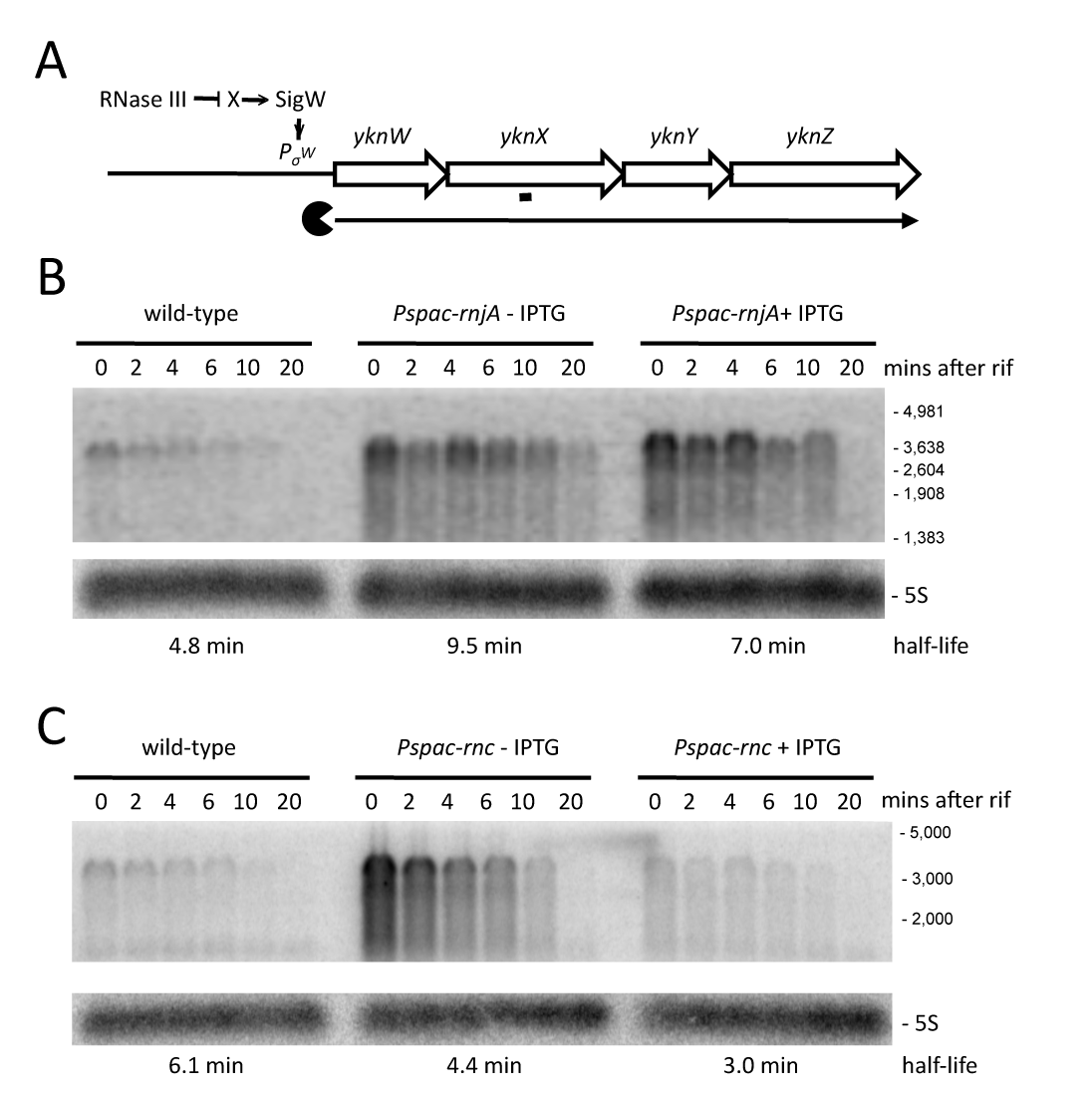

Supplement: Figure S4 — Degradation of the yknWXYZ mRNA depends on RNase J1 while its transcription is dependent on RNase III. (A) Structure and predicted degradation pathway of the yknWXYZ transcript. ORFs are shown as large white arrows, transcript as a thin black arrow. The ‘Pacman’ symbol represents 5′-3′ degradation by RNase J1. A short thick line indicates the position of the probe used. PσW indicates the approximate promoter position and relevant sigma factor mapped in [31]. The schematic also depicts RNase III initiated degradation of a transcript encoding an unknown factor X early in the SigW cascade. (B) Northern blot of total mRNA isolated at times after addition of rifampicin (rif) from wild-type and RNase J1 depleted (Pspac-rnjA−IPTG) and RNase J1 induced (Pspac-rnjA+IPTG) cells. The blot was probed with 5′-labeled oligo CCB814 (Table S7) and reprobed with oligo HP246 against 5S rRNA. The half-life of the yknWXYZ transcript is given under the Northern blot. Migration positions of RNA markers are shown to the right of the blot. (C) Northern blot of total mRNA isolated at times after addition of rifampicin (rif) from wild-type and RNase III depleted (Pspac-rnc−IPTG) and RNase III induced (Pspac-rnc+IPTG) cells. Description as in panel (B). (TIF) [file pgen.1002520.s004.tif]

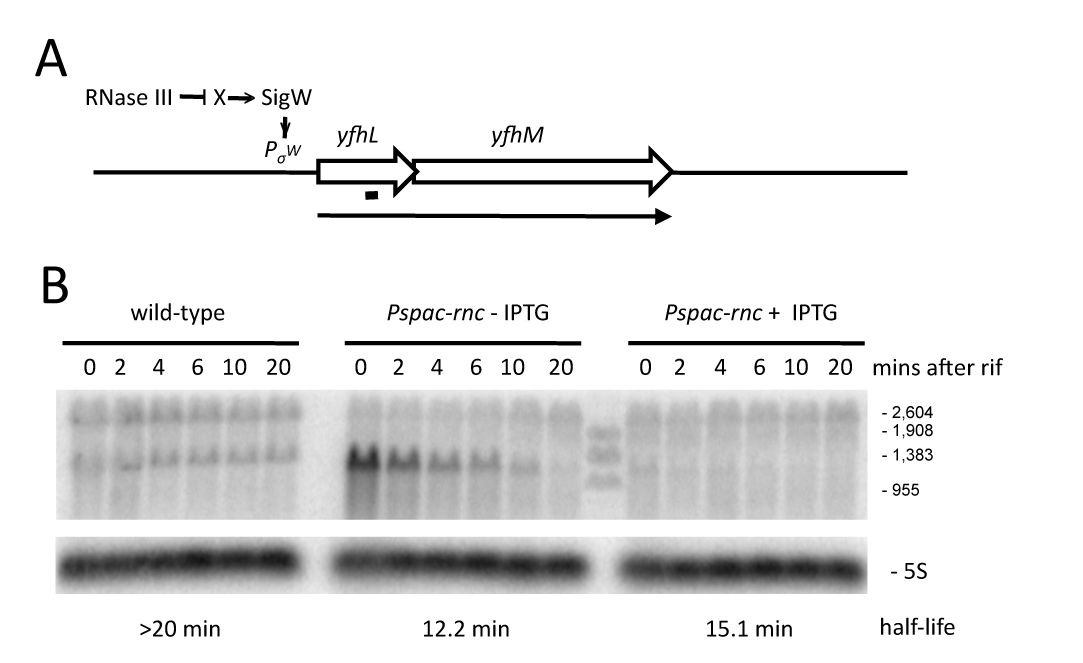

Supplement: Figure S5 — Transcription of the yfhLM mRNA is stimulated in an RNase III mutant. (A) Structure and predicted degradation pathway of the yfhLM transcript. ORFs are shown as large white arrows, transcript as a thin black arrow. A short thick line indicates the position of the probe used. PσW indicates the putative promoter and sigma factor for the transcript predicted by the DBTBS website (http://dbtbs.hgc.jp/) and in agreement with the size detected by Northern blot. The schematic also depicts RNase III initiated degradation of a transcript encoding an unknown factor X early in the SigW cascade. (B) Northern blot of total mRNA isolated at times after addition of rifampicin (rif) from wild-type and RNase III depleted (Pspac-rnc−IPTG) and RNase III induced (Pspac-rnc+IPTG) cells. The blot was probed with 5′-labeled oligo CCB809 (Table S7) and reprobed with oligo HP246 against 5S rRNA. The half-life of the yfhLM transcript is given under the Northern blot. Migration positions of RNA markers are shown to the right of the blot. (TIF) [file pgen.1002520.s005.tif]

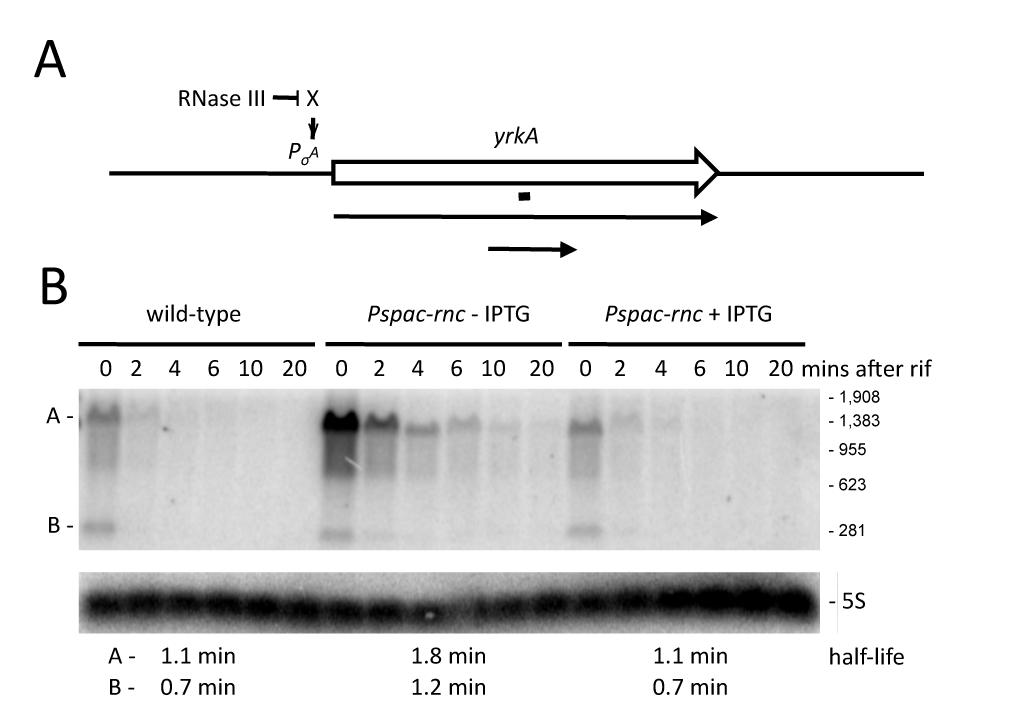

Supplement: Figure S6 — Transcription of the yrkA mRNA is stimulated in an RNase III mutant. (A) Structure and predicted degradation pathway of the yrkA transcript. ORFs are shown as large white arrows, transcript as a thin black arrow. A short thick line indicates the position of the probe used. PσA indicates the putative promoter and sigma factor for the transcript predicted by the DBTBS website (http://dbtbs.hgc.jp/) and in agreement with the size detected by Northern blot. The schematic also depicts RNase III initiated degradation of a transcript encoding an unknown factor X that activates yrkA transcription. (B) Northern blot of total mRNA isolated at times after addition of rifampicin (rif) from wild-type and RNase III depleted (Pspac-rnc−IPTG) and RNase III induced (Pspac-rnc+IPTG) cells. The blot was probed with 5′-labeled oligo CCB811 (Table S7) and reprobed with oligo HP246 against 5S rRNA. The half-lives of the yrkA transcripts (A, B) are given under the Northern blot. Migration positions of RNA markers are shown to the right of the blot. (TIF) [file pgen.1002520.s006.tif]

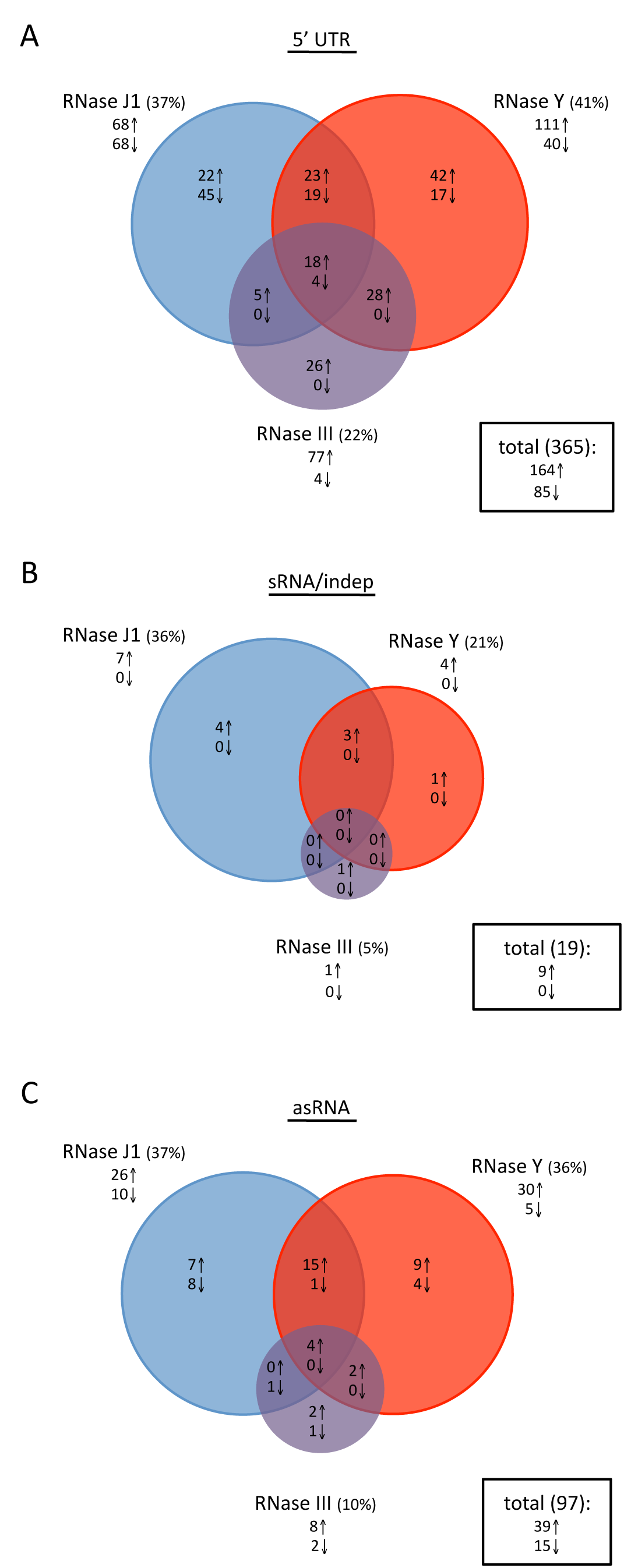

Supplement: Figure S7 — Effects of RNase J1, Y and III depletion on abundance of new B. subtilis segments. The Venn diagrams show the number of (A) 5′-UTRs, (B) potential sRNAs and (C) asRNAs altered in each of the three mutant strains CCB034 (RNase J1), CCB294 (RNase Y) and CCB288 (RNase III). Upward pointing arrows indicate the number of RNAs identified by Nicolas et al. (unpublished data) showing increased abundance; downward pointing arrows indicate decreased abundance. The areas of the circles are proportional to the number of RNAs showing altered abundance in each strain. The total number of RNAs affected in the experiment is shown in the rectangle to the right of each Venn diagram. (TIF) [file pgen.1002520.s007.tif]

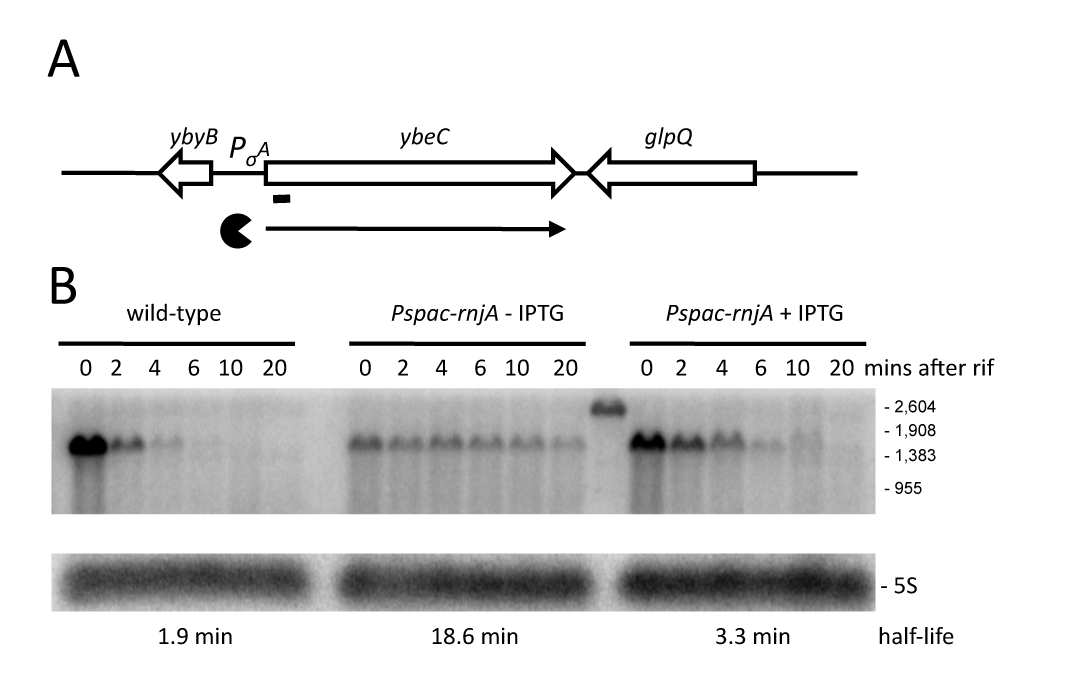

Supplement: Figure S8 — Both transcription and degradation of the ybeC transcript are dependent on RNase J1. (A) Structure and predicted degradation pathway of ybeC. ORFs are shown as large white arrows, transcript as a thin black arrow. The ‘Pacman’ symbol represents 5′-3′ degradation by RNase J1. A short thick line indicates the position of the probe used. PσA indicates the putative promoter and sigma factor for the transcript predicted by the DBTBS website (http://dbtbs.hgc.jp/) and in agreement with the size detected by Northern blot. (B) Northern blot of total mRNA isolated at times after addition of rifampicin (rif) from wild-type and RNase J1 depleted (Pspac-rnjA−IPTG) and RNase Y induced (Pspac-rnjA+IPTG) cells. The blot was probed with 5′-labeled oligo CCB825 (Table S7) and reprobed with oligo HP246 against 5S rRNA. The half-life of the transcript is given under the Northern blot. Migration positions of RNA markers are shown to the right of the blot. (TIF) [file pgen.1002520.s008.tif]

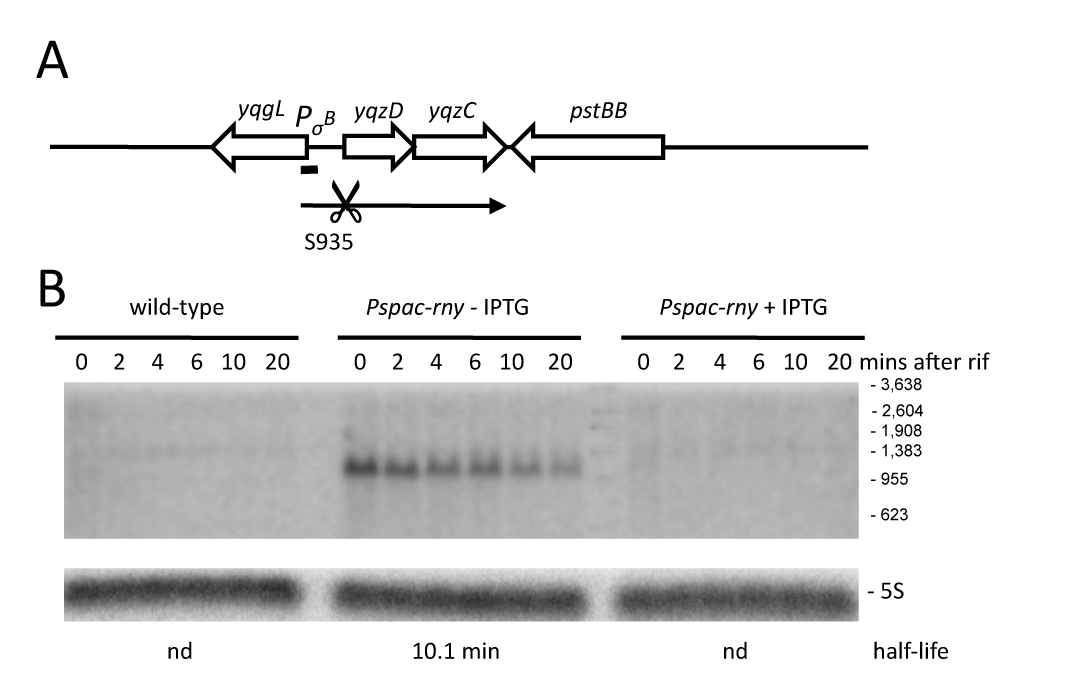

Supplement: Figure S9 — Degradation of the 5′-UTR (S935) of the yqzDC operon mRNA is dependent on RNase Y. (A) Structure and predicted degradation pathway of S935. ORFs are shown as large white arrows, transcript as a thin black arrow. Scissors indicate cleavage by RNase Y; ‘Pacman’ symbols represent 5′-3′ degradation by RNase J1. A short thick line indicates the position of the probe used. PσB indicates the putative promoter and sigma factor for the transcript predicted by the DBTBS website (http://dbtbs.hgc.jp/) and in agreement with the size detected by Northern blot. (B) Northern blot of total mRNA isolated at times after addition of rifampicin (rif) from wild-type and RNase Y depleted (Pspac-rny−IPTG) and RNase Y induced (Pspac-rny+IPTG) cells. The blot was probed with 5′-labeled oligo CCB821 (Table S7) and reprobed with oligo HP246 against 5S rRNA. The half-life of the transcript is given under the Northern blot. Migration positions of RNA markers are shown to the right of the blot. (TIF) [file pgen.1002520.s009.tif]
