# Supplementary material for: Three Essential Ribonucleases—RNase Y, J1, and III—Control the Abundance of a Majority of Bacillus subtilis mRNAs
Source: PLoS Genet. 2012 Mar 8;8(3):e1002520. doi: 10.1371/journal.pgen.1002520 (PMC3297567; doi:10.1371/journal.pgen.1002520)
Supplement: Table S1 — Expression levels of rnc, rnjA, rny and downstream genes compared to wild-type. (DOCX) [file pgen.1002520.s010.docx]

**Table S1. Expression levels of *rnc, rnjA, rny* and downstream genes compared to wild-type.**

| Strain | gene, downstream gene | -IPTG | +IPTG |
| --- | --- | --- | --- |
| CCB288 | *rnc, smc* | 0.023, 1.20 | 1.44, 2.48 |
| CCB034 | *rnjA* | 0.013 | 0.17 |
| CCB294 | *rny, ymdB* | 0.020, 0.82 | 1.11, 1.34 |
| CCB012 (polar) | *rny, ymdB* | 0.008, 0.028 | 0.40, 0.26 |
