# Supplementary material for: Three Essential Ribonucleases—RNase Y, J1, and III—Control the Abundance of a Majority of Bacillus subtilis mRNAs
Source: PLoS Genet. 2012 Mar 8;8(3):e1002520. doi: 10.1371/journal.pgen.1002520 (PMC3297567; doi:10.1371/journal.pgen.1002520)
Supplement: Table S6 — Oligonucleotides used in this study. Hybridizing sequences in first PCR cycle are in upper case; non-hybridizing sequences are lower case. (DOCX) [file pgen.1002520.s015.docx]

**Table S6. Oligonucleotides used in this study.**

| *Name* | *Sequence* |
| --- | --- |
| CC768 | GCGCTGATAGACGGAAAACTG |
| CC770 | GACTGCTAGTTCTTTGCCGTTTGC |
| CC774 | cttgttactagtCGCAAAAAAGCTATGAAAAGATGTTTACGCCAGGGAGC |
| CC775 | cttgttactagtGCCGTTTGCTTTCACATATGTGATCCCTTTCCC |
| CC797 | GATATTTATATGAGCGCTATAGATATGTACAAATTTGACC |
| CC798 | CAACCAAGTTCATAGCAAGAGGAGGTGAAAGTatgaatacatacgaacaaattaataaagtgaaaaaaatacttcgg |
| CC799 | ccgaagtatttttttcactttattaatttgttcgtatgtattcatACTTTCACCTCCTCTTGCTATGAACTTGGTTG |
| CC800 | gtaaatttaactataaactatttaaataacagattaaaaaaattataaAGTGATGCGCTAAGCATCACTTTATTTTTTTGAC |
| CC801 | GTCAAAAAAATAAAGTGATGCTTAGCGCATCACTttataatttttttaatctgttatttaaatagtttatagttaaatttac |
| CC802 | atatatgaattcGAACGTTGCTCTAGAATAATTCTACAC |
| CC803 | atatatgcatgcCCTTTCTTCTTGAAAATTCCTTGCCGTC |
| CC807 (*yjoB*) | CCGAAAGTGCCGTAGCCCGCCGCAGCTCTTTC |
| CC809 (*yfhL*) | GCAAACCCATTCCCGCAATCACCAAGCCTGACAG |
| CC811 (*yrkA*) | CGATCTCTCTTCTCGGAATCATAATTTCTTTGGCG |
| CC813 (*fosB*) | CTGGTTCTTCATTAAGCGCTAGCCAGATGCCGTTC |
| CC814 (*yknX*) | CTAAATCTGACACCCGGTTGGCAAGTGACTGCCGC |
| CC821 (*yqzD* 5') | GGAAAGGAGAATGTTAGGAGTCCATGGATAAACAC |
| CC825 (*ybeC*) | GAACAGCCAGCCTGAACCGATCATCGACCCGAGCC |
| CC826 (*spoIISA*) | GCATAGGTTTTCAGCAGCTTATCAATACCTTCAGC |
| CC832 (*mreBH*) | GAAACGATATCCTCATCAAGCTGGTCTCCGCCGAT |
| CC852 (*yrzFH* ig) | CATTTGTTCAGGCGACAGCTTAATTCGATAGCCTCTC |
| CC853 (*proI* 5') | GCTCTCTTAGGCCATTGCCTTGTATACTATTCCTCGTC |
| CC854 (*yhbF* ig) | CACCGTCTCTCCCCCTCTTCAATTTTTCCGTGCTGTC |
| CC857 (as *yknT*) | GCAGGAGCTTCTATCTGCTGAAACCTATAAACGCAGAGCCG |
| CC859 (as *yfkF* | CTTGGCTTTGCCGCAGGCCCGTTCATGGTGCCGCTTG |
| CC900 (*sigW*) | CTAAGAGCCTCTCTGCCTCTGTGAATCCGCG |
| HP246 (5S rRNA) | ATCGGCGCTGAAGAGCTTAACTTCC |

Hybridizing sequences in first PCR cycle are in upper case; non-hybridizing sequences are lower case.
